# The hornwort genome and early land plant evolution

**DOI:** 10.1038/s41477-019-0588-4

**Published:** 2020-02-10

**Authors:** Jian Zhang, Xin-Xing Fu, Rui-Qi Li, Xiang Zhao, Yang Liu, Ming-He Li, Arthur Zwaenepoel, Hong Ma, Bernard Goffinet, Yan-Long Guan, Jia-Yu Xue, Yi-Ying Liao, Qing-Feng Wang, Qing-Hua Wang, Jie-Yu Wang, Guo-Qiang Zhang, Zhi-Wen Wang, Yu Jia, Mei-Zhi Wang, Shan-Shan Dong, Jian-Fen Yang, Yuan-Nian Jiao, Ya-Long Guo, Hong-Zhi Kong, An-Ming Lu, Huan-Ming Yang, Shou-Zhou Zhang, Yves Van de Peer, Zhong-Jian Liu, Zhi-Duan Chen

**Affiliations:** 1 State Key Laboratory of Systematic and Evolutionary Botany, Institute of Botany, Chinese Academy of Sciences, Beijing, China; 2 University of Chinese Academy of Sciences, Beijing, China; 3 PubBio-Tech Services Corporation, Wuhan, China; 4 Key Laboratory of Southern Subtropical Plant Diversity, Fairy Lake Botanical Garden, Shenzhen & Chinese Academy of Science, Shenzhen, China; 5 BGI-Shenzhen, Shenzhen, China; 6 Key Laboratory of National Forestry and Grassland Administration for Orchid Conservation and Utilization at College of Landscape Architecture, Fujian Agriculture and Forestry University, Fuzhou, China; 7 Department of Plant Biotechnology and Bioinformatics, Ghent University, Ghent, Belgium; 8 VIB Center for Plant Systems Biology, Ghent, Belgium; 9 Department of Biology, Huck Institutes of the Life Sciences, Pennsylvania State University, University Park, PA USA; 10 Department of Ecology and Evolutionary Biology, University of Connecticut, Storrs, CT USA; 11 Key Laboratory for Plant Diversity and Biogeography of East Asia, Kunming Institute of Botany, Chinese Academy of Sciences, Kunming, China; 12 Center for Plant Diversity and Systematics, Institute of Botany, Jiangsu Province and Chinese Academy of Sciences, Nanjing, China; 13 Sino–Africa Joint Research Center, Chinese Academy of Sciences, Wuhan, China; 14 College of Forestry and Landscape Architecture, South China Agricultural University, Guangzhou, China; 15 Center for Microbial Ecology and Genomics, Department of Biochemistry, Genetics and Microbiology, Pretoria, South Africa; 16 College of Horticulture, Nanjing Agricultural University, Nanjing, China; 17 Fujian Colleges and Universities Engineering Research Institute of Conservation and Utilization of Natural Bioresources, College of Forestry, Fujian Agriculture and Forestry University, Fuzhou, China

**Keywords:** Plant evolution, Phylogenetics, Comparative genomics

## Abstract

Hornworts, liverworts and mosses are three early diverging clades of land plants, and together comprise the bryophytes. Here, we report the draft genome sequence of the hornwort *Anthoceros angustus*. Phylogenomic inferences confirm the monophyly of bryophytes, with hornworts sister to liverworts and mosses. The simple morphology of hornworts correlates with low genetic redundancy in plant body plan, while the basic transcriptional regulation toolkit for plant development has already been established in this early land plant lineage. Although the *Anthoceros* genome is small and characterized by minimal redundancy, expansions are observed in gene families related to RNA editing, UV protection and desiccation tolerance. The genome of *A. angustus* bears the signatures of horizontally transferred genes from bacteria and fungi, in particular of genes operating in stress-response and metabolic pathways. Our study provides insight into the unique features of hornworts and their molecular adaptations to live on land.

## Abstract (web summary)

A draft genome sequence of the hornwort *Anthoceros augustus* confirms the phylogenetic relationships among the three clades of bryophytes and provides insight into the unique characteristics of hornworts and their adaptations to live on land.

## Main

Land plants (Embryophyta) probably originated in the early Palaeozoic^1^, initiating the colonization of the terrestrial habitat. Because bryophytes (hornworts, liverworts and mosses) emerged from the early split in the diversification of land plants, they are key to the study of early land plant evolution (Supplementary Note 1.1). Unlike other extant land plants, the vegetative body of bryophytes is the haploid gametophyte, the sporophyte is always unbranched and permanently attached to the maternal plant, and both generations lack lignified vascular tissue^2^. Bryophytes occur in nearly all terrestrial habitats on all continents but are absent from marine environments^3^.

With only 200–250 species worldwide, the diversity of hornworts is much lower than that of the other six extant lineages of embryophytes (angiosperms, gymnosperms, ferns, lycophytes, mosses and liverworts)^4^. Long considered sister to all other land plants, or sister to all extant vascular plants, hornworts have recently been resolved as sister to the setaphytes (that is, the mosses and liverworts) within monophyletic bryophytes^1,5–8^. Still, hornworts possess a series of distinct features^9^. For instance, most hornworts have chloroplasts with CO_2_-concentrating pyrenoids, which have not been found in any other land plants but are widespread among green algae^10^. Other unusual features of hornworts include the persistent basal meristem in the sporophyte and mucilage-filled cavities for colonial symbionts on the gametophyte^11^. Most hornworts form tight symbiotic relationships with cyanobacteria^12^ and fungal endophytes (especially Glomeromycota and Mucoromycotina)^13^.

Here, we present the draft genome of *A. angustus* Steph. (Anthocerotaceae) (see Methods, Supplementary Figs. 1 and 2, and Supplementary Note 1.2). Completion of this high-quality hornwort genome complements previously sequenced representatives of the mosses (*Physcomitrella patens*^14^) and liverworts (*Marchantia polymorpha*^15^) and provides a unique opportunity to revisit bryophyte phylogeny, early land plant evolution and the adaptation of plants to live on land.

## Genome assembly and annotation

We sequenced the genome of *A. angustus* (a single individual of unknown sex from the dioecious species) using a combination of Illumina and Oxford Nanopore high-throughput sequencing systems (see Methods). We generated 126.53 Gb raw reads from Illumina and 63.61 Gb raw reads from Nanopore sequencing platforms, and retained 17.10 Gb and 3.78 Gb, respectively, after filtering, error-correction and decontamination (see Methods, Supplementary Figs. 2–4 and Supplementary Tables 1–3). Finally, we obtained an optimized assembly of 119 Mb with a contig N50 length of 796.64 kb and a scaffold N50 length of 1.09 Mb (Table 1 and Supplementary Table 4). Approximately 97.66% of the vegetative gametophyte transcriptome data for *A. angustus* genome annotation can be mapped to the assembled genome (Supplementary Table 5). Repeat sequences comprise 64.21% of the assembled genome, with transposable elements (TEs) being the major component (Table 1 and Supplementary Tables 6 and 7). Among the TEs, long terminal repeats (LTRs) are the most abundant (Supplementary Table 7). We used a combination of de novo, homology-based and RNA sequence-based predictions to obtain gene models for the *A. angustus* genome (Supplementary Table 8). In total, we predicted 14,629 protein-coding genes with an average coding-sequence length of 1.31 kb and an average of 4.81 exons per gene (Table 1, Supplementary Fig. 5 and Supplementary Table 8). About 85% of these predicted genes have their best hits on plant sequences from the National Center for Biotechnology Information (NCBI) non-redundant database (Supplementary Fig. 6), and 78.39% were functionally annotated through Swissprot, TrEMBL, Pfam, gene ontology (GO) and Kyoto Encyclopedia of Genes and Genomes (KEGG) (Supplementary Tables 9 and 32). Our annotation captured 89.64% of the 956 genes in the BUSCO plantae dataset^16^ (85.04% complete gene models plus 4.60% fragmented gene models), compared with 93.51% and 92.15% captured in *P. patens*^14^ and *M. polymorpha*^15^, respectively (Supplementary Table 10). In addition to protein-coding genes, we also identified 30 known mature micro RNAs (miRNAs), 180 novel mature miRNAs, 347 transfer RNAs, 94 ribosomal RNAs and 83 small nuclear RNAs (snRNAs) in the *A. angustus* genome (Supplementary Table 11). Nine mature miRNA sequences that appear conserved among land plants (miR156/157, miR159/319, miR160, miR165/166, miR170/171, miR408, miR477, miR535 and miR536)^17^ were also found in *A. angustus* (Supplementary Table 12).

**Table 1:** Assembly and annotation statistics of the draft genome of *A. angustus*

**Assembly features**	
Total length of scaffolds (bp)	119,333,152
Longest scaffold (bp)	3,809,330
N50 of scaffold (bp)	1,092,075
Total length of contigs (bp)	119,122,644
Longest contig (bp)	3,254,985
N50 of contig (bp)	796,636
GC ratio (%)	49.60
**Genome annotation**	
Number of protein-coding genes	14,629
Average gene or CDS length (bp)	1,972.11/1,313.24
Average exon/intron length (bp)	272.63/172.61
Average exon per gene	4.81
Average intron per gene	3.81
Total size of TEs (bp)	72,224,921
TEs in genome (%)	60.52

CDS, coding sequence.

## Comparative genomic analysis

**Fig. 1:**
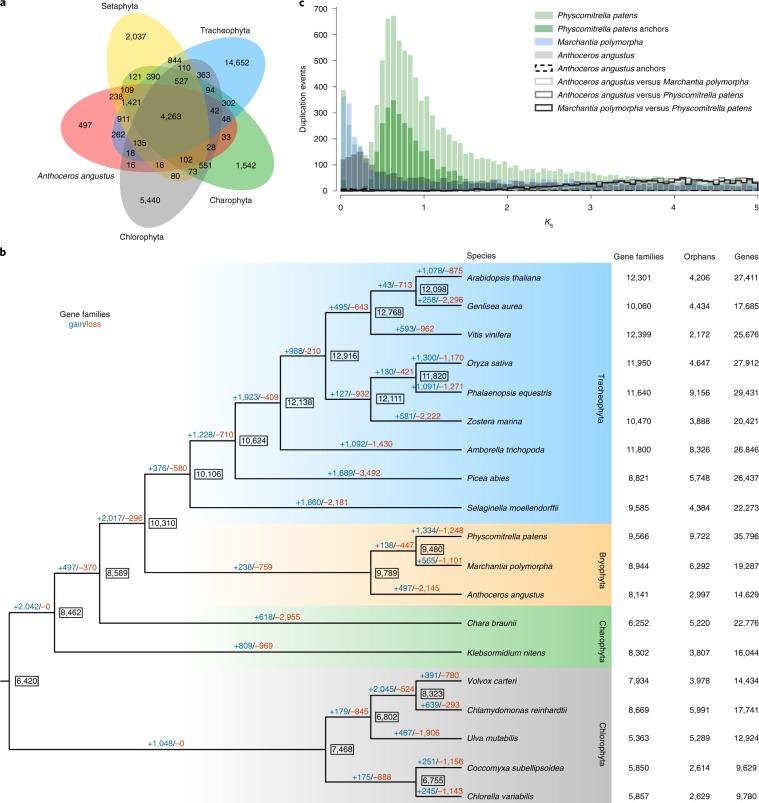
Comparative genomic analysis of *A. angustus* and 18 other plant species. **a**, Comparison of the number of gene families identified by OrthoMCL. The Venn diagram shows the shared and unique gene families in *A. angustus*, Setaphyta, Tracheophyta, Charophyta and Chlorophyta. The gene-family number is listed in each of the components. **b**, Gene-family gain (+)/loss (−) among 19 green plants. The numbers of gained (blue) and lost (red) gene families are shown above the branches. The boxed number indicates the gene-family size at each node. The number of gene families, orphans (single-copy gene families) and number of predicted genes is indicated next to each species. **c**, Comparison of whole paranome, anchor pair and one-to-one orthologue distribution of the number of synonymous substitutions per synonymous site (*K*_S_) across the three bryophyte species (*P. patens*, *M. polymorpha* and *A. angustus*).

For sequence similarity-based clustering of homologues, we used the predicted proteomes of *A. angustus* and 18 other green plants with fully-sequenced genomes (that is, 11 other land plants, two charophyte green algae and five chlorophyte green algae; Supplementary Table 13). Genes of *A. angustus* are distributed among 7,644 gene families that are shared with other plants, and 497 gene families that appear to be unique to *A. angustus* (Fig. 1a and Supplementary Table 14). In the shared gene families, most *A. angustus* genes (that is, 9,680) cluster with land plant genes, and only a very small number (that is, 107) specifically cluster with green algae genes (Supplementary Fig. 7). The gene families unique to *A. angustus* are enriched in various biosynthetic categories (for example, terpenoid and zeatin) and various activity categories (for example, nutrient reservoir activity and catechol oxidase activity) (Supplementary Tables 15 and 16).

Phylogenetic inferences from 85 single-copy nuclear genes sampled for *A. angustus* and 18 other green plants resolve hornworts (*A. angustus*), mosses (*P. patens*) and liverworts (*M. polymorpha*) as a monophyletic group, with hornworts sister to mosses and liverworts, which agrees with inferences from 852 nuclear genes sampled from 103 plant species^1^ (Fig. 1b, Supplementary Figs. 8 and 9, Supplementary Table 17 and Supplementary Notes 2.1 and 2.2). The divergence (Supplementary Figs. 10 and 11, Supplementary Tables 18–20 and Supplementary Note S2.3) of the extant crown group of hornworts is estimated at 275.62 million years ago (Ma) (95% highest posterior density, 179.3–384.6 Ma) (middle Carboniferous–early Jurassic) (Supplementary Fig. 11 and Supplementary Table 20), which is comparable to the crown age of hornworts estimated based on two organellar sequences from 77 hornworts and 11 other land plants^10^. These estimates are thus older than those inferred from the fossil record, considering that the oldest putative hornwort fossil is a spore from the Lower Cretaceous Baqueró Formation, Argentina (from 145 to 100 Ma) that resembles the spores of extant *Anthoceros*^18^.

Comparative genomics shows that the genome of *A. angustus* has lost many gene families (that is, 2,145) and comparatively only modest gains (that is, 497) (Fig. 1b). A similar trend characterizes the genome of *Marchantia* and of the ancestor common to all bryophytes, whereas *P. patens* has gained more families (that is, 1,334) than it has lost (that is, 1,248; Fig. 1b). Thus, bryophyte genomes may not only harbour a number of genes and gene families comparable to those of vascular plants and in particular seed plants (Fig. 1b) but may also be highly dynamic through evolutionary time.

Many, if not most, land plants harbour genomic signatures of ancient whole-genome duplication (WGD)^19^. However, like that of *Marchantia*^15^, the genome of *Anthoceros* lacks evidence of having undergone a WGD (Fig. 1c, Supplementary Fig. 13 and Supplementary Note 3.1), which confirms the hypothesis drawn previously from the analysis of transcriptomic data^20^. The chromosomal arrangement of genes is not much conserved among the three bryophyte lineages (Supplementary Fig. 14a,b and Supplementary Note 3.2), which likely reflects the ancient divergence of these different lineages of bryophytes. For example, the longest co-linear block corresponds to a mere five anchor pairs for both *A. angustus* versus *P. patens* and *A. angustus* versus *M. polymorpha*, whereas within the *A. angustus* genome, the largest co-linear segment consists of six anchor pairs (Supplementary Fig. 14).

The *A. angustus* genome contains a much lower percentage of multi-copy gene families than that of single-copy gene families, implying low genetic redundancy (Supplementary Table 17), which is similar to what has been observed for the liverwort *Marchantia*^15^.

## Transcription factors

The *A. angustus* genome comprises 333 putative transcription factor (TF) genes covering 61 families, a number that is highly similar to that of the other two bryophyte genomes (Supplementary Fig. 15, Supplementary Table 21 and Supplementary Note 4.1). The diversity of TF genes in extant plants is rather stable (Supplementary Fig. 15) and resulted from two ancient bursts of TF families during the diversification of green plants: one concomitant with the origin of streptophytes and the other with the transition to land^15,21^. In plants, genes encoding TFs are among the most highly retained following polyploidy^22^, a pattern reflected in the comparison of the three bryophyte genomes^14,15^. *A. angustus* and *M. polymorpha*, whose genome did not undergo WGDs hold a small number of TF compared to *P. patens*, which experienced at least one WGD in its ancestry, resulting in a substantially larger number of TF genes (Supplementary Fig. 15). It supports the hypothesis that the WGD is an important mechanism for expansion of TF families^23^.

**Fig. 2:**
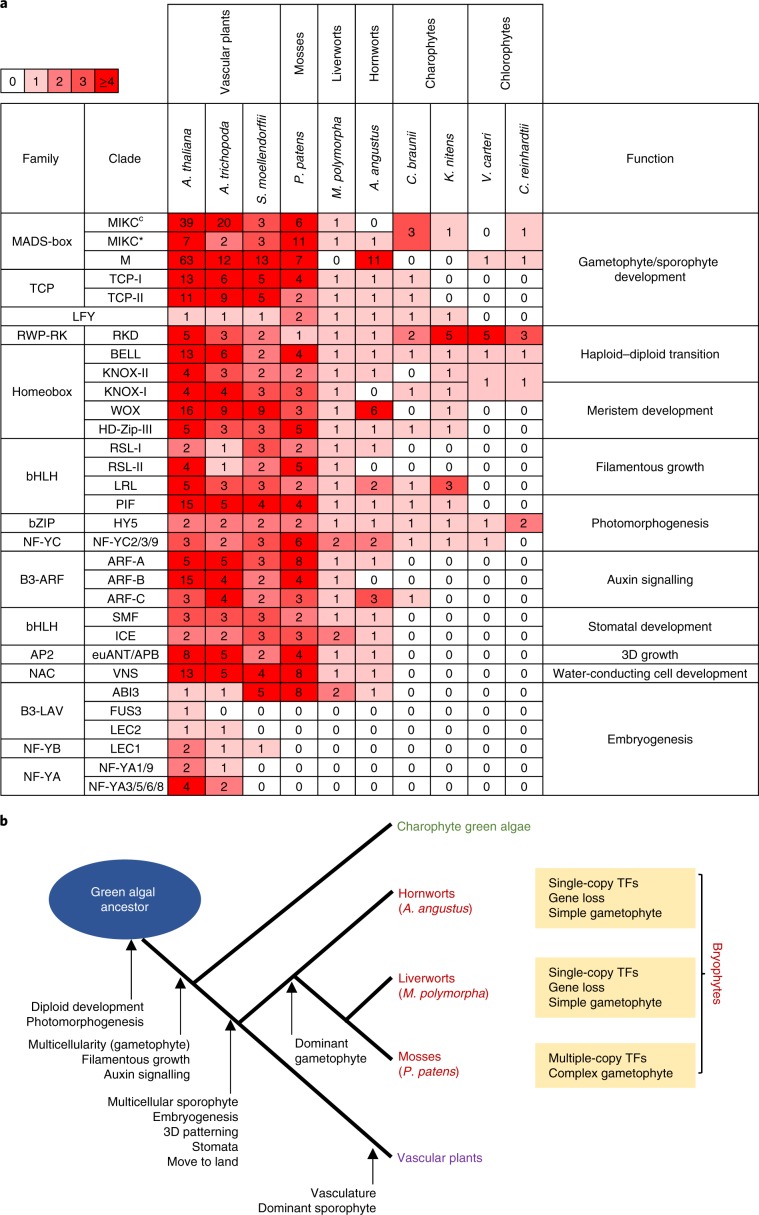
Major TFs for plant body plan and evolutionary innovations within plants. **a**, Overview for the number of major TFs for plant body plan in ten green plants. Colour key on the upper left of the heatmap denotes the TF numbers. **b**, Major innovations in plants and evolutionary features of three bryophyte lineages.

Phylogenetic analyses of 24 gene families contributing to the development of plant body plans or adaptation to the terrestrial environment, including 16 TF gene families^24,25^ (Fig. 2a, Supplementary Figs. 16–54, Supplementary Table 22 and Supplementary Note 4.2), confirm that a considerable number of genes, such as genes involved in gametophyte or sporophyte development, haploid–diploid transition, meristem development, filamentous growth, photomorphogenesis and auxin signalling (Fig. 2), composed the genetic toolkit of plants before the conquest of land^26^. In particular, the TF genes for filamentous growth and auxin signalling arose in charophyte green algae^27,28^ (Fig. 2b), which are thought to be the closest living relatives to extant land plants, implying the preliminary establishment of relatively more complex body plan in these basal streptophytes for plant terrestrial adaptation^29^. Furthermore, a set of genes underlying key morphological innovations for terrestrial adaptation probably evolved along with the colonization of land^30,31^ (Fig. 2b), such as *SMF* and *ICE* for stomatal development (Supplementary Figs. 29 and 30), *APB*, *CLE* and *CLV1* for 3D growth (Supplementary Figs. 36 and 50–52), and *VNS* for water-conducting-cell development (Supplementary Fig. 38). The sporophyte morphology of bryophytes is relatively simple, and many of the genes involved in the elaborate regulation of embryogenesis^32^, such as *FUS3*, *LEC1*, *LEC2*, *NF-YA1/9* and *NF-YA3/5/6/8* are absent in *A. angustus*, *Marchantia* and *Physcomitrella* (Fig. 2a and Supplementary Figs. 39–41). The *ABI3* genes that mainly function in embryo maturation and seed desiccation tolerance in flowering plants are present in bryophytes, and have roles in desiccation tolerance in their vegetative tissues^33^.

In *A. angustus*, most genes involved in the development of plant body plans have a single copy, and a few *A. angustus* TF gene families even lost a subset of duplicates (Fig. 2a and Supplementary Figs. 16–52). For example, in the bHLH family, the class I *RSL* gene that controls the development of rhizoids and root hairs, thought to have been important for the colonization of land^34^, is present in the *A. angustus* genome, whereas the class II *RSL* genes responsible for regulating protonema differentiation in *P. patens* or root hair elongation in *A. thaliana* by auxin^35^ are absent (Supplementary Fig. 27 and Supplementary Note 4.2). The lack of class II *RSL* genes in *A. angustus* might be related to the morphological simplification of this species with respect to tip-growing filamentous structures^2^. For the *KNOX* genes from the homeobox gene family, the *A. angustus* genome retains one class II *KNOX* gene for haploid-to-diploid morphological transition^36^, but lacks class I *KNOX* genes (Supplementary Fig. 23), whose activity is necessary for seta extension in the sporophytes in *P. patens*^37^. The absence of this gene might be linked to the absence of setae in hornworts^2^. The genome of *A. angustus* also holds few type II MIKC^C^ *MADS-box*, class B *ARF*, *NCARF* and short *PIN* genes, as a result of gene losses suggested by our phylogenetic analysis (Supplementary Figs. 17, 42, 45 and Supplementary Note 4.2). The class II *RSL*, class B *ARF*, *NCARF* and short *PIN* genes all have auxin-related functions (Supplementary Note 4.2). Since these auxin-related genes were consistently lost in *A. angustus*, this hornwort species possesses the simplest auxin molecular toolkit among all investigated land plants so far^38^. Thus, like the liverwort *M. polymorpha*^15^, *A. angustus* exhibits low redundancy for genes shaping the plant body plan (Fig. 2b). Such a limited toolkit may be characteristic of the ancestor to bryophytes and hence, perhaps, of the earliest land plants with a dominant thalloid gametophyte, and provide the foundation to explaining the architectural simplicity of these plants. By contrast, the genome of *P. patens*, which develops a leafy stem, has the most TF genes involved in the development of plant body plans among the compared bryophytes (Fig. 2b). Although the genome of *A. angustus* seems poor in genes composing the network underlying the development of its body plan, the TF gene families linked to responses to terrestrial environmental stimuli exhibit lineage-specific gene expansions in *A. angustus*, namely, the *LISCL* genes for mycorrhizal signalling in the *GRAS* gene family^39^ (Supplementary Fig. 53) and the clade *SIP1* for ABA signalling under water stress in the *Trihelix* gene family^40^ (Supplementary Fig. 54).

## Gene-family expansion

**Fig. 3:**
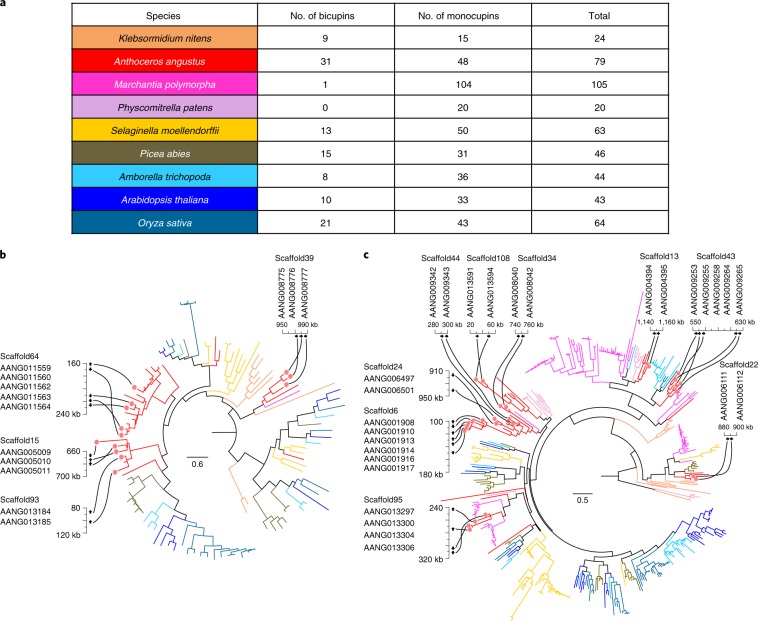
Expansion of *cupin* gene family in *A. angustus*. **a**, A summary of the number of *cupin* genes from nine species based on a Pfam search of cupin_1 domain (PF00190). **b**,**c**, Phylogenetic trees show *cupin* genes in nine plant genomes: *bicupins* (**b**) and *monocupins* (**c**). The colour of each branch corresponds to the background colour for each species in **a**. The tandem duplicated gene clusters are ordered and shown on scaffolds of the *A. angustus* genome. The scale bars in the trees show the number of amino acid substitutions per site.

Besides two TF gene families, the *A. angustus* genome harbours a variety of other uniquely expanded gene families (Supplementary Fig. 55). The genome comprises an very large number of pentatricopeptide repeat (*PPR*) genes for plant organellar RNA processing^41^, accounting for approximately 7.90% of the predicted protein-coding genes. The expanded *PPR* genes are PLS-class *PPR* genes (Supplementary Fig. 55, Supplementary Tables 23 and 24 and Supplementary Note 5.1). Most of the PLS-class PPR proteins in *A. angustus* were predicted to be localized in the mitochondrion or chloroplast (Supplementary Table 24). The expansion of the PLS-class *PPR* genes correlates with the large number of RNA editing sites estimated in the organellar genomes of *A. angustus* (Supplementary Table 23). Our findings add further support to the hypothesis that an increase in the number of both RNA editing sites and *PPR* genes (especially the PLS-class PPR) occurred after the separation of land plants from green algae^41,42^ (Supplementary Table 23). The reduced number of *PPR* genes and absence of RNA editing in marchantiid liverworts are most probably secondary losses (Supplementary Table 23), as the organellar RNA editing and plant-specific extensions of *PPR* genes were also found in jungermanniid liverworts^43^. Through RNA editing, the PPR proteins could act as ‘repair’ factors that alleviate DNA damage caused by increased UV exposure in terrestrial environments^41^. Other stress-response gene families have also expanded in *A. angustus*, such as cupin and cytochrome P450 (CYP) (Supplementary Fig. 55). Two groups of cupin (PF00190) proteins—that is, monocupins and bicupins—can be recognized on the basis of the number of cupin domains^44^. In *A. angustus*, the *cupin* gene family has undergone a significant expansion (Supplementary Table 25) such that it comprises more *bicupin* genes than any other plant (Fig. 3a, Supplementary Figs. 56 and 57, Supplementary Table 25 and Supplementary Note 5.2). Expansion of the *cupin* gene family in *A. angustus* resulted mainly from tandem gene duplications (Fig. 3b,c and Supplementary Note 5.2). Since bicupins (that is, 11S and 7S seed storage proteins) are desiccation-tolerant proteins in higher land plants^44^, the large number of *bicupin* genes in *A. angustus* could indicate adaptation for coping with drought stress in the terrestrial environment. The large number of *A. angustus*-specific *monocupin* genes are homologous to the *P. patens PpGLP6* gene (XP_001782709.1) (Supplementary Fig. 57 and Supplementary Note 5.2), which encodes a protein with manganese-containing extracellular superoxide dismutase (SOD) activity to respond to oxidative stress in terrestrial environments^45^. The *CYP* genes for primary and secondary metabolism have also expanded in *A. angustus* (Supplementary Fig. 55 and Supplementary Note 5.3). For instance, genes belonging to the subfamilies *CYP71* and *CYP85* contain 56 and 46 genes, respectively (Supplementary Figs. 58–61 and Supplementary Tables 26 and 27). The *A. angustus CYP* genes were assigned to 28 KEGG pathways, of which ‘flavonoid 3′-monooxygenase/flavonoid 3′,5′-hydroxylase’ and ‘abscisic acid 8′-hydroxylase’ were the most representative (Supplementary Table 28). Within the *CYP71* gene subfamily, genes homologous to flavonoid 3′-hydroxylase (monooxygenase) (*F3'H*) or flavonoid 3′,5′-hydroxylase (*F3*′*5*′*H*) genes that are involved in flavonoid biosynthesis^46^ are highly expanded in *A. angustus* (Supplementary Fig. 59 and Supplementary Note 5.3). Because flavonoids have an important role in UV-B protection^46^, the expansion of flavonoid biosynthesis related genes in *A. angustus* might again represent a molecular adaptation to life in the terrestrial environment. Among the *CYP85* genes, the genes homologous to abscisic acid 8′-hydroxylase genes involved in abscisic acid catabolism during drought stress response^47^ are also uniquely abundant in *A. angustus* (Supplementary Fig. 60 and Supplementary Note 5.3), and may account for the high desiccation tolerance of *A. angustus*. Like the *cupin* gene family, many of the above expanded gene families occur in tandem arrays (Supplementary Table 29). At least 9.82% of protein-coding genes in *A. angustus* form ‘tandem’ clusters in the genome (Supplementary Table 30 and Supplementary Note 5.4), compared with only 1% in *P. patens*^14^ and 5.9% in *M. polymorpha*^15^.

## CO_2_-concentrating mechanism

Hornworts are the only extant land plant lineage harbouring a pyrenoid-based CO_2_-concentrating mechanism (CCM) similar to that of green algae^9,48^ (Supplementary Note 6.1), for which the key components have been identified^49^. To clarify whether the CCM components of green algae have orthologues in hornworts and other land plants, we searched the *A. angustus* genome and other plant genomes or transcriptomes with reference to the CCM genes from chlorophyte green algae *Chlamydomonas reinhardtii*^49,50^ (Supplementary Figs. 62–71 and Supplementary Note 6.2). *A. angustus* and all other green plants harbour orthologues of *CAH1/2* whose expression is modulated by external inorganic carbon concentration; of *CemA,* which maintains stromal pH balance; of *LCI11,* which mediates the entry of HCO^3−^ in the thylakoid lumen; and of *RCA1* and *RBCS1/2*, which regulate CO_2_ fixation by Rubisco (Supplementary Figs. 62, 65 and 69–72). By contrast, orthologues of *CCP1/2*, which mediate the entry of HCO^3-^ into the chloroplast stroma and of *EPYC1*, which regulate CO_2_ fixation by Rubisco were only present in chlorophyte green algae (Supplementary Figs. 67 and 72 and Supplementary Note 6.2). The three inorganic carbon transporters (*HLA3*, *LCI1* and *LCIA*-like genes) only occur in bryophytes and green algae, whereas the *A. angustus* genome lacks the related orthologues (Supplementary Figs. 63, 66 and 72 and Supplementary Note 6.2). Unexpectedly, the three kinds of carbonic anhydrases (CAH3, CAH9 and LCIB/C), which are essential components of CCM, are conserved in non-angiosperm land plants and green algae (Supplementary Figs. 62, 64, 68 and 72). The *A. angustus* genome retains the orthologues of both *LCIB/C* and *CAH3* genes, but has no copy of *CAH9* (Supplementary Fig. 72). Besides green algae, the essential CCM components occur in both hornworts and other non-angiosperm land plants that lack pyrenoids (Supplementary Fig. 72). It implies that the CCM could be an ancestral mechanism of CO_2_ fixation by plants, and pyrenoids for CCM are homologous between hornworts and green algae, whereas both CCM components and pyrenoids have undergone multiple losses in land plants in response to atmospheric changes in terrestrial environments^10,48^.

## Horizontal gene transfer

**Fig. 4:**
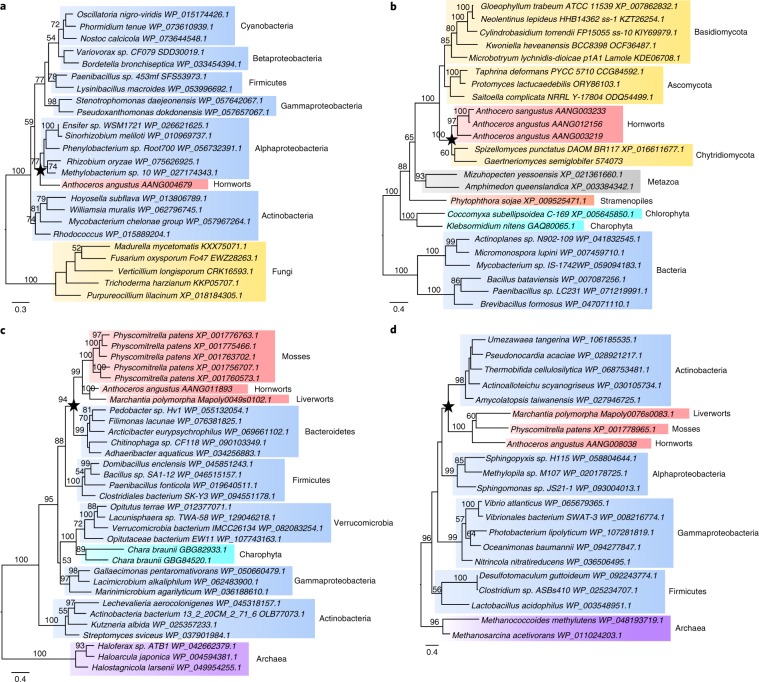
Phylogenetic affinities of genes horizontally transferred to *A. angustus*. **a**, Phylogenetic tree of glyoxalase (PF13468). **b**, Phylogenetic tree of NAD-binding dehydrogenase (PF08635). **c**, Phylogenetic tree of glucuronyl hydrolase (PF07470). **d**, Phylogenetic tree of DNA methyltransferase (PF02870 and PF01035). The stars indicate that the *Anthoceros* sequence or bryophyte sequences formed a monophyletic clade with homologues of putative HGT donor, reflecting *Anthoceros*-specific or bryophyte-specific HGT events. Maximum-likelihood bootstrap support values ≥50% are shown above the branches. Red, hornworts and other bryophytes; cyan, green algae; grey, metazoan; orange, stramenopiles; blue, bacteria; yellow, fungi; purple, archaea. The homologues from the kingdom other than the one that HGT donors are involved in are used as the outgroup. The scale bars in the trees show the number of amino acid substitutions per site.

Horizontal gene transfer (HGT) from bacteria or fungi has been reported for both the moss *P. patens*^51^ and the liverwort *M. polymorpha*^15^. Consistent with those observations, the taxonomic distribution of BLASTP hits following careful phylogenetic analysis and manual inspection suggested that 19 genes from 14 families originated from HGTs from either bacteria or fungi (Supplementary Fig. 6 and Supplementary Note 7.1). Bacterial donors are distributed among nine families: Actinobacteria (three gene families), Alphaproteobacteria (two gene families), Bacteroidetes (two gene families), Firmicutes (one gene family) and Verrucomicrobia (one gene family). Five families were acquired from fungi, belonging to Ascomycota, Basidiomycota, hornwort-symbiotic Chytridiomycota or Mucoromycota^13^ (Fig. 4a,b, Supplementary Figs. 73–84 and 86 and Supplementary Table 31). The detection of specific HGT in all three fully sequenced bryophytes is remarkable, and is probably related to the fact that these organisms form symbioses with diverse bacteria and fungi, which, together with the weakly protected tissues in the early developmental stages in the life cycle of these plants, provide the possibility for HGT^51^. In addition, we found that two families originating from HGT from bacteria are shared by the three bryophyte lineages, and one originating from a HGT from fungi is shared between hornworts and liverworts only (Fig. 4c,d, Supplementary Figs. 85 and 86, Supplementary Table 31 and Supplementary Note 7.2). The HGT genes mentioned above (SCUO value 0.2127) exhibit a significantly more biased codon-usage pattern than non-HGT genes (SCUO value 0.1595) (Supplementary Fig. 87a), which may be linked to their higher GC content (57.58%) than non-HGT genes (53.26%) (Supplementary Fig. 87b).

The HGT-derived genes in *A. angustus* mainly contribute to metabolic processes, oxidation–reduction and stress response (Supplementary Table 31). Some transferred genes related to carbohydrate metabolism are predicted to encode glucuronyl (AANG011893) and glycosyl hydrolases (AANG004297) (Fig. 4c, Supplementary Fig. 79 and Supplementary Table 31), which function in cell wall synthesis and modification and might extend the metabolic flexibility of *A. angustus* in changing environments^52^. The Alphaproteobacteria-derived gene AANG004679 encodes glyoxalase, which is related to drought stress tolerance^53^ (Fig. 4a). The Actinobacteria-derived DNA methyltransferase genes that are present only in the three groups of bryophytes are related to DNA repair^54^ (Fig. 4d). The hornworts and liverworts share the fungi-derived terpene synthase-like (*MTPSL*) genes (Supplementary Fig. 85). Terpene synthases are pivotal enzymes for the biosynthesis of terpenoids, which serve as chemical defences against herbivores and pathogens^55^. Some horizontally transferred genes in *A. angustus*, such as NAD-binding dehydrogenase (Fig. 4b) and *MTPSL* genes (Supplementary Fig. 85), underwent subsequent gene duplications. The results suggest that the acquisition of foreign genes might have provided additional means for environmental adaptation during evolution of the hornwort lineage.

## Conclusions

As land pioneers, the three bryophyte groups form a well-supported monophyletic lineage, with hornworts sister to liverworts and mosses. The genome of hornwort *A. angustus* shows no evidence of WGDs and low genetic redundancy for networks underlying plant body plan, which may be congruent with an overall simple body plan. Hornworts have retained the essential components of CCM found in green algae in response to the atmospheric changes in terrestrial environments. Meanwhile, the gene inventory in *A. angustus* expanded mainly through tandem duplication and HGT. In particular, the expansion of specific gene families and the acquisition of foreign genes have provided additional metabolic abilities in hornworts that probably facilitated their survival in a terrestrial environment. Together, our results indicate how the draft genome of *A. angustus* provides a useful model for studying early land plant evolution and the mechanism of plant terrestrial adaptation.

## Methods

### Sample preparation and sequencing

The natural populations of *A. angustus* Steph. were collected from Jinping County, Yunnan Province, China. The voucher specimen has been deposited at the herbarium, Institute of Botany, Chinese Academy of Sciences, Beijing, China with collection number W1879-2010-01-18. The sporophytes of *A. angustus* were detached from the gametophytes, sterilized in 10% sodium hypochlorite and subsequently rinsed with distilled water^56^. The sporangium was opened and the spores were homogenized and spread onto the 1/2 KnopII agar medium^57^ in Petri dishes (Supplementary Fig. 1b). The culture temperature was between 21 °C and 25 °C. Spores germinated within a couple of days, and then the sporelings started to grow. After approximately three to four weeks, the gametophyte started to grow (Supplementary Fig. 1c,d). Since spores are aposymbiotic, we did not find the phenomenon of mucilage-filled cavities colonization by cyanobacteria on the *A. angustus* gametophyte during the sterile culture. A gametophyte from a single spore was selected and cultured by asexual propagation. The tissue yielded from subculture was used for genome and RNA sequencing. We tried to induce sexual reproduction by dropping the growth temperature of gametophyte cultures to 10 °C and 16 °C, respectively; however until now they have not yet produced reproductive organs. Therefore, the sequenced *A. angustus* is indeed a single-sex individual, which is sequenced at the gametophyte phase of its life cycle.

Genomic DNA was isolated using the Plant DNAzol reagent for genomic DNA extraction (Life Technologies) according to the manufacturer’s protocols. For whole-genome shotgun sequencing, ten sequencing libraries with insert sizes ranging from 170 bp to 40 kb were generated (Supplementary Table 1). Sequencing libraries were constructed using a library construction kit (Illumina). All libraries were sequenced on the Illumina HiSeq 2000 platform. Raw sequencing reads were trimmed with Trimmomatic (v.0.33)^58^. Only high-quality reads with a total length of 126,532,381,412 bp were used for further analysis (Supplementary Table 1). For Oxford Nanopore sequencing, we constructed a genomic DNA library using the ONT 1D ligation sequencing kit (SQK-LSK108) according to the manufacturer’s instructions. The sequencing used a single 1D flow cell on a PromethION sequencer (Oxford Nanopore Technologies). A total of 63,614,292,295 bp raw reads were generated, of which 36,070,452,175 bp were retained for further analysis after filtering and trimming (Supplementary Table 3).

Total RNA was extracted using the PureLink Plant RNA reagent (Life Technologies) and further purified using TRIzol reagent (Invitrogen). For transcriptome sequencing (RNA sequencing), libraries with insert sizes ranging from 200 bp to 500 bp were constructed using the mRNA-Seq Prep Kit (Illumina) and then sequenced using the Illumina HiSeq 2000 platform. For small-RNA sequencing, the library was generated from RNA sample using the Truseq Small RNA Preparation kit (Illumina) and sequenced on the Illumina HiSeq 2500 platform.

### Decontamination

The GC content versus *k*-mer frequency distribution pattern of the Illumina raw reads (Supplementary Table 1) after trimming presented two large groups: one group with a low *k*-mer frequency (<50) and a wide GC content distribution range (median number at 0.7), and the other group with a high *k*-mer frequency (60–165) and a concentrated GC content distribution range (median number at 0.5) (Supplementary Fig. 2a). The BLASTN results against the NCBI nucleotide database revealed that the former sequences were mainly from a variety of bacteria and the latter were the real genome sequences of *A. angustus*. We also investigated the *k*-mer distributions of the raw reads from the other two published hornwort genomic sequences, *A. agrestis* (accession: ERX714368)^59^ and *Anthoceros punctatus* (accession: SRX538621)^60^, and found a similar distribution pattern as that of *A. angustus*, containing two groups, one for the contaminant sequences and the other for sequences of the plant itself (Supplementary Fig. 2c,d). Because external bacterial contaminations from the laboratory cause *A. angustus* to turn yellow and die during culturing, and all three *Anthoceros* species through axenic cultures still have the same bacterial contamination problems (Supplementary Fig. 2a,c,d), we infer that these bacterial contaminations are from symbiotic bacteria of *Anthoceros* that might accompany spores hiding in the sterilized sporangium. Furthermore, we performed the DAPI staining analysis^61^ to investigate the distribution of symbiotic bacteria in *A. angustus*. The gametophytes were stained by 0.2 mg l^−1^ DAPI (4′,6-diamidino-2-phenylindole dihydrochloride; Sigma, cat. no. D9564) for five minutes. The stained gametophytes were washed three times, and then observed using confocal microscopy. The bacterial micro-colonies were observed on the outer surface, as well as in the intercellular space of the gametophytes of *A. angustus* (Supplementary Fig. 3). Based on the GC content versus *k*-mer frequency distribution pattern of the Illumina raw reads and the result of the DAPI staining, we could imagine that there is a certain amount of bacterial sequences remaining in the genome sequencing data of *A. angustus*. In order to isolate them, we performed a series of decontamination steps. After generating the *k*-mer frequency, we chose the high-abundance *k*-mer depth (60–165) and retained the corresponding reads for further analysis. This treatment yielded filtered reads with a total length of 17,099,027,576 bp (Supplementary Table 2). The distribution pattern of GC content versus *k*-mer frequency of the *A. angustus* filtered reads is depicted in Supplementary Fig. 2b, which shows an entire group with a sequencing depth of approximately 150×. Furthermore, we performed error correction for filtered Nanopore reads using decontaminated Illumina reads by Nextdenovo (v.2.0)^62^, resulting in 9,247,957,448 bp corrected reads (Supplementary Table 3). Through MEGABLAST against the NCBI nucleotide database, we further removed 5,463,972,682 bp prokaryotic sequences or organellar sequences, and finally got 3,783,984,766 clean reads with a sequencing depth of approximately 35× (Supplementary Table 3). A total of approximately 185× coverage was obtained finally.

### Genome size estimation

To estimate the genome size of *A. angustus*, we used clean Illumina reads to calculate the *k*-mer distribution. According to the Lander–Waterman theory^63^, the genome size can be determined by dividing the total number of *k*-mers by the peak value of the *k*-mer distribution. Because we sequenced the haploid gametophyte of *A. angustus*, only one peak was found in the *k*-mer distribution. The total number of *k*-mers was 14,092,039,150, and the position of the peak was at 132 (Supplementary Fig. 4). The peak was used as the expected *k*-mer depth and substituted into the formula genome size = total *k*-mer/expected *k*-mer depth, and the haploid genome size was estimated to be 106,757,872 bp (Supplementary Fig. 4).

### Genome assembly and assessment

The clean Nanopore reads after filtering and decontamination were assembled with wtdbg-1.2.8. After finishing the pre-assembly (148 Mb), iterative polishing was conducted using Pilon (v.1.22)^64^ in which clean Illumina reads were aligned with the pre-assembled contigs. The pre-assembled contig sequences were performed with the MEGABLAST search against the NCBI nucleotide database to further remove prokaryotic sequences or organellar DNA. A total of approximately 29 Mb of data were removed. Further, we combined the final pre-assembled contig sequences from Nanopore sequencing and clean paired-read data from Illumina sequencing into scaffolds using SSPACE (v.3.0)^65^ tool (Supplementary Table 4). Genome assembly completeness was assessed using the plantae database of 956 single-copy orthologues using BUSCO (v.3)^16^ with a BLAST threshold *E*-value of 1 × 10^−5^ (Supplementary Table 10).

### Transcriptome assembly and mapping

We used Trimmomatic^58^ to remove adaptors from the raw reads of transcriptome sequences and filter out low-quality reads before assembly. The resulting high-quality reads were de novo assembled and annotated using Trinity (v.2.5.1)^66^. For genes with more than one transcript, the longest transcript was chosen as the unigene and used to predict open reading frames (ORFs) using TransDecoder (v.5.0.2) (https://github.com/TransDecoder/TransDecoder/wiki). Finally, we obtained 39,044 unigenes, 26,805 of which had predicted ORFs. To extend the validation of genome assembly, the transcriptome was compared to the reference assembly using BLASTN, with an *E*-value <1 × 10^−5^. Of the 26,805 transcripts (>200 bp), 97.66% were successfully mapped back to the final assembled genome (Supplementary Table 5).

### Repeat prediction

Tandem Repeats Finder (v.4.09)^67^ was used to search for tandem repeats in the *A. angustus* genome. Both homology-based and de novo approaches were used to search for TEs. In the homology-based approach, we used RepeatMasker (v.4.1.0)^67^ and RepeatProteinMask^68^ with the Repbase^69^ database of known repeat sequences to search for the TEs in the *A. angustus* genome. In the de novo approach, we used LTR_FINDER (v.1.0.2)^70^, PILER (v.1.3.4.)^71^ and RepeatModeler (v.1.0.3)^72^ to construct a de novo repeat sequence database for *A. angustus* and then used RepeatMasker to search for repeats in the genome. All the repeats identified by different methods were combined into the final repeat annotation after removing the redundant repeats. The predicted repeats covered 64.21% of the genome sequence (Supplementary Table 6). The categories of predicted TEs in the *A. angustus* genome are summarized in Supplementary Table 7.

### Genome annotation

To predict protein-coding genes, three approaches were used: (1) de novo gene prediction, (2) homology-based prediction, and (3) RNA-sequencing annotation. For de novo prediction, AUGUSTUS (v.2.5.5)^73^ and GlimmerHMM (v3.0.1)^74^ were applied to predict genes. For homology-based prediction, we mapped the protein sequences of five published green plant genomes (*Arabidopsis thaliana*, *Selaginella moellendorffii*, *P. patens*, *M. polymorpha* and *Klebsormidium nitens*) onto the *A. angustus* genome using TBLASTN, with a threshold *E*-value of 1 × 10^−5^, and then used GeneWise (v.2.4.1)^75^ to predict gene structures. The de novo set and five homologue-based results were combined by MAKER (v.1.0)^76^ to integrate a consensus gene set (Supplementary Table 8). To supplement and improve the gene set, we aligned the RNA-sequencing data to the genome using TopHat (v2.1.1)^77^, and the alignments were used as input for Cufflinks (v.2.2.1)^78^ with default parameters. We manually combined the MAKER gene set and ORFs of transcripts to form the final gene set that contains 14,629 genes (Supplementary Table 8).

The *A. angustus* predicted genes were aligned against the sequences in NCBI non-redundant protein database using BLASTP^79^ (*E*-value <1 × 10^−5^). According to the NCBI taxonomy categories of best BLAST hits, the source of *A. angustus* genes were classified (Supplementary Fig. 6). Functional annotation of these predicted genes was obtained by aligning the protein sequences of these genes against the sequences in public protein databases using BLASTP^79^ (*E*-value <1 × 10^−5^, identity >30% and coverage >70%, excluding annotations only characterized as hypothetical or predicted protein), including, SwissProt^80^, TrEMBL^80^, Pfam^81^, GO^82^ and KEGG^83^ (Supplementary Tables 9 and 32).

### Identification of non-coding RNA genes

To obtain a reliable profile of *A. angustus* miRNAs, we used mapped reads from small-RNA sequencing with reference to the *A. angustus* draft genome to search against miRNA sequences in *A. thaliana*, *Oryza sativa*, *S. moellendorffii*, *P. patens* and *C. reinhardtii* from miRBase (http://www.mirbase.org/) for predicting the known miRNAs. The mapped reads were also used to identify novel miRNAs using miREvo (v.1.2)^84^ software. The tRNA genes were searched by tRNAscan-SE (v.1.3.1)^85^. The rRNA genes were predicted by aligning plant rRNA sequences from NCBI (*A. thaliana* and *Anthoceros agrestis*) to the *A. angustus* genome by BLASTN. The snRNA genes were predicted using INFERNAL (v.1.1)^86^ to search from the Rfam database.

### Gene-family identification

To construct the dataset for gene-family clustering, the protein-coding genes from the genomes of *A. angustus* and 18 other green plants were used, including those of seven angiosperms (*A. thaliana*, *Genlisea aurea*, *Vitis vinifera*, *O. sativa*, *Phalaenopsis equestris*, *Zostera marina* and *Amborella trichopoda*), one gymnosperm (*Picea abies*), one lycophyte (*S. moellendorffii*), two bryophytes (moss *P. patens* and liverwort *M. polymorpha*), two charophytes (*Chara braunii* and *K. nitens*) and five chlorophytes (*Volvox carteri*, *Chlamydomonas reinhardtii*, *Ulva mutabilis*, *Coccomyxa subellipsoidea* and *Chlorella variabilis*) (Supplementary Table 13). We chose the longest transcript to represent each gene and removed mitochondrial and chloroplast genes. After performing an all-against-all BLASTP search with a threshold *E*-value of 1 × 10^−5^, identity >30% and coverage >30%, orthogroups or putative gene families or subfamilies were identified using OrthoMCL (v.2.0)^87^, on the basis of a collection of 397,132 predicted protein-coding genes from the above 19 Viridiplantae genomes. A 5-way comparison of *A. angustus*, Setaphyta (*M. polymorpha* and *P. patens*), Tracheophyta (vascular plants) (*A. thaliana*, *V. vinifera*, *O. sativa*, *Z. marina*, *P. equestris*, *A. trichopoda*, *P. abies*, *G. aurea* and *S. moellendorffii*), Charophyta (*C. braunii* and *K. nitens*) and Chlorophyta (*V. carteri*, *C. reinhardtii*, *U. mutabilis*, *C. subellipsoidea* and *C. variabilis*) is shown in Fig. 1a. For *A. angustus*-specific gene families, we conducted GO and KEGG enrichment analyses via an enrichment pipeline (https://sourceforge.net/projects/enrichmentpipeline/).

### Phylogenomics

We extracted 85 single-copy gene families shared by 19 Viridiplantae for phylogenomic analysis (Supplementary Note 2.1). The amino acid alignments of each single-copy gene family were aligned by MAFFT (v.7)^88^, and the nucleotide alignments were generated separately with TranslatorX (v0.9)^89^ on the basis of the corresponding amino acid translation. The amino acid data, the complete nucleotide data and the first and second codon positions, as well as the third codon positions, were concatenated as super-matrices. These data matrices were used for maximum likelihood phylogenetic analyses by RAxML (v.7.2.3)^90^ with the GTR + Γ and JTT models for nucleotide and amino acid data, respectively. For each analysis, the bootstrap support was estimated based on 300 pseudoreplicates using a GTR + CAT approximation. To estimate the degree of substitutional saturation for the four concatenated datasets mentioned above (Supplementary Note 2.2), we plotted the uncorrected *p*-distances against the inferred distances using the method described by Forterre and Philippe^91^. The level of saturation was estimated by computing the slope of the regression line in the plot; the shallower the slope, the greater the degree of saturation. The maximum composite likelihood method was used to calculate the inferred distances for nucleotide data and Poisson correction was used to calculate the inferred distances for the amino acid data.

To improve the taxon sampling in bryophytes for divergence time estimation, the transcriptome sequences of 22 other bryophytes were downloaded from the 1KP database^92^ (http://www.onekp.com/public_data.html) and used in subsequent analyses (Supplementary Table 18 and Supplementary Note 2.3). The divergence time was estimated using the MCMCTree program in the PAML package (v.4.7)^93^ under the nucleotide general time reversible (GTR) substitution model and with the independent rate model as the molecular clock model. The Markov chain Monte Carlo (MCMC) process consists of 500,000 burn-in iterations and 1,500,000 sampling iterations (1 sample per 150 iterations). The same parameters were executed twice to obtain a stable result. We applied nine node constraints in the age estimate (Supplementary Fig. 10). The minimum and maximum constraints for each node are shown in Supplementary Table 19.

Gene-family sizes were inferred from the gene-family profile obtained by the program OrthoMCL. The minimum ancestral gene families were estimated using DOLLOP program included in the PHYLIP package (v.3.695)^94^ to determine gene-family gain or loss evolutions of gene families. There are 8,141 gene families in the *A. angustus* genome, 8,944 in *M. polymorpha* and 9,566 in *P. patens*, and 9,789 ancestral families in the ancestral bryophyte lineage (Fig. 1b).

### *K*_S_ distribution and co-linearity analysis

All *K*_S_ distributions were constructed using wgd (v.3.0)^95^ using default settings. The *M. polymorpha* and *P. patens* genome data was acquired from the PLAZA resource^96^. Pairwise co-linearity analyses within and between *A*. *angustus*, *M. polymorpha* and *P. patens* were conducted using I-ADHoRe 3.0^97^ with the following parameter settings: gap_size = 30, cluster_gap = 35, q_value = 0.75, prob_cutoff = 0.01, anchor_points = 3, alignment_method = ‘gg2’, level_2_only = ‘false’, table_type = ‘family’ and multiple_hypothesis_correction = ‘FDR’. Within-genome co-linearity analyses were based on the paralogous families inferred with wgd, whereas the between-genome co-linearity analyses were conducted using gene families inferred with OrthoFinder using default settings.

### Analysis of TFs

We used the genome-wide TF prediction program iTAK (v.1.7)^98^ (http://bioinfo.bti.cornell.edu/cgi-bin/itak/index.cgi) with default parameters to preliminarily identify TFs in the above 19 Viridiplantae (Supplementary Tables 13 and 22). The reconstruction of the ancestral state for the individual TF family was performed using Mesquite (v.3.51)^99^ (http://mesquiteproject.org/), and the most parsimonious assumption was taken.

### Phylogenetic analysis of gene families

Generally, HMMER search^100^ with a domain profile or BLAST search using known protein sequences from other plants as queries was performed to retrieve the sequences from the *A. angustus* genome (Supplementary Notes 4–6). The results of TF prediction by iTAK^98^ were used as references. Multiple sequence alignments were performed using the MAFFT^88^ program (https://mafft.cbrc.jp/alignment/software/). The maximum-likelihood phylogenetic trees were implemented with RAxML-HPC2 on XSEDE^101^ through the CIPRES Science Gateway (v.3.3) (https://www.phylo.org/), estimating branch support values by bootstrap iterations with 1,000 replicates.

### Gene-family expansion identification

To understand gene-family expansion or contraction in *A. angustus* compared with that in 18 other green plants, the mean gene-family size was calculated for all gene families (excluding orphans and species-specific families). The number of genes per species for each family was transformed into a matrix of z-scores to centre and normalize the data. The first 100 families with the largest gene-family size in *A*. *angustus* were selected (Supplementary Fig. 55). The clustering and visualization were performed using Genesis (v.3.0)^102^. The functional annotation of each family was predicted on the basis of sequence similarity to entries in the Pfam protein domain database, where more than 30% of proteins in the family share the same protein domain. Transposon-derived gene families were removed because the distribution of such families is likely to be a consequence of the gene models derived from a repeat-masked genome sequence and therefore may be artefactual^103^.

### Tandem duplication definition

Genes were defined as tandemly arrayed genes if they belonged to the same family, were located within 100 kb each other, and were separated by zero, one or fewer, five or fewer, or ten or fewer non-homologous intervening ‘spacer’ genes^104^. Therefore, the four sets of tandem gene definitions were analysed.

### HGT event identification

In this study, we used two different strategies to identify candidates for *A. angustus*-specific and bryophyte-specific HGTs. For *A. angustus*-specific HGTs, we submitted 14,629 predicted coding genes of *A. angustus* to a BLASTP search against the NCBI protein database (*E*-value cutoff of 1 × 10^−5^) (Supplementary Note 7.1). The proteins with the best BLAST hits in bacterial or fungal sequences were extracted. After sequences without support of transcript evidence were excluded, a series of parameters were used to filter the candidates (Supplementary Note 7.1). For the bryophyte-specific HGT, we extracted gene families that are common to at least two of the three members of bryophytes (moss *P. patens*, liverwort *M. polymorpha* and hornwort *A. angustus*). To preliminarily determine whether these clusters are HGT candidates, we submitted the corresponding *A. angustus* members of each cluster to the NCBI protein database for BLASTP search and checked the taxonomy report of the top 1,000 BLAST hits (Supplementary Note 7.2). The homologues of published HGTs in *P. patens*^51^ and *M. polymorpha*^15^ were also investigated in the *A. angustus* genome. All candidate HGTs were subjected to phylogenetic analysis for verification. Synonymous codon-usage order values and GC contents of HGT and non-HGT genes were calculated by CodonO^105^.

### Reporting Summary

Further information on experimental design is available in the Nature Research Reporting Summary linked to this article.

## Supplementary information

Supplementary Information Supplementary Notes 1–7, Figs. 1–87 and Tables 1–20.

Reporting Summary

Supplementary Tables Supplementary Tables 21–32.

## Data Availability

The *A. angustus* genome project has been deposited at the NCBI under the BioProject number PRJNA543716. The genome sequencing data were deposited in the Sequence Read Archive database under the accession number SRR9696346. The *A. angustus* transcriptome project has been deposited at the NCBI under BioProject PRJNA543724. The transcriptome sequencing data were deposited in the Sequence Read Archive database under the accession number SRR9662965. The assembled genome sequences, gene models and miRNA data are available via DRYAD (10.5061/dryad.msbcc2ftv). All data that support the findings of this study are also available from the corresponding authors upon request.
