# Supplementary material for: The hornwort genome and early land plant evolution
Source: Nat Plants. 2020 Feb 10;6(2):107–18. doi: 10.1038/s41477-019-0588-4 (PMC7027989; doi:10.1038/s41477-019-0588-4)
Supplement: Supplementary file 1 — Supplementary Notes 1–7, Figs. 1–87 and Tables 1–20. [file 41477_2019_588_MOESM1_ESM.pdf]

nature plants

SUPPLEMENTARY INFORMATION

https://doi.org/10.1038/s41477-019-0588-4

In the format provided by the authors and unedited.

The hornwort genome and early land plant evolution

Jian Zhang[1,18], Xin-Xing Fu[1,2,18], Rui-Qi Li[1,18], Xiang Zhao[3,18], Yang Liu[4,5,18], Ming-He Li[6,18], Arthur Zwaenepoel[7,8,18], Hong Ma[9], Bernard Goffinet[10], Yan-Long Guan[11], Jia-Yu Xue[12], Yi-Ying Liao[4,13], Qing-Feng Wang[13], Qing-Hua Wang[1], Jie-Yu Wang[6,14], Guo-Qiang Zhang[6], Zhi-Wen Wang[3], Yu Jia[1], Mei-Zhi Wang[1], Shan-Shan Dong[4], Jian-Fen Yang[4], Yuan-Nian Jiao[1], Ya-Long Guo[1], Hong-Zhi Kong[1], An-Ming Lu[1], Huan-Ming Yang[5], Shou-Zhou Zhang[4,19]*, Yves Van de Peer[7,8,15,16,19]*, Zhong-Jian Liu[6,14,17,19]* and Zhi-Duan Chen[1,13,19]*

[1]State Key Laboratory of Systematic and Evolutionary Botany, Institute of Botany, Chinese Academy of Sciences, Beijing, China. [2]University of Chinese Academy of Sciences, Beijing, China. [3]PubBio-Tech Services Corporation, Wuhan, China. [4]Key Laboratory of Southern Subtropical Plant Diversity, Fairy Lake Botanical Garden, Shenzhen & Chinese Academy of Science, Shenzhen, China. [5]BGI-Shenzhen, Shenzhen, China. [6]Key Laboratory of National Forestry and Grassland Administration for Orchid Conservation and Utilization at College of Landscape Architecture, Fujian Agriculture and Forestry University, Fuzhou, China. [7]Department of Plant Biotechnology and Bioinformatics, Ghent University, Ghent, Belgium. [8]VIB Center for Plant Systems Biology, Ghent, Belgium. [9]Department of Biology, Huck Institutes of the Life Sciences, Pennsylvania State University, University Park, PA, USA. [10]Department of Ecology and Evolutionary Biology, University of Connecticut, Storrs, CT, USA. [11]Key Laboratory for Plant Diversity and Biogeography of East Asia, Kunming Institute of Botany, Chinese Academy of Sciences, Kunming, China. [12]Center for Plant Diversity and Systematics, Institute of Botany, Jiangsu Province and Chinese Academy of Sciences, Nanjing, China. [13]Sino–Africa Joint Research Center, Chinese Academy of Sciences, Wuhan, China. [14]College of Forestry and Landscape Architecture, South China Agricultural University, Guangzhou, China. [15]Center for Microbial Ecology and Genomics, Department of Biochemistry, Genetics and Microbiology, Pretoria, South Africa. [16]College of Horticulture, Nanjing Agricultural University, Nanjing, China. [17]Fujian Colleges and Universities Engineering Research Institute of Conservation and Utilization of Natural Bioresources, College of Forestry, Fujian Agriculture and Forestry University, Fuzhou, China. [18]These authors contributed equally: Jian Zhang, Xin-Xing Fu, Rui-Qi Li, Xiang Zhao, Yang Liu, Ming-He Li, Arthur Zwaenepoel. [19]These authors jointly supervised this work: Shou-Zhou Zhang, Yves Van de Peer, Zhong-Jian Liu, Zhi-Duan Chen. *e-mail: shouzhouz@126.com; yves.vandepeer@psb.vib-ugent.be; zjliu@fafu.edu.cn; zhiduan@ibcas.ac.cn

Supplementary Information

The hornwort genome and early land plant evolution

Supplementary Notes **2**

S1. Plant materials **2**

S1.1. The introduction of bryophytes 2

S1.2. Choice of hornwort species for genome sequencing 2

S2. Phylogenomics **2**

S2.1. Phylogenomic analysis 3

S2.2. Substitutional saturation analysis 3

S2.3. Divergence time analysis 3

S3. Whole genome duplication **4**

S3.1. K_S distribution analysis 4

S3.2. Co-linearity analysis 4

S4. Transcription factors **5**

S4.1. Transcription factor prediction 5

S4.2. Transcription factors for plant body plan and/or environmental adaptation 5

S5. Gene family expansion **6**

S5.1. Pentatricopeptide repeat (PPR) gene family 6

S5.2. Cupin gene family 7

S5.3. Cytochrome P450s 8

S5.4. Tandem duplication 11

S6. CO_2 concentrating mechanism **11**

S6.1. pyrenoid and CO_2-concentrating mechanism in hornworts 11

S6.2. CO_2-concentrating mechanism components 12

S7. Horizontal gene transfer **12**

S7.1. Identification of *A. angustus*-specific HGTs 12

S7.2. Identification of bryophyte-specific HGTs 13

References **13**

Supplementary Figures **20**

Supplementary Tables **124**

Supplementary Tables (excel) **145**

Supplementary Notes

S1. Plant materials

S1.1. The introduction of bryophytes

Bryophytes consist of three major lineages: Bryophyta (mosses), Marchantiophyta (liverworts), and Anthocerotophyta (hornworts). Resolving their relationships is critical for our understanding of the early diversification of land plants. Bryophytes play important ecological roles in local nutrient flow and global geochemical cycles. With about 18,000 species they hold a remarkable diversity of land plants, and are distributed cross all habitats except marine ecosystems[1,2]. Bryophytes lack flowers, seeds and highly specialized vascular tissues characteristic of tracheophytes, and their dominant haploid gametophytes are either thalloid or leafy shoots[3]. Rhizoids rather than true roots help bryophytes to anchor to substrate and uptake water and minerals. Rhizoids also play a role in the interactions of bryophytes with microbes in the substrate[4], with many bryophytes able to form symbiotic associations with bacteria in thalloid/leafy shoots[5–7] and fungi in thalloid/leafy shoots or rhizoids[8–12].

S1.2. Choice of hornwort species for genome sequencing

Hornworts possess special features, such as the algal-like chloroplasts (carbon-concentrating pyrenoid)[13,14], mucilage cavities invaded by *Nostoc* in the thallus[5], and meristematic zone at the base of the capsule allowing it to grow for a long period[15]. Most hornworts establish symbiotic relationships with both bacteria and fungi[5,6,10]. To date, only the whole genome of a moss (*Physcomitrella patens*) and a liverwort (*Marchantia polymorpha*), have been published[16,17] among bryophyte lineages. The hornwort species *Anthoceros agrestis* has been suggested for use as a model system[18], although its genome was sequenced under low coverage only[19], as was that of *A. punctatus*[20]; detailed genome assembly and comprehensive analyses are still needed. A complete hornwort genome would complete the gap in sampling the phylogenetic breadth of land plant lineages for investigating the genome evolution among major extant lineages emerging from the early diversification of land plants. These comparisons help to explore how early land plants and their morphological complexity evolved and identify the evolutionary history of key innovations for the transition from water to land. We chose *Anthoceros angustus* Steph., the first described hornwort species from China[21], as a representative hornwort for genome sequencing (see **Methods**). *Anthoceros* is the largest genus of hornworts, with species occurring around the world[22]. *A. angustus* is dioecious and characterized by the presence of gemmae along the apical margin of the thallus and exhibits a high variation in spore architecture[21] (**Supplementary Fig. 1a**).

S2. Phylogenomics

S2.1. Phylogenomic analysis

Phylogenomic analysis based on 85 single-copy nuclear genes from 19 representative green plants (see **Methods** and **Supplementary Table 17**) supports two topological hypotheses: one in which the hornwort (*Anthoceros angustus*), moss (*Physcomitrella patens*) and liverwort (*Marchantia polymorpha*) form a monophyletic lineage and the hornwort is sister to a clade comprising the moss and the liverwort (bootstrap support = 100% in **Supplementary Fig. 8b** and bootstrap support = 100% in **Supplementary Fig. 8c**), and the other where the hornwort is sister to all other land plants (bootstrap support = 100% in **Supplementary Fig. 8a**). The monophyly of bryophytes is supported by inferences from amino acid sequences, as well as from in-frame nucleotide alignment considering first- and second-codon positions only (**Supplementary Fig. 8b,c**). The topology wherein the hornwort is sister to all other land plants was inferred from the in-frame nucleotide alignment using all codon positions (**Supplementary Fig. 8a**), and is thus likely drawn from signal in the third-codon positions (**Supplementary Fig. 8d**).

S2.2. Substitutional saturation analysis

We estimated the degree of substitutional saturation for the four concatenated datasets used in the phylogenetic analysis (see **Methods**). In general, the amino acid data are less saturated than the complete nucleotide data (**Supplementary Fig. 9a**,**b**). Within the nucleotide datasets, the third-codon positions appear much more saturated than do the first- and second-codon positions (**Supplementary Fig. 9c**,**d**). Since the saturated sites from third-codon positions have a negative effect on the phylogenetic inference[23], we consider the monophyly of bryophytes to be the most likely hypothesis as inferred from the amino acid and the first- and second-codon positions dataset (**Supplementary Fig. 8b,8c**), a hypothesis congruent with that proposed based 852 nuclear genes sampled for 103 plant species[24].

S2.3. Divergence time analysis

The predicted protein sequences of 22 bryophyte transcriptomes (see **Methods** and **Supplementary Table 18**) were aligned against the predicted proteins of the aforementioned 85 single-copy nuclear genes in *A. angustus* using BLASTP with *E*-value < 1e-10. The homologous sequences obtained from transcriptome data of 22 bryophyte species were then integrated into the matrix of the 85 single-copy nuclear genes from the genome data of 19 Viridiplantae species. The alignments of protein and CDS sequences followed the pipeline mentioned in Methods. The missing genes in newly added bryophyte species were treated as gaps in the alignment. The new phylogeny is congruent with the result of the 19-taxon analysis mentioned above, i.e., the in-frame nucleotide alignment with first- and second-codon positions also supported the monophyly of bryophytes and the hornworts as sister to the clade of mosses and liverworts (bootstrap support = 100% in **Supplementary Fig. 10**). Each of the bryophyte lineages (Bryophyta, Marchantiophyta, and Anthocerotophyta) is monophyletic, with 100% bootstrap support (**Supplementary Fig. 10**). Under the current sampling, the phylogeny suggests that *Anthoceros* arose from the second split within hornworts, as previously suggested[14].

This dataset was further used to estimate divergence times (see **Methods**). Fossil-based age estimates (see **Methods** and **Supplementary Table 19**) date the origin of crown embryophytes (land plants) at 503.36 Mya (95% HPD 478.6–516.6 Mya) (middle Cambrian-early Ordovician), which was followed by the divergence of bryophytes shortly after 482.1 Mya (95% HPD 457.1–502.9 Mya) (**Supplementary Fig. 11**). The diversification leading to the extant hornworts started much later, at 275.62 Mya (95% HPD 179.3–384.6 Mya) (middle Carboniferous-early Jurassic) (**Supplementary Fig. 11** and **Supplementary Table 20**), which is comparable to the crown age of hornworts estimated previously based on organellar sequences[14].

S3. Whole genome duplication

S3.1. K_S distribution analysis

The well-conserved ancient WGD signature in *Physcomitrella patens* is clearly represented in both the whole paranome and anchor pair K_S distributions (**Fig. 1c** and **Supplementary Fig. 12**). No WGD related signatures could be discerned for *Marchantia polymorpha* or *Anthoceros angustus* (**Fig. 1c** and **Supplementary Fig. 12**). Curiously, relatively recent duplicates are underrepresented in *A. angustus*, given the deviation from the expected exponential distribution under a constant duplication and loss (of fixed duplicates) model. Such deviation is not associated with any increase in anchor pairs around the apparent peak, nor with a large number of duplication events in the whole paranome K_S distribution, and is therefore not to be linked with any large-scale event such as a WGD or large segmental duplication. Provided that no significant amounts of duplicate pairs were undetected, this may either signal a decreased duplication rate or an increased loss rate in the hornwort lineage in its recent evolutionary past. Alternatively, this signature may correspond to a temporal increase in the duplication rate (or decrease in duplicate loss rate) around an age that corresponds to $K_S \approx 0.3$. Under the assumption of similar average duplication and loss rates as well as similar gene content in the hornwort lineage compared to other bryophytes (especially *Marchantia*), this alternative explanation is, however, less likely than a recent decrease/increase in duplication/loss rate. The co-linear dot plot analysis revealed no evidence of WGD events in *A. angustus* (**Supplementary Fig. 13**).

S3.2. Co-linearity analysis

We performed co-linearity analyses across the three fully sequence bryophyte genomes (*P. patens*, *M. polymorpha* and *A. angustus*) (**Supplementary Fig. 14**), and did not find evidence for a well-conserved gene order across the three bryophyte lineages, which may reflect the ancient divergence among these lineages (**Supplementary Fig. 14a**). The maximum co-linear block length in terms of number of anchor pairs between *A. angustus* and *P. patens*, was five, spanning 338.6 and 1465.6 kb on scaffold 8 and chromosome 16 of *A. angustus* and *P. patens*, respectively (**Supplementary Fig. 14b**). Likewise, the longest co-linear block between *A. angustus* and *M. polymorpha*, consisted of five anchor pairs, spanning 232.5 kb and 557.2 kb on scaffold 55 and scaffold 16 of *A. angustus* and *M. polymorpha*, respectively (**Supplementary Fig. 14b**). Lastly,

the longest co-linear segment within the *A. angustus* genome consisted of six anchor pairs, spanning 43.3 kb and 47.6 kb on scaffolds 6 and 85, respectively (**Supplementary Fig. 14c**).

S4. Transcription factors

S4.1. Transcription factor prediction

Transcription factors (TFs) are the main components of transcriptional regulation in eukaryotes and act through activating or repressing gene expression[25]. TFs are central players of the genetic toolkit for plant development and environmental response and hence greatly contribute to shaping the evolution of morphological complexity[26]. The predicted numbers of TFs in different species used in our study (see **Methods**) are summarized in **Supplementary Table 21**. We identified 333 putative TF genes of 61 TF families in *Anthoceros angustus*, a number similar to that of the other two bryophytes (**Supplementary Table 21**). In addition to the *VARL* family, specific to chlorophyte green algae, seven other TF gene families (*C2C2-YABBY*, *NOZZLE*, *SAP*, *ULT*, *FAR1*, *VOZ*, *S1Fa*-like, and *SRS*) are missing in *A. angustus* (**Supplementary Table 21**). These families have specialized functions in flowering plants, such as lateral organ, shoot, flower, and root development. The results of individual ancestral state reconstruction (see **Methods**) were integrated in a global view of TF gene family evolution for green plants (**Supplementary Fig. 15**).

S4.2. Transcription factors for plant body plan and/or environmental adaptation

To investigate the evolutionary history of gene families in *A. angustus*, thought to be involved in plant body plan, we examined 22 gene families[27–30] including 14 TF gene families (*MADS-box*, *TCP*, *LFY*, *Homeobox*, *RWP-RK*, *LAV*, *NF-YB*, *NF-YA*, *bHLH*, *bZIP*, *NF-YC*, *NAC*, *AP2*, and *ARF*) (**Fig. 2a** and **Supplementary Figs. 16–42**) and eight other related gene families (*Aux/IAA*, *PIN*, *Phytochrome*, *Crytochrome*, *NOG1*, *DEK1*, *CLE*, and *CLV1/BAM*) (**Supplementary Figs. 43–51**). The number of genes in the gene clades/families associated with plant body plan in the different green plants is summarized in **Fig. 2a** and **Supplementary Fig. 52**. To investigate the evolutionary history of gene families in *A. angustus*, thought to be related to land plant adaptation, we examined two TF gene families[31–33] (*GRAS* and *Trihelix*) (**Supplementary Figs. 53** and **54**).

The *A. angustus* genome exhibited minimal redundancy for plant body plan genes (**Fig. 2a** and **Supplementary Fig. 52**), and some duplicated gene copies have retained in other land plants but were missing in *A. angustus* (**Supplementary Figs. 17, 23, 27, 42** and **45**). For *MADS-box* genes, *A. angustus* has 11 type I genes and one type II MIKC* gene but lost type II MIKC^C genes (**Supplementary Figs. 16** and **17** and **Supplementary Table 22**). Given that type I genes are required for proper development of the female gametophyte in *Arabidopsis thaliana*[34–36], the retention of type I genes in *A. angustus* might suggest a role in the development of the ancestral gametophytic plant body form. MIKC* genes have been suggested to be conserved regulators of the gametophytic generation of land plants[37–39]. The presence of the MIKC* gene in *A. angustus* is

congruent with the hypothesis of a conserved role of MIKC* genes in gametophyte development during land plant evolution. MIKC^C genes are mainly involved in controlling sporophyte development[40–42]. The expansion of MIKC^C genes in seed plants (**Supplementary Fig. 17**) is correlated with the increased plant complexity[43], especially unique sporophytic reproductive organs, such as seeds, flowers, and fruits. The number of MIKC^C genes in non-seed plants is low, as might be expected from their simple sporophytic architecture (**Supplementary Fig. 17**). *A. angustus* lacks the MIKC^C genes (**Supplementary Fig. 17**).

For *KNOX* genes, *A. angustus* retained one class II *KNOX* gene (**Supplementary Fig. 23**), whose homologs are involved in haploid-to-diploid morphological transition in green plants[44,45]. However, it lost class I *KNOX* genes (**Supplementary Fig. 23**), which play conserved roles in promoting cell proliferation in sporophytes for both flowering plants and the moss *Physcomitrella patens* [46–48]. Nevertheless, the corresponding gene in the liverwort *Marchantia polymorpha* exhibits no localized meristematic activity and may uncover unique functions[49]. Recently, the *P. patens* class I *KNOX* gene *MKN2* has been suggested to be necessary and sufficient for seta extension from an intercalary region in the sporophytes[50]. Hornwort sporophytes lack setae and the setae in liverwort sporophytes elongate just prior to spore release[51]. Therefore, the absence of class I *KNOX* gene in the hornwort *A. angustus* and the non-existence of typical function of class I *KNOX* gene in the liverwort *M. polymorpha* might be correlated with the different shoot growth patterns within hornworts and liverworts compared to those in other land plants[52]. For *ARF* genes, our analysis confirmed the three canonical *ARF* genes, class A, B and C, and one non-canonical *ARF* gene (*NCARF*) in land plants, and the *NCARF*s are closely related to the class A *ARF*s (**Supplementary Fig. 42**). It is consistent with the previous studies[53,54]. The class A *ARF*s act primarily as transcriptional activators for the regulation of auxin-responsive genes in plant morphogenesis and *NCARF*s are positive regulators of auxin transcriptional responses through class A *ARF*s, while the classes B and C *ARF*s are mainly as transcriptional repressors[53–58]. We found that *A. angustus* only retained classes A and C *ARF* genes (**Supplementary Figs. 42** and **44**). However, the class B *ARF* and the *NCARF* genes were not found in *A. angustus* (**Supplementary Fig. 42**) or other hornwort species, such as *Nothoceros*[53] and *A. agresitis*[54]. The class B *ARF* genes have been retained in all the other land plants, and the *NCARF* genes have been retained in liverworts, mosses and lycophytes[53,54](**Supplementary Fig. 42**). Therefore, these two kinds of *ARF* genes seem to be lost in hornworts during evolution. It implies that the hornwort *A. angustus* has an auxin-mediated transcriptional system with a minimal set of components. The *A. angustus* has one long *PIN* gene and its long PIN protein localizes to the plasma membrane (**Supplementary Fig. 45**), similar to the moss *P. patens* and other land plants, implying that it might have similar functions in the regulation of auxin distribution in the gametophore and the development of sporophytes as in *P. patens*[59]. No short *PIN* genes functioning in PIN auxin efflux[60] were found in the *A. angustus* genome (**Supplementary Fig. 45**).

S5. Gene family expansion

S5.1. Pentatricopeptide repeat (PPR) gene family

The PPR proteins form one of the largest protein families in land plants[61–63]. The majority of PPR proteins localize to organelles and are involved in post-transcriptional processes within mitochondria and chloroplasts, including RNA editing, RNA splicing, and RNA cleavage and translation[61,62]. RNA editing is a post-transcriptional modification of nuclear, mitochondrial or chloroplast genome-encoded transcripts[64,65]. RNA editing is essential in plants, since many mutants resulting from specific site changes exhibit strong deleterious phenotypes[64]. The PPR family consists of two major classes, P and PLS[66,67]. PLS-class PPR proteins can be divided into PLS, E, and DYW subclasses based on their C-terminal domains[66,67].

The PFAM profile PF01535 PPR motif was used to preliminarily identify sequences containing PPR motif(s) in *Anthoceros angustus*, and PPR proteins containing single PPR motifs were filtered. Using the HMMER matrices defined by *Arabidopsis* PLS, E, and DYW motifs, we searched for these motifs in *A. angustus* PPR proteins to confirm their subclasses. We also searched independently for the DYW deaminase motif (PFAM profile PF14432) and retained the corresponding proteins with a single DYW motif. The subcellular location for each PPR protein in *A. angustus* was predicted in TargetP 1.1[68] (http://www.cbs.dtu.dk/services/TargetP/). Putative RNA editing sites for *A. angustus* organellar genomes (mitochondrial genome-NC_037476.1 and chloroplast genome-NC_004543.1) were annotated using BLASTX prediction as implemented in the software PREPACT2[69] (http://www.prepact.de/prepact-main.php) with default BLAST options.

A total of 1,156 PPR proteins were identified in *A. angustus*, consisting of 65 P-class proteins and 1,091 PLS-class proteins (**Supplementary Tables 23** and **24**). In the PLS-class of PPR proteins, 516 E-type PPR proteins, 337 DYW-type PPR proteins, and 238 PLS-type PPR proteins were included (**Supplementary Tables 23** and **24**). Obviously, the number of PLS-class PPR proteins is far greater than that of the P-class PPR proteins (**Supplementary Table 23**). Most of the *A. angustus* PLS-class PPR proteins were predicted to localize to the mitochondria or chloroplasts (**Supplementary Table 24**). The organellar genomes of *A. angustus* hold 970 chloroplast editing sites and 550 mitochondrial editing sites (**Supplementary Table 23**). The PLS-class *PPR* genes function mainly in RNA editing. Our inferences suggested that the expansion of PLS-class *PPR* genes is correlated with the large number of RNA editing sites estimated in organellar genomes of *A. angustus* (**Supplementary Table 23**). The *PPR* genes, especially the PLS-class *PPR* genes, are much more numerous in both *A. angustus* and the lycophyte *Selaginella moellendorffii* than in other land plants, which is consistent with the large number of RNA editing sites in their organellar genomes (**Supplementary Table 23**). In contrast, no PLS-class *PPR* genes were found in the charophyte green alga *Klebsormidium nitens* and chlorophyte green alga *Chlamydomonas reinhardtii*[66]. In the liverwort *Marchantia polymorpha*, only one PLS-class *PPR* gene was identified[17], and no RNA editing sites were found in its organellar genomes[70,71], indicating that the editing ability may have been lost during the evolution of *M. polymorpha*[65].

S5.2. Cupin gene family

The extensive superfamily of cupins combines functionally and structurally diverse proteins,

covering a wide variety of enzymes as well as non-enzymatic seed storage proteins[72,73]. According to the number of cupin domains, cupins can be divided into two groups: monocupins with a single copy of the cupin domain and bicupins with two copies of the cupin domain[73,74]. Germins/oxalate oxidases (G-OXOs) and germin-like proteins (GLPs) are two well-known plant monocupins[75,76]. G-OXOs and GLPs have been implicated in cell wall modification and in the response of plants to biotic and abiotic stress[77,78]. The bicupin group mainly contains plant seed storage globulin proteins (11S legumin type and 7S vicilin type) and microbial oxalate decarboxylases (OD)[73–75]. Globulin protein provides essential nutrition for seed germination and development[73] and is also involved in desiccation tolerance[79].

We investigated members of the plant cupin family from the charophyte *K. nitens*, the hornwort *A. angustus*, the liverwort *M. polymorpha*, the moss *Physcomitrella patens*, the lycophyte *Selaginella moellendorffii*, the gymnosperm *Picea abies*, the angiosperms *Amborella trichopoda*, *Arabidopsis thaliana*, and *Oryza sativa* by performing an HMMER search using the cupin_1 domain (PFAM profile PF00190) as a query against these plant proteomes. To distinguish between monocupins and bicupins, we first examined the number of cupin domains for each cupin protein and then constructed an alignment and phylogenetic tree for monocupins and bicupins, respectively. The full-length protein sequences were aligned using ClustalW in Geneious 8.1.9 program and then were manually adjusted for alignments. The detailed phylogenetic analysis is described in the **Methods** section. The *A. angustus* genome encodes 79 cupin proteins, which is the highest among the nine species under investigation (**Fig. 3a** and **Supplementary Table 25**). Among these *A. angustus* cupins, 31 are bicupins, and 48 are monocupins (**Fig. 3a**). The number of bicupins in *A. angustus*, in particular, is extremely higher than that in other green plants (**Fig. 3a**). The liverwort *M. polymorpha* exhibits the most monocupins among the nine species but contains only one bicupin protein (**Fig. 3a**). By contrast, the moss *P. patens* has a relatively small cupin family, including 20 monocupins and no bicupins. The specific expansion of *cupin* genes in *A. angustus* and *M. polymorpha* may be related to their specific biological properties. The differences in gene dosage and the functional diversity of the *cupin* gene family among these three lineages of bryophytes need to be further studied.

In the absence of WGD in *A. angustus*, this gene family expansion might result from small-scale gene duplications. Tandem duplication events mainly contribute to the evolution of the *cupin* gene family in many angiosperms[80,81], and likely account for the novel copies of *cupin* genes in *A. angustus*. The *cupin* genes located in the same scaffold, separated by a maximum of five genes, and clustered in a clade were considered tandem duplicated genes. Our phylogenetic analysis revealed that *A. angustus bicupin* genes were mainly distributed in three clades, and two of the three clades resulted from *A. angustus*-specific expansion (**Supplementary Fig. 56**). Thirteen *A. angustus bicupin* genes were arranged in tandem (**Fig. 3b**). The *A. angustus monocupin* genes composed three clades, with the largest one containing 25 members that resulted from an *A. angustus*-specific expansion (**Supplementary Fig. 57**). Twenty-seven *A. angustus monocupin* genes were present in tandem (**Fig. 3c**). Thus, tandem duplications appear to play an important role in the expansion of the *cupin* gene family in *A. angustus*.

S5.3. Cytochrome P450s

The c*ytochrome P450* (*CYP*) genes make up one of the largest and oldest gene superfamilies in plants and participate in a variety of biochemical pathways to produce primary and secondary metabolites such as phenylpropanoids, terpenoids, and lipids, as well as plant growth regulators such as auxin, gibberellins, and jasmonic acid[82–85]. These metabolites function as growth and developmental signals and mediate biotic and abiotic stress responses[82,85]. Some species-specific P450 families/subfamilies further drive the evolution of species-specialized chemical diversity[84]. Plant CYPs were initially classified into two main clades, A-type and non-A-type[86].

Arabidopsis, *Physcomitrella*, and *Selaginella* CYP proteins were obtained from the P450 homepage (http://drnelson.uthsc.edu/cytochromeP450.html), and *Marchantia* CYPs from the *M. polymorpha* genome[17]. The *Anthoceros*, *Klebsormidium*, and *Chlamydomonas* CYPs were identified by performing an HMMER search using the Cytochrome P450 domain (PFAM profile PF00067) as a query against these plants' proteomes (genome version shown in **Supplementary Table 13**). The raw protein data were obtained through a BLASTP search to primarily identify their clan classification. Possible pseudogenes and highly divergent sequences were included in CYP counting but removed from the phylogenetic analysis. The P450 domain regions of these protein sequences were retrieved, and the corresponding sequences were aligned using ClustalW and used to build the phylogenetic tree on CIPRES (see **Methods**). Our phylogenetic analysis of CYP proteins from *A. thaliana*, *S. moellendorffii*, *P. patens*, *M. polymorpha*, *A. angustus*, *K. nitens*, and *C. reinhardtii* revealed 12 plant CYP clans, CYP51, CYP71, CYP72, CYP74, CYP85, CYP86, CYP97, CYP710, CYP711, CYP746, CYP727, and CYP747 (**Supplementary Figs. 58–61,** and **Supplementary Tables 26** and **27**). The CYP number in five land plants was much higher than that in two green algae, suggesting a significant expansion of CYPs during the water-to-land transition, especially for CYP71 clan (**Supplementary Table 26**). In total, 135 non-redundant *CYP* genes were identified in the *A. angustus* genome, which were classified in ten clans except for *CYP711* and *CYP746* (**Supplementary Table 26**). The CYP71 and CYP85 clans were the two largest clans in *A. angustus*, containing 56 and 46 members, respectively (**Supplementary Table 26**). The *A. angustus CYP* genes were further assigned to 24 KEGG pathways, of which 'flavonoid 3'-monooxygenase' and 'abscisic acid 8'-hydroxylase' were the most representative (**Supplementary Table 28**). *Anthoceros angustus* also retained the member of green algae *CYP747* genes, which all other land plants have lost (**Supplementary Table 26**).

Among the *CYP71* clan (A-type *CYP* genes), the *CYP73*, *CYP98*, *CYP78*, *CYP701* and *CYP703* families are conserved in land plants, and *A. angustus* has homologs for all these families (**Supplementary Figs. 58** and **59**). The CYP73 (C4H, cinnamate 4-hydroxylase) and CYP98 (C3'H, coumaryl shikimic acid/spermidine meta-hydroxylases) catalyse the first two hydroxylations of the phenolic ring in the phenylpropanoid pathway[87]. This pathway generates precursors for land plant-associated compounds such as lignins ('wood molecules') and flavonoids (UV protectants)[82]. In the moss *P. patens*, *PpCYP98* controls the formation of cuticle, an ancestral structure for stress tolerance in terrestrial environments, which shares common features with lignin, cutin and suberin[88]. *Anthoceros angustus* has three *CYP73* genes (AANG008092, AANG004216, and AANG012089) and one *CYP98* gene (AANG006992) (**Supplementary Figs. 58** and **59**), which need functional investigation to explore the ancestral roles of these key factors for land

colonization by plants.

In *P. patens*, *CYP78* genes function in protonema growth and gametophore formation[89]. One *CYP78* (AANG000841) was identified in *A. angustus* (**Supplementary Fig. 59**). The *CYP701* family in the *CYP71* clan and the *CYP88* family in the *CYP85* clan encode ent-kaurenoic acid oxidases, which are involved in gibberellin biosynthesis[90,91]. In *P. patens*, *CYP701B1* is involved in the synthesis of the diterpene-derived regulator for the blue-light avoidance response[92]. In our analysis, *CYP701* members were found in all investigated land plants, and one *CYP701* (AANG004667) was identified in *A. angustus* (**Supplementary Fig. 58**). Four *A. angustus* (AANG008406, AANG007216, AANG010861, and AANG009306) and three *M. polymorpha* *CYP88* members were found, while the *CYP88* family member was absent in *P. patens* (**Supplementary Fig. 60**). The *CYP703* family has been suggested to be involved in spore/pollen wall development in land plants[93]. Three *CYP703* (AANG004926, AANG004927, and AANG004929) were identified in *A. angustus* (**Supplementary Fig. 59**). In the *CYP71* clan, *CYP* genes with KEGG annotation as flavonoid 3'-monooxygenase/flavonoid 3'-hydroxylase (F3'H) are highly abundant in *A. angustus* (**Supplementary Table 28**). F3'Hs are involved in flavonoid biosynthesis, the components of which play an important role in UV-B protection[94]. F3'Hs are encoded by members of the *CYP75* family[95]. In our analysis, the typical *CYP75* family only includes members of flowering plants, and the *A. angustus*-specific expanded F3'H CYPs did not group together with this clade (**Supplementary Fig. 59**). It seems that *Anthoceros* F3'Hs represent a new *CYP* family that has evolved independently of angiosperm F3'Hs (**Supplementary Fig. 59**).

Among the non-A-type *CYP* genes, the *CYP51*, *CYP710*, and *CYP97* families are conserved from green algae to land plants (**Supplementary Figs. 60** and **61**). The members of the *CYP51* and *CYP710* families are involved in sterol biosynthesis[82,83]. The *Anthoceros* genome encodes one *CYP51* and two *CYP710* genes (**Supplementary Fig. 60**). The *CYP97* family functions in xanthophyll (hydroxylated carotenoid) biosynthesis[82] and is divided into three subfamilies, *CYP97A*, *CYP97B*, and *CYP97C*, that arose before the divergence of green plants (**Supplementary Fig. 61**). The *A. angustus* genome holds three *CYP97* genes, one in each subfamy: AANG012455 in *CYP97A*, AANG011020 in *CYP97B*, and AANG009798 in *CYP97C* (**Supplementary Fig. 61**). The *CYP74* family is the only family within the *CYP74* clan. The *CYP74* family participates in the synthesis of oxylipin derivatives and allene oxide in the octadecanoid and jasmonate pathways and further mediates plant defence against herbivores, pathogens, or abiotic stresses[82,83,96]. Our analysis suggested that the *CYP74* family is conserved in streptophytes (**Supplementary Fig. 60**), which is consistent with the finding of Koeduka et al. (2015)[97]. A single *CYP74* gene (AANG012100) occurs in *A. angustus* (**Supplementary Fig. 60**).

The *CYP72*, *CYP86*, and *CYP85* clans have expanded dramatically and possess multiple families, similar to the *CYP71* clan (**Supplementary Figs. 58–61**). The members of the *CYP72* clan are involved in the metabolism of hydrophobic compounds, such as fatty acids, isoprenoids, cytokinins, brassinosteroids and gibberellins[83]. Thirteen members of the *CYP72* clan occur in the *A. angustus* genome, versus five in *P. patens* and eight in *M. polymorpha* (**Supplementary Fig. 61**). In the *CYP86* clan, the *CYP86*, *CYP94* and *CYP704* families are conserved in land plants, and

A. angustus contains three *CYP86* genes, three *CYP94* genes, and five *CYP704* genes (**Supplementary Fig. 61**). The *CYP86* and *CYP94* families encode fatty acid hydroxylases or alkane hydroxylase, which contribute essential biomolecules that cover surfaces including aerial parts, roots, and pollen[82]. CYP704s are also fatty acid hydroxylases and are involved in sporopollenin and cutin biosynthesis[98,99]. Compared to that of other land plants, the *CYP85* clan in *A. angustus* is significantly expanded and contains 46 members (**Supplementary Fig. 60** and **Supplementary Table 26**). Members of the *CYP85* clan function in the modification of cyclic terpenes and sterols in brassinosteroids[100], abscisic acid (ABA)[101] and gibberellins[91], which are important metabolites in plant development and environmental response. In the *CYP85* clan, the *CYP707A* family (*ABA 8'-hydroxylase* genes) in angiosperms functions in ABA catabolism and is further involved in drought stress response and seed germination[102,103]. Based on the KEGG annotation, the expanded members of the *CYP85* clan in *A. angustus* mainly encode ABA 8'-hydroxylases, and some members even form an independently new *CYP* family (**Supplementary Table 28**), implying that desiccation tolerance is likely an ancestral trait for land plants and that its molecular mechanism might combine conservative and specific features.

S5.4. Tandem duplication

Tandem duplication of genes plays a more important role in shaping genomes for stress adaptations than WGDs, segmental transposition-duplications, or ectopic duplication and translocation[104,105]. In *A. angustus*, no evidence of WGD was found (**Fig. 1c**); meanwhile, tandem duplication events have been suggested to contribute to the expansion of the *cupin* family (**Figs. 3b,c**). Therefore, we investigated TAGs and corresponding tandem duplications in the *A. angustus* genome.

According to arrays of zero, one, five, or ten adjacent paralogs (see **Methods**), the TAGs in *A. angustus* ranged from 1,436 to 2,621 (**Supplementary Table 30**). Therefore, at least 9.82% of the protein-coding genes in *A. angustus* occur in the tandem array (**Supplementary Table 30**). Based on the gene annotation data, we found that the gene family expansion in *A. angustus* mostly resulted from tandem gene duplications, similar to the case in the *cupin* family (**Supplementary Table 29**). As mentioned above, these expanded families are mainly involved in the adaptive response to environmental stimuli (**Supplementary Notes S5.1–5.3**). Accordingly, the tandem gene duplications might contribute to the adaptive evolution of the hornwort *A. angustus* during the colonization of terrestrial ecosystems.

S6. CO_2 concentrating mechanism

S6.1. pyrenoid and CO_2-concentrating mechanism in hornworts

Hornworts are considered as the only extant land plant lineage with the pyrenoid-based CO_2-concentrating mechanism (CCM) relying on multiple energized inorganic carbon (Ci) uptake systems that is also present in green algae[13,106,107]. Pyrenoids are non-membrane-bound, proteinaceous structures found inside chloroplasts, which contain a matrix packed with the

CO_2-fixing enzyme Rubisco. It has been suggested that the ancestor of hornworts lacked pyrenoids and hornworts may have re-evolved the pyrenoid during evolution[14,106]. Further, the pyrenoids were gained and lost several times within hornworts over the last 100 Mya under varying concentrations of atmospheric CO_2[14]. The pyrenoid is present in *Anthoceros*, while *A. angustus* contains a starch-free area that resembles a pyrenoid[14,108]. Since pyrenoid-containing and pyrenoid-lacking species occur in various terrestrial environments, the advantage of a pyrenoid-based CCM in hornworts is puzzling[13,106]. The function of the pyrenoid in hornworts remains one of many unanswered ecophysiological questions, since no other extant land plants occupying similar habitats have the unique plastid like hornworts[13].

S6.2. CO_2-concentrating mechanism components

Many of CCM components appear to be unique to green algae[109,110]. Recently, homologs of the green alga *Chlamydomonas reinhardtii LCIB* (low-CO_2 inducible B gene) have been identified in hornwort transcriptomes and genomes[107], however, no sequences have been published. Here, in order to check if hornworts recruit similar CCM genes of green algae, we used *C. reinhardtii* CCM related sequences[109,111,112] as queries via BLASTP search against the genome data of target species inlcuding *A. angustus* and other ten green plants (*Arabidopsis thaliana*, *Oryza sativa*, *Selaginella moellendorffii*, *Physcomitrella patens*, *Marchantia polymorpha*, *Klebsormidium nitens*, *Chara braunii*, *Volvox carteri*, *Chlorella variabilis*, and *Coccomyxa subellipsoidea*) (*E*-value < 1e-7). The CCM genes without orthologs from investigated land plant genomes were further used to align with the transcriptome sequences from the 1KP database (http://www.onekp.com/public_read_data.html) via TBLASTN. Then we performed phylogenetic inferences for each gene clusters (**Supplementary Figs. 62–71**), except for *EPYC1* (Cre10.g436550.t1.2 from *C. reinhardtii* and XP_002946604.1 from *Volvox carteri*) and *LCI1* (Cre03.g162800.t1.2 from *C. reinhardtii* and WNGH_scaffold_2086979 from moss *Aulacomnium heterostichum*) that only have limited sequence data. The summary of the putatvie CCM componets in green plants is shown in **Supplementary Fig. 72.**

S7. Horizontal gene transfer

S7.1. Identification of *A. angustus*-specific HGTs

The taxonomic distribution of the initial BLASTP results indicated that some of the hornwort *Anthoceros angustus* genes are possibly transferred from bacteria or fungi (**Supplementary Fig. 6**). In *A. angustus*, the best BLAST hits of 94 sequences are bacterial sequences, and 63 are fungal sequences (**Supplementary Fig. 6**). We used these *A. angustus* sequences to BLAST against the *A. angustus* transcriptome mentioned in the **Methods** and found a total of 86 candidates with transcript BLAST hits. These candidates were further used to perform fine BLAST against the latest NCBI non-redundant (nr) protein database with the parameter of 1,000 max target sequences. To avoid the effects of low-quality sequences and contamination from microbe sequences[113] in identifying putative HGTs, we used a series of parameters to filter the candidates, including 30–70% identity with the best hits that are from bacteria or fungi, more than 150 amino acids, and more

than 50% amino acid coverage of blast alignment on query sequence. Candidates that did not meet any one of these criteria were excluded. Through manual inspection, 19 sequences were found to be putative HGT candidates that might be specific to *A. angustus* (**Supplementary Table 31**). All these genes were embedded among typical plant genes on *A. angustus* scaffolds and do not seem to be contaminated sequences.

The HGT candidates were further checked through phylogenetic analysis, in which *A. angustus* genes clustered with only bacterial or fungal clades or formed a sister group with clades that included only bacterial or fungal genes. The homologous sequences from organisms other than the kingdom including the HGT donors (or when unavailable, the distant related bacteria or fungi) were used as outgroup in rooting the trees. The corresponding homologs from other species used in phylogenetic analysis were retrieved based on the taxonomic distribution of fine BLAST results, following the criteria, a reciprocal coverage of >50% and a minimum of 30% amino acid identity in the aligned region. The full-length protein sequences were used in the alignment. The detailed alignment and phylogenetic analysis are shown in the **Methods** section. Finally, 19 candidates in *A. angustus* were confirmed as horizontally transferred genes from bacteria or fungi by performing phylogenetic analysis to verify the reliability of HGT (**Fig. 4a**,**b, Supplementary Figs. 73–84** and **Supplementary Table 31**). Genomic structural and functional annotations indicate that the up- and down-stream genes of these *Anthoceros* HGT genes are annotated as plant genes (**Supplementary Figs. 73–84** and **86a–d**).

S7.2. Identification of bryophyte-specific HGTs

In this study, we investigated the putative HGTs shared only by *A. angustus* and other bryophytes (*Physcomitrella patens* and/or *Marchantia polymorpha*). We extracted 268 gene families (clusters) only shared by three bryophytes or only shared by *A. angustus* and *P. patens* or only shared by *A. angustus* and *M. polymorpha* among the gene families (clusters) (see **Methods** and **Fig. 1a)**. We submitted the corresponding *A. angustus* members of each cluster to the NCBI Web BLAST service (nr) and checked the taxonomy report of the top 1,000 BLAST hits. In addition to the best BLAST hits of *P. patens* and *M. polymorpha*, the following top BLAST hits come from bacteria or fungi with identities from 30% to 70% and coverage higher than 50%. If the BLAST results met the above criteria, the corresponding genes were treated as bryophyte-specific HGT candidates. Through the confirmation of phylogenetic analysis following the same pipeline mentioned above (see **Methods**) and the investigation of homologs of published HGTs in *P. patens*[114] and *M. polymorpha*[17], we confirmed two HGTs from bacteria only shared by three bryophytes and one HGT from fungi shared only between *A. angustus* and *M. polymorpha* (**Fig. 4c,d, Supplementary Fig. 85** and **Supplementary Table 31**). Genomic structural and functional annotations indicate that the up- and down- stream genes of these *Anthoceros* HGT genes are annotated as plant genes (**Supplementary Figs. 85b**, **86e**,**f**).

References

1 Goffinet, B. & Shaw, A. J. (eds.). *Bryophyte Biology* (2nd edition) 565 p (Cambridge University Press, 2009).

2 Kürschner, H. Life strategies and adaptations in bryophytes from the Near and Middle East. *Turk. J. Bot.* **28**, 73–84 (2004).

3 Ligrone, R., Duckett, J. G. & Renzaglia, K. S. Major transitions in the evolution of early land plants: a bryological perspective. *Ann. Bot.* **109**, 851–871 (2012).

4 Jones, V. A. & Dolan, L. The evolution of root hairs and rhizoids. *Ann. Bot.* **110**, 205–212 (2012).

5 Adams, D. G. & Duggan, P. S. Cyanobacteria-bryophyte symbioses. *J. Exp. Bot.* **59**, 1047–1058 (2008).

6 Tang, J. Y., Ma, J., Li, X. D. & Li, Y. H. Illumina sequencing-based community analysis of bacteria associated with different bryophytes collected from Tibet, China. *BMC Microbiol.* **16**, 276 (2016).

7 Santi, C., Bogusz, D. & Franche, C. Biological nitrogen fixation in non-legume plants. *Ann. Bot.* **111**, 743–767 (2013).

8 Zhang, Y. & Guo, L. D. Arbuscular mycorrhizal structure and fungi associated with mosses. *Mycorrhiza* **17**, 319–325 (2007).

9 Bidartondo, M. I. & Duckett, J. G. Conservative ecological and evolutionary patterns in liverwort-fungal symbioses. *Proc. R. Soc. B* **277**, 485–492 (2010).

10 Desirò, A., Duckett, J. G., Pressel, S., Villarreal, J. C. & Bidartondo, M. I. Fungal symbioses in hornworts: a chequered history. *Proc. R. Soc. B* **280**, 1759 (2013).

11 Field, K. J., Pressel, S., Duckett, J. G., Rimington, W. R. & Bidartondo, M. I. Symbiotic options for the conquest of land. *Trends Eco. Evol.* **30**, 477–486 (2015).

12 Hoysted, G. A. et al. A mycorrhizal revolution. *Curr. Opin. Plant Biol.* **44**, 1–6 (2018).

13 Villarreal, J. C. & Renzaglia, K. S. The hornworts: important advancements in early land plant evolution. *J. Bryol.* **37**, 157–170 (2015).

14 Villarreal, J. C. & Renner, S. S. Hornwort pyrenoids, carbon-concentrating structures, evolved and were lost at least five times during the last 100 million years. *Proc. Natl. Acad. Sci. USA* **109**, 18873–18878 (2012).

15 Qiu, Y. L. et al. The deepest divergences in land plants inferred from phylogenomic evidence. *Proc. Natl. Acad. Sci. USA* **103**, 15511–15516 (2006).

16 Rensing, S. A. et al. The *Physcomitrella* genome reveals evolutionary insights into the conquest of land by plants. *Science* **319**, 64–69 (2008).

17 Bowman, J. L. et al. Insights into land plant evolution garnered from the *Marchantia polymorpha* genome. *Cell* **171**, 287–304 (2017).

18 Szövényi, P. et al. Establishment of *Anthoceros agrestis* as a model species for studying the biology of hornworts. *BMC Plant Biol.* **15**, 98 (2015).

19 Szövényi, P. The genome of the model species *Anthoceros agrestis*. *Adv. Bot. Res.* **78**, 189–211 (2016).

20 Li, F. et al. Horizontal transfer of an adaptive chimeric photoreceptor from bryophytes to ferns. *Proc. Natl Acad. Sci. USA* **111**, 6672–6677 (2014).

21 Peng, T. & Zhu, R. L. A revision of the genus *Anthoceros* (Anthocerotaceae, Anthocerotophyta) in China. *Phytotaxa* **100**, 21–35 (2013).

22 Villarreal, J. C., Cargill, D. C., Hagborg, A., Soderstrom, L. & Renzaglia, K. S. A synthesis of hornwort diversity: patterns, causes and future work. *Phytotaxa* **9**, 150–166 (2014).

23 Breinholt, J. W. & Kawahara, A. Y. Phylotranscriptomics: saturated third codon positions

radically influence the estimation of trees based on next-gen data. *Genome Biol. Evol.* 5, 2082–2092 (2013).

24 Puttick, M. N. et al. The interrelationships of land plants and the nature of the ancestral embryophyte. *Curr. Biol.* **28**, 733–745 (2018).

25 de Mendoza, A. et al. Transcription factor evolution in eukaryotes and the assembly of the regulatory toolkit in multicellular lineages. *Proc. Natl Acad. Sci. USA* **110**, E4858–E4866 (2013).

26 Lang, D. et al. Genome-wide phylogenetic comparative analysis of plant transcriptional regulation: a timeline of loss, gain, expansion, and correlation with complexity. *Genome Biol. Evol.* **2**, 488–503 (2010).

27 Sakakibara, K. Technological innovations give rise to a new era of plant evolutionary developmental biology. *Adv. Bot. Res.* **78**, 3–35 (2016).

28 Ishizaki, K. Evolution of land plants: insights from molecular studies on basal lineages. *Biosci. Biotechnol. Biochem.* **81**, 73–80 (2017).

29 Rensing, S. A. Great moments in evolution: the conquest of land by plants. *Curr. Opin. Plant. Biol.* **42**, 49–54 (2018).

30 Szövényi, P., Waller, M. & Kirbis, A. Evolution of the plant body plan. *Curr. Top. Dev. Biol.* **131**, 1–34 (2019).

31 Cenci, A. & Rouard, M. Evolutionary analyses of *GRAS* transcription factors in angiosperms. *Front. Plant Sci.* **8**, 273 (2017).

32 Grosche, C., Genau, A. C. & Rensing, S. A. Evolution of the symbiosis-specific *GRAS* regulatory network in bryophytes. *Front. Plant Sci.* **9**, 1621 (2018).

33 Kaplan-Levy, R. N., Brewer, P. B., Quon, T. & Smyth, D. R. The *trihelix* family of transcription factors-light, stress and development. *Trends Plant Sci.* 17, 163–171 (2012).

34 Portereiko, M. F. et al. *AGL80* is required for central cell and endosperm development in *Arabidopsis*. *Plant Cell* **18**, 1862–1872 (2006).

35 Colombo, M. et al. *AGL23*, a type I MADS-box gene that controls female gametophyte and embryo development in *Arabidopsis*. *Plant J.* **54**, 1037–1048 (2008).

36 Kang, I. H., Steffen, J. G., Portereiko, M. F., Lloyd, A. & Drews, G. N. The AGL62 MADS domain protein regulates cellularization during endosperm development in *Arabidopsis*. *Plant Cell* **20**, 635–647 (2008).

37 Gramzow, L. et al. *Selaginella* genome analysis – entering the 'homoplasy heaven' of the MADS world. *Front. Plant Sci.* **3**, 214 (2012).

38 Zobell, O., Faigl, W., Saedler, H. & Münster, T. MIKC* MADS-box proteins: conserved regulators of the gametophytic generation of land plants. *Mol. Biol. Evol.* **27**, 1201–1211 (2010).

39 Kwantes, M., Liebsch, D. & Verelst, W. How MIKC* MADS-box genes originated and evidence for their conserved function throughout the evolution of vascular plant gametophytes. *Mol. Biol. Evol.* **29**, 293–302 (2012).

40 Singer, S. D., Krogan, N. T. & Ashton, N. W. Clues about the ancestral roles of plant MADS-box genes from a functional analysis of moss homologues. *Plant Cell Rep.* **26**, 1155–1169 (2007).

41 Gramzow, L. & Theissen, G. A hitchhiker's guide to the MADS world of plants. *Genome biol.* **11**, 214 (2010).

42 Smaczniak, C., Immink, R. G., Angenent, G. C. & Kaufmann, K. Developmental and evolutionary diversity of plant MADS-domain factors: insights from recent studies. *Development* **139**, 3081–3098 (2012).

43 Thangavel, G. & Nayar, S. A Survey of MIKC type MADS-box genes in non-seed plants: algae, bryophytes, lycophytes and ferns. *Front. Plant Sci.* **9**, 510 (2018).

44 Sakakibara, K. et al. *KNOX2* genes regulate the haploid-to-diploid morphological transition in land plants. *Science* **339**, 1067–1070 (2013).

45 Joo, S. et al. Gene regulatory networks for the haploid-to-diploid transition of *Chlamydomonas reinhardtii*. *Plant Physiol.* **175**, 314–332 (2017).

46 Singer, S. D. & Ashton, N. W. Revelation of ancestral roles of *KNOX* genes by a functional analysis of *Physcomitrella* homologues. *Plant Cell Rep.* **26**, 2039–2054 (2007).

47 Sakakibara, K., Nishiyama, T., Deguchi, H. & Hasebe, M. Class 1 *KNOX* genes are not involved in shoot development in the moss *Physcomitrella patens* but do function in sporophyte development. *Evol. Dev.* **10**, 555–566 (2008).

48 Hay, A. & Tsiantis, M. *KNOX* genes: versatile regulators of plant development and diversity. *Development* **137**, 3153–3165 (2010).

49 Frank, M. H. & Scanlon, M. J. Transcriptomic evidence for the evolution of shoot meristem function in sporophyte-dominant land plants through concerted selection of ancestral gametophytic and sporophytic genetic programs. *Mol. Biol. Evol.* **32**, 355–367 (2015).

50 Coudert, Y., Novák, O. & Harrison, C. J. A KNOX-cytokinin regulatory module predates the origin of indeterminate vascular plants. *Curr. Biol.* **29**, 2743–2750 (2019).

51 Glime, J. M. in *Bryophyte Ecology* (ed Glime, J. M.) Volume 1, Chapt. 2-8 (Michigan Technological University and the International Association of Bryologists, 2017).

52 Plackett, A. R. G., Di Stilio, V. S. & Langdale, J. A. Ferns: the missing link in shoot evolution and development. *Front. Plant Sci.* **6**, 972 (2015).

53 Flores-Sandoval, E. et al. Class C ARFs evolved before the origin of land plants and antagonize differentiation and developmental transitions in *Marchantia polymorpha*. *New Phytol.* **218**, 1612–1630 (2018).

54 Mutte, S. K. et al. Origin and evolution of the nuclear auxin response system. *Elife* **7**, pii: e33399 (2018).

55 Ulmasov, T., Hagen, G. & Guilfoyle, T. J. Activation and repression of transcription by auxin-response factors. *Proc. Natl Acad. Sci. USA* **96**, 5844–5849 (1999).

56 Kato, H. et al. Auxin-mediated transcriptional system with a minimal set of components is critical for morphogenesis through the life cycle in *Marchantia polymorpha*. *PLoS Genet.* **11**, e1005084 (2015).

57 Kato, H. et al. The roles of the sole activator-type auxin response factor in pattern formation of *Marchantia polymorpha. Plant Cell Physiol.* **58**, 1642–1651 (2017).

58 Bowman, J. L., Briginshaw, L. N., Fisher, T. J. & Flores-Sandoval, E. Something ancient and something neofunctionalized-evolution of land plant hormone signaling pathways. *Curr. Opin. Plant Biol.* **47**, 64–72 (2019).

59 Bennett, T. A. et al. Plasma membrane-targeted PIN proteins drive shoot development in a moss. *Curr. Biol.* **24**, 2776–2785 (2014).

60 Mravec J, et al. Subcellular homeostasis of phytohormone auxin is mediated by the ER-localized PIN5 transporter. *Nature* **459**, 1136–1140 (2009).

61 Fujii, S. & Small, I. The evolution of RNA editing and pentatricopeptide repeat genes. *New Phytol.* **191**, 37–47 (2011).
62 Barkan, A. & Small, I. Pentatricopeptide repeat proteins in plants. *Annu. Rev. Plant Biol.* **65**, 415–442 (2014).
63 Cheng, S. et al. Redefining the structural motifs that determine RNA binding and RNA editing by pentatricopeptide repeat proteins in land plants. *Plant J.* **85**, 532–547 (2016).
64 Chateigner-Boutin, A.-L. & Small, I. Plant RNA editing. *RNA Biol.* **7**, 213–219 (2010).
65 Ichinose, M. & Sugita, M. RNA editing and its molecular mechanism in plant organelles. *Genes* **8**, 5 (2017).
66 Cheng, S. et al. Redefining the structural motifs that determine RNA binding and RNA editing by pentatricopeptide repeat proteins in land plants. *Plant J.* **85**, 532–547 (2016).
67 Lurin, C. et al. Genome-wide analysis of *Arabidopsis* pentatricopeptide repeat proteins reveals their essential role in organelle biogenesis. *Plant Cell* **16**, 2089–2103 (2004).
68 Emanuelsson, O., Nielsen, H., Brunak, S. & von Heijne, G. Predicting subcellular localization of proteins based on their N-terminal amino acid sequence. *J. Mol. Biol.* **300**, 1005–1016 (2000).
69 Lenz, H. & Knoop, V. PREPACT 2.0: predicting C-to-U and U-to-C RNA editing in organelle genome sequences with multiple references and curated RNA editing annotation. *Bioinform. Biol. Insights* **7**, 1–19 (2013).
70 Ohyama, K. Chloroplast and mitchondrial genomes from a liverwort, *Marchantia polymorpha*—gene organization and molecular evolution. *Biosci. Biotech. Bioch.* **60**, 16–24 (1996).
71 Rüedinger, M., Polsakiewicz, M. & Knoop, V. Organellar RNA editing and plant-specific extensions of pentatricopeptide repeat proteins in jungermanniid but not in marchantiid liverworts. *Mol. Biol. Evol.* **25**, 1405–1414 (2008).
72 Dunwell, J. M. Cupins: a new superfamily of functionally diverse proteins that include germins and plant storage proteins. *Biotechnol. Genet. Eng. Rev.* **15**, 1–32 (1998).
73 Dunwell, J. M., Purvis, A. & Khuri, S. Cupins: the most functionally diverse protein superfamily? *Phytochemistry* **65**, 7–17 (2004).
74 Dunwell, J. M., Khuri, S. & Gane, P. J. Microbial relatives of the seed storage proteins of higher plants: conservation of structure and diversification of function during evolution of the cupin superfamily. *Microbiol. Mol. Biol. Rev.* **64**, 153–179 (2000).
75 Khuri, S., Bakker, F. T. & Dunwell, J. M. Phylogeny, function, and evolution of the cupins, a structurally conserved, functionally diverse superfamily of proteins. *Mol. Biol. Evol.* **18**, 593–605 (2001).
76 Bernier, F. & Berna, A. Germins and germin-like proteins: plant do-all proteins. But what do they do exactly? *Plant Physiol. Biochem.* **39**, 545–554 (2001).
77 Patnaik, D. & Khurana, P. Germins and germin like proteins: an overview. *Indian J. Exp. Biol.* **39**, 191–200 (2001).
78 Dunwell, J. M., Gibbings, J. G., Mahmood, T. & Naqvi, S. M. S. Germin and germin-like proteins: evolution, structure, and function. *Crit. Rev. Plant Sci.* **27**, 342–375 (2008).
79 Bäumlein, H., Braun, H., Kakhovskaya, I. A. & Shutov, A. D. Seed storage proteins of spermatophytes share a common ancestor with desiccation proteins of fungi. *J. Mol. Evol.* **41**, 1070–1075 (1995).

80 Li, C., Li, M., Dunwell, J. M. & Zhang, Y. M. Gene duplication and an accelerated evolutionary rate in 11S globulin genes are associated with higher protein synthesis in dicots as compared to monocots. *BMC Evol. Biol.* **12**, 15 (2012).
81 Li, L., Xu, X., Chen, C. & Shen, Z. Genome-wide characterization and expression analysis of the germin-like protein family in rice and *Arabidopsis*. *Int. J. Mol. Sci.* **17**, 1622 (2016).
82 Mizutani, M. & Ohta, D. Diversification of P450 genes during land plant evolution. *Annu. Rev. Plant Biol.* **61**, 291–315 (2010).
83 Nelson, D. & Werck-Reichhart, D. A P450-centric view of plant evolution. *Plant J.* **66**, 194–211 (2011).
84 Hamberger, B. & Bak, S. Plant P450s as versatile drivers for evolution of species-specific chemical diversity. *Philos. Trans. R. Soc. Lond. B Biol. Sci.* **368**, 20120426 (2013).
85 Xu, J., Wang, X. Y. & Guo, W. Z. The cytochrome P450 superfamily: key players in plant development and defense. *J. Integr. Agr.* **14**, 1673–1686 (2015).
86 Bak, S. et al. Cytochromes P450. *Arabidopsis Book* **9**, e0144 (2011).
87 Ehlting, J., Hamberger, B., Million-Rousseau, R. & Werck-Reichhart, D. Cytochromes P450 in phenolic metabolism. *Phytochem. Rev.* **5**, 239–270 (2006).
88 Renault, H. et al. A phenol-enriched cuticle is ancestral to lignin evolution in land plants. *Nat. Commun.* **8**, 14713 (2017).
89 Katsumata, T. et al. Involvement of the CYP78A subfamily of cytochrome P450 monooxygenases in protonema growth and gametophore formation in the moss *Physcomitrella patens*. *Biosci. Biotechnol. Biochem.* **75**, 331–336 (2011).
90 Helliwell, C. A., Poole, A., Peacock, W. J. & Dennis, E. S. *Arabidopsis* ent-kaurene oxidase catalyzes three steps of gibberellin biosynthesis. *Plant Physiol.* **119**, 507–510 (1999).
91 Helliwell, C. A., Chandler, P. M., Poole, A., Dennis, E. S. & Peacock, W. J. The CYP88A cytochrome P450, ent-kaurenoic acid oxidase, catalyzes three steps of the gibberellin biosynthesis pathway. *Proc. Natl. Acad. Sci. USA* **98**, 2065–2070 (2001).
92 Miyazaki, S., Nakajima, M. & Kawaide, H. Hormonal diterpenoids derived from ent-kaurenoic acid are involved in the blue-light avoidance response of *Physcomitrella patens*. *Plant Signal. Behav.* **10**, e989046 (2015).
93 Wallace, S., Fleming, A., Wellman, C. H. & Beerling, D. J. Evolutionary development of the plant and spore wall. *AoB Plants* **2011**, plr027 (2011).
94 Agati, G. et al. Functional roles of flavonoids in photoprotection: new evidence, lessons from the past. *Plant Physiol. Biochem.* **72**, 35–45 (2013).
95 Seitz, C., Ameres, S., Schlangen, K., Forkmann, G. & Halbwirth, H. Multiple evolution of flavonoid 3′,5′-hydroxylase. *Planta*, **242**, 561–573 (2015).
96 Hughes, R. K., De Domenico, S. & Santino, A. Plant cytochrome CYP74 family: biochemical features, endocellular localisation, activation mechanism in plant defence and improvements for industrial applications. *Chembiochem.* **10**, 1122–1133 (2009).
97 Koeduka, T. et al. Biochemical characterization of allene oxide synthases from the liverwort *Marchantia polymorpha* and green microalgae *Klebsormidium flaccidum* provides insight into the evolutionary divergence of the plant CYP74 family. *Planta* **242**, 1175–1186 (2015).
98 Dobritsa, A. A. et al. CYP704B1 is a long-chain fatty acid omega-hydroxylase essential for sporopollenin synthesis in pollen of *Arabidopsis*. *Plant Physiol.* **151**, 574–589 (2009).
99 Li, H. et al. Cytochrome P450 family member CYP704B2 catalyzes the omega-hydroxylation

of fatty acids and is required for anther cutin biosynthesis and pollen exine formation in rice. *Plant Cell* **22**, 173–190 (2010).

100 Yokota, T. et al. Occurrence of brassinosteroids in non-flowering land plants, liverwort, moss, lycophyte and fern. *Phytochemistry* **136**, 46–55 (2017).

101 Kushiro, T. et al. The *Arabidopsis* cytochrome P450CYP707A encodes ABA 8'-hydroxylases: key enzymes in ABA catabolism. *EMBO J.* **23**, 1647–1656 (2004).

102 Umezawa, T. et al. CYP707A3, a major ABA 8'-hydroxylase involved in dehydration and rehydration response in *Arabidopsis thaliana*. *Plant J.* **46**, 171–182 (2006).

103 Okamoto, M. et al. CYP707A1 and CYP707A2, which encode abscisic acid 8'-hydroxylases, are indispensable for proper control of seed dormancy and germination in *Arabidopsis*. *Plant Physiol.* **141**, 97–107 (2006).

104 Hanada, K., Zou, C., Lehti-Shiu, M. D., Shinozaki, K. & Shiu, S. H. Importance of lineage-specific expansion of plant tandem duplicates in the adaptive response to environmental stimuli. *Plant Physiol.* **148**, 993–1003 (2008).

105 Oh, D. H., Dassanayake, M., Bohnert, H. J. & Cheeseman, J. M. Life at the extreme: lessons from the genome. *Genome Biol.* **13**, 241 (2012).

106 Hanson, D. T., Renzaglia, K. & Villareal, J. C. in *Photosynthesis of Bryophytes and Early Land Plants* (eds Hanson, D. T. & Rice, S. K.) 1–8 (Springer, 2014).

107 Li, F. W., Villarreal, J. C. & Szövényi, P. Hornworts: an overlooked window into carbon-concentrating mechanisms. *Trends Plant Sci.* **22**, 275–277 (2017).

108 Vaughn, K. C. et al. The anthocerote chloroplast: A review. *New Phytol.* **120**, 169–190 (1992).

109 Meyer, M. & Griffiths, H. Origins and diversity of eukaryotic CO_2-concentrating mechanisms: lessons for the future. *J. Exp. Bot.* **64**, 769–786 (2013).

110 Meyer, M. T. et al. Will an algal CO_2-concentrating mechanism work in higher plants? *Curr. Opin. Plant. Biol.* **31**, 181–188 (2016).

111 Mackinder, L. C. M. A spatial interactome reveals the protein organization of the algal CO_2-concentrating mechanism. *Cell* **171**, 133–147 (2017).

112 Mackinder, L. C. M. The *Chlamydomonas* CO_2 -concentrating mechanism and its potential for engineering photosynthesis in plants. *New Phytol.* **217**, 54–61 (2018).

113 Ku, C. & Martin, W. F. A natural barrier to lateral gene transfer from prokaryotes to eukaryotes revealed from genomes: the 70 % rule. *BMC Biol.* **14**, 89 (2016).

114 Yue, J., Hu, X., Sun, H., Yang, Y. & Huang, J. Widespread impact of horizontal gene transfer on plant colonization of land. *Nat. Commun.* **3**, 1152 (2012).

Supplementary Figures

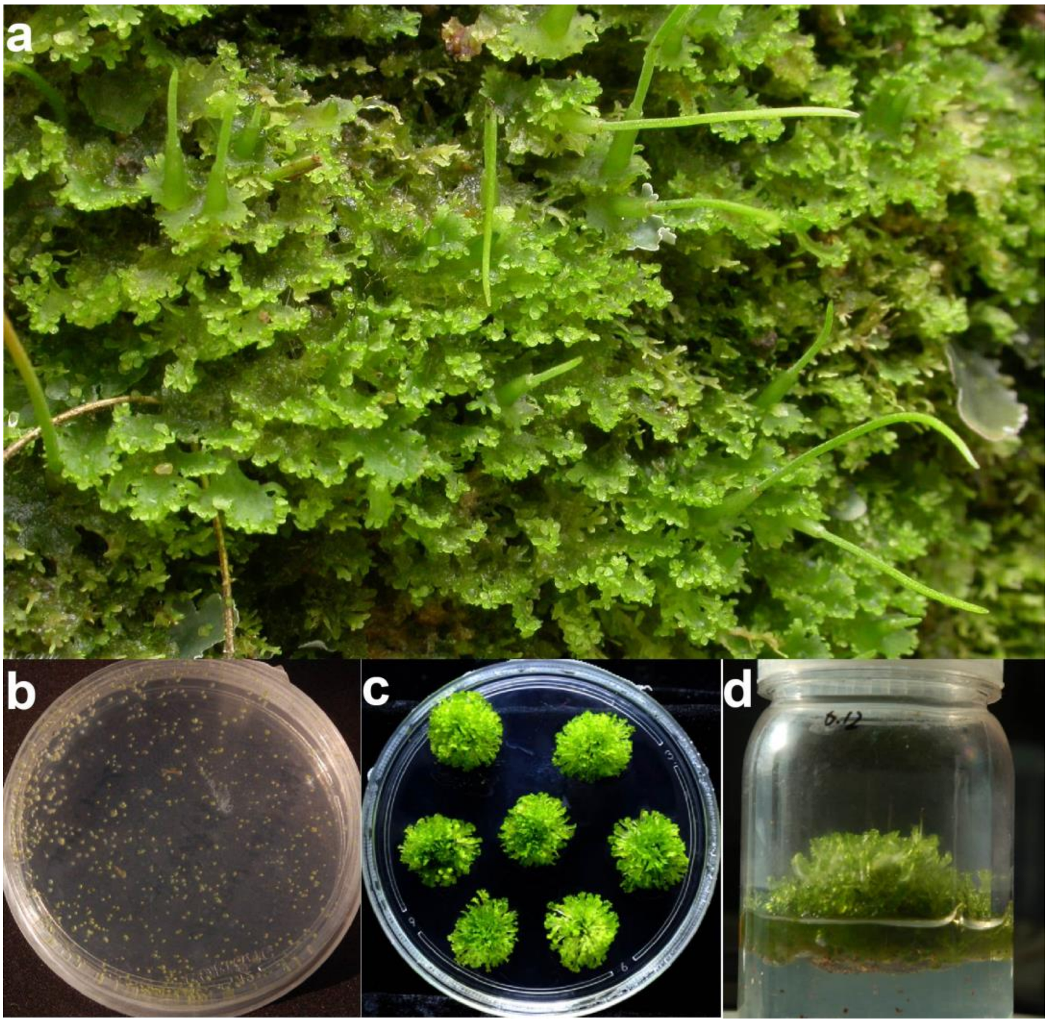

Supplementary Figure 1. The morphology of *Anthoceros angustus*. a, Population in the field. **b-d**, Tissue cultures initiated from spores. **b**, Spores germinating on medium, **c-d**, Gametophyte grown from a single spore. The spore germination experiment was repeated three times independently with similar growth patterns.

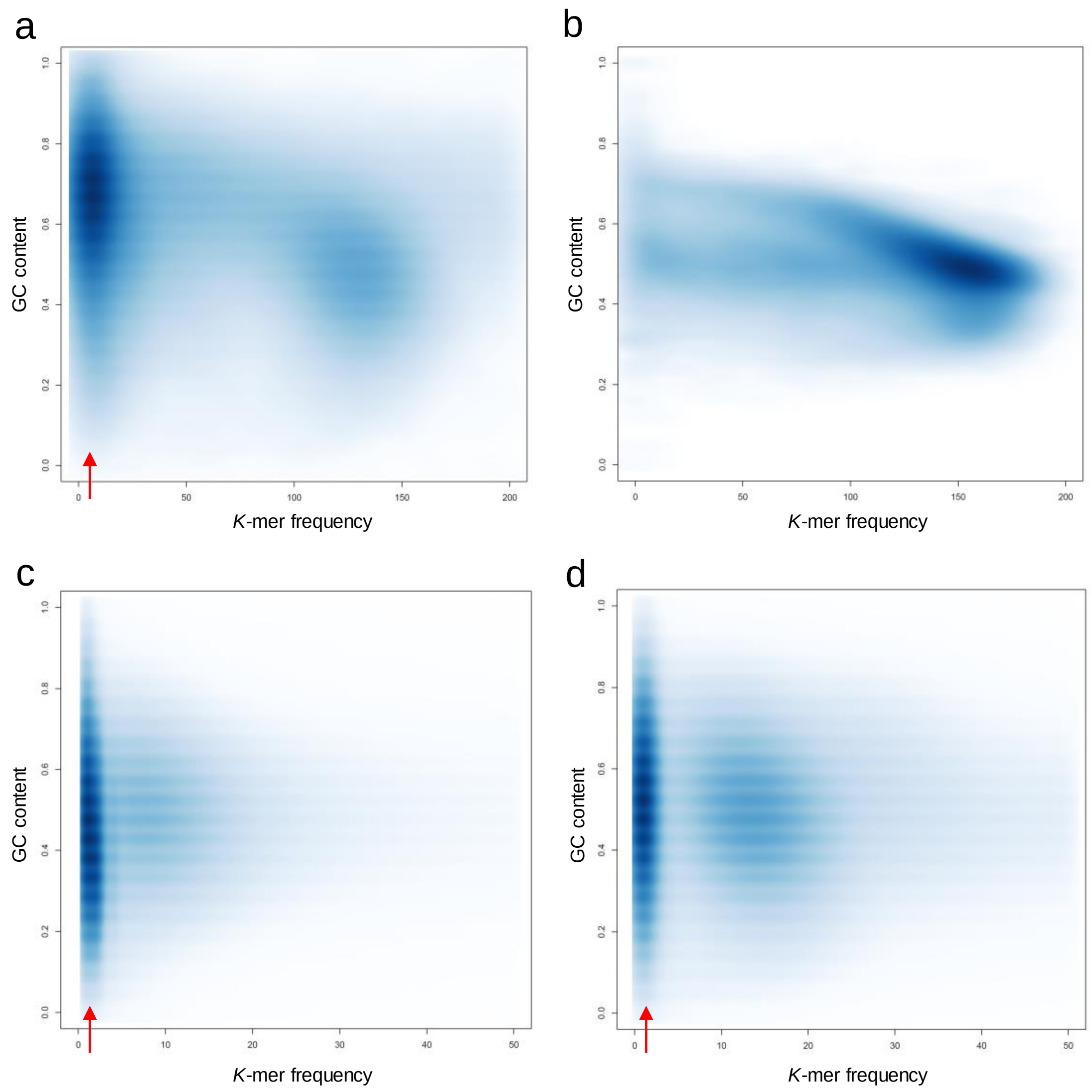

Supplementary Figure 2. Potential contamination within genome sequencing data of hornwort species. a, GC content *vs* *K*-mer frequency of Illumina raw genome sequencing reads of *Anthoceros angustus*. **b**, GC content *vs* *K*-mer frequency of *A. angustus* Illumina raw genome sequencing reads after filtering and decontamination. **c**, GC content *vs* *K*-mer frequency of *A. agrestis* Illumina raw genome sequencing reads of ERX714368. **d**, GC content *vs* *K*-mer frequency of *A. punctatus* raw genome sequencing reads of SRX538621. The red arrow indicates the potential contamination within the genome data.

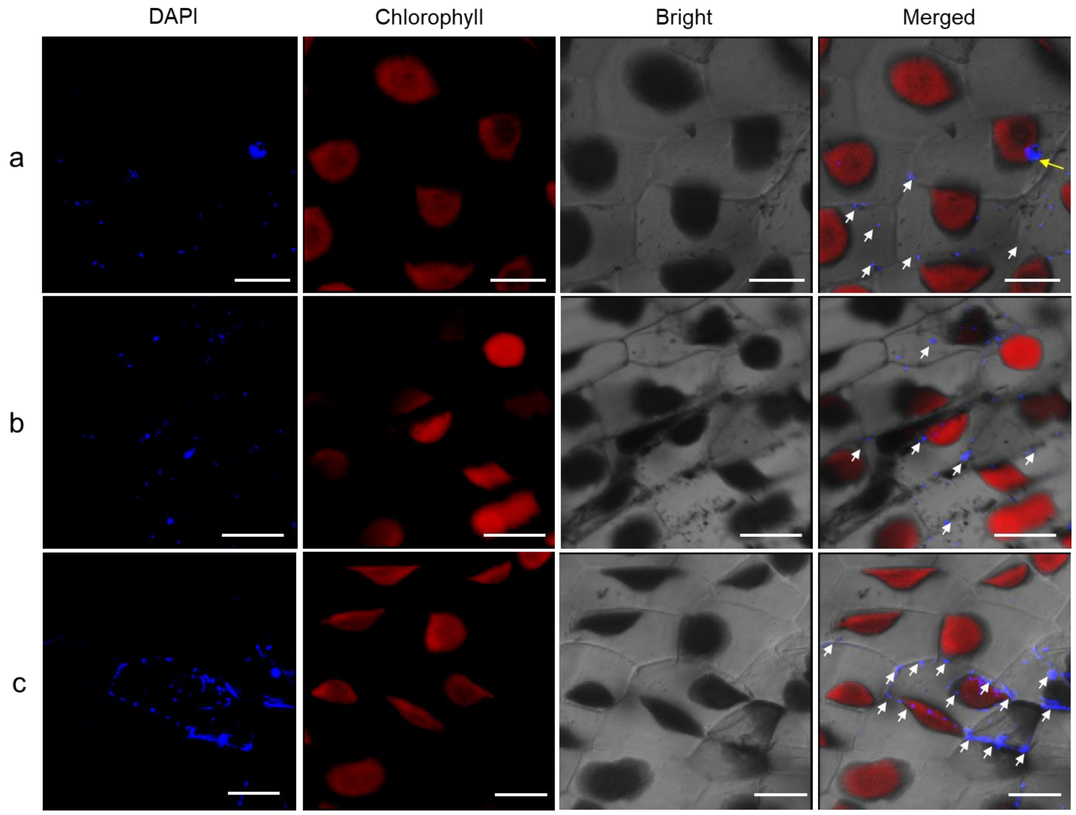

Supplementary Figure 3. DAPI staining of the *Anthoceros angustus* gametophytes. The DAPI staining experiment was repeated three times independently with similar staining patterns. Rows **a-c** indicate the results from different regions of the gametophytes. As shown in merged tunnel, the yellow arrow indicates the cell nucleus, and white arrow indicates the symbiotic bacteria. Scale bar 25μM.

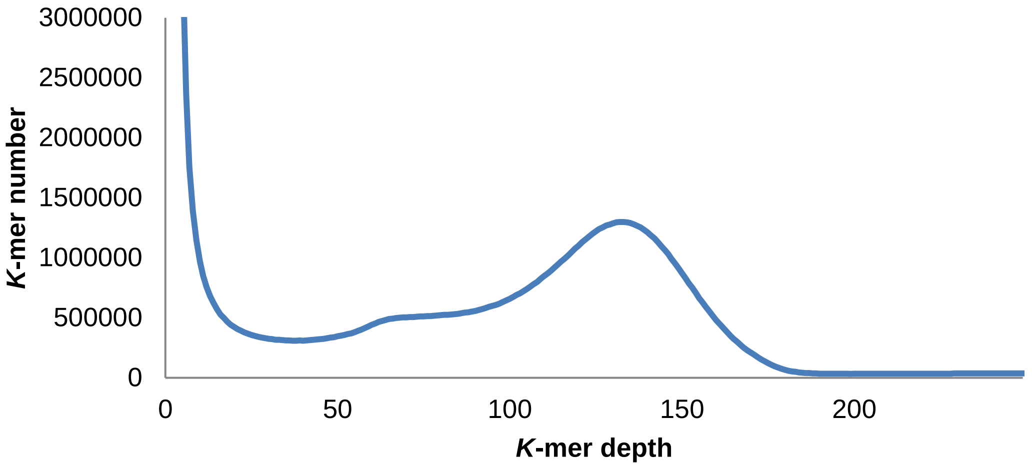

Supplementary Figure 4. *K*-mer distribution of Illumina sequencing reads. Based on this distribution, we estimate the genome of *Anthoceros angustus* to be approximately 107 Mb in size.

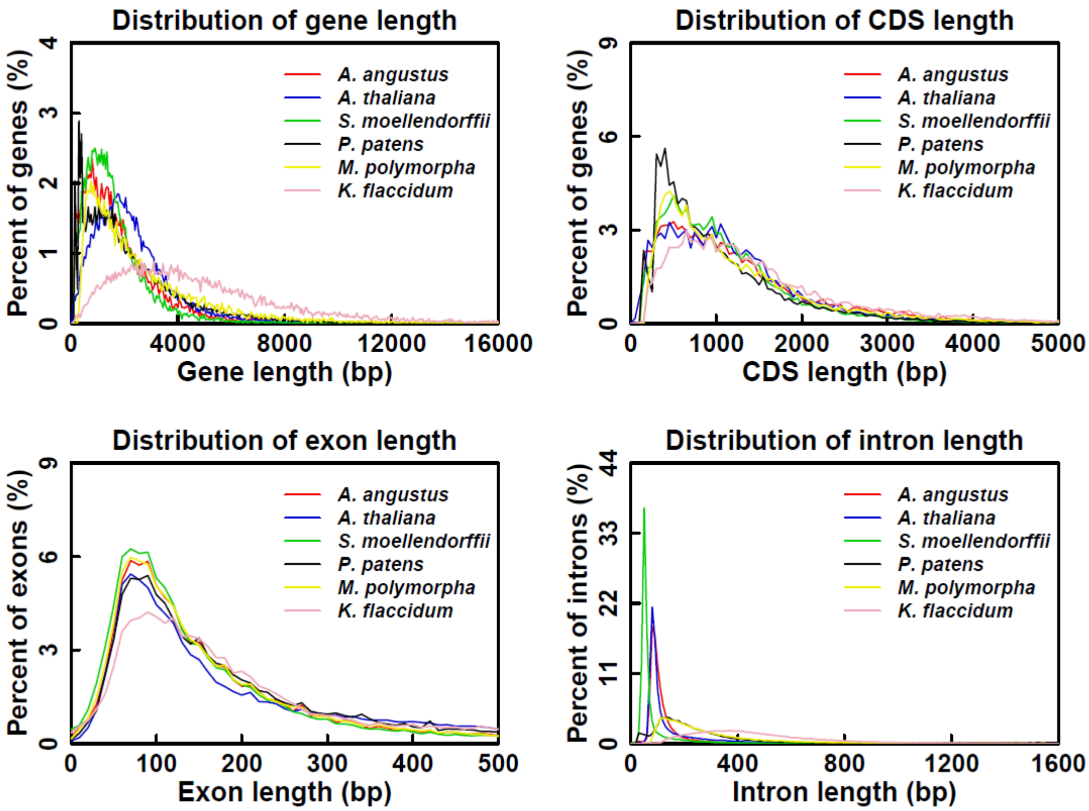

Supplementary Figure 5. Comparison of gene parameters of the *Anthoceros angustus* genome to the *Arabidopsis thaliana*, *Selaginella moellendorffii*, *Physcomitrella patens*, *Marchantia polymorpha*, and *Klebsormidium nitens* genomes. No unexpected differences were observed among *A. angustus* and other land plant genomes, reflecting the similar gene structure among land plants. While significant differences were observed between the genomes of the charophyte green alga *K. nitens* and of land plants.

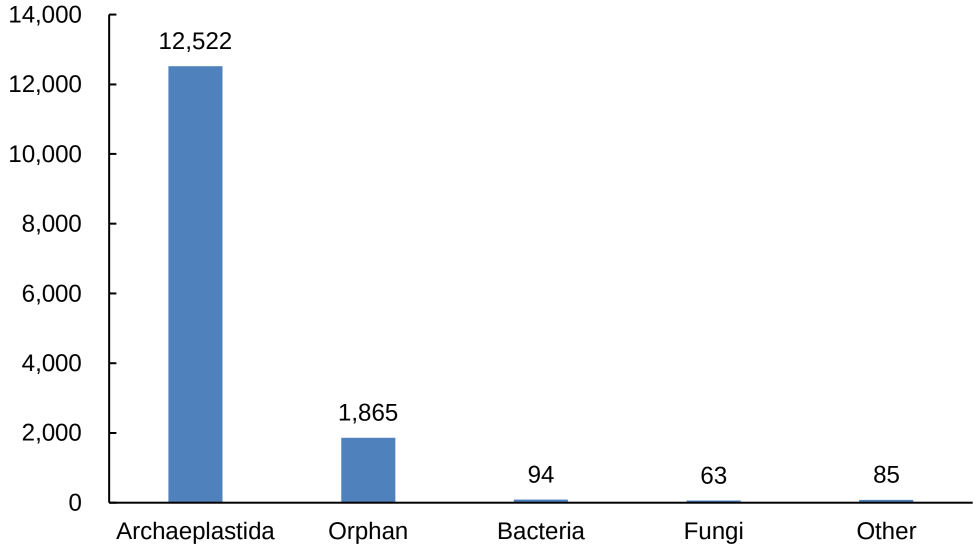

Supplementary Figure 6. Distribution of best BLAST hits of predicted proteins of *Anthoceros angustus* against the NCBI non-redundant (NR) database. BLASTP was performed with an *E*-value cutoff of 1e-5. The classification of the corresponding organisms was extracted according to NCBI taxonomy.

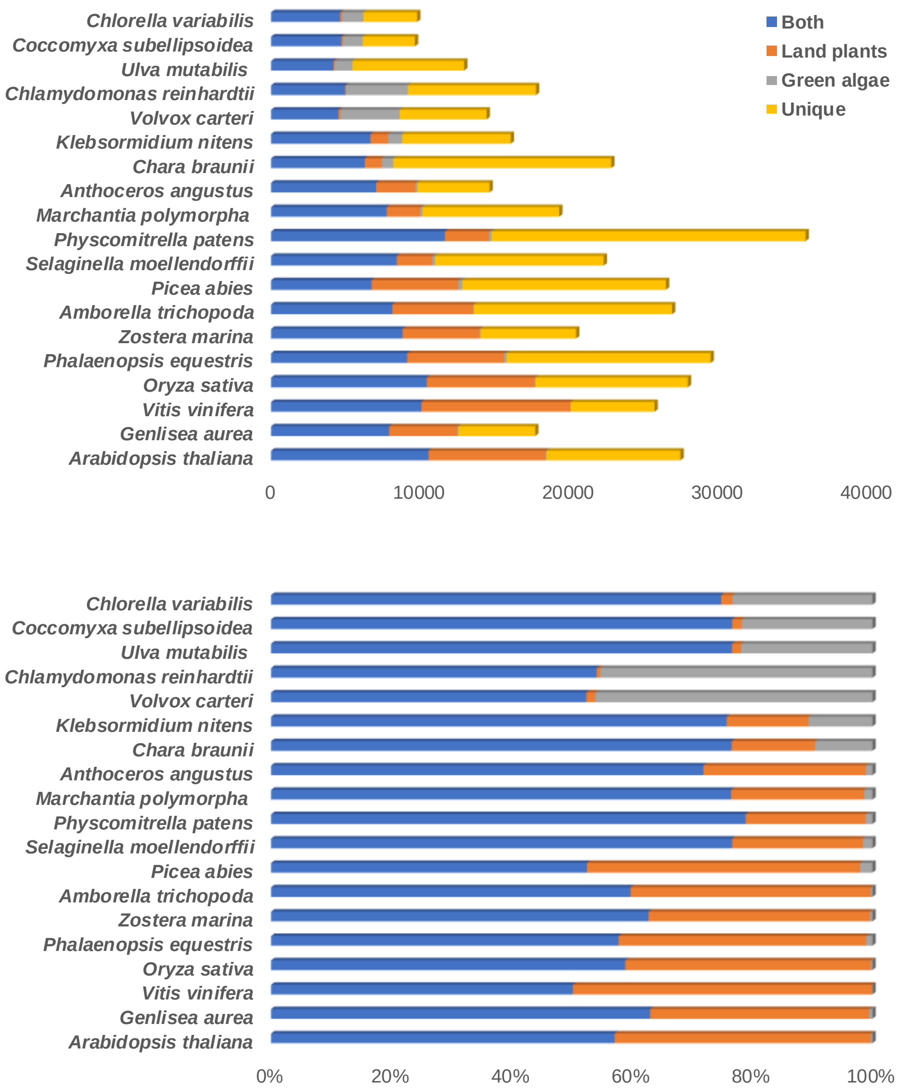

Supplementary Figure 7. Comparative characterization of protein content of 19 green plants. The length of the column represents the number of proteins. Based on classification via OrthoMCL (**Supplementary Table 14**), proteins found both in green algae and land plants represented in blue, proteins only shared among land plants are represented in orange, proteins only shared among green algae are represented in grey, and proteins without reciprocal best hit to other species are represented in yellow. The upper and lower panels represent the number and the percentage of proteins, respectively, and the unique genes (yellow) in each species were excluded for percentage data.

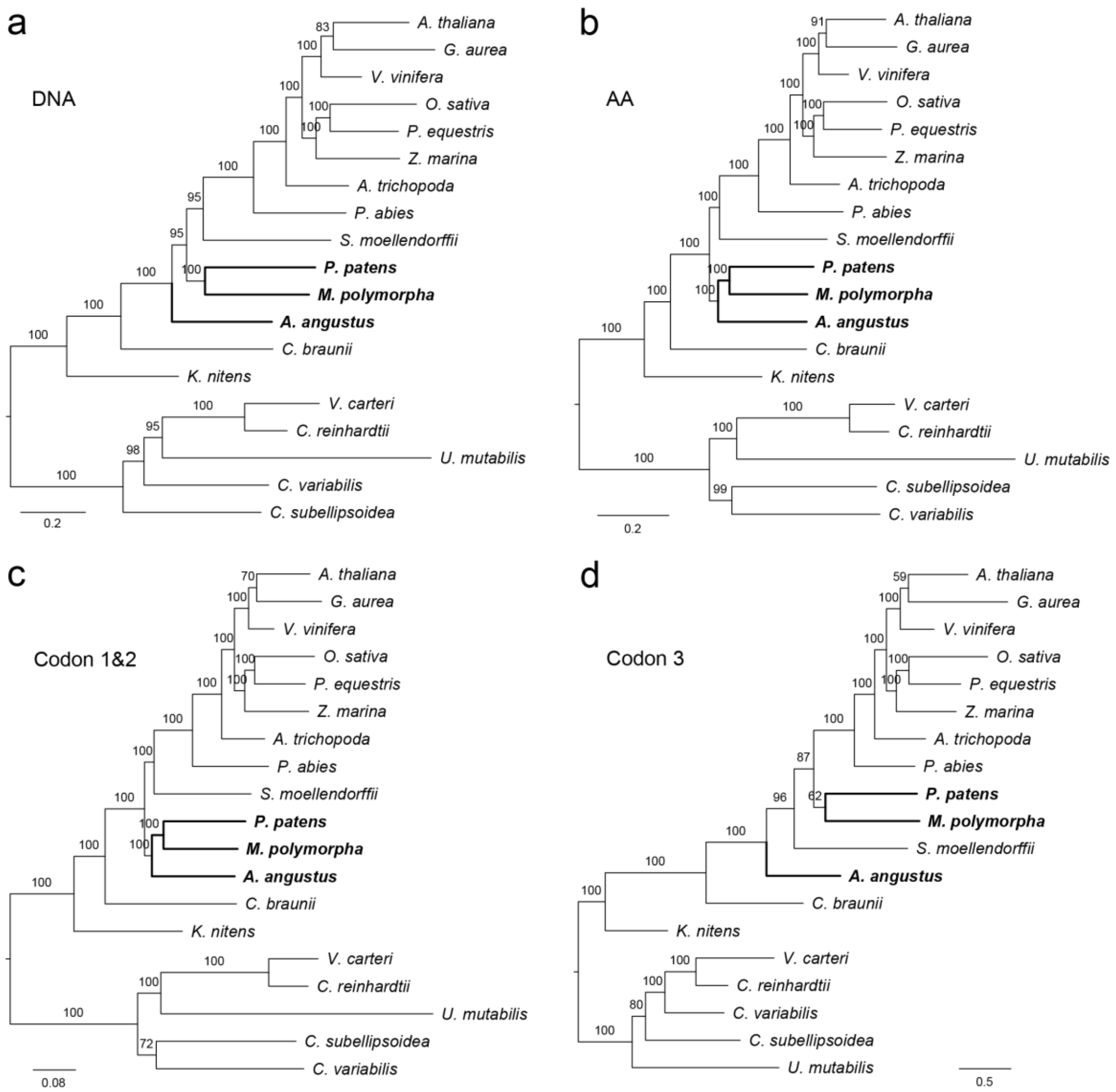

Supplementary Figure 8. Maximum likelihood phylogenies of 19 green plants inferred from 85 single-copy nuclear genes. **a**, Phylogenetic tree based on the in-frame nucleotide alignment with all codon positions. **b**, Phylogenetic tree based on the amino acid alignment. **c**, Phylogenetic tree based on the in-frame nucleotide alignment with first- and second-codon positions. **d**, Phylogenetic tree based on the in-frame nucleotide alignment of only third-codon positions. Bootstrap support values ≥ 50% are shown above the branches.

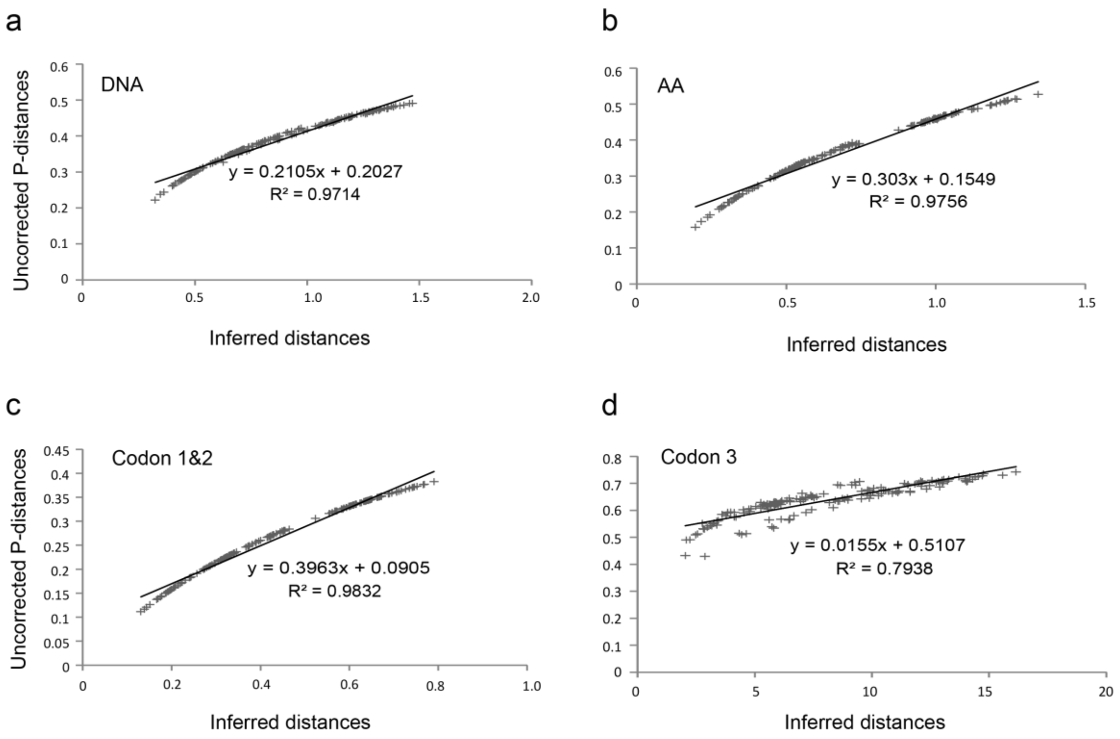

Supplementary Figure 9. Saturation levels of the 19 taxa, 85 single-copy nuclear gene data set, in the concatenated complete nucleotide data (a), amino acid data (b), the first + second codon positions (c), and the third codon positions (d). Pairwise comparisons between all exemplars were performed in each dataset. The uncorrected P-distances were plotted against the inferred distances. The x-axis represents the distance inferred by the maximum-likelihood method, and y-axis represents the uncorrected distance observed for the same taxa pair. The saturation level was estimated by calculating the slope of the regression line, i.e., the lesser the slope, the greater the level of saturation.

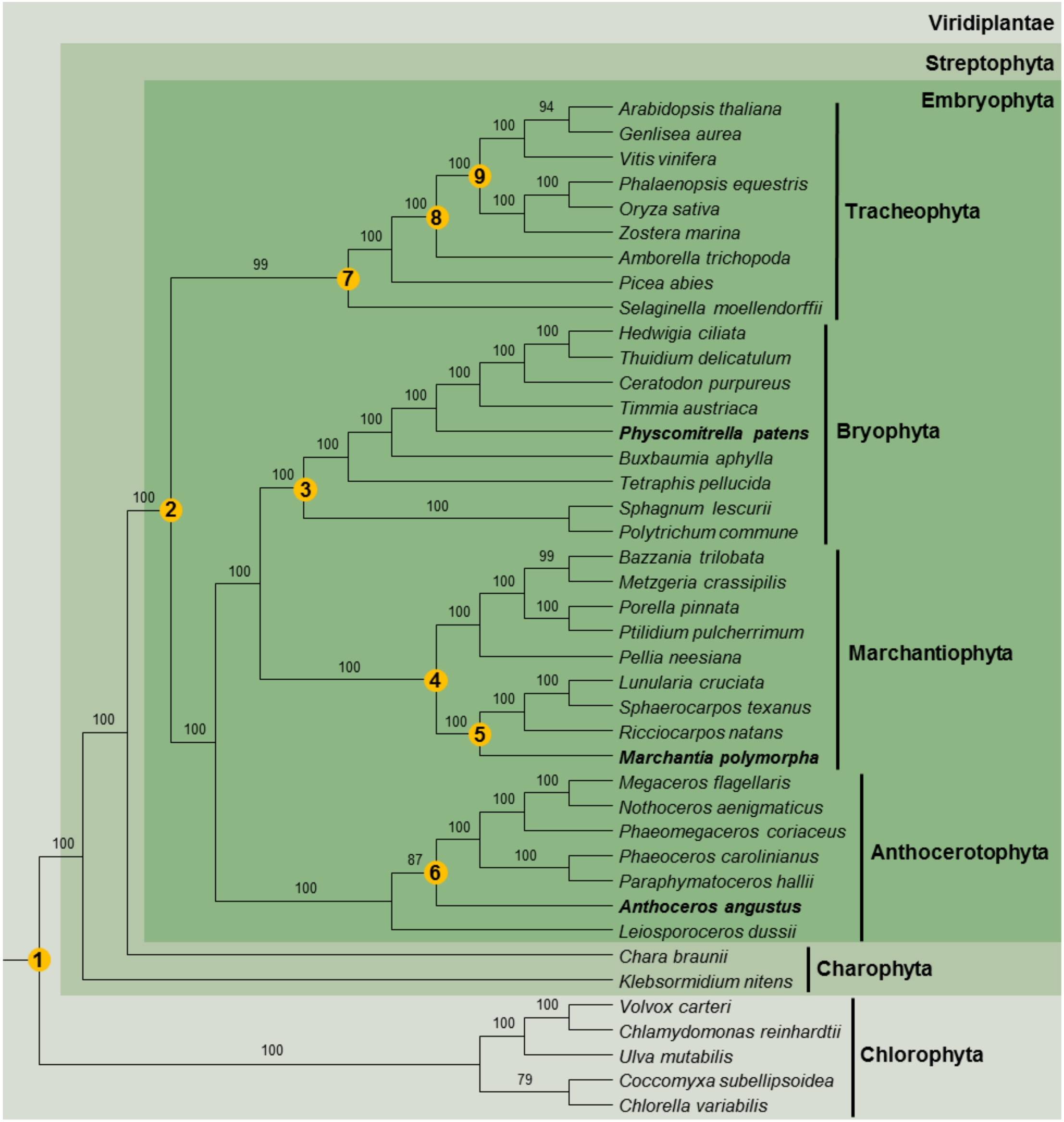

Supplementary Figure 10. Cladogram of maximum likelihood inference from 85 single-copy nuclear genes extracted from genomes of 19 green plants and transcriptomes from additional 22 bryophyte species. The phylogenetic tree is based on the in-frame nucleotide alignment of the first and second-codon positions. The nodes to which fossil calibrations were applied are marked with numbered yellow circles. The three bryophytes with bold fonts have genomic data, and the other 22 bryophytes have transcriptome data from 1KP database. Bootstrap support values ≥ 50% are shown above branches.

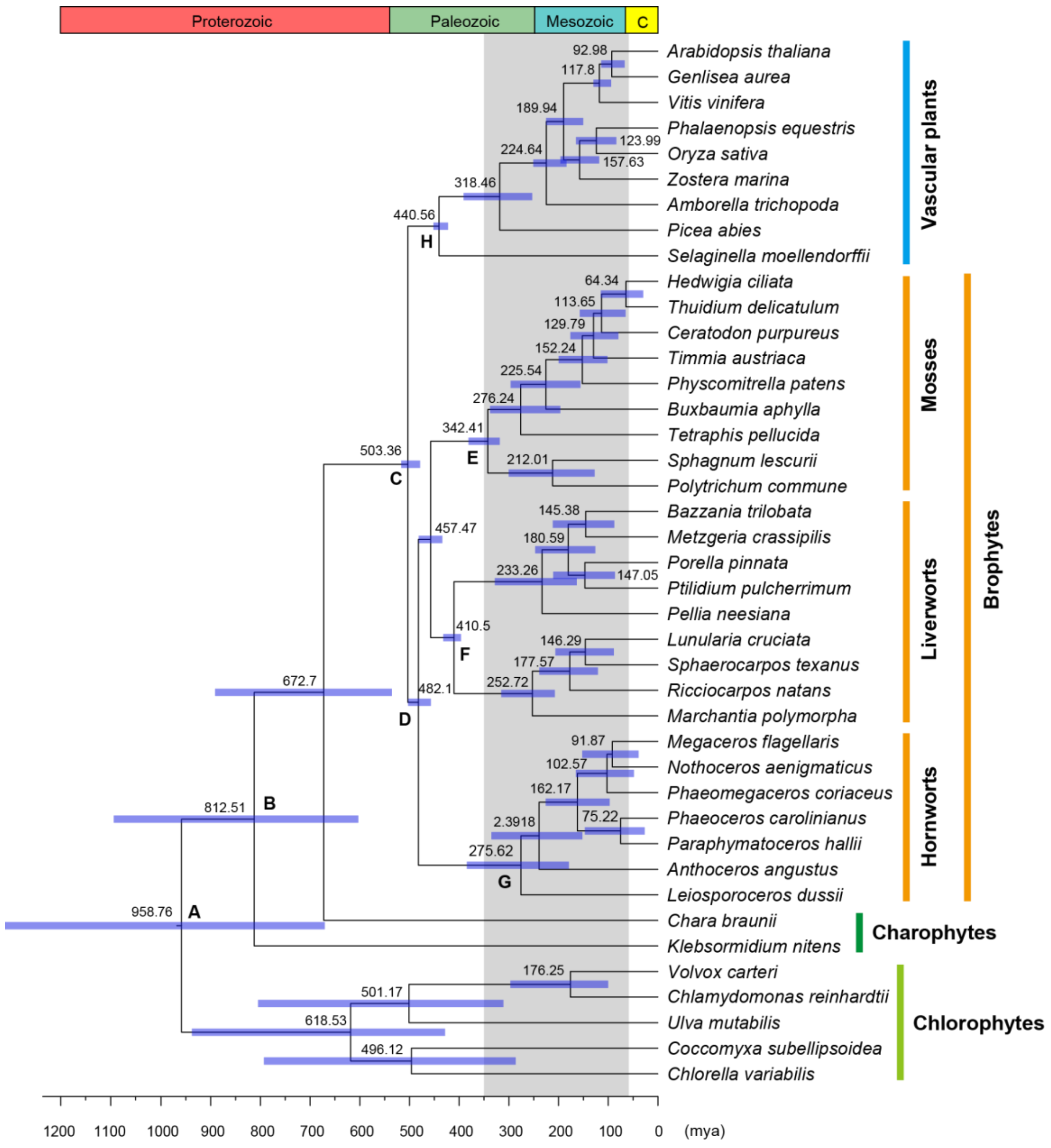

Supplementary Figure 11. Dated chronogram tree for 41 green plants based on relationships inferred from 85 single-copy nuclear genes extracted from genomes of 19 green plants and transcriptomes from additional 22 bryophyte species. Values beside nodes represent the node ages. The blue bars represent 95% highest posterior density (HPD) interval of the node ages. The 95% HPD intervals of age estimates for nodes A-H are indicated in **Supplementary Table 19**. The shade indicates the major diversification times for bryophytes.

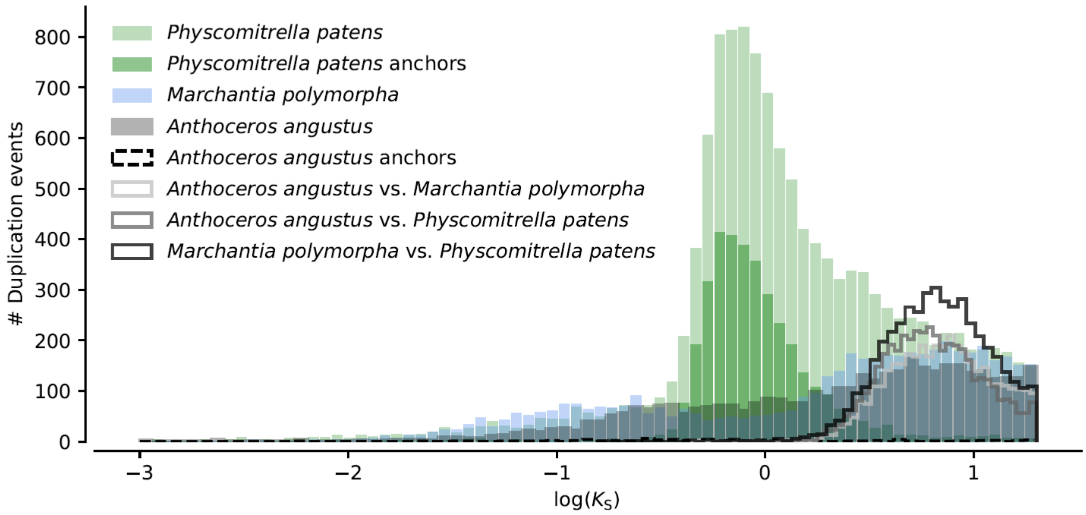

Supplementary Figure 12. Comparison of whole paranome, anchor pair and one-to-one ortholog K_S distributions across the three bryophyte species (*Physcomitrella patens*, *Marchantia polymorpha* and *Anthoceros angustus*) on a logarithmic scale.

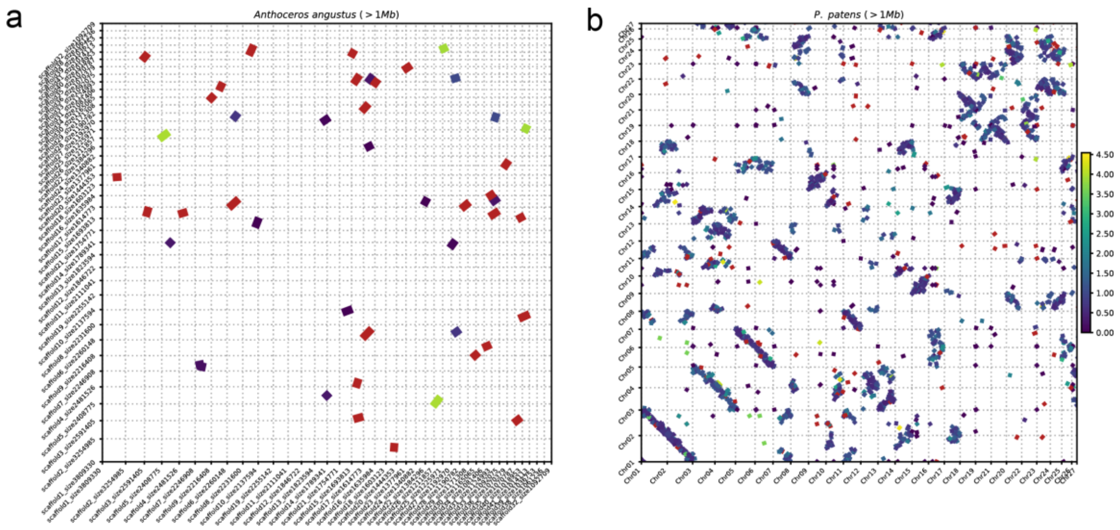

Supplementary Figure 13. Co-linear dot plots for *Anthoceros angustus* and *Physcomitrella patens*. Co-linear dot plots with segments colored by median K_S value of the constituent anchor pairs. Segments are marked in red when the median K_S value exceeded 5. **a**, Co-linear dot plot for *A. angustus*. **b**, For the sake of comparison, a co-linear dot plot for *P. patens*, which has undergone at least one WGD event (Rensing et al., 2008).

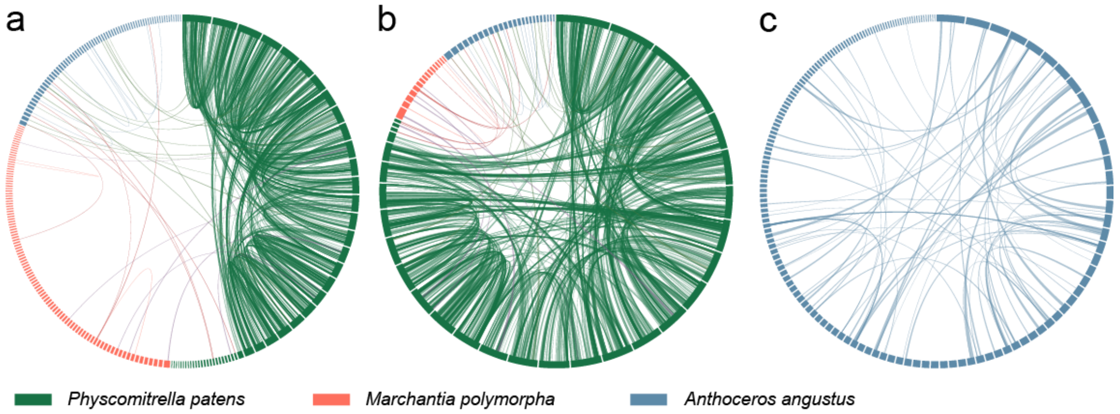

Supplementary Figure 14. Circular representation of co-linear regions identified across the three bryophyte genomes. a, Collinearity between scaffolds/chromosomes larger than 500 kb in length. **b**, The same co-linear regions as in **a**, but excluding scaffolds that did not have any co-linear region. **c**, Co-linear regions on scaffolds larger than 50 kb identified based on the whole paranome of *A. angustus*.

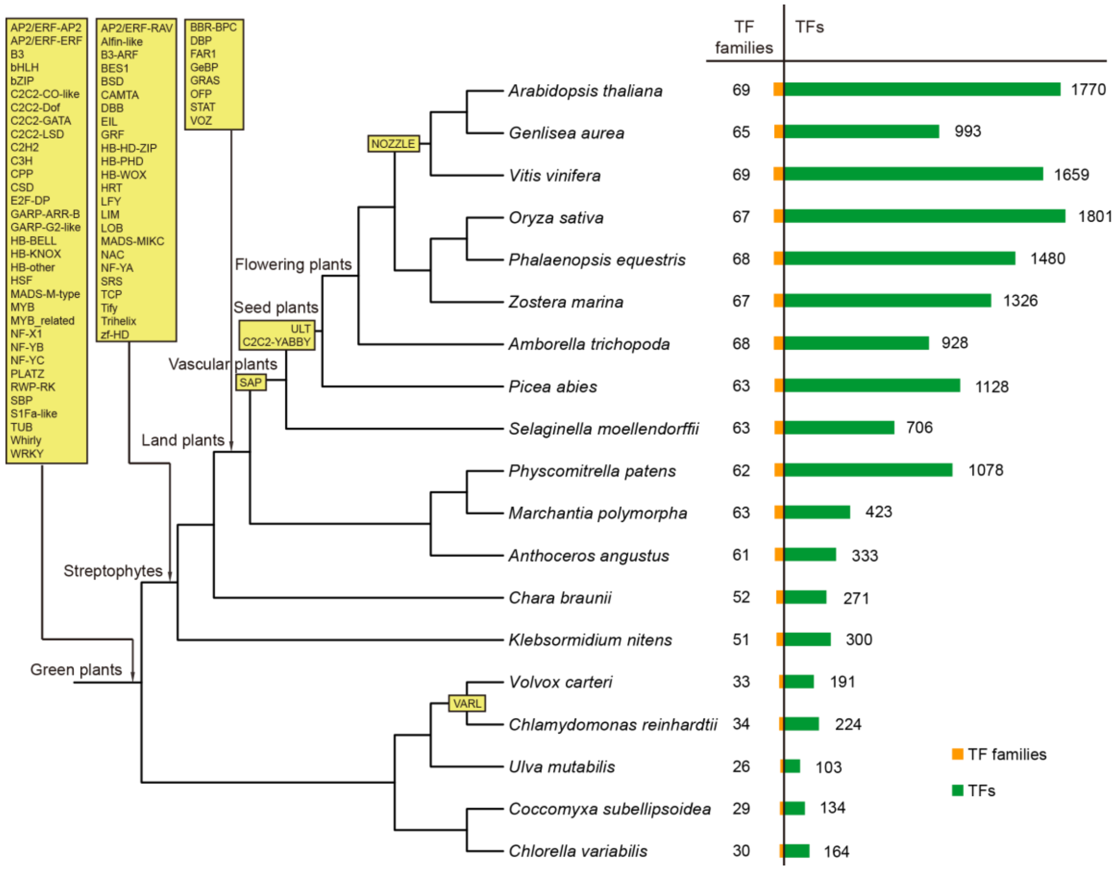

Supplementary Figure 15. Evolution and diversity of transcription factors identified by iTAK in *Anthoceros angustus* and other 18 green plants. The numbers of transcription factors (TFs) (green) and TF gene families (orange) are shown. The origin of TF gene families during the diversification of land plants is estimated by ancestral state reconstruction and labeled above the branch.

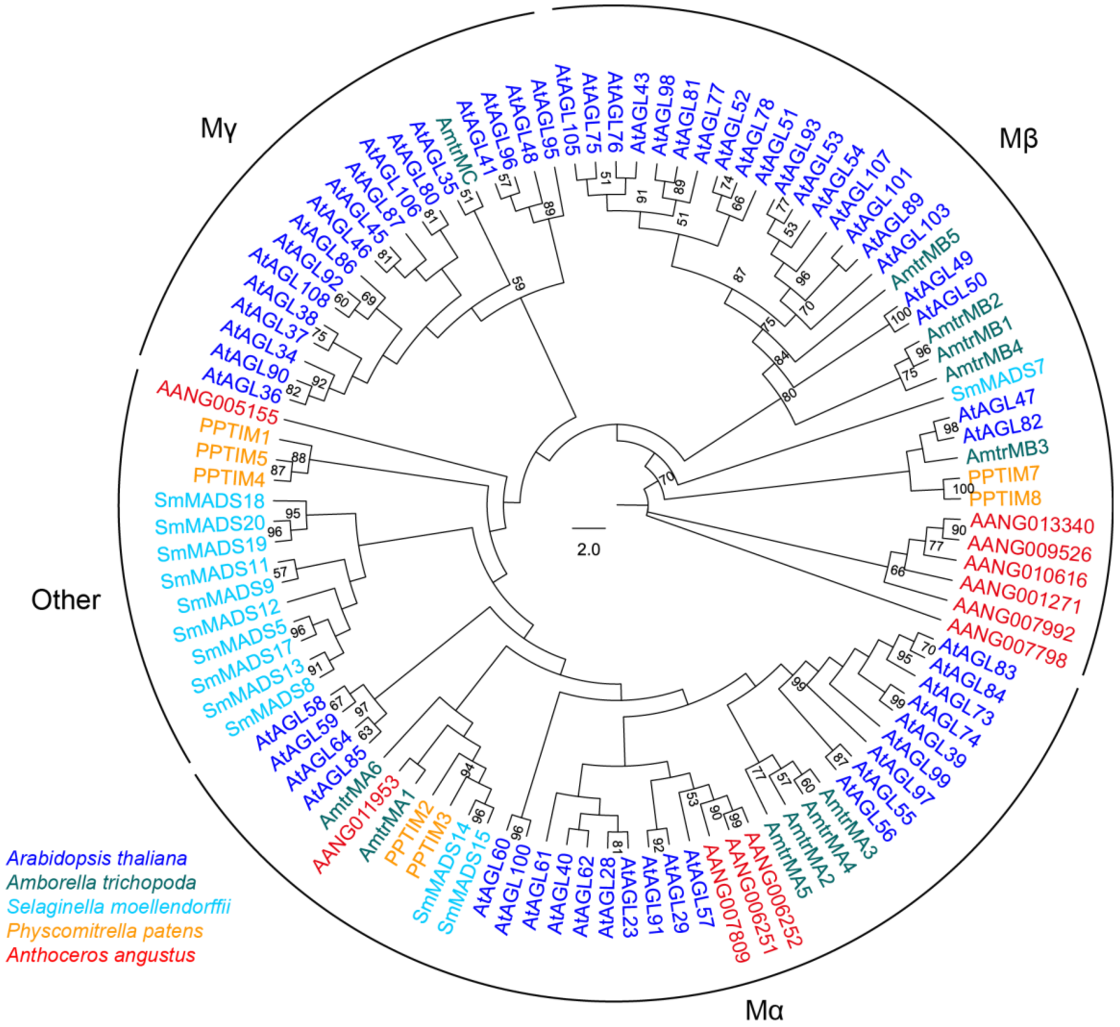

Supplementary Figure 16. Phylogeny of Type I MADS-box proteins from land plants. Tree was constructed using an amino acid alignment of MADS domain (PFAM profile PF00319) sequences. Bootstrap support values ≥ 50% are shown above the branches. No type I genes were identified in the liverwort *Marchantia polymorpha* and the charophyte green algae *Klebsormidium nitens* and *Chara braunii* genomes. The *Anthoceros angustus* genome comprises 11 putative type I genes (**Supplementary Table 22**), of which four were grouped in the Mα sub-clade, six in the Mβ sub-clade, and one in the unnamed sub-clade only with members from the lycophyte and moss. No members of the Mγ sub-clade were uncovered in the *A. angustus*, *Selaginella moellendorffii* or *Physcomitrella patens* genomes, implying that the Mγ sub-clade might have originated after the divergence of lycophytes from other vascular plants.

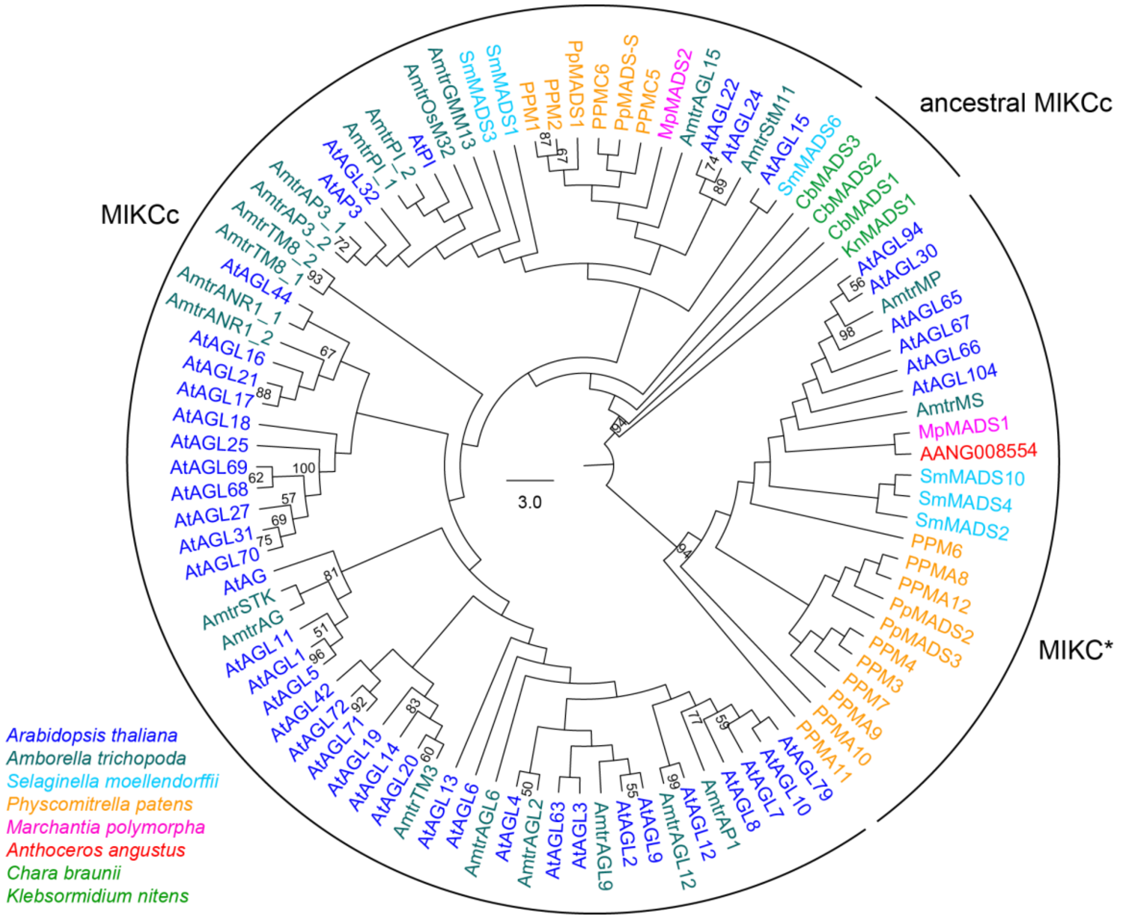

Supplementary Figure 17. Phylogeny of Type II MADS-box proteins from land plants and green algae. Tree was constructed using an amino acid alignment of MADS domain (PFAM profile PF00319) sequences. Bootstrap support values ≥ 50% are shown above the branches. Three and one type II genes were found in the genome of the charophyte green algae *Klebsormidium nitens* and *Chara braunii*, belonging to the ancestral MIKC^{C} gene that gave rise to MIKC^{C} and MIKC* type genes of land plants. Only one MIKC* type II gene (AANG008554) and no MIKC^{C} type II genes were found in the *Anthoceros angustus* genome (**Supplementary Table 22**).

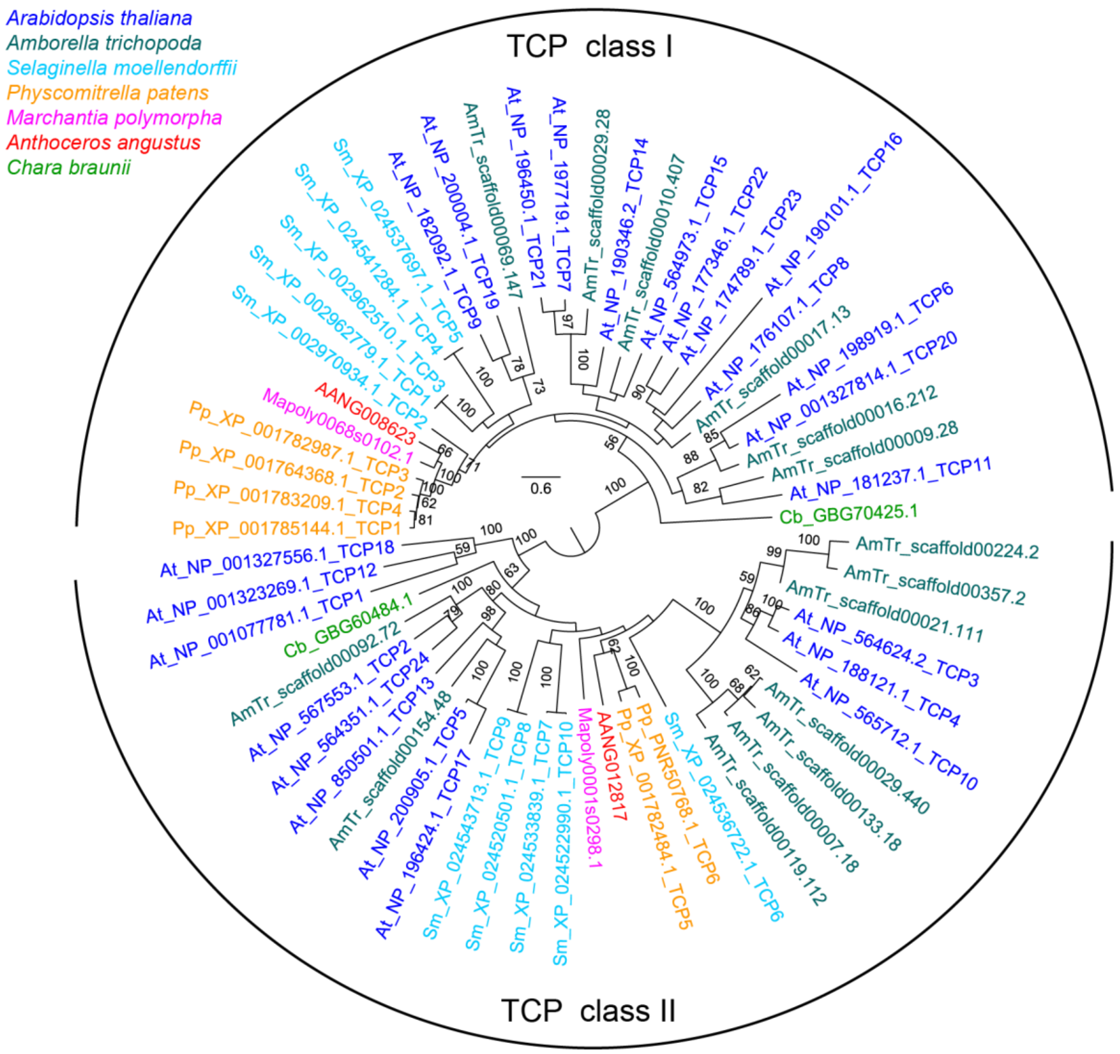

Supplementary Figure 18. Phylogeny of TCP proteins from land plants and green algae. Bootstrap support values ≥ 50% are shown above the branches. No *TCP* genes were found in the chlorophyte green algal genomes. The two *TCP* genes identified in the *Anthoceros angustus* genome belong to class I (AANG008623) and class II (AANG012817) clades, respectively, a pattern identical to that observed for the liverwort *Marchantia polymorpha* and charophyte green alga *Chara braunii*, suggesting that the ancestral land plant had two *TCP* genes and these genes predated the emergence of land plants.

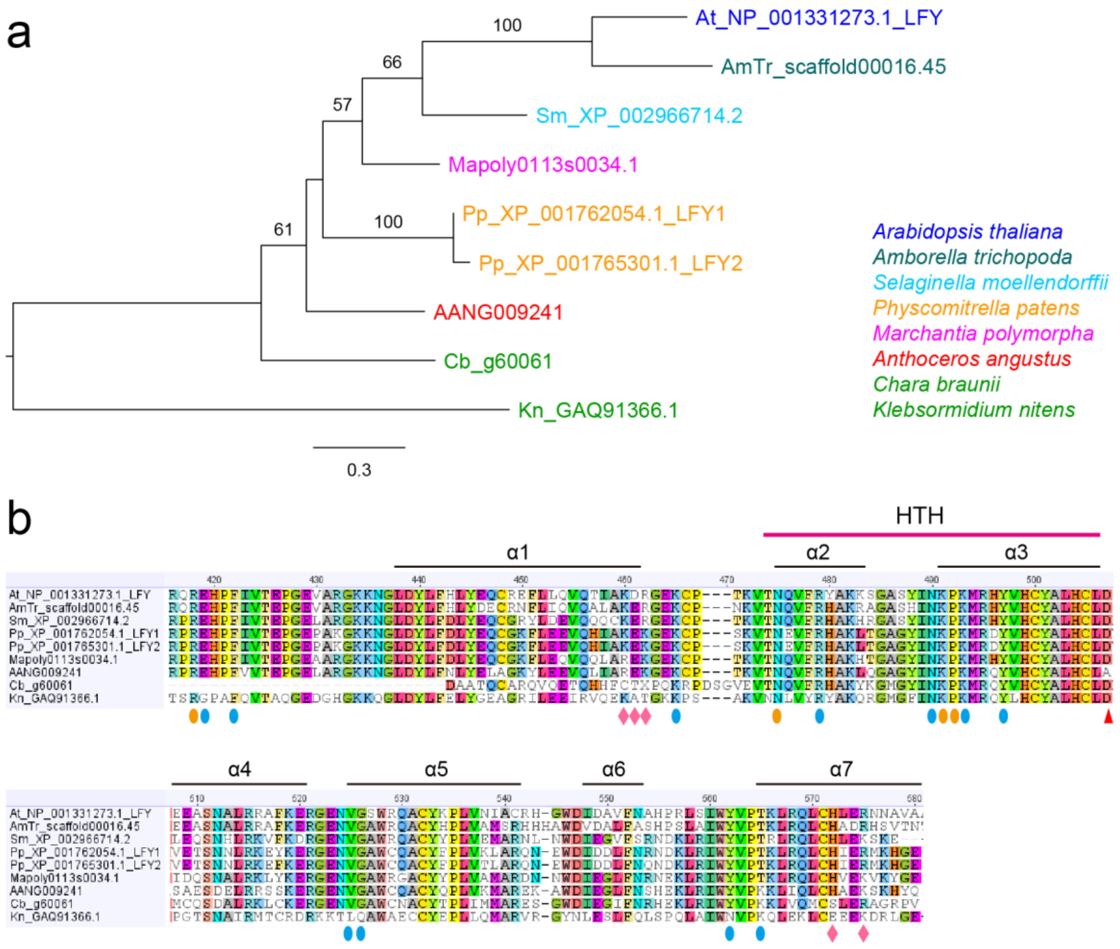

Supplementary Figure 19. Relationships and charcateristics of LFY proteins of land plants and green algae. a, Phylogeny of LFY proteins rooted on the *Klebsormidium nitens* sequence. Bootstrap support values ≥ 50% are shown above the branches. No *LFY* genes were found in the chlorophyte green algal genomes. In *Anthoceros angustus*, the *LFY* gene family comprises a single-copy gene (AANG009241). **b**, The alignment of the DBD domain sequences for DNA-binding in LFY proteins. The secondary structure annotation (α, alpha helices; HTH, helix-turn-helix domain) is indicated above the alignment. Orange dots indicate contacts with DNA bases; blue dots show contacts with phosphate backbone; pink diamonds present residues involved in DBD dimerization; red triangles indicate specific residues in the *A. angustus* LFY protein. Most of residues for DNA contacts of the *A. angustus* LFY protein are highly conserved with those of other land plants and only several residues exhibited to be *A. angustus*-specific.

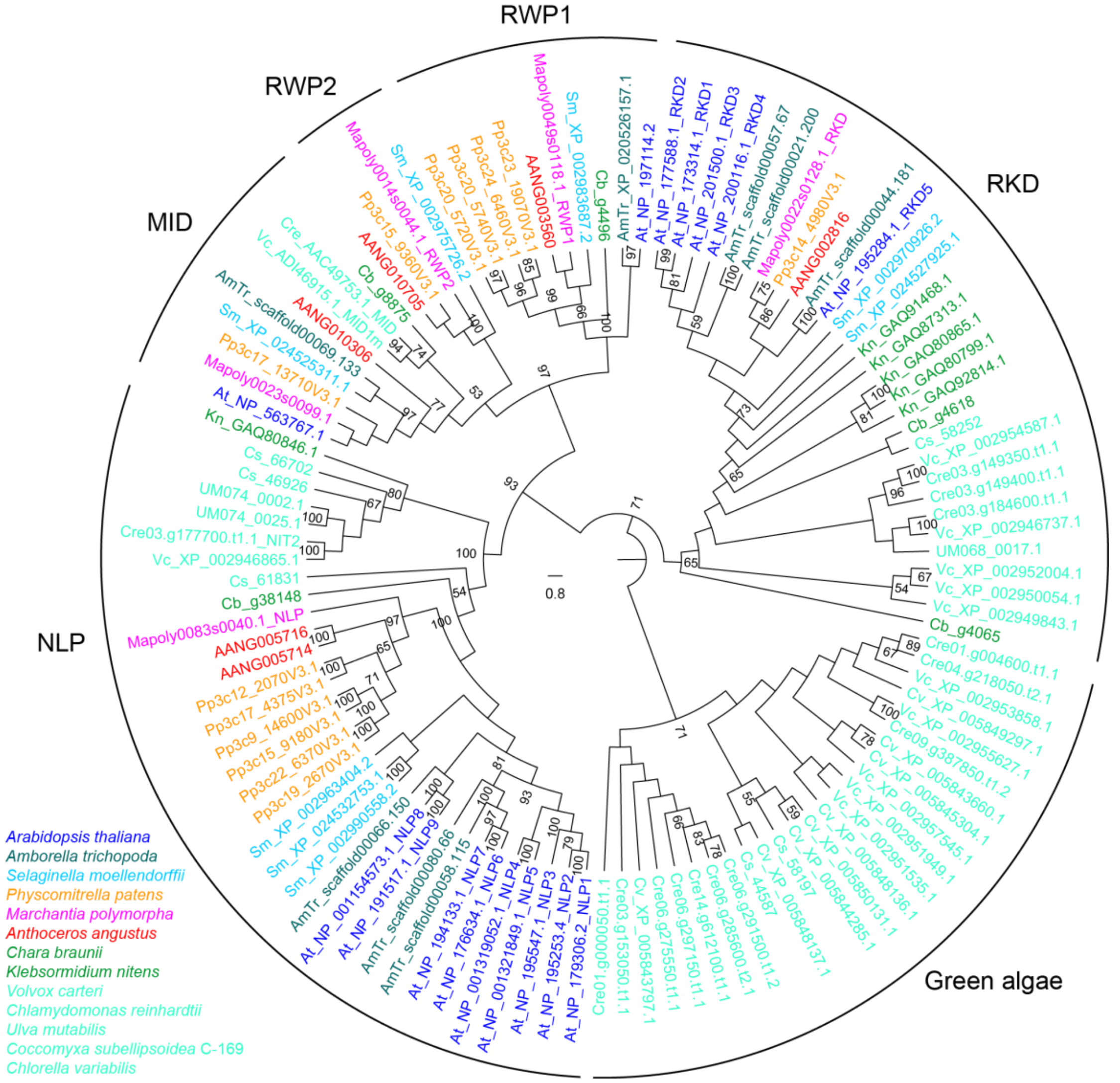

Supplementary Figure 20. Phylogeny of RWP-RK proteins from land plants and green algae. Bootstrap support values ≥ 50% are shown above the branches. The RWP-RK proteins compose six clades (i.e., RKD, NLP, MID, RWP1, RWP2, and green algae-specific clade), and except for green algae-specific clade, all clades are highly supported. The *Anthoceros angustus* genome comprises one *RKD* gene, two *NLP* genes, one *MID* gene, one *RWP1* gene, and one *RWP2* gene.

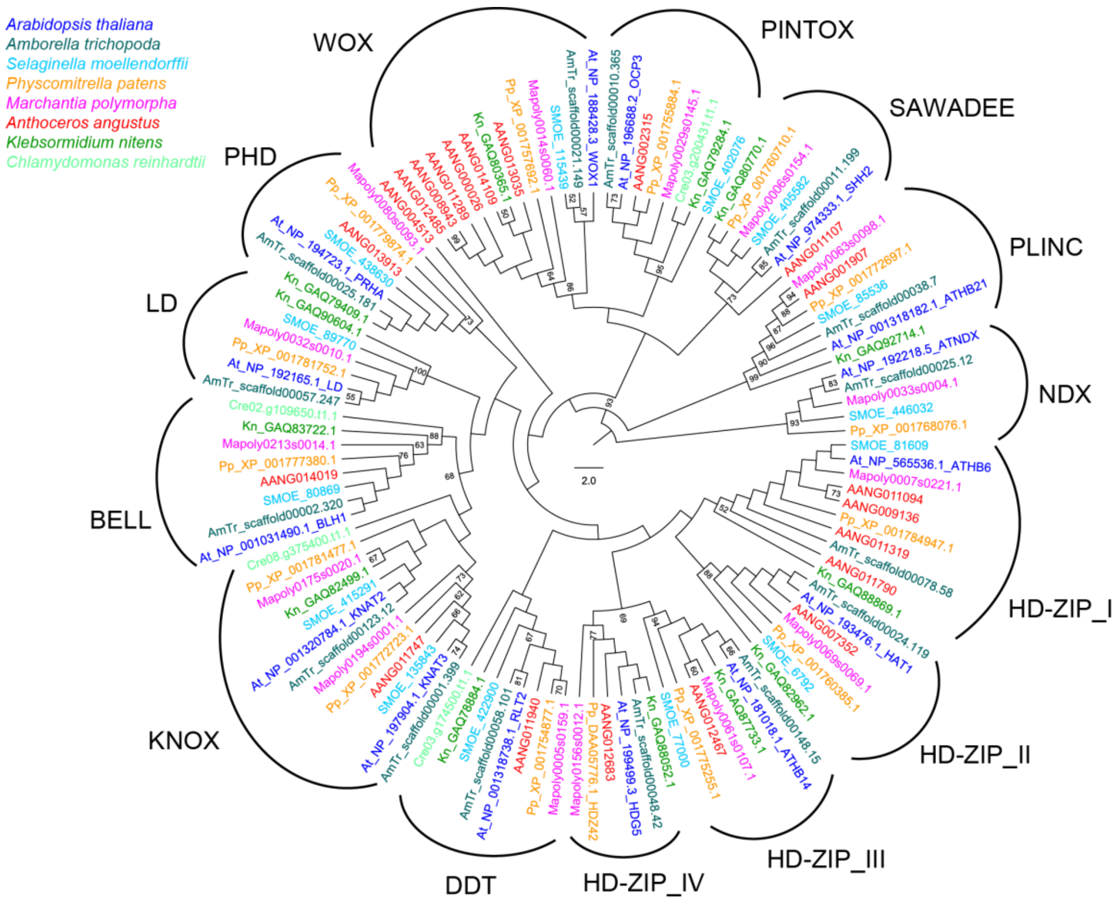

Supplementary Figure 21. Phylogeny of homeodomain proteins from land plants and algae. The classification of homeodomain proteins of *Anthoceros angustus* is based on the phylogenetic analysis of an amino acid alignment of homeodomain (HD) (PFAM profile PF00046) sequences of HD proteins using one or two representative homeodomain HD proteins for each HD class from *A. angustus* and other green plant HD proteins. Bootstrap support values ≥ 50% are shown above the branches. The 20 *A. angustus* HD proteins are distribued among 12 of the 14 HD classes, except for one (AANG004513) not grouping with any classes. In *A. angustus*, *WOX* and class I *HD-ZIP homeobox* genes occurred in multiple paralogs, whereas other class homeobox genes were represented by a single copy.

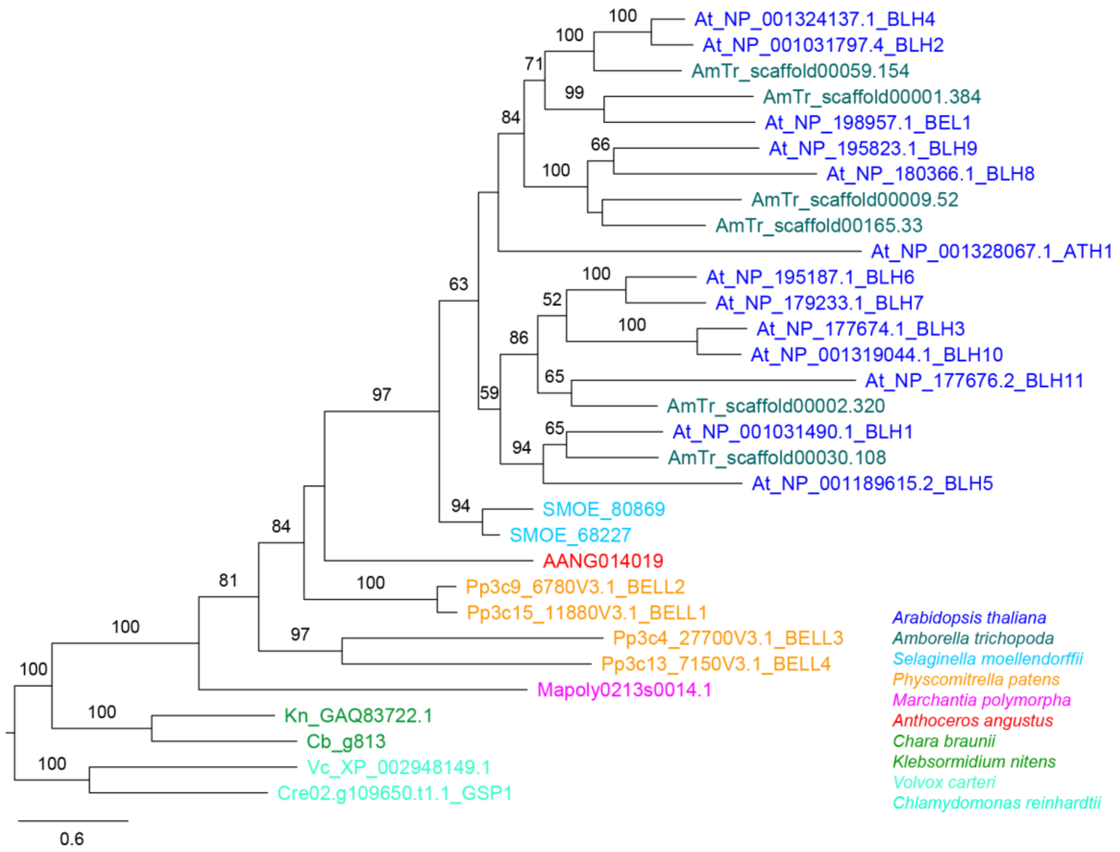

Supplementary Figure 22. Phylogeny of BELL class homeodomain proteins from land plants and green algae. The ML tree is rooted on sequences of the chlorophyte green algae. Bootstrap support values ≥ 50% are shown above the branches. Multiple copies of *BELL* genes occur in angiosperms, but only one copy (AANG014019) in the genomes of *Anthoceros angustus* and *Marchantia polymorpha*.

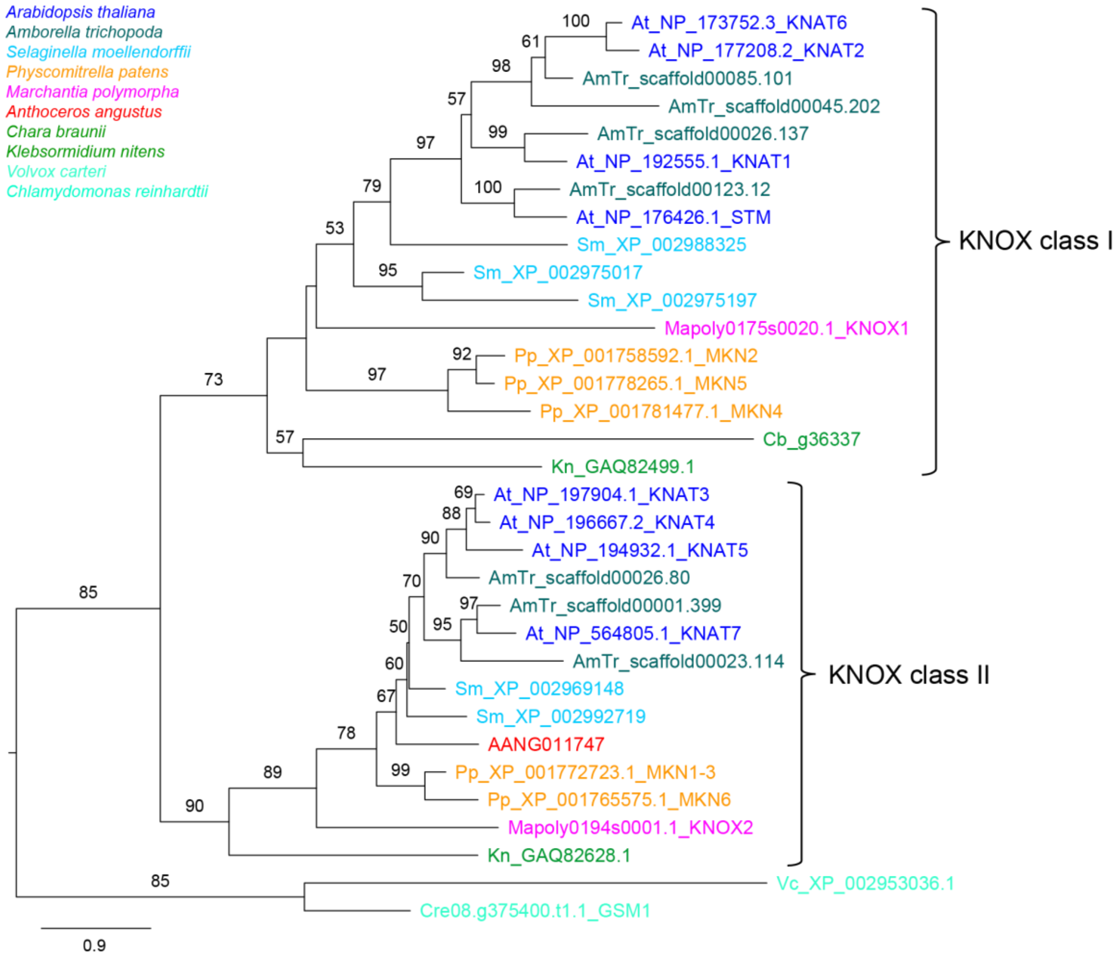

Supplementary Figure 23. Phylogeny of KNOX class homeodomain proteins from land plants and green algae. The ML tree shown is rooted on sequences of the chlorophyte green algae. Bootstrap support values ≥ 50% are shown above the branches. A single *KNOX* gene (AANG011747), belonging to the class II *KNOX* subgroup, occurs in the *Anthoceros angustus* genome. No class I *KNOX* genes were found in *A. angustus*.

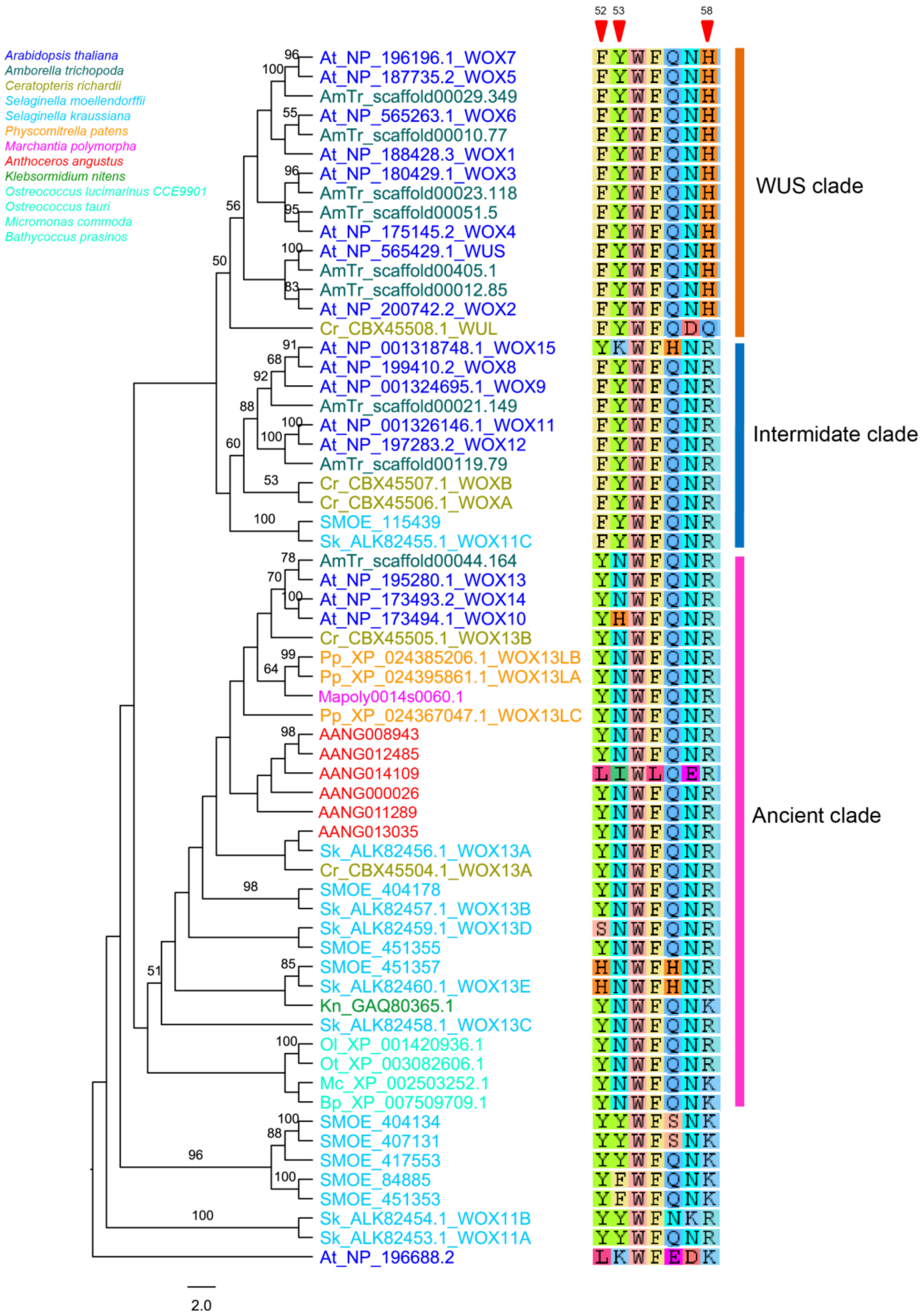

Supplementary Figure 24. Phylogeny of WOX class homeodomain proteins from land plants and green algae. The NP_196688.2 (OCP3) sequence of *Arabidopsis thaliana* was used as outgroup. Bootstrap support values ≥ 50% are shown above the branches. The homeodomain residues 52, 53 and 58 were indicated by red arrows for different clade classification in the alignment. The phylogenetic tree of WOX proteins was divided into three clades, WUS, WOX9 and WOX13. There are six *WOX* genes found in the *Anthoceros angustus* genome. The *WOX*

genes from three bryophytes (*A. angustus*, *Marchantia polymorpha*, and *Physcomitrella patens*) and five green algae (*Klebsormidium nitens*, *Ostreococcus lucimarinus* CCE9901, *Ostreococcus tauri*, *Micromonas commoda*, and *Bathycoccus prasinos*) are all grouped in the *WOX13* clade. With the exception of AANG014109 with peptide variation, all the other *Anthoceros* WOX proteins shared the WOX13-specific peptides (YNWFQNR) in their homeodomains. Our analysis implied that the members of the *WOX13* clade might have originated in the early stages of green plant evolution, while *WUS* and *WOX9* clades might have evolved in vascular plants.

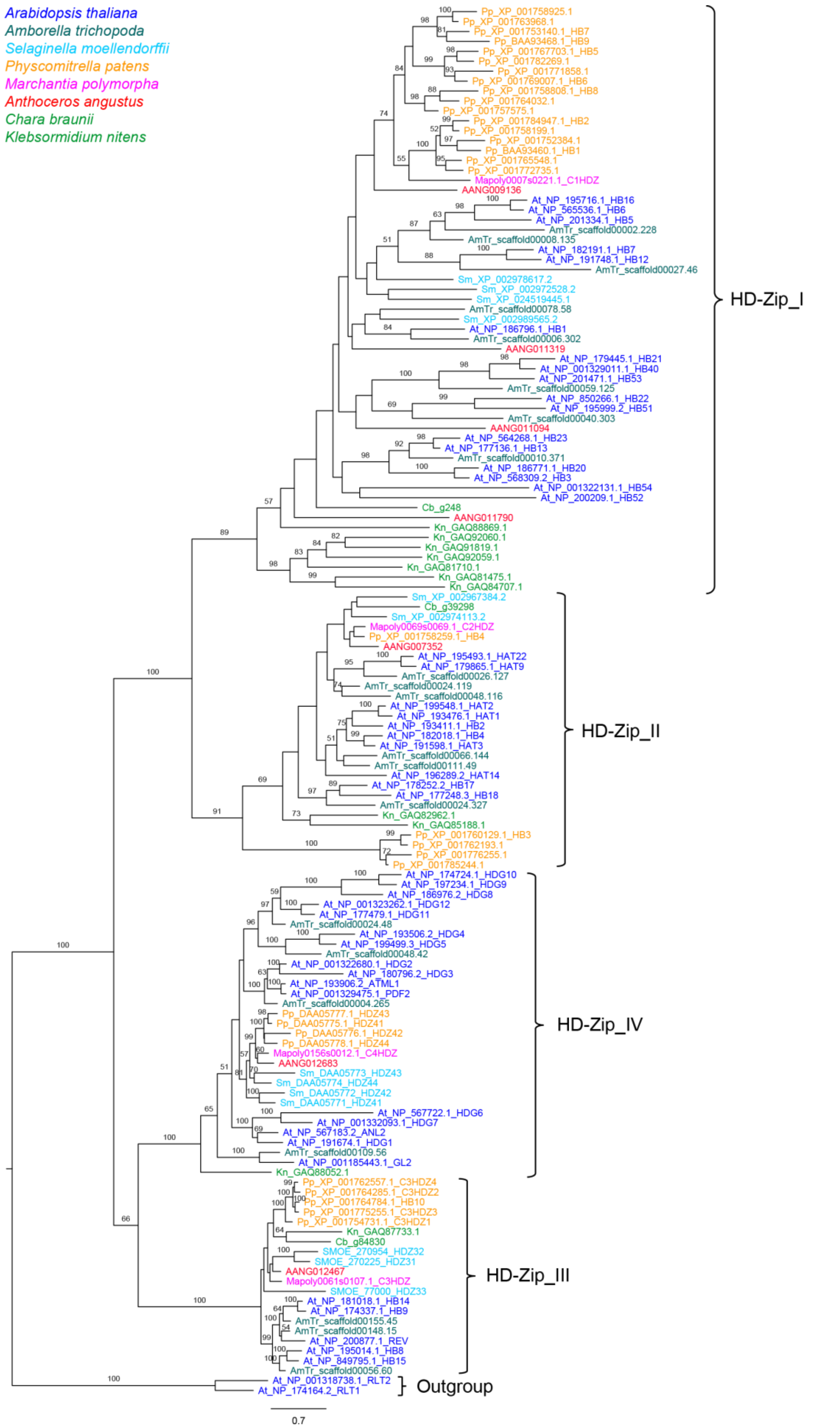

Supplementary Figure 25. Phylogeny of HD-Zip class homeodomain proteins from land

plants and green algae. The NP_174164.2_RLT1 and NP_001318738.1_RTL2 sequences of *Arabidopsis thaliana* were used as outgroup. Bootstrap support values ≥ 50% are shown above the branches. The seven *Anthoceros angustus* HD-Zip proteins were resolved among four HD-Zip classes, a single copy for class II to IV HD-Zips, respectively and four paralogs for class I HD-Zip. By comparison, the liverwort *Marchantia polymorpha* holds a single copy for each HD-Zip class, and the moss *Physcomitrella patens* has multiple copies for each HD-Zip class. This result is consistent with the presence of whole genome duplication in *P. patens* and the absence of whole-genome duplication in *A. angustus* and *M. polymorpha* (**Fig. 2c**). The multiple copies for class I HD-Zip genes in *A. angustus* might result from small-scale gene duplication.

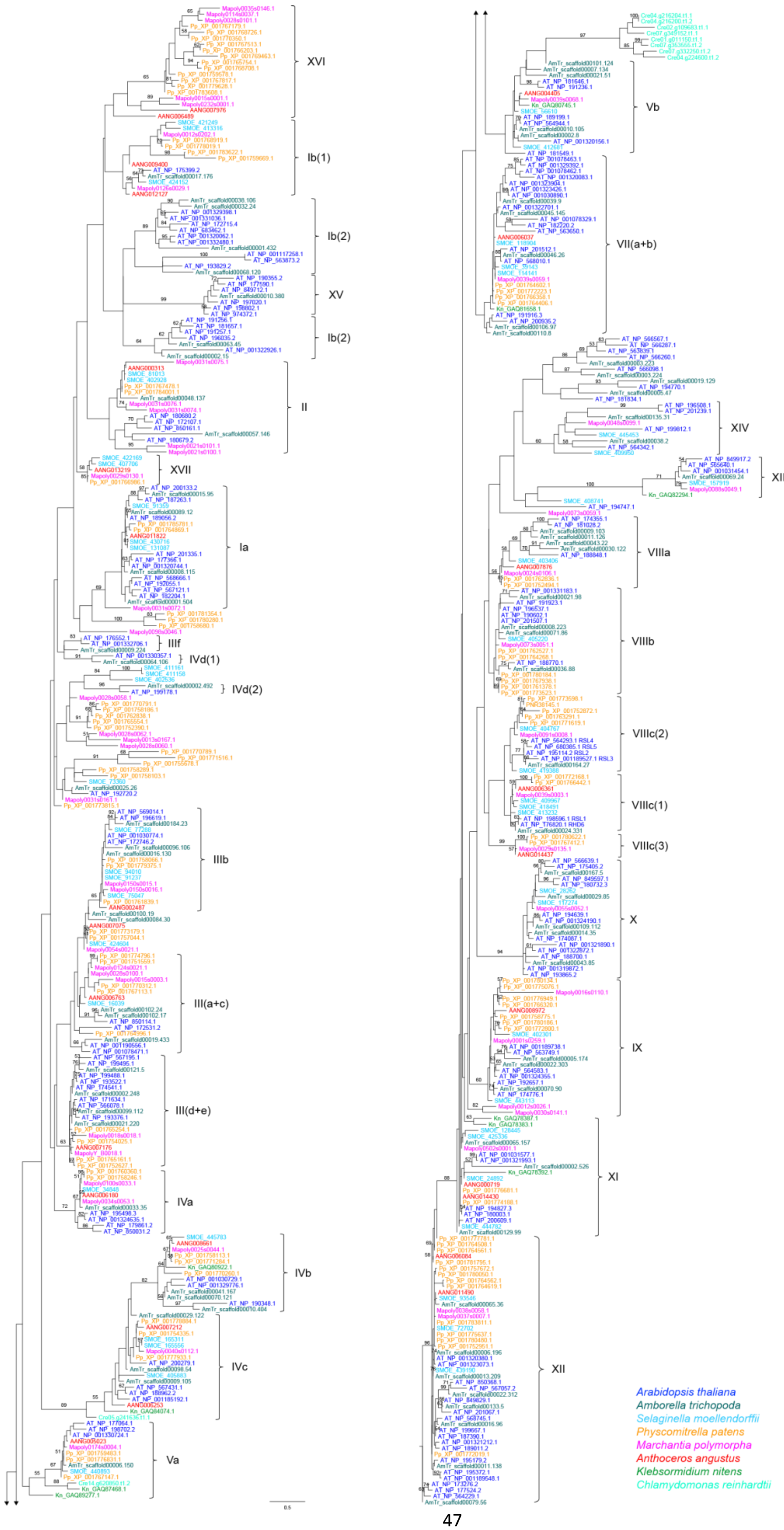
XVI
Ib(1)
Ib(2)
XV
Ib(2)
II
XVII
Ia
IIIf
IVd(1)
IVd(2)
IIIb
III(a+c)
III(d+e)
IVa
IVb
IVc
Va
Vb
VII(a+b)
XIV
XIII
VIIIa
VIIIb
VIIIc(2)
VIIIc(1)
VIIIc(3)
X
IX
XI
XII
Arabidopsis thaliana
Amborella trichopoda
Selaginella moellendorffii
Physcomitrella patens
Marchantia polymorpha
Anthoceros angustus
Klebsormidium nitens
Chlamydomonas reinhardtii
0.5

Supplementary Figure 26. Phylogeny of bHLH proteins from land plants and green algae. Tree was constructed using the alignment of basic helix-loop-helix domain sequences. Bootstrap support values ≥ 50% are shown above the branches. Phylogenetic analysis identified 30 subfamilies within the plant *bHLH* transcription factor family. Among these, two subfamilies (Va and IVc) include land plants and green algae proteins and may thus have occurred in the common ancestors of green plants; five (XI, XIII, VII(a+b), Vb, and IVb) include land plants and charophyte green algae and may have evolved in ancestral streptophytes; 14 are restricted to land plants and their evolution may date back to the colonization of land; one (XVII) is restricted to non-seed plants implying its loss of in seed plants; two (VIIIc(3) and XVI) are bryophytes-specific that were either lost before the origin of vascular plants or evolved in the ancestor of a monophyletic bryophyte clade. A total of 26 bHLH proteins were identified in the *Anthoceros angustus* genome, which covered 20 *bHLH* subfamilies.

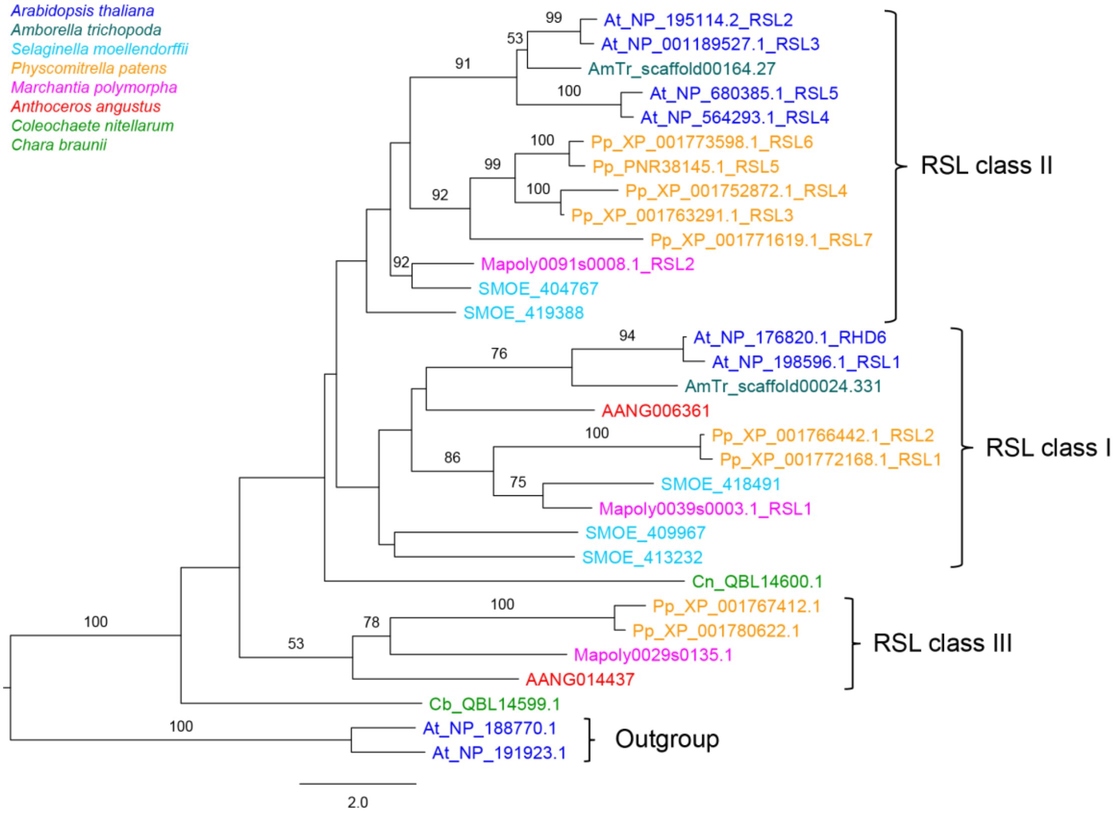

Supplementary Figure 27. Phylogeny of bHLH-VIIIc proteins (RSL) from land plants and green algae. Two bHLH-VIIIb protein sequences from *Arabidopsis thaliana* (NP_188770.1 and NP_191923.1) were used as outgroup. Bootstrap support values ≥ 50% are shown above the branches. No bHLH VIIIc members were found in chlorophyte green algae genomes. A single copy of bHLH VIIIc gene is present in both charophyte green algae, i.e., *Chara braunii* (QBL14599.1) that develops rhizoids and *Coleochaete nitellarum* (QBL14600.1) that does not develop rhizoids. However, the sequences are highly divergent from those of land plants. The phylogenetic tree of bHLH proteins revealed that land plant bHLH-VIIIc proteins are distributed among three classes, VIIIc(1), VIIIc(2), and VIIIc(3) (**Supplementary Fig. 25**). The clade VIIIc(1) refers to class I RSL and VIIIc(2) refers to class II RSL, while VIII(3) is newly named as class III RSL. Only one member of *RSL* class I bHLH gene (AANG006361) and no class II *RSL* gene occurs in the *Anthoceros angustus* genome. The class III RSL clade is bryophyte-specific, representing the ancestral *RSL* genes.

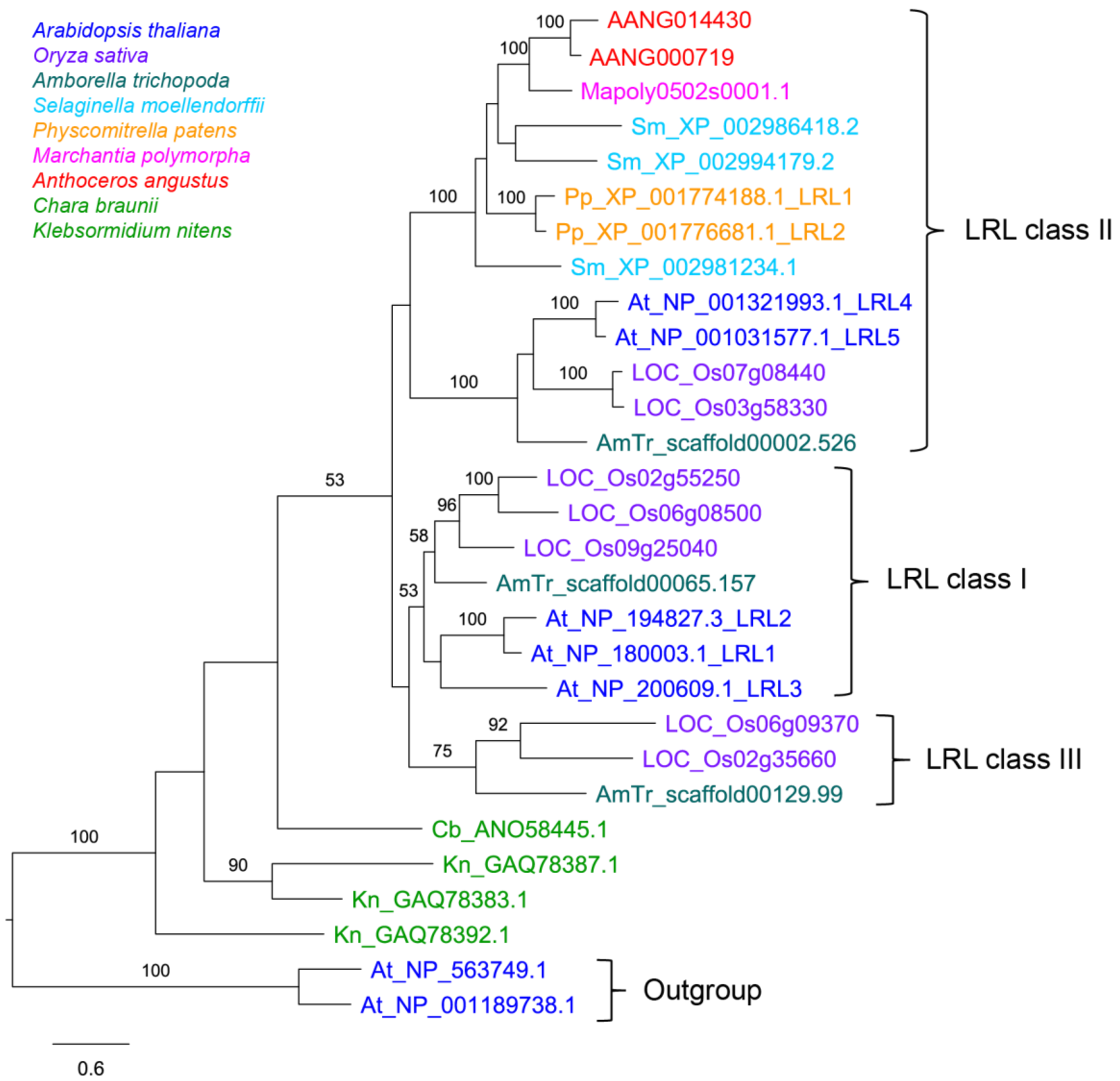

Supplementary Figure 28. Phylogeny of bHLH-XI proteins (LRL) from land plants and green algae. Two bHLH-IX protein sequences from *Arabidopsis thaliana* (NP_563749.1 and NP_001189738.1) were used as outgroup. Bootstrap support values ≥ 50% are shown above the branches. The phylogenetic analysis suggested that in flowering land plants, bHLH-XI transcription factors formed three well-supported clades, termed here as class I, II, and III LRLs. Two *LRL* bHLH genes (AANG014430 and AANG000719), both belonging to the class II *LRL* subgroup, occur in the *Anthoceros angustus* genome.

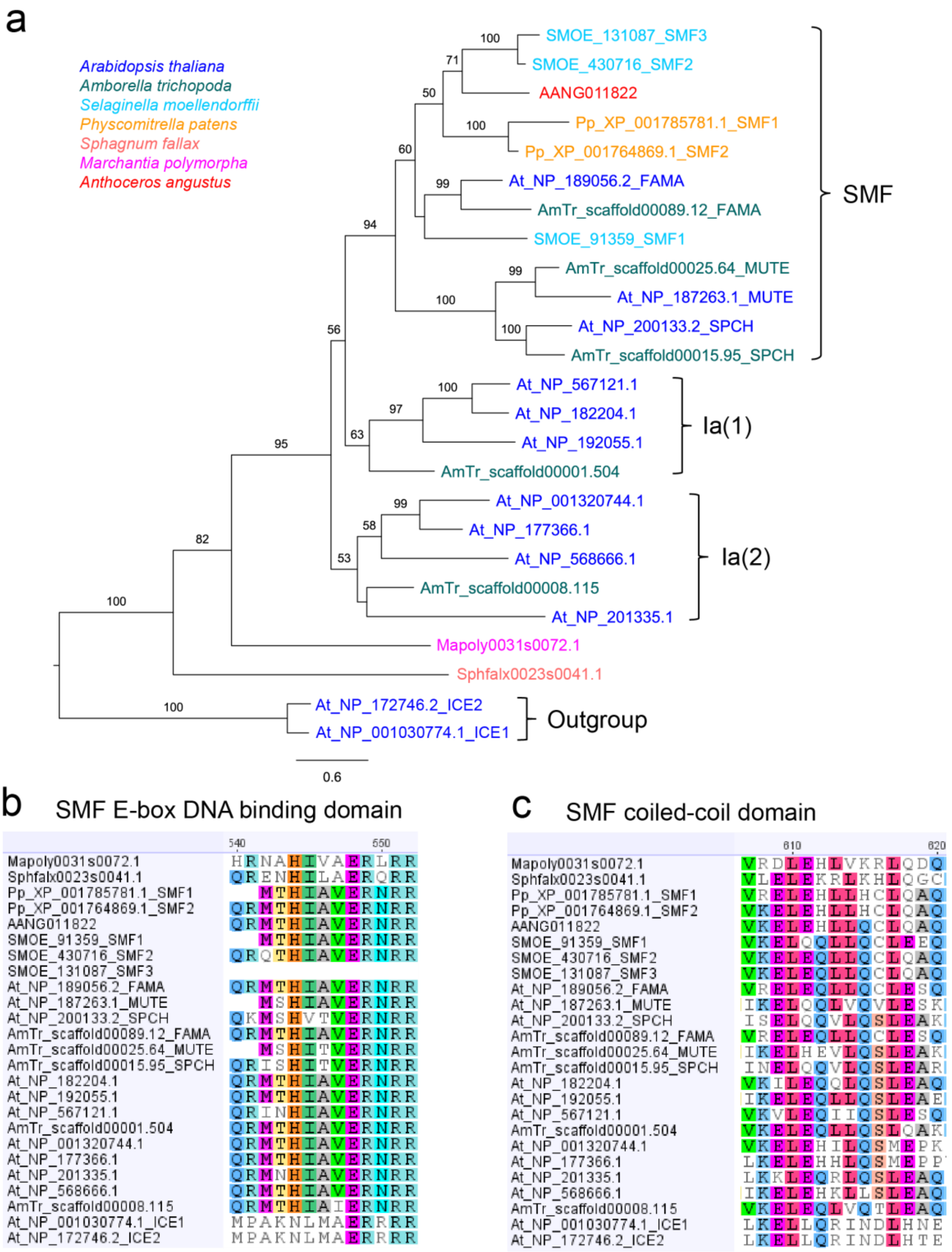

Supplementary Figure 29. Relationships and characteristics of bHLH-Ia proteins from land plants. a, Phylogeny of bHLH-Ia proteins. The SPCH, MUTE, and FAMA-like bHLHs refer to as SMFs. The NP_001030774.1_ICE1 and NP_172746.2_ICE2 sequences of *Arabidopsis thaliana* were used as outgroup. Bootstrap support values ≥ 50% are shown above the branches. The phylogenetic tree revealed three major clades within the bHLH Ia subfamily, SMF, Ia(1), and Ia(2). In the bHLH Ia subfamily, no green algal members were found, implying that this subfamily might have evolved in the common ancestor of land plants. The *SMF* clade includes members from land plants, while Ia(1) and Ia(2) clades are restricted to angiosperms. One *SMF* gene

(AANG011822) is identified in the *Anthoceros angustus* genome. The *SMF* clade was further classified into *FAMA*, *SPCH*, and *MUTE* three subclades. The SMF homologs of non-seed plants with stomata grouped into the FAMA subclade, whereas, putative *SMF* homologs in liverwort *Marchantia polymorpha* lacking stomata and moss *Sphagnum fallax* with pseudostomata do not group into the canonical *SMF* clade. **b**, Sequence alignment of DNA-binding domain sequences of SMF proteins. c, Sequence alignment of coiled-coil domain sequences of SMF proteins. The putative *A. angustus* SMF protein shows conserved key amino acid residues in the bHLH binding domains and coiled-coil domains, while SMF homologs in *M. polymorpha* and *S. fallax* show clear divergence in their bHLH regions.

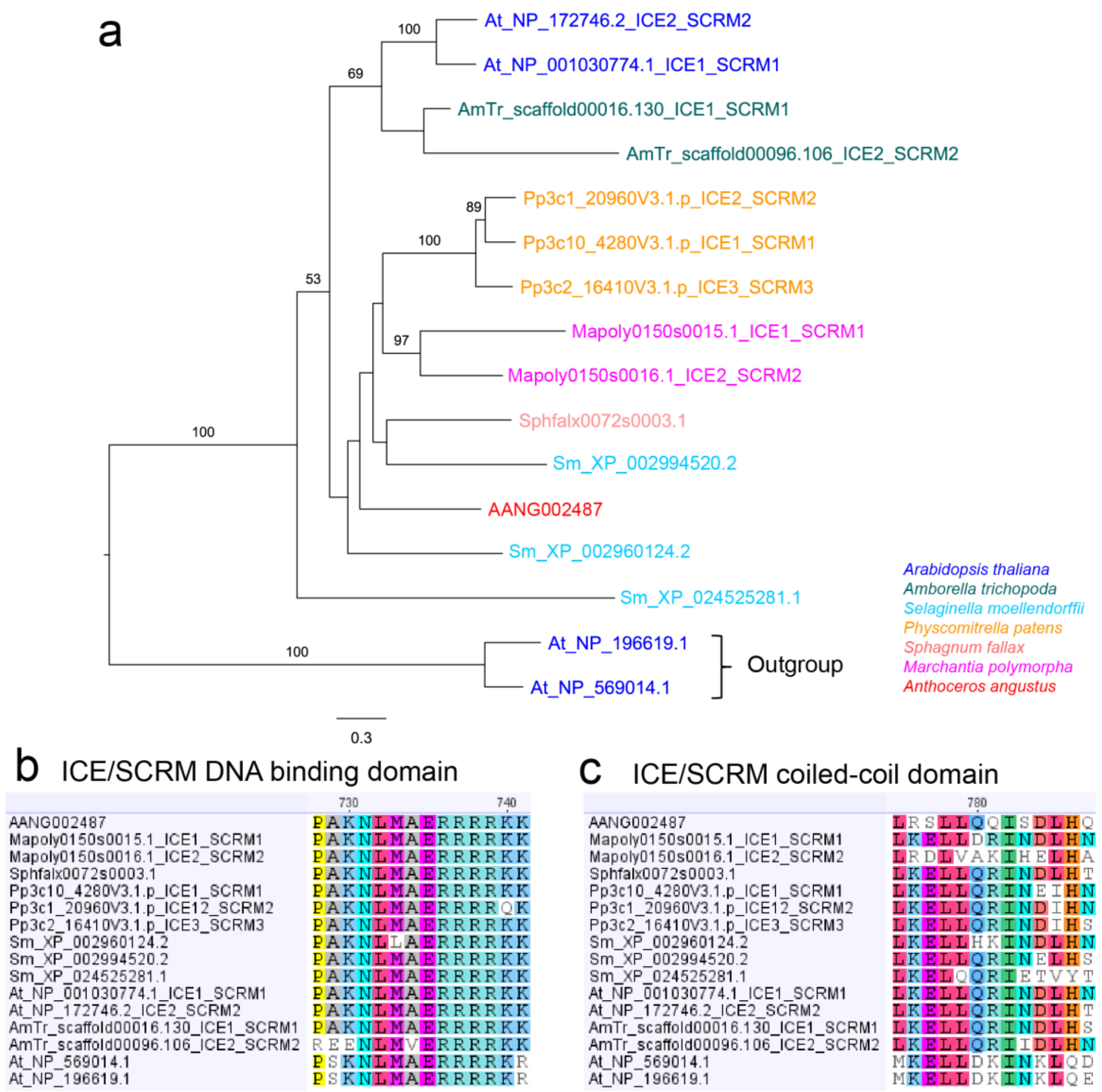

Supplementary Figure 30. Relationships and characteristics of bHLH-IIIb proteins (ICE/SCRM) from land plants. a, Phylogeny of bHLH-IIIb proteins. Two bHLH-IIIb protein sequences from *Arabidopsis thaliana* (NP_196619.1 and NP_569014.1) were used as outgroup. Bootstrap support values ≥ 50% are shown above the branches. One *ICE/SCRM* gene (AANG002487) was identified in the *Anthoceros angustus* genome. **b**, Sequence alignment of DNA-binding domain sequences of ICE/SCRM proteins. **c**, Sequence alignment of coiled-coil domain sequences of ICE/SCRM proteins. The putative *A. angustus* ICE/SCRM protein shares a very high degree of identity in the ICE/SCRM binding domains but divergent residues in the coiled-coil domains with other land plants.

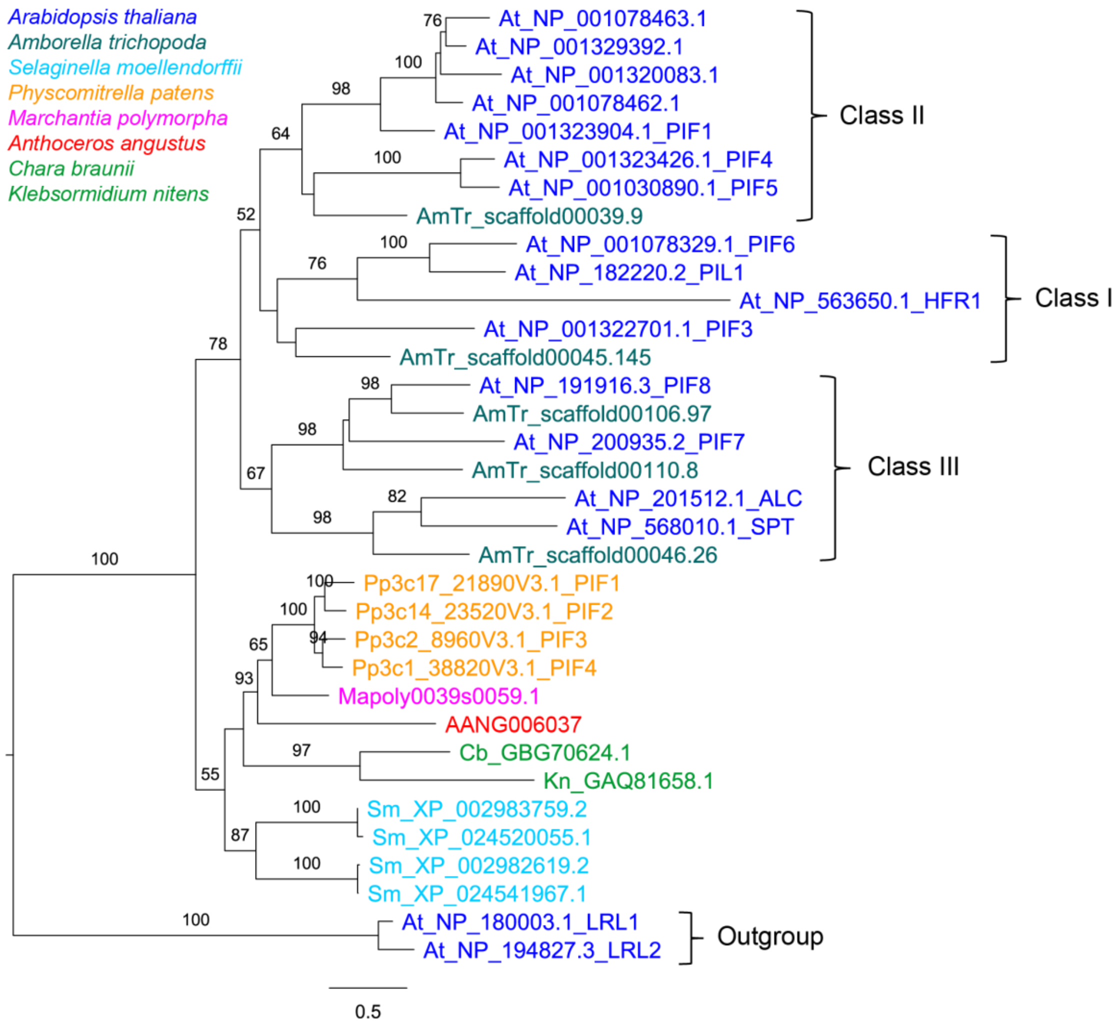

Supplementary Figure 31. Phylogeny of bHLH-VII(a+b) proteins (PIF) from land plants and green algae. The NP_180003.1_LRL1 and NP_194827.3_LRL2 sequences of *Arabidopsis thaliana* were used as outgroup. Bootstrap support values ≥ 50% are shown above the branches. A single bHLH VII(a+b) protein was identified in the charophyte green algae *Chara braunii* and *Klebsormidium nitens*, while no members were found in chlorophyte green algae. The bHLH VII(a+b) subfamily might have arisen in the common ancestor of streptophytes, before the water-to-land transition of plants. Phylogenetic analysis suggested that in flowering plants, bHLH-VII(a+b) transcription factors formed three clades, termed here as class I (PIF1/4/5), II (PIF3/6), and III (PIF7/8). One PIF transcription factor (AANG006037) is identified in the *Anthoceros angustus* genome. It grouped together with PIF protein sequences from moss *Physcomitrella patens* and liverwort *Marchantia polymorpha* with high bootstrap value.

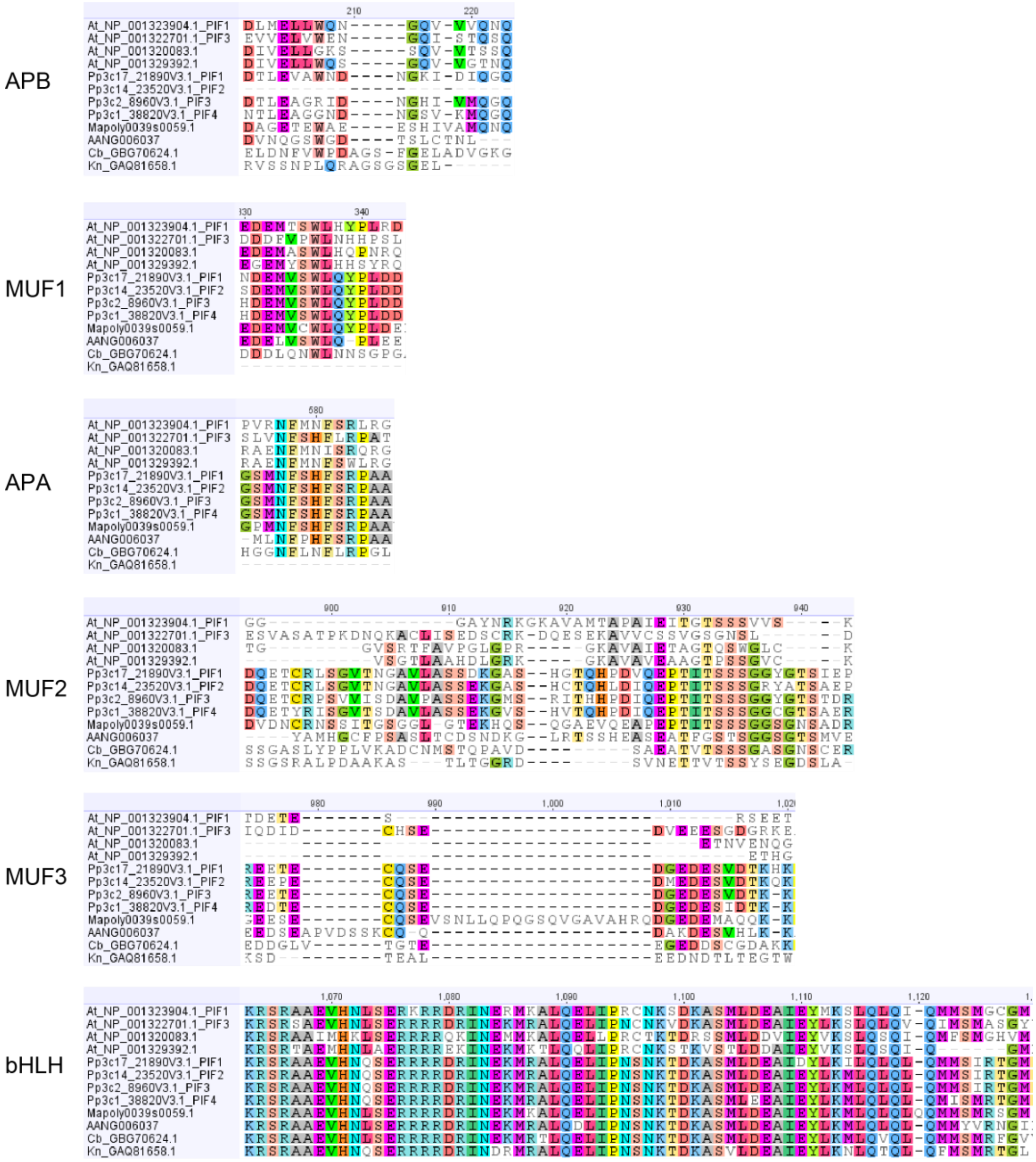

Supplementary Figure 32. Phylogeny of PIF proteins from land plants and green algae. The sequences of APB, MUF1/2/3 and APA motifs, and bHLH domain are indicated in the alignment. The *Anthoceros angustus* PIF protein (AANG006037) harbors a highly conserved bHLH domain at the C-terminus, a conserved Active Phytochrome A binding (APA) motif, and three putative MUF motifs. It lacks the Active Phytochrome B binding (APB) motif that is similar to that of the liverwort *Marchantia polymorpha* PIF protein.

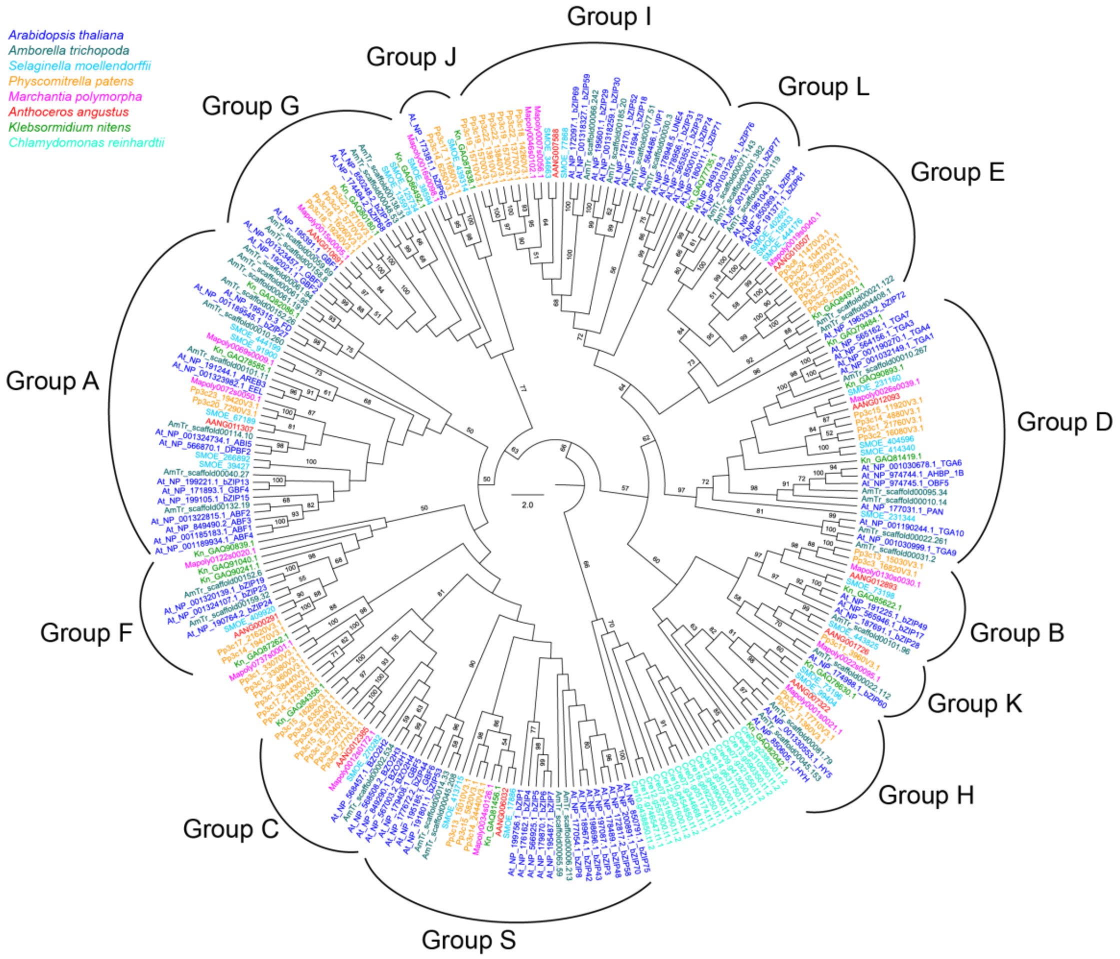

Supplementary Figure 33. Phylogeny of bZIP proteins from land plants and green algae. Bootstrap support values ≥ 50% are shown above the branches. Phylogenetic analysis identified 13 groups (groups A to L and S) within the plant bZIP transcription factor family. Group L is seemingly exclusive to angiosperms and the other 12 groups include members from charophyte green algae and land plants. Group H also contained two sequences from the chlorophyte green alga *Chlamydomonas reinhardtii*. All the other bZIP proteins in *C. reinhardtii* formed an independent clade. The *Anthoceros angustus* genome encodes 11 bZIP proteins, with a single copy in all the above groups except J and L.

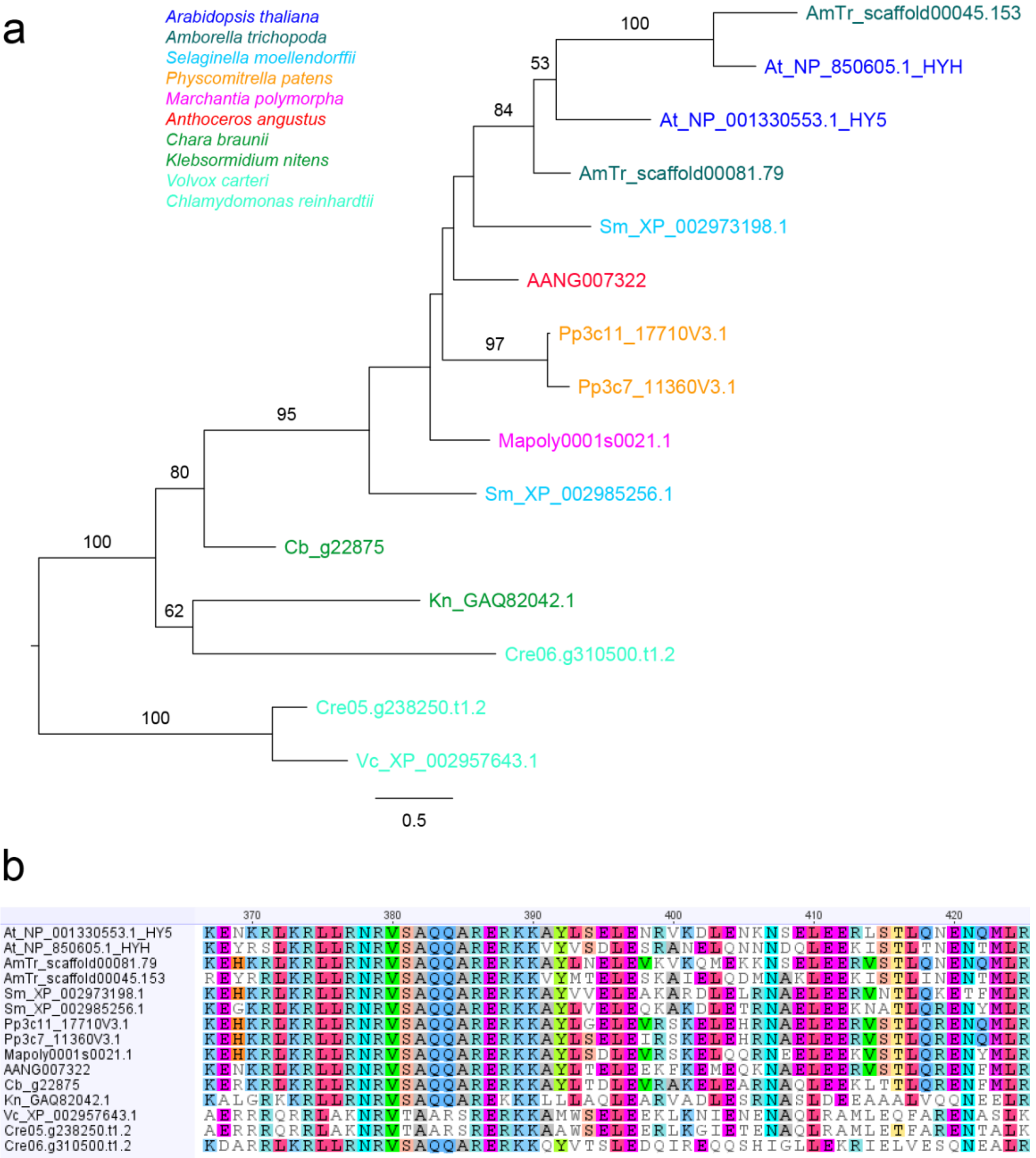

Supplementary Figure 34. Relationships and characteristics of of HY5 proteins from land plants and green algae. a, Phylogeny of HY5 proteins rooted on sequences of the chlorophyte green algae. Bootstrap support values ≥ 50% are shown above the branches. The *HY5* genes belong to the group H of the bZIP family, which includes members from both streptophytes and chlorophytes. The *Anthoceros angustus* genome encodes only a single HY5 protein (AANG007322). **b**, Sequence alignment of bZIP domain sequences of HY5 proteins. The *A. angustus* HY5 protein shows a high identity to homologs of other green plants in bZIP domain alignment.

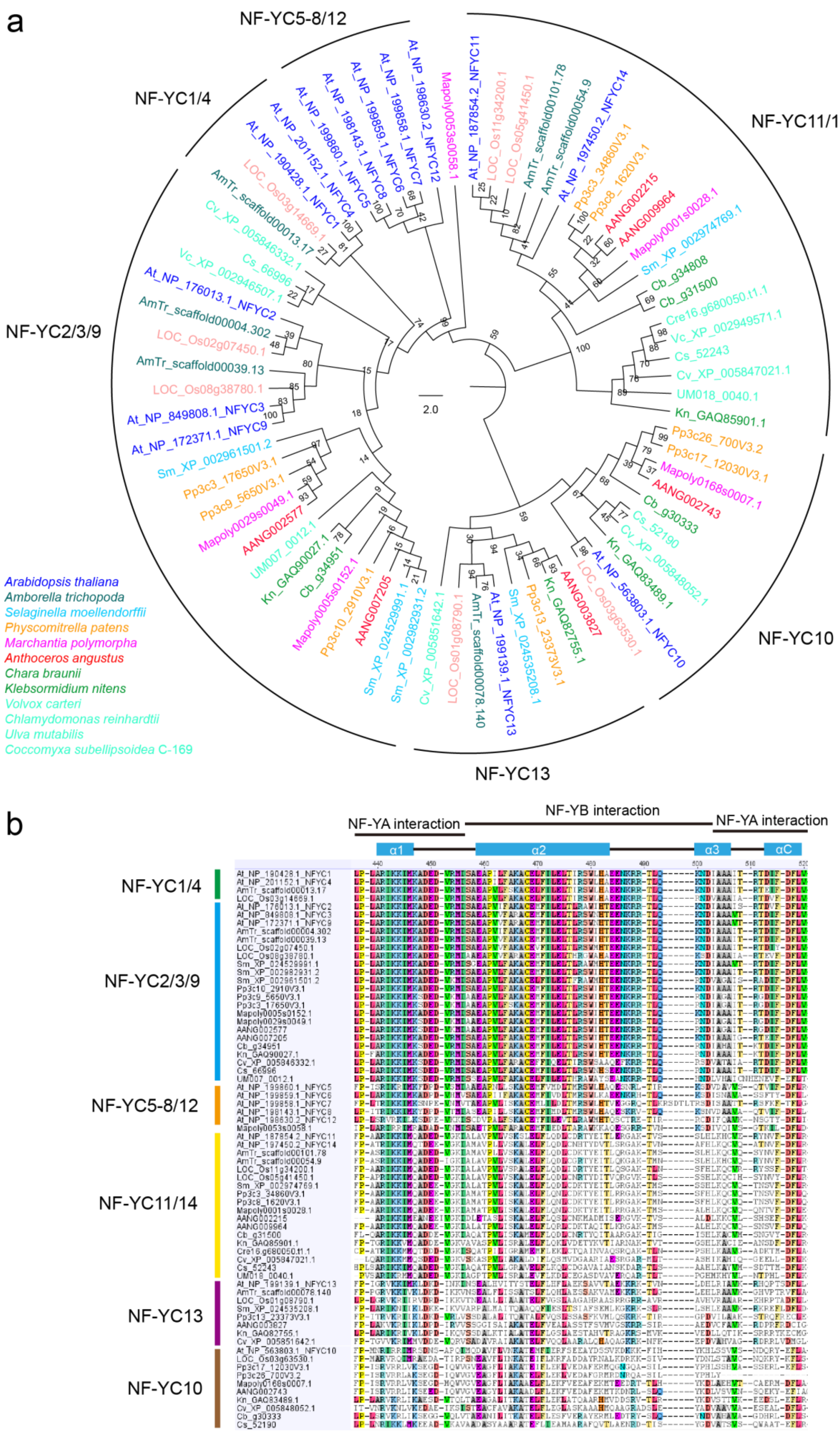
a
NF-YC5-8/12
NF-YC1/4
NF-YC11/14
NF-YC2/3/9
NF-YC10
NF-YC13
2.0
Arabidopsis thaliana
Amborella trichopoda
Selaginella moellendorffii
Physcomitrella patens
Marchantia polymorpha
Anthoceros angustus
Chara braunii
Klebsormidium nitens
Volvox carteri
Chlamydomonas reinhardtii
Ulva mutabilis
Coccomyxa subellipsoidea C-169
b
NF-YA interaction
NF-YB interaction
NF-YA interaction
α1
α2
α3
αC
NF-YC1/4
NF-YC2/3/9
NF-YC5-8/12
NF-YC11/14
NF-YC13
NF-YC10

Supplementary Figure 35. Relationships and characteristics of NF-YC proteins from land plants and green algae. a, Phylogeny of NF-YC proteins. Bootstrap support values ≥ 50% are shown above the branches. NF-YC proteins are resolved in six groups, which are named based on the members from *Arabidopsis*, NF-YC1/4, NF-YC2/3/9, NF-YC5-8/12, NF-YC10, NF-YC13, and NF-YC11/14. The NF-YC1/4 and NF-YC5-8/12 groups include only members of angiosperms, while the other four groups include members from charophyte green algae and land plants. The group NF-YC11/14 also contains one sequence from chlorophyte green alga *Chlamydomonas reinhardtii*, suggesting that it is an ancient group that occurred in the ancestor of green plants. The *Anthoceros angustus* genome encodes six NF-YC proteins belonging to four groups of the NF-YC family except for NF-YC1/4 and NF-YC5-8/12. **b**, Sequence alignment of conserved domains of NF-YC proteins. The *A. angustus* NF-YC proteins contained the conserved DNA-binding and protein interaction domains.

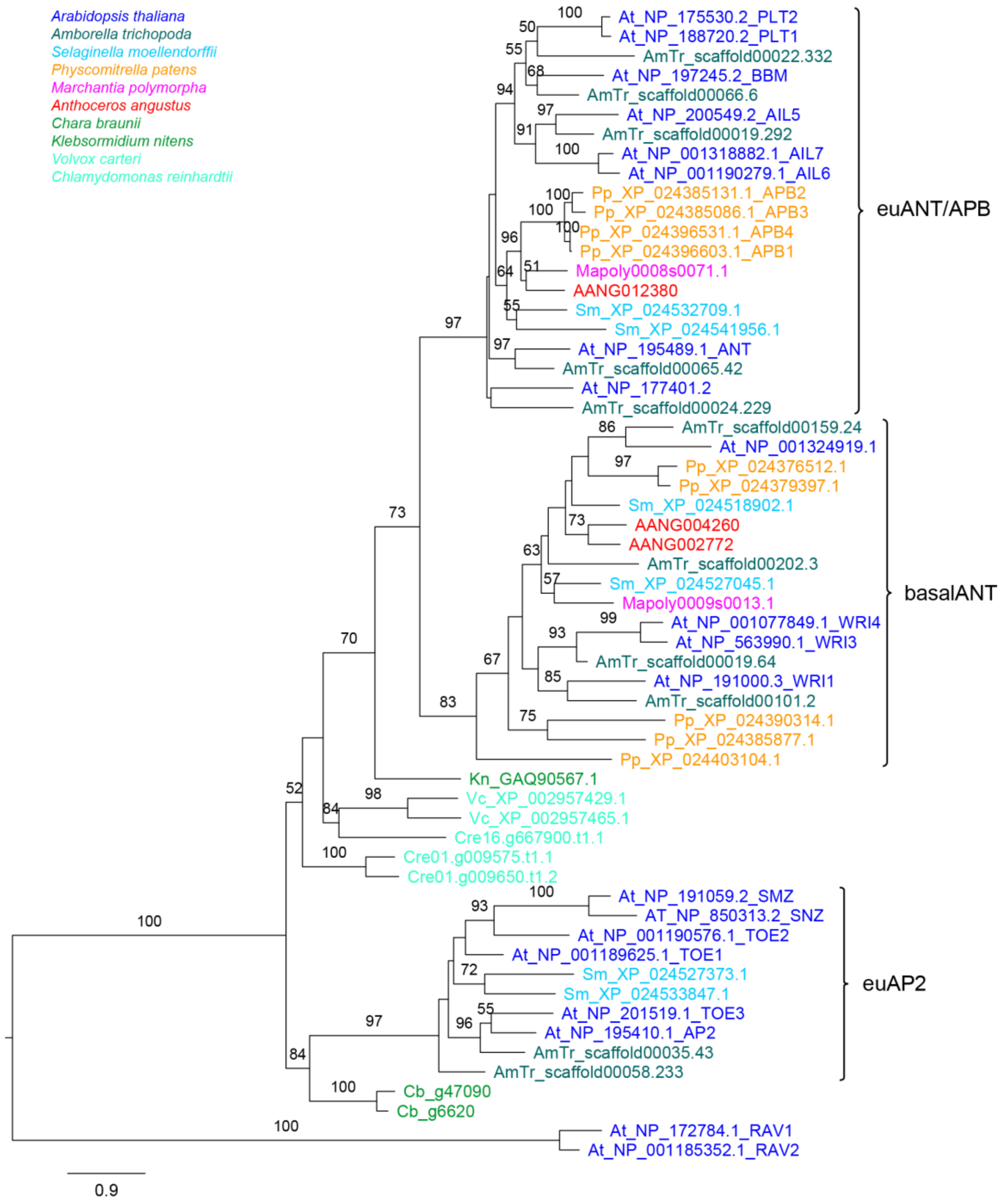

Supplementary Figure 36. Phylogeny of AP2 domain proteins from land plants and green algae. The NP_172784.1_RAV1 and NP_001185352.1_RAV2 sequences of *Arabidopsis thaliana* was used as outgroup. Bootstrap support values ≥ 50% are shown above the branches. The phylogenetic analysis resolved AP2 domain proteins in three lineages, euANT, basalANT, and euAP2. The four *APB* genes of *Physcomitrella patens* for 3D growth are members of the *euANT* lineage. ANT-like proteins are found in the charophyte green alga *Klebsormidium nitens* and the chlorophyte green algae *Chlamydomonas reinhardtii* and *Volvox Carteri*. Two euAP2-like proteins are found in the charophyte green alga *Chara braunii*. One APB-like protein (AANG012813) and two basalANT member (AANG004260 and AANG002772) occur in the *Anthoceros angustus* genome.

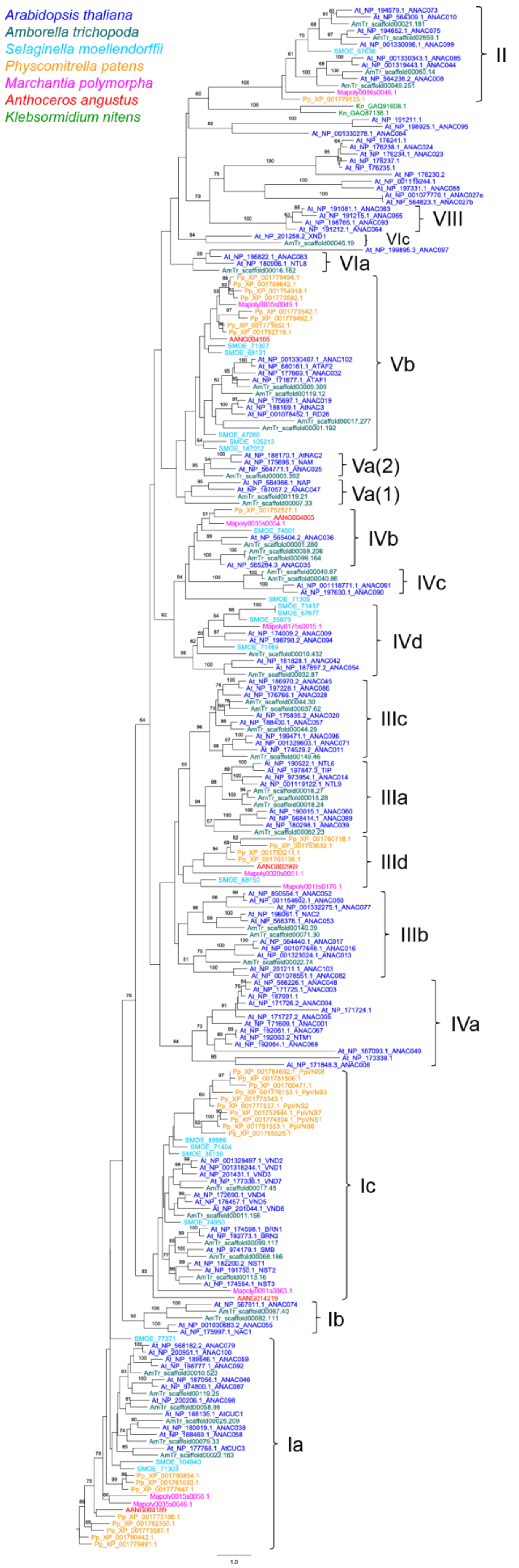

Arabidopsis thaliana
Amborella trichopoda
Selaginella moellendorffii
Physcomitrella patens
Marchantia polymorpha
Anthoceros angustus
Klebsormidium nitens
II
VIII
VIc
VIa
Vb
Va(2)
Va(1)
IVb
IVc
IVd
IIIc
IIIa
IIId
IIIb
IVa
Ic
Ib
Ia
1.0

Supplementary Figure 37. Phylogeny of NAC proteins from land plants and green algae. Bootstrap support values ≥ 50% are shown above the branches. NAC proteins are resolved in 18 subfamilies. Subgroup IIId is new and includes only NAC protein sequences from bryophytes and *Selaginella moellendorffii*. In addition to IIId, the subfamilies Ia, Ic, II, IVb, IVd, and Vb also include members from bryophytes. Other subfamilies are mainly restricted to flowering plants. The five *Anthoceros angustus* NAC proteins belong to Ia, Ic, IIId, IVb, and Vb subfamilies, respectively, and the homologs of II and IVd NACs were absent in *A. angustus*.

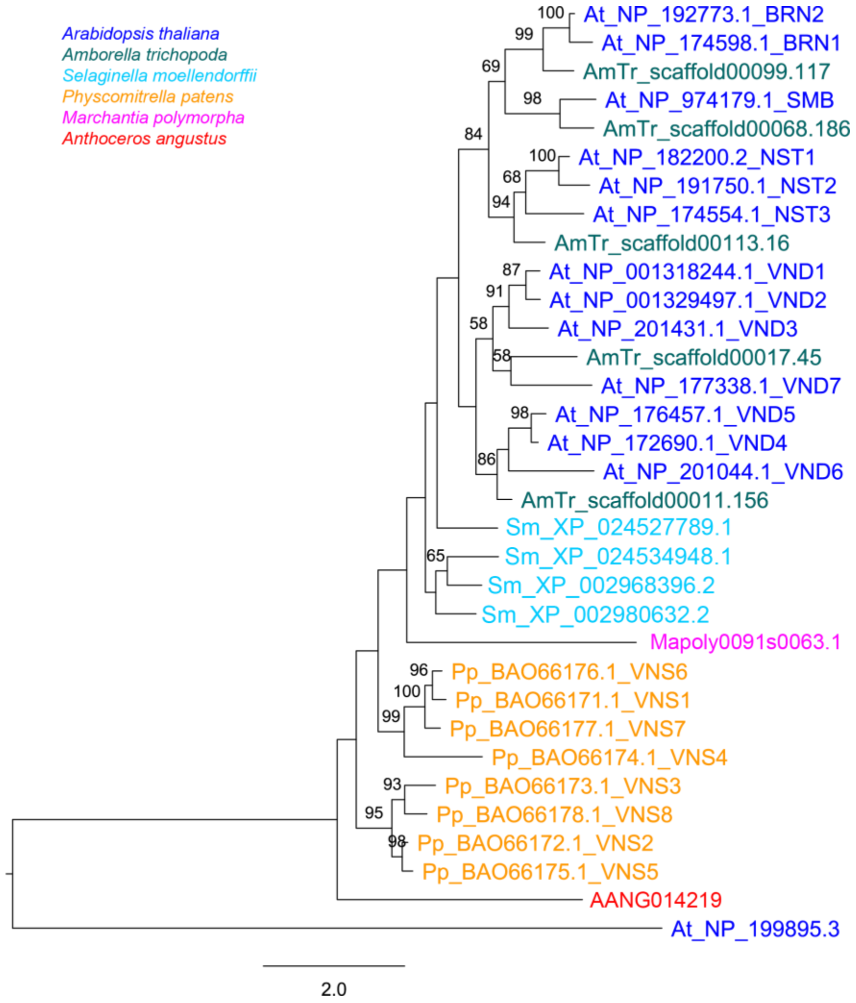

Supplementary Figure 38. Phylogeny of VNS proteins from land plants. The NP_199895.3 (ANAC097) sequence of *Arabidopsis thaliana* was used as outgroup. Bootstrap support values ≥ 50% are shown above the branches. Four *VNS* genes were found in the lycophyte *Selaginella moellendorffii* and eight in the moss *Physcomitrella patens*, while the liverwort *Marchantia polymorpha* and the hornwort *Anthoceros angustus* (AANG014219) have each a single *VNS* gene.

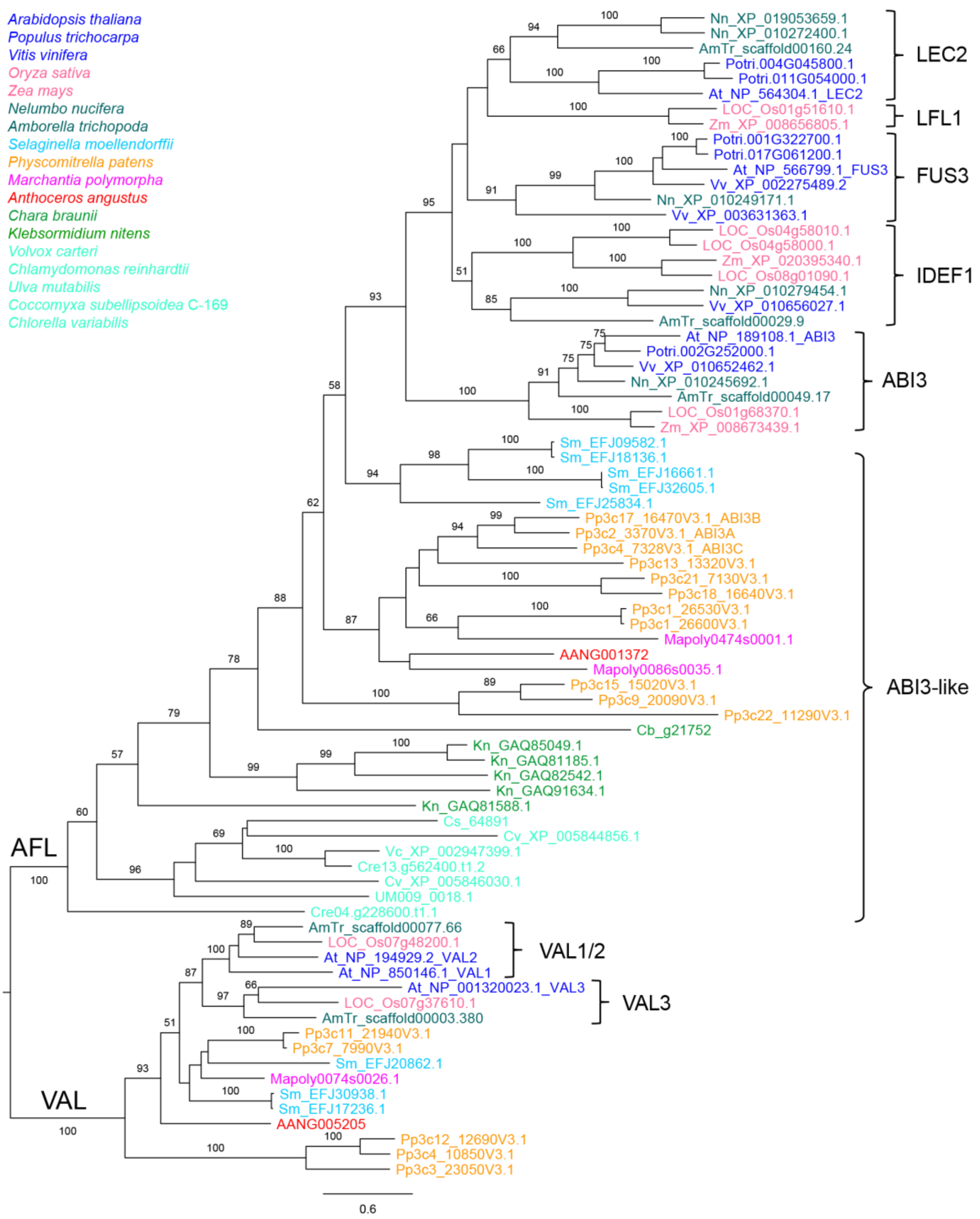

Supplementary Figure 39. Phylogeny of B3-LAV proteins from land plants and green algae. Bootstrap support values ≥ 50% are shown above the branches. Two *LAV* genes occur in the *Anthoceros angustus* genome: AANG001372 belongs to *AFL* subfamily and AANG005205 belongs to *VAL* subfamily. Angiosperm *AFL* genes cluster in five lineages (*ABI3*, *LEC2*, *FUS3*, *IDEF*, and *LFL1*), of which the *ABI3* clade is sister to the remainder. The *A. angustus AFL* gene clustered with other bryophyte *AFL* genes, and belong to a lineage of *ABI3*-like genes.

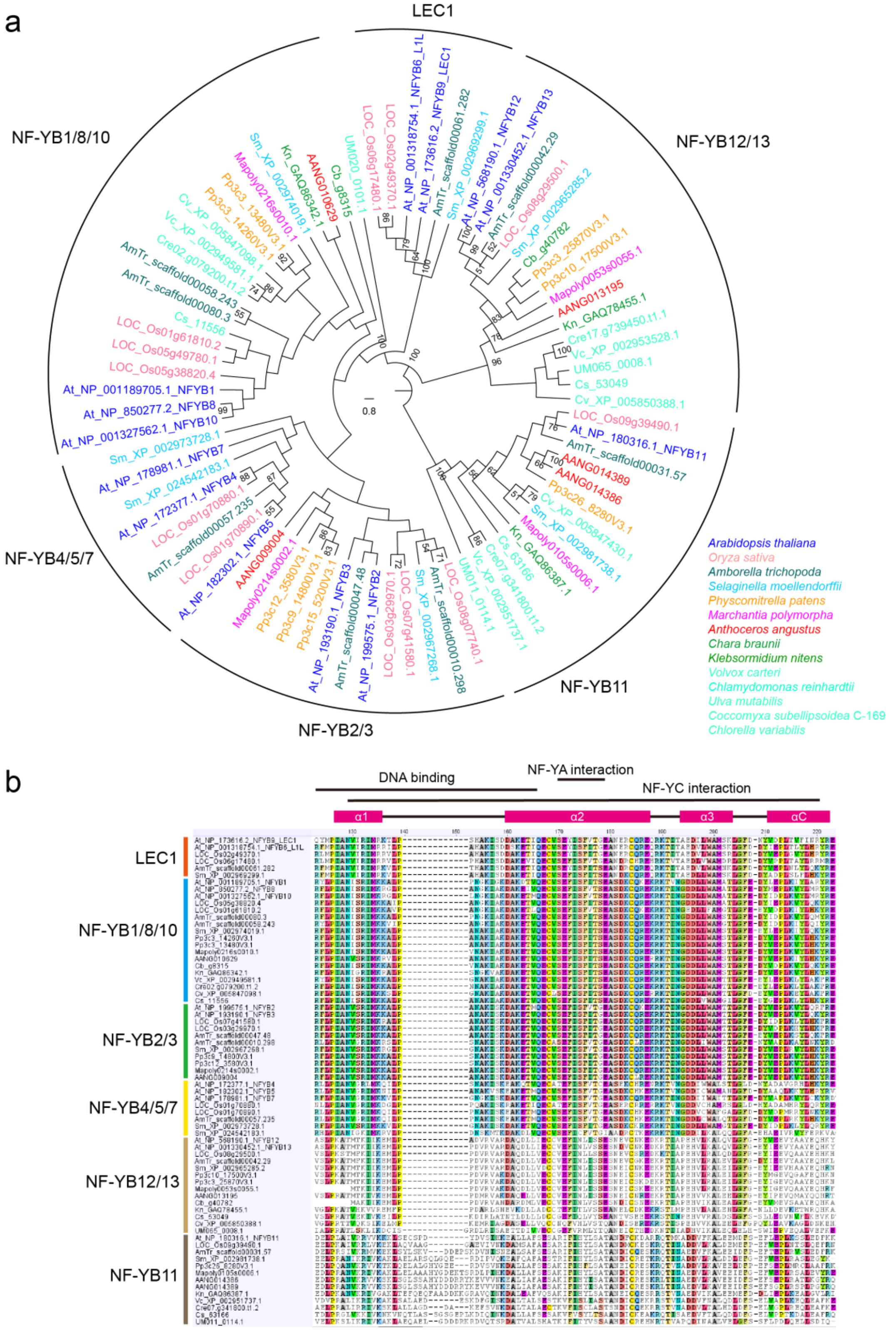

Supplementary Figure 40. Relationships and characteristics of NF-YB proteins from land plants and green algae. a, Phylogeny of NF-YB proteins. Bootstrap support values ≥ 50% are shown above the branches. NF-YB proteins are resolved in six clades, including the LEC1-type and five non-LEC1-type clades. The non-LEC1-type clades were named according to the members of *Arabidopsis*, namely NF-YB1/8/10, NF-YB2/3, NF-YB4/5/7, NF-YB12/13, and NF-YB11. The

five *Anthoceros angustus NF-YB* genes were resolved in the *NF-YB1/8/10* (AANG010629), *NF-YB2/3* (AANG009004), *NF-YB12/13* (AANG013195) and *NF-YB11* (AANG014386 and AANG014389) clades. **b**, Sequence alignment of conserved domains of NF-YB proteins. The *A. angustus* NF-YB proteins contained the conserved DNA-binding and protein interaction domains.

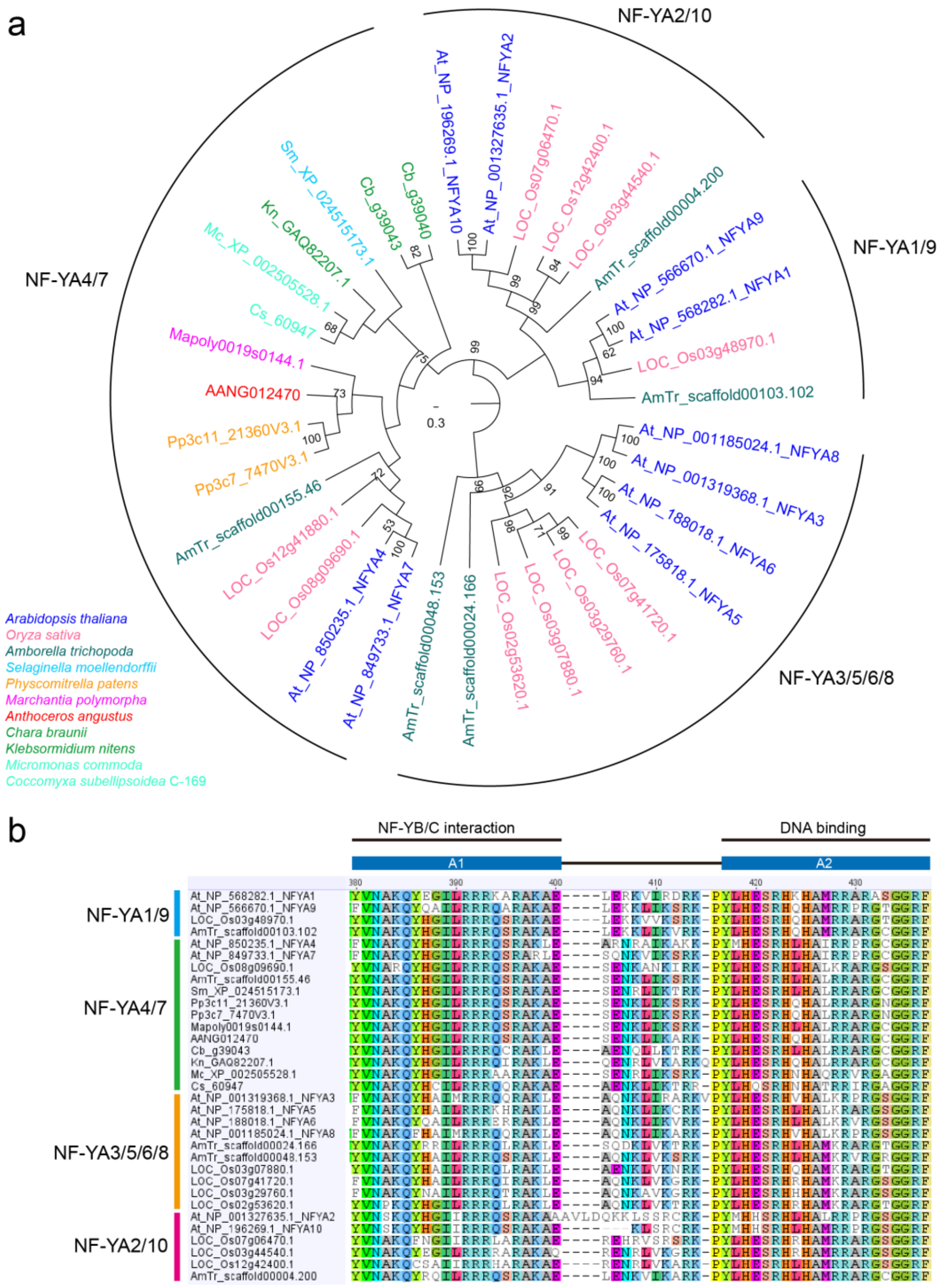

Supplementary Figure 41. Relationships and characteristics of NF-YA proteins from land plants and green algae. a, Phylogeny of NF-YA proteins. Bootstrap support values ≥ 50% are shown above the branches. Phylogenetic analysis showed that NF-YA proteins were divided into four clades, which were named according to the members of *Arabidopsis*, NF-YA1/9, NF-YA2/10, NF-YA3/5/6/8, and NF-YA4/7. A single *NF-YA* gene (AANG012470) occurs in the *Anthoceros angustus* genome. It belongs to the *NF-YA4/7* clade, which is the sole clade comprising homologs from green algae. **b**, Sequence alignment of conserved domains of NF-YA proteins. The *A. angustus* NF-YA protein contained the conserved DNA-binding and protein interaction domains.

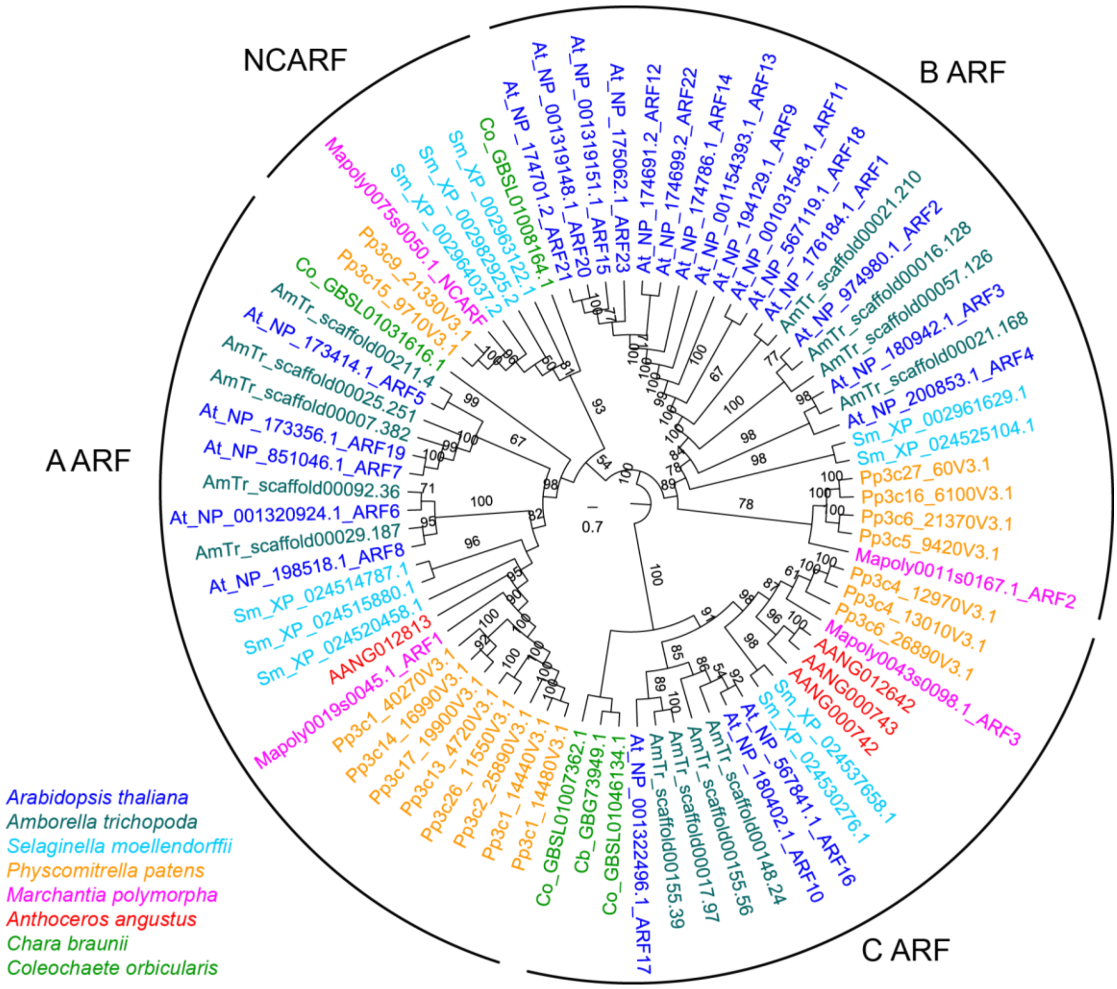

Supplementary Figure 42. Phylogeny of B3-ARF proteins from land plants and green algae. Bootstrap support values ≥ 50% are shown above the branches. The phylogenetic analysis revealed three canonical ARF classes, A, B, and C, and one non- canonical (NC) ARF clade sister to class A. The class A and class C ARFs include members of both land plants and charophyte green algae, indicating at least some classes of land plant ARFs already existed in freshwater green algae. The class B ARFs are restricted to land plants in our analysis. The NCARFs without B3 domain were only found in non-seed plants, implying loss of corresponding genes in seed plants. The *Anthoceros angustus* genome holds one class A *ARF* gene (AANG012813) and three class C *ARF* genes (AANG012642, AANG000742 and AANG000743), and lacks genes of class B ARF and NCARF.

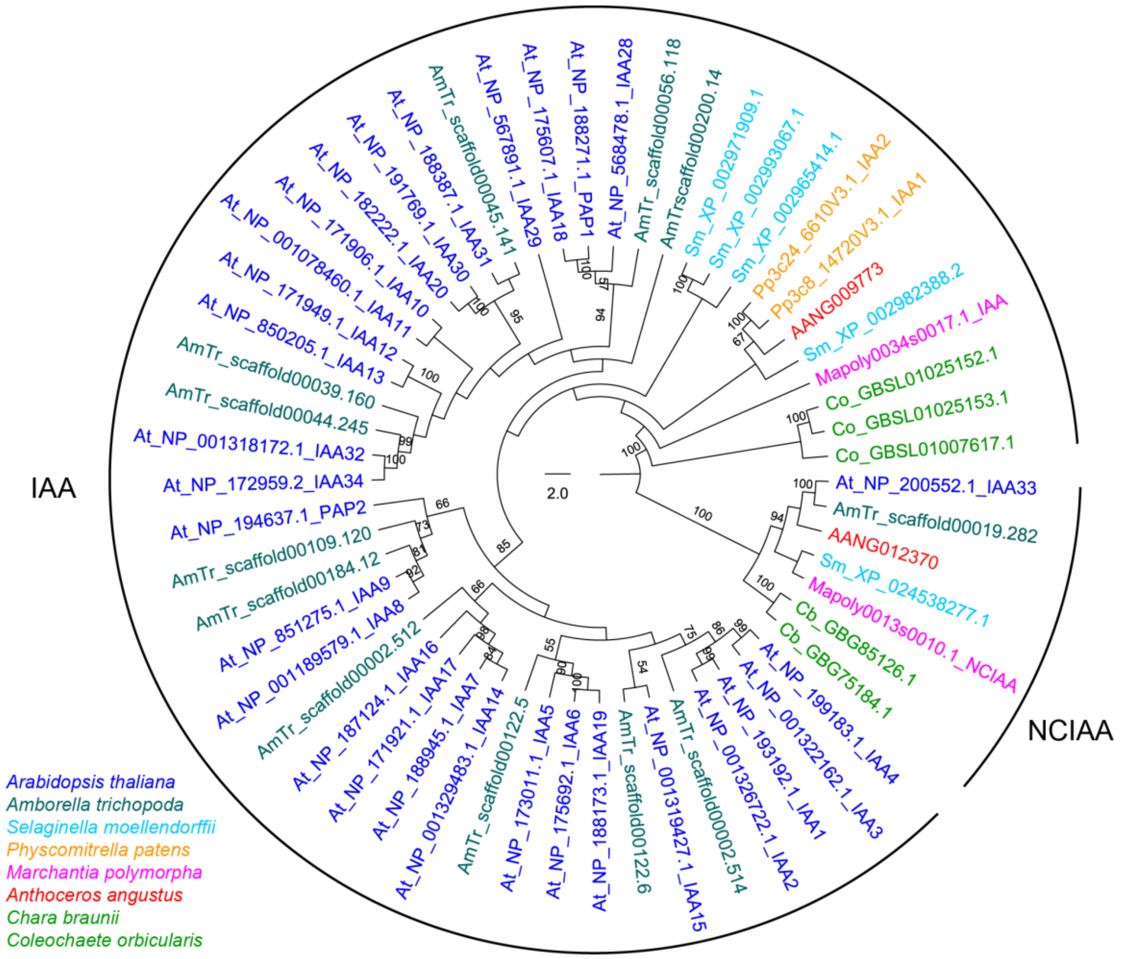

Supplementary Figure 43. Phylogeny of Aux/IAA proteins from land plants and green algae. Bootstrap support values ≥ 50% are shown above the branches. Canonical Aux/IAAs (IAA) and non-canonical Aux/IAAs (NCIAA) compose two sister groups. The IAA clade and NC IAA clade include members of both land plants and charophyte green algae, suggesting that an origin of Aux/IAA proteins in the common ancestor of streptophytes. Two *Aux/IAA* genes occur in the the *Anthoceros angustus* genome, with AANG009773 resolved in the *IAA* clade and AANG012370 in the *NCIAA* clade. Compared to the low number of *Aux/IAA* genes in the lycophyte, bryophytes and charophyte green algae, flowering plants hold a highly expanded set of *Aux/IAA* genes.

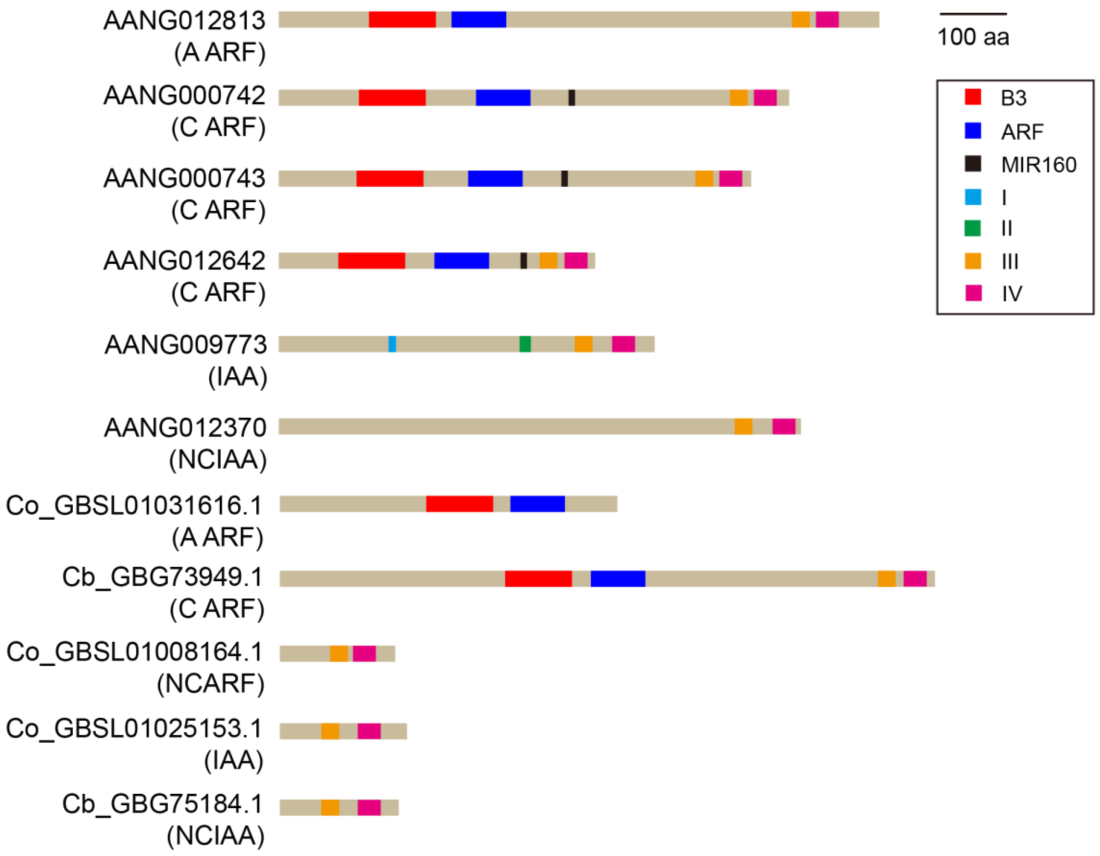

Supplementary Figure 44. Domain architecture of ARF and Aux/IAA proteins. Co: *Coleochaete orbicularis*; Cb: *Chara braunii*. All four *Anthoceros angustus* (AANG012813, AANG000742, AANG000743, and AANG012642) ARFs have typical ARF structures like those of other land plants, containing a B3 DNA binding domain, an ARF domain and domains III and IV for protein-protein interaction with Aux/IAAs. Furthermore, the three class C ARFs (AANG000742, AANG000743, and AANG012642) also possess microRNA160 (miR160) target sites. In contrast, no miR160 target sites were found in charophyte green algae class C ARFs, implying that the regulation of ARFs by miR160 might have evolved in the common ancestor of land plants. In *A. angustus*, AANG009773 (IAA clade member) has four conserved characteristic domains (I-IV) and AANG012370 (NCIAA clade member) only contains domain III and IV. In charophyte green algae, members of both IAA clade and NCIAA clade only contains domain III and IV and lack domains I and II for stabilizing Aux/IAA proteins, suggesting the domains I and II have evolved in land plants.

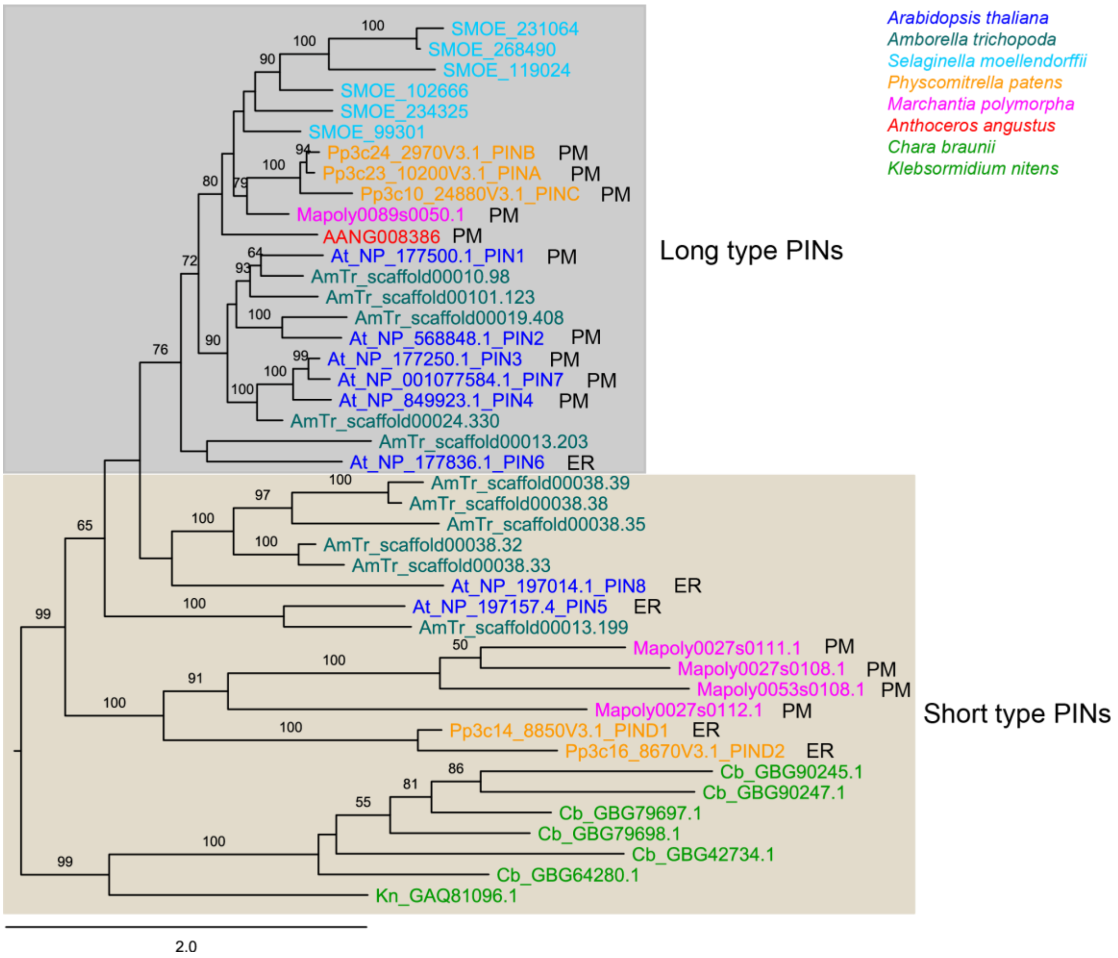

Supplementary Figure 45. Phylogeny of PIN proteins from land plants and green algae. The tree is rooted on sequences of the charophyte green algae. Bootstrap support values ≥ 50% are shown above the branches. The subcellular localization is shown near the protein name, plasma-membrane (PM) and endoplasmic reticulum (ER). PIN proteins occur in both land plants and charophyte green algae, implying that the origin of PIN proteins could be traced back to the common ancestor of streptophytes. The seven charophyte green algae PIN proteins are all 'short type' PINs according to the size of their central HL. The *Anthoceros angustus* genome lacks short type PIN proteins but holds a single PIN protein (AANG008386), which is resolved within the long PIN protein clade, and is located in the plasma-membrane.

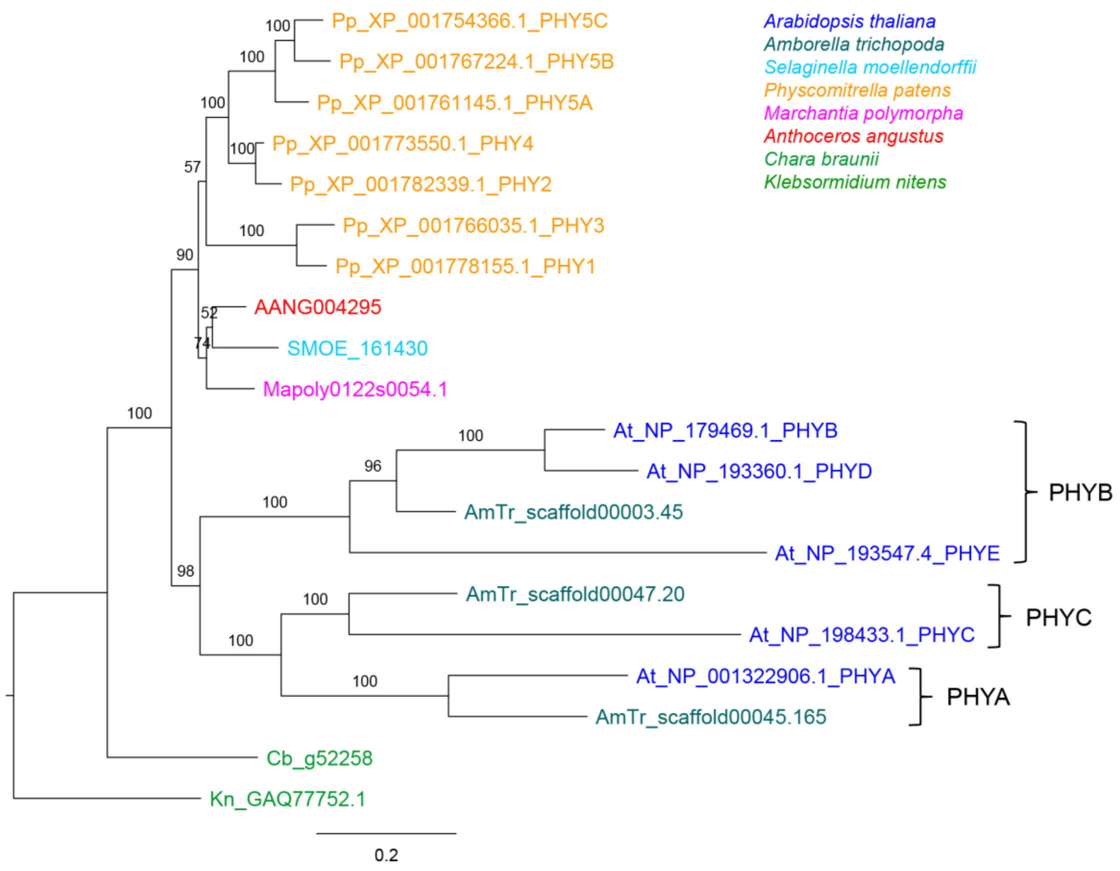

Supplementary Figure 46. Phylogeny of phytochrome proteins from land plants and green algae. The ML tree shown is rooted with the charophyte green alga *Klebsormidium nitens* sequence. Bootstrap support values ≥ 50% are shown above the branches. In the phylogenetic analysis, canonical phytochrome sequences form a well-supported monophyletic group composed of three clades (i.e., PHYA, PHYB and PHYC) in the flowering plants. Phytochromes are encoded by a single-copy gene in the hornwort *Anthoceros angustus* (AANG004295) and the liverwort *Marchantia polymorpha*, but seven copies in the moss *Physcomitrella patens*.

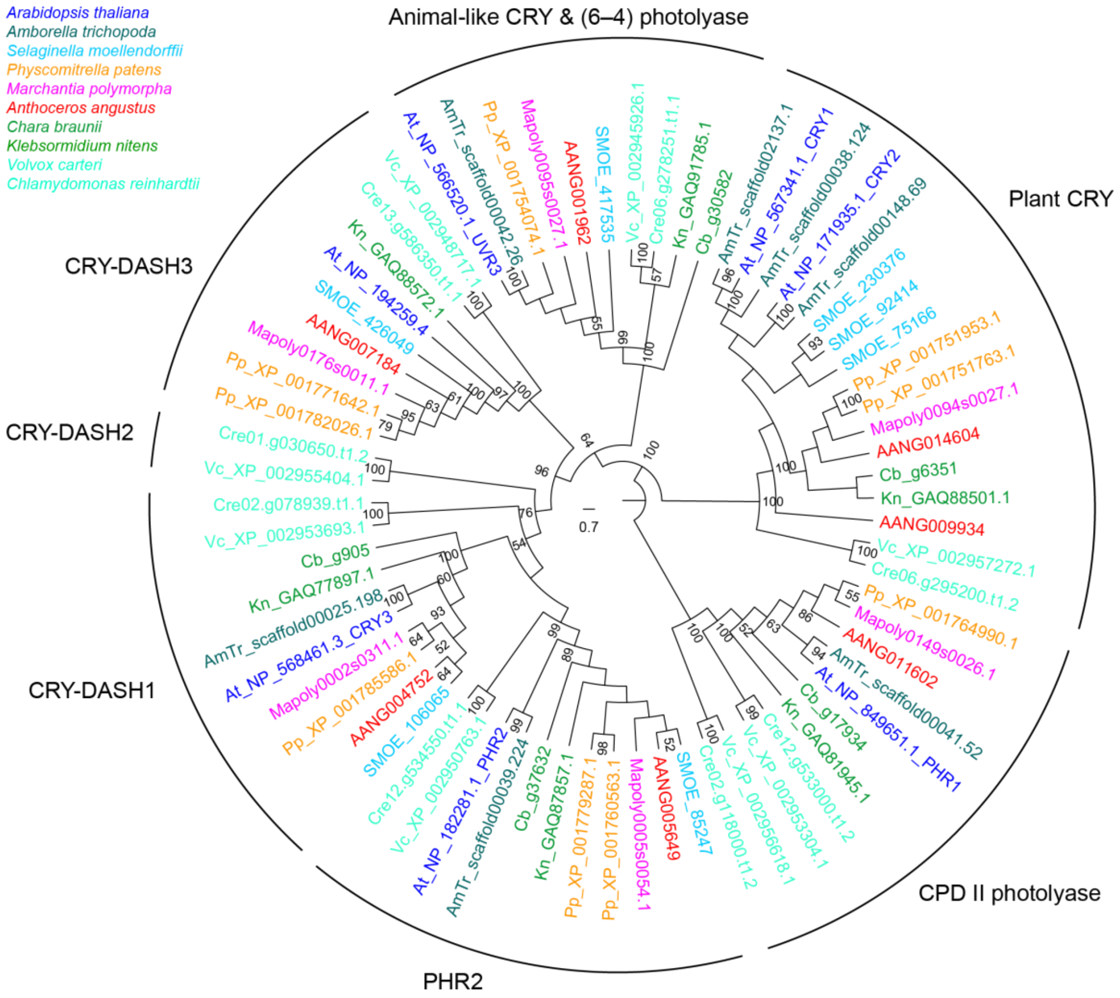

Supplementary Figure 47. Phylogeny of cryptochrome proteins from land plants and green algae. Bootstrap support values ≥ 50% are shown above the branches. The CPF family in plant lineages comprises seven well-supported clades, including the plant CRY, CPD II photolyase, animal-like CRY & (6–4) photolyase, CRY- DASH1, CRY- DASH2, CRY- DASH3, and PHR2. The CRY-DASH3 is a newly identified clade. The CRY-DASH2 is a green algae specific clade. The other five clades include members of both land plants and green algae. The seven *Anthoceros angustus* CPF proteins are distributed among six of the seven clades, two in the plant CRY clade, one in the CPD II photolyase clade, one in the animal-like CRY & (6–4) photolyase clade, one in the CRY- DASH1 clade, one in the CRY- DASH3, and one in the PHR2 clade.

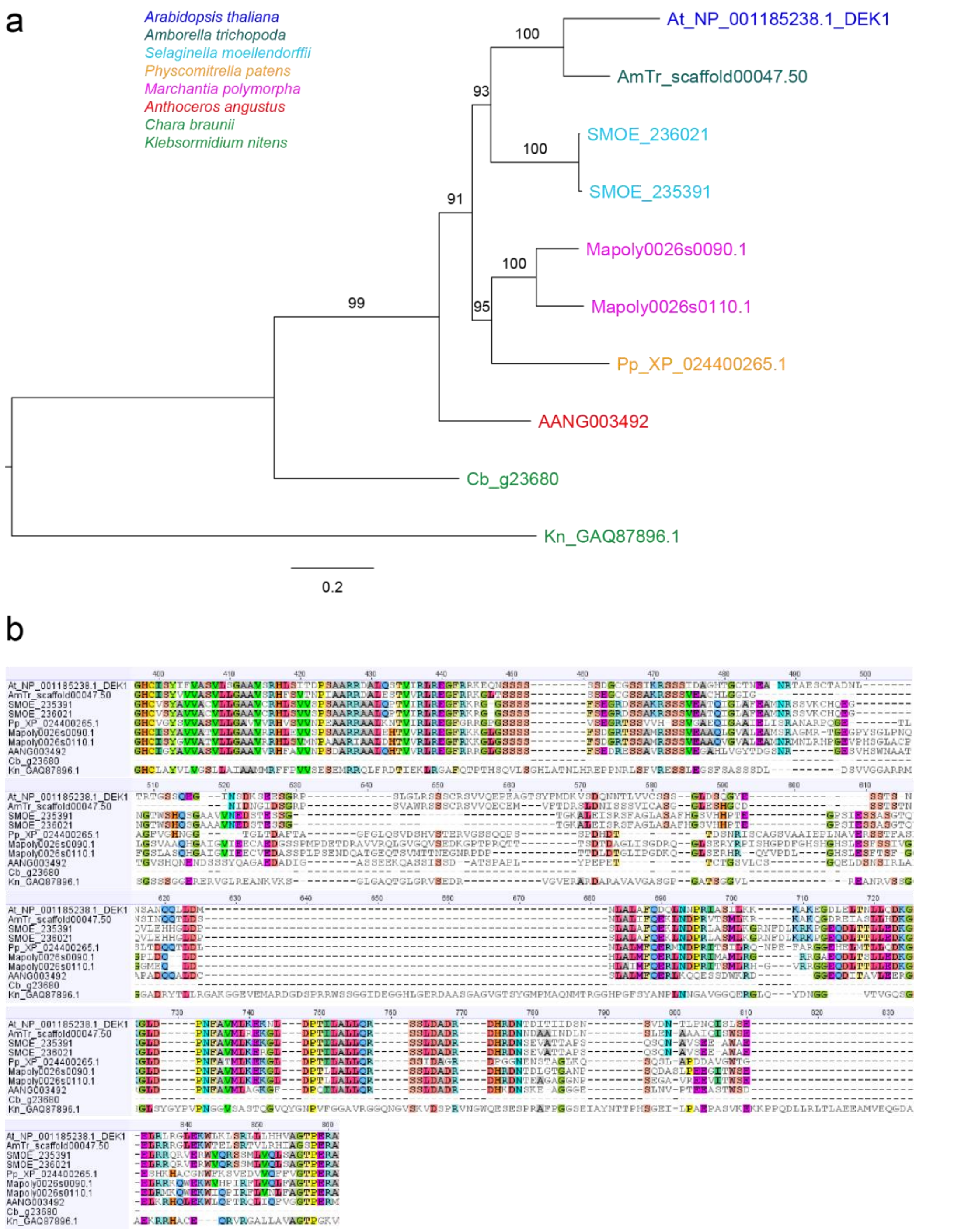

Supplementary Figure 48. Relationships and characteristics of DEFECTIVE KERNEL1 (DEK1) proteins from land plants and green algae. a, Phylogeny of DEK1 proteins rooted with the charophyte green alga *Klebsormidium nitens* sequence. Bootstrap support values ≥ 50% are shown above the branches. One DEK1 homologous sequence (AANG003492) occurs in the *Anthoceros angustus* genome. **b**, Alignment of Loop sequences of DEK1 proteins. The Loop sequences are conserved in land plants, while the charophyte green alga *K. nitens* Loop sequence is highly divergent from the corresponding land plant sequences and *C. braunii* lacks the Loop sequence. It point toward a functional divergence of DEK1 proteins in algae and land plants.

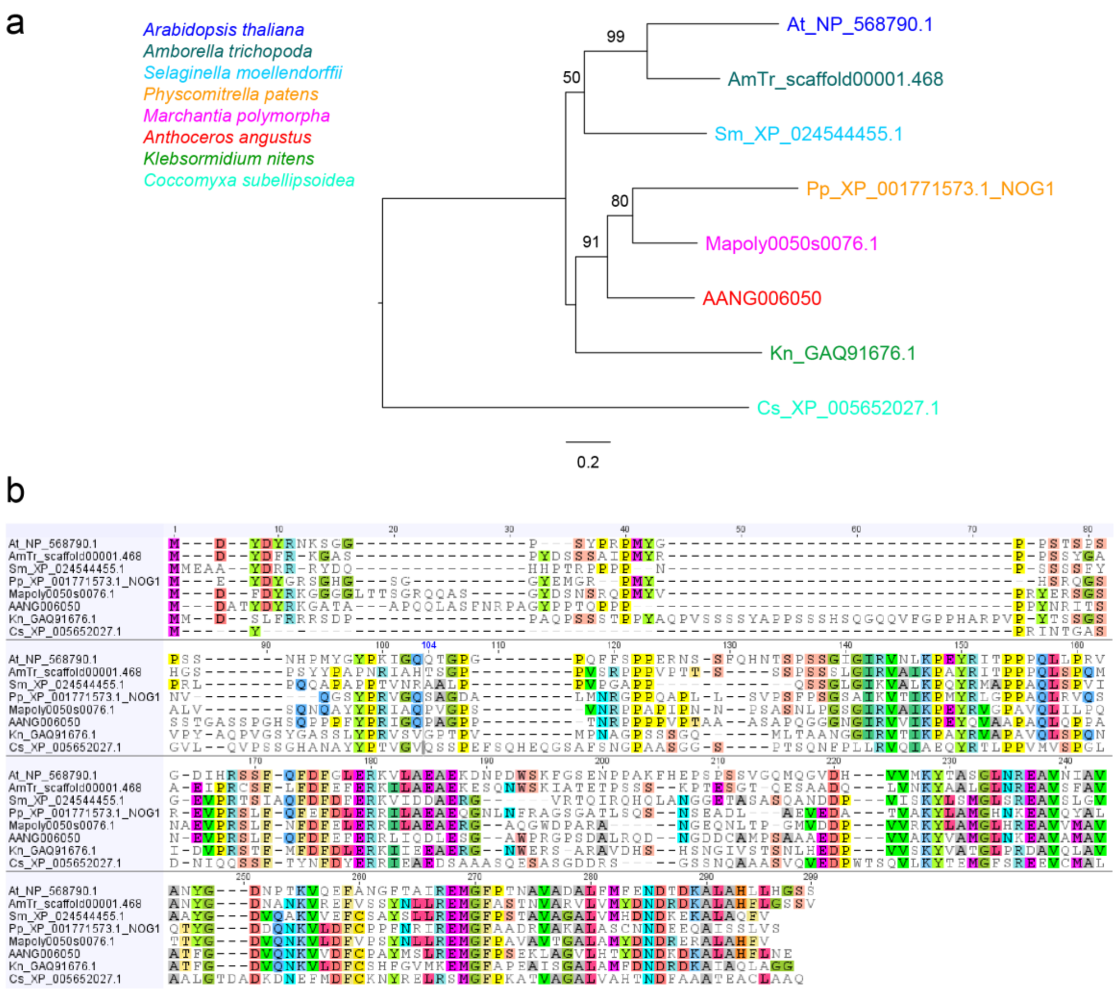

Supplementary Figure 49. Relationships and characteristics of NO GAMETOPHORES 1 (NOG1) proteins from land plants and green algae. a, Phylogeny of NOG1 proteins rooted with the chlorophyte green alga *Coccomyxa subellipsoidea* sequence. Bootstrap support values ≥ 50% are shown above the branches. One NOG1 homologous sequence (AANG006050) occurs in the *Anthoceros angustus* genome. **b**, Sequence alignment of NOG1 proteins. The chlorophyte green alga NOG1 protein is highly divergent from the corresponding streptophyta sequences.

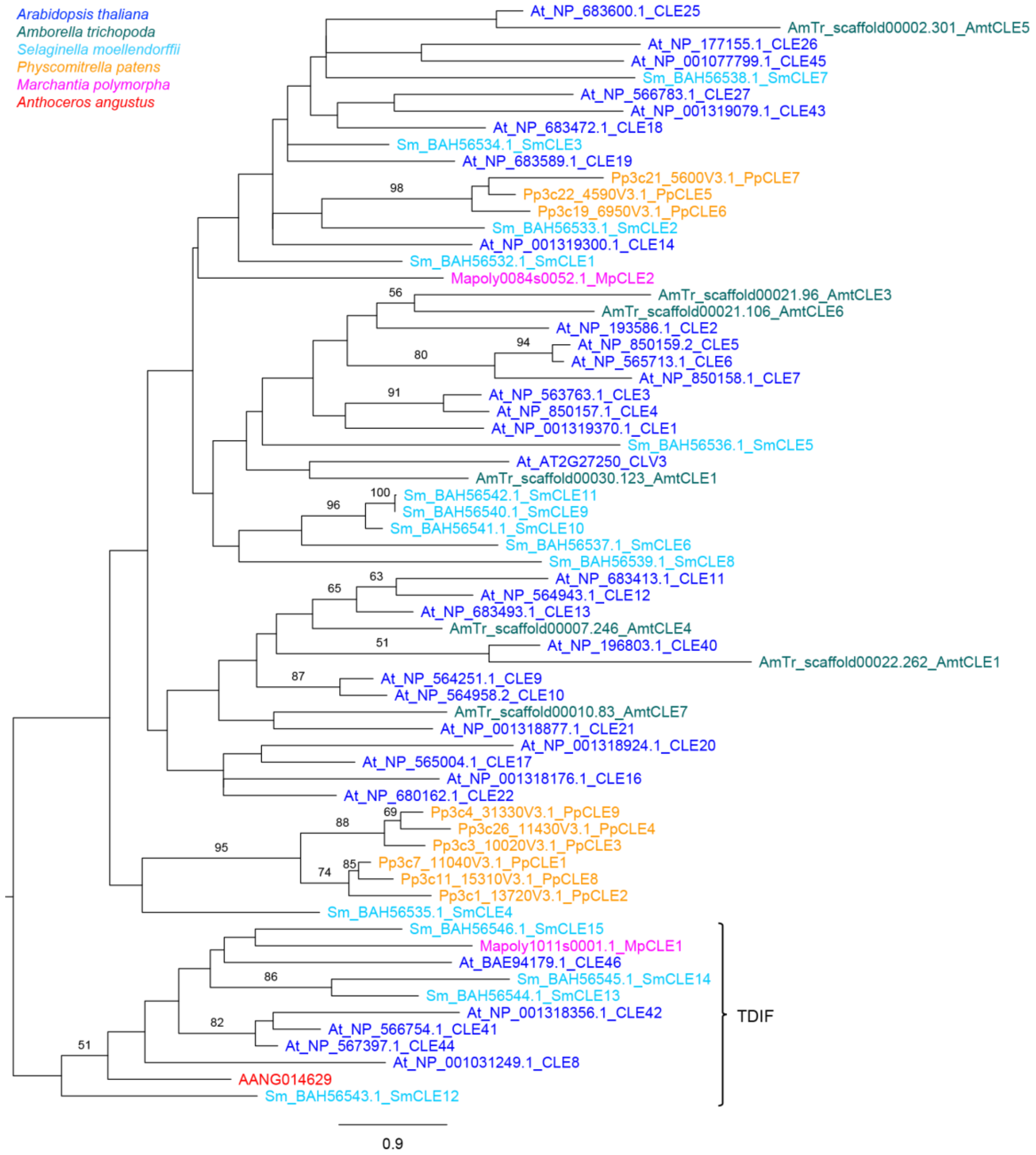

Supplementary Figure 50. Phylogeny of CLE proteins from land plants. The ML tree shown is rooted with the tracheary element differentiation inhibitory factor (TDIF) clade of CLEs. Bootstrap support values ≥ 50% are shown above the branches. The *Anthoceros angustus* genome encodes one TDIF-type CLE (AANG014629) that is homologous to MpCLE1 of *Marchantia polymorpha* regulating cell proliferation in the haploid meristem. No CLE homologs were found in the green algae genomes.

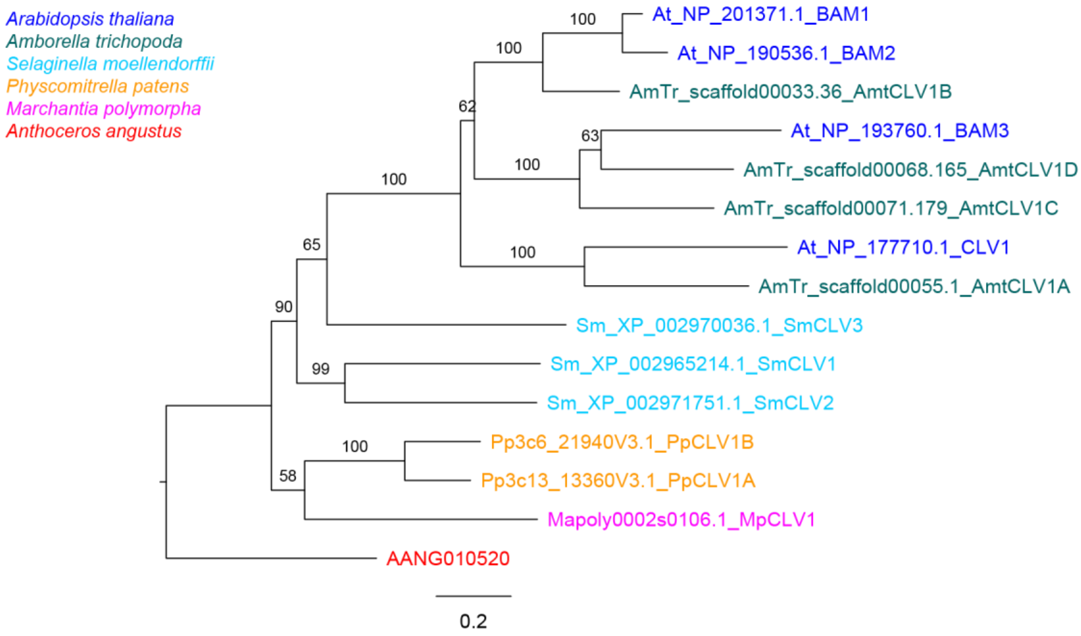

Supplementary Figure 51. Phylogeny of CLV1/BAM proteins from land plants. The ML tree shown is rooted with the *Anthoceros angustus* sequence. Bootstrap support values ≥ 50% are shown above the branches. The *A. angustus* genome encodes one CLV1 protein (AANG010520). No CLV1 homologs were found in the green algae genomes.

		Vascular plants			Mosses	Liverworts	Hornworts	Charophytes		Chlorophytes		
**Family**	**Clade**	***A. thaliana***	***A. trichopoda***	***S. moellendorffii***	***P. patens***	***M. polymorpha***	***A. angustus***	***C. braunii***	***K. nitens***	***V.carteri***	***C. reinhardtii***	**Function**
PHY		5	3	1	7	1	1	1	1	0	0	photomorphogenesis
CRY	plant CRY	2	3	3	2	1	2	1	1	1	1	
Aux/IAA		29	16	9	2	2	2	2	0	0	0	auxin signaling
PIN		8	10	6	5	5	1	6	1	0	0	
DEK1		1	1	2	1	2	1	1	1	0	0	3D growth
NOG1		1	1	1	1	1	1	0	1	0	0	
CLE		32	7	15	9	2	1	0	0	0	0	
CLV1/BAM		4	4	3	2	1	1	0	0	0	0	

Supplementary Figure 52. Summary of gene families (non-transcription factor) for plant body plan in land plants and green algae. Heat map overview for the number of genes was shown.

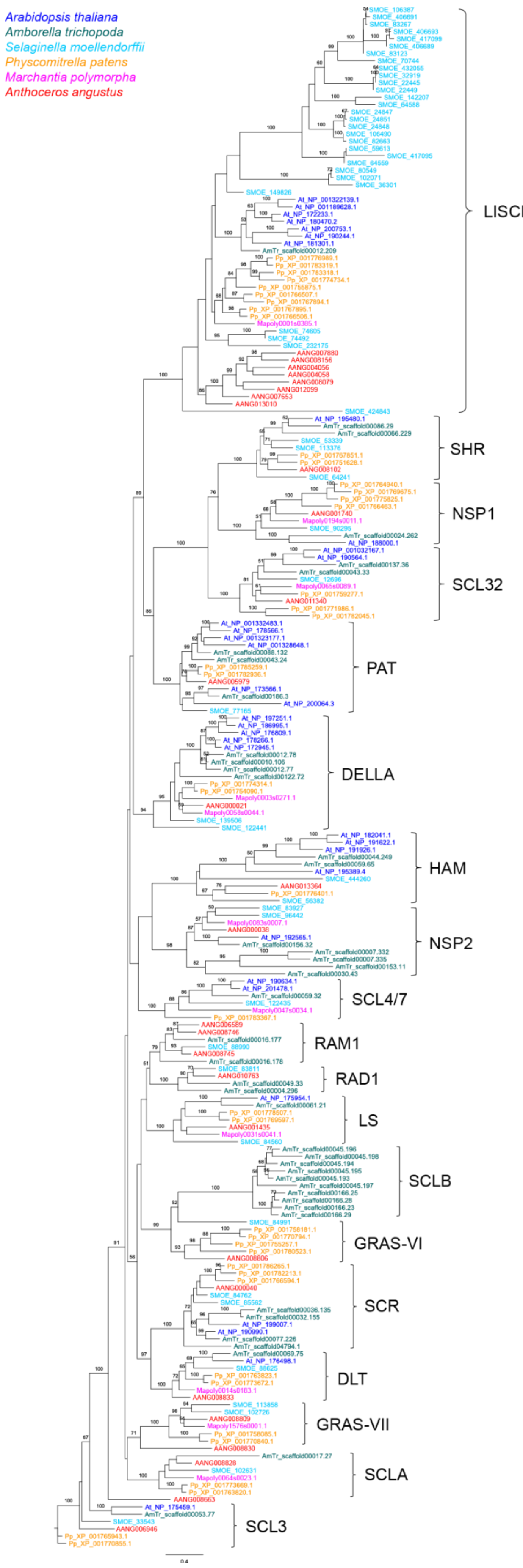

Arabidopsis thaliana
Amborella trichopoda
Selaginella moellendorffii
Physcomitrella patens
Marchantia polymorpha
Anthoceros angustus
LISCL
SHR
NSP1
SCL32
PAT
DELLA
HAM
NSP2
SCL4/7
RAM1
RAD1
LS
SCLB
GRAS-VI
SCR
DLT
GRAS-VII
SCLA
SCL3
0.4

Supplementary Figure 53. Phylogeny of *GRAS* genes from land plants, inferred from the amino acid alignment of the GRAS domain (PFAM profile PF03514). Bootstrap support values ≥ 50% are shown above the branches. Land plant GRAS proteins clustered in 19 clades, of which 17 contain (except for SCL4/7 and SCLB clades) *Anthoceros angustus* orthologs. For most *GRAS* clades, the *A. angustus* genome holds one to three coppes, while for LISCL GRAS proteins eight copies are present. In total, 28 *GRAS* genes are identified in the *A. angustus* genome.

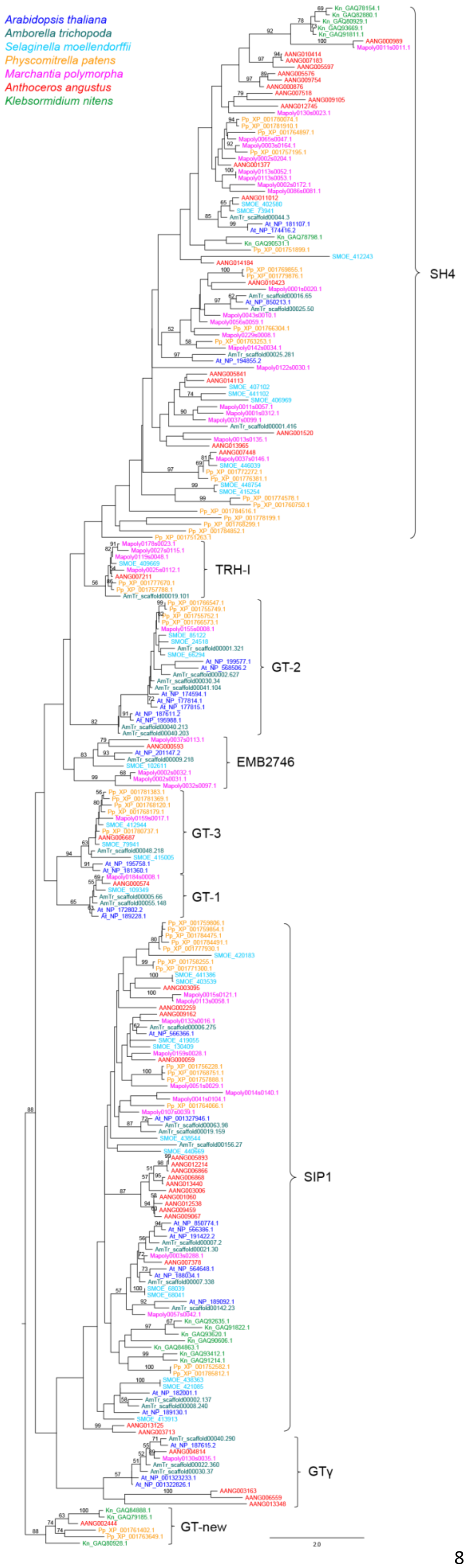
Arabidopsis thaliana
Amborella trichopoda
Selaginella moellendorffii
Physcomitrella patens
Marchantia polymorpha
Anthoceros angustus
Klebsormidium nitens
SH4
TRH-I
GT-2
EMB2746
GT-3
GT-1
SIP1
GTγ
GT-new
2.0

Supplementary Figure 54. Phylogeny of *Trihelix* genes from land plants and green algae, inferred from an amino acid alignment of the Trihelix domain (PFAM profile PF13837). Bootstrap support values ≥ 50% are shown above the branches. Streptophyte trihelix proteins are resolved in nine clades, of which eight were previously known clades (i.e., GT-1, GT-3, EMB2746, GT-2, SH4, GTγ, SIP1, and TRH-I) and one new one, named GT-new, which contains four sequences from *Anthoceros angustus*, two from *Physcomitrella patens*, and three from *Klebsormidium nitens*. In total 45 *trihelix* genes were identified in the *A. angustus* genome. Compared to other land plants, bryophytes have more trihelix members, which may be associated with the responses to light and stress during adaptation to terrestrial environments. Two notable examples are found in *SIP1* and *SH4* clades. The visible lineage-specific expansions in hornwort *A. angustus* (17 members) and moss *P. patens* (13 members) were shown in the *SIP1* clade.

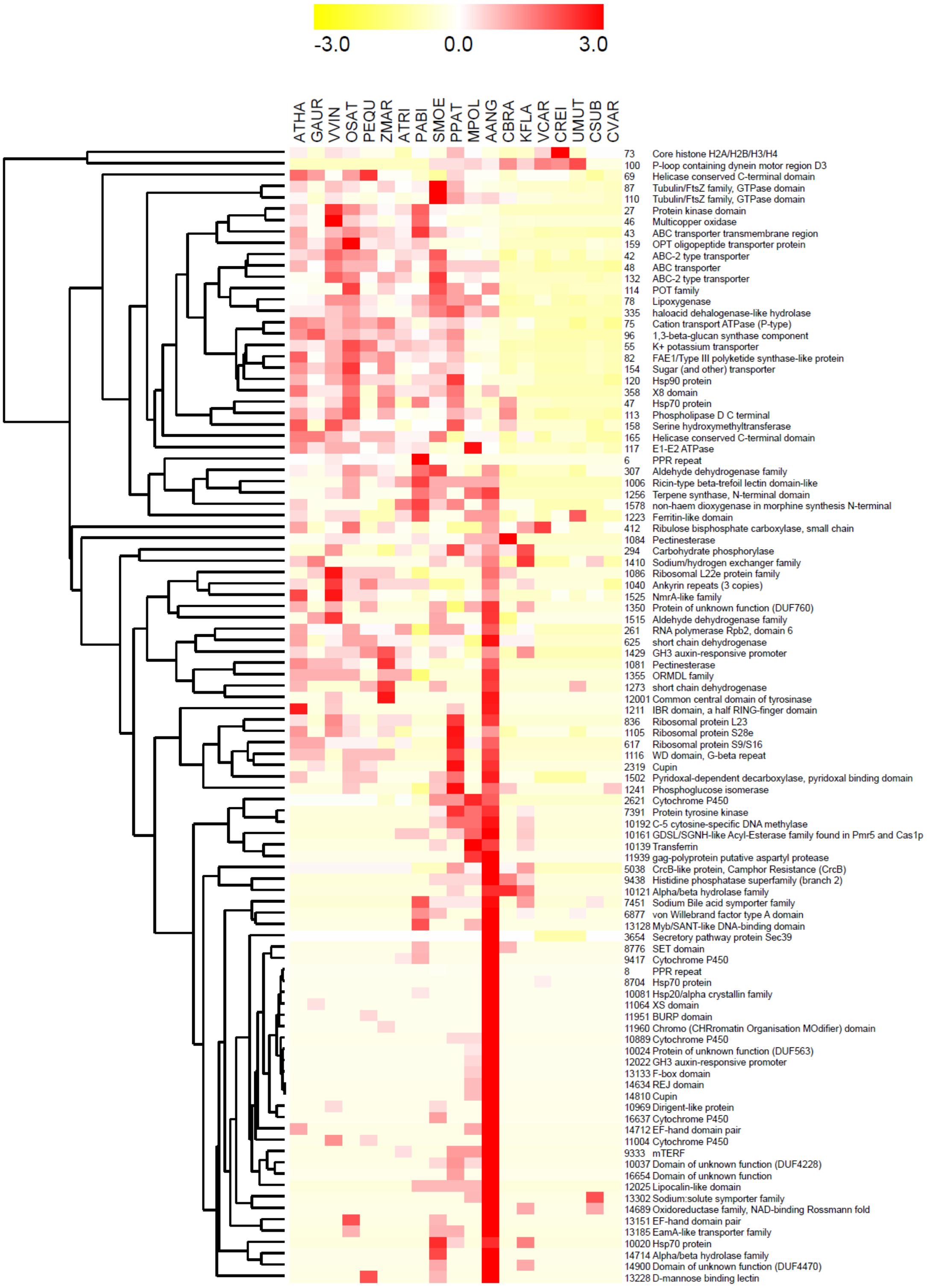

Supplementary Figure 55. Hierachical clustering of the 100 largest *Anthoceros angustus* gene families. For each gene family in each species the z-score was calculated to indicate the degree of family expansion/contraction and the resultant z-score matrix was clustered using Pearson correlation as a distance measure. The yellow and red scale (based on z-scores) show where gene family sizes are substantially smaller or larger than the mean gene family size. Red blocks represent gene family expansions, and yellow blocks represent gene family contraction. On the

right side, the gene family IDs are shown followed by the corresponding functional description. ATHA = *Arabidopsis thaliana*; GAUR = *Genlisea aurea*; VVIN = *Vitis vinifera*; OSAT = *Oryza sativa*; PEQU = *Phalaenopsis equestris*; ZMAR = *Zostera marina*; ATRI = *Amborella trichopoda*; PABI = *Picea abies*; SMOE = *Selaginella moellendorffii*; PPAT = *Physcomitrella patens*; MPOL = *Marchantia polymorpha*; AANG = *Anthoceros angustus*; CBRA = *Chara braunii*; KNIT = *Klebsormidium nitens*; VCAR = *Volvox carteri*; CREI = *Chlamydomonas reinhardtii*; UMUT = *Ulva mutabilis*; CSUB = *Coccomyxa subellipsoidea*; CVAR = *Chlorella variabilis*.

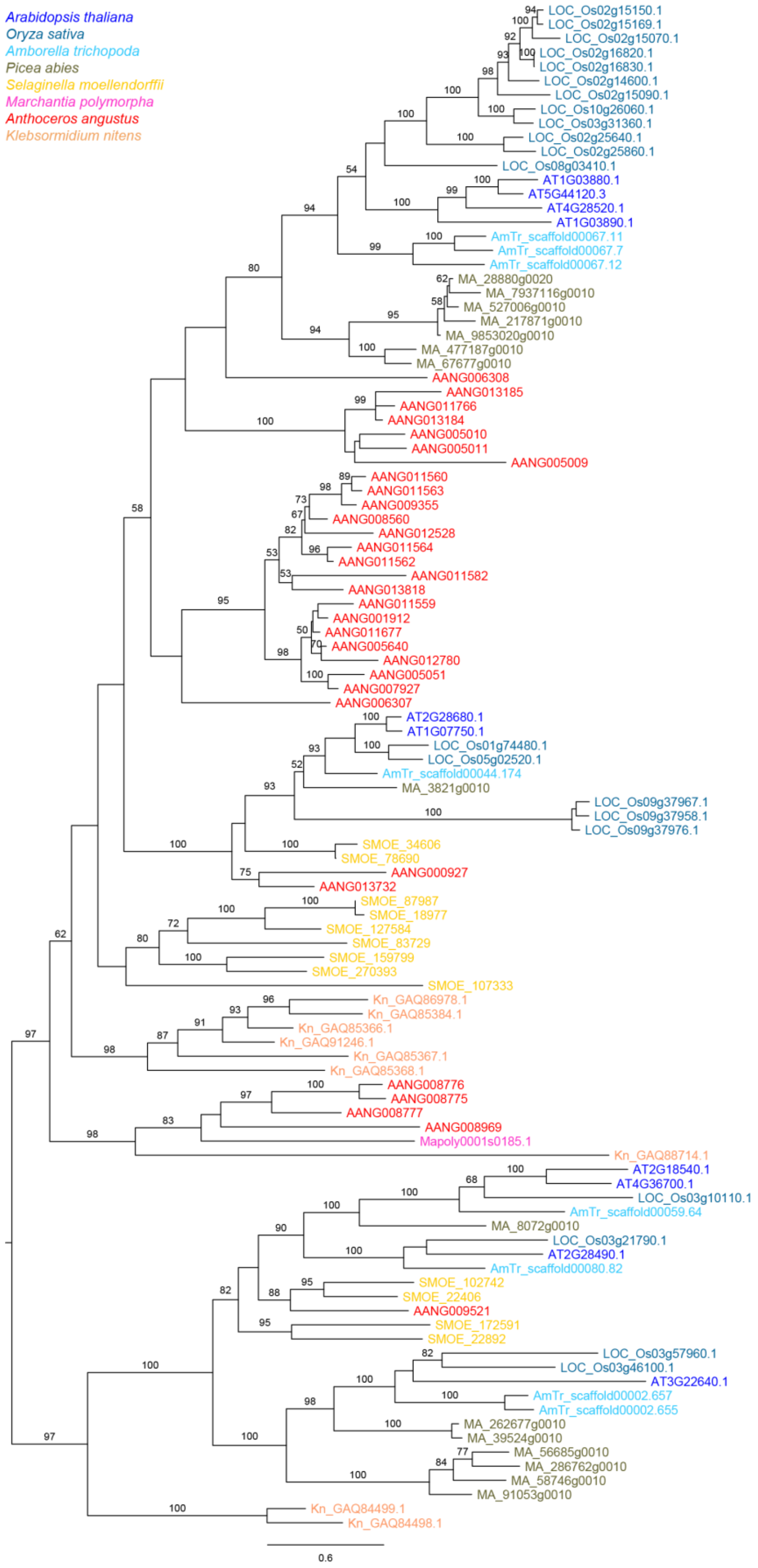

Supplementary Figure 56. Phylogeny of bicupin proteins from land plants and green algae. Bootstrap support values ≥ 50% are shown above the branches.

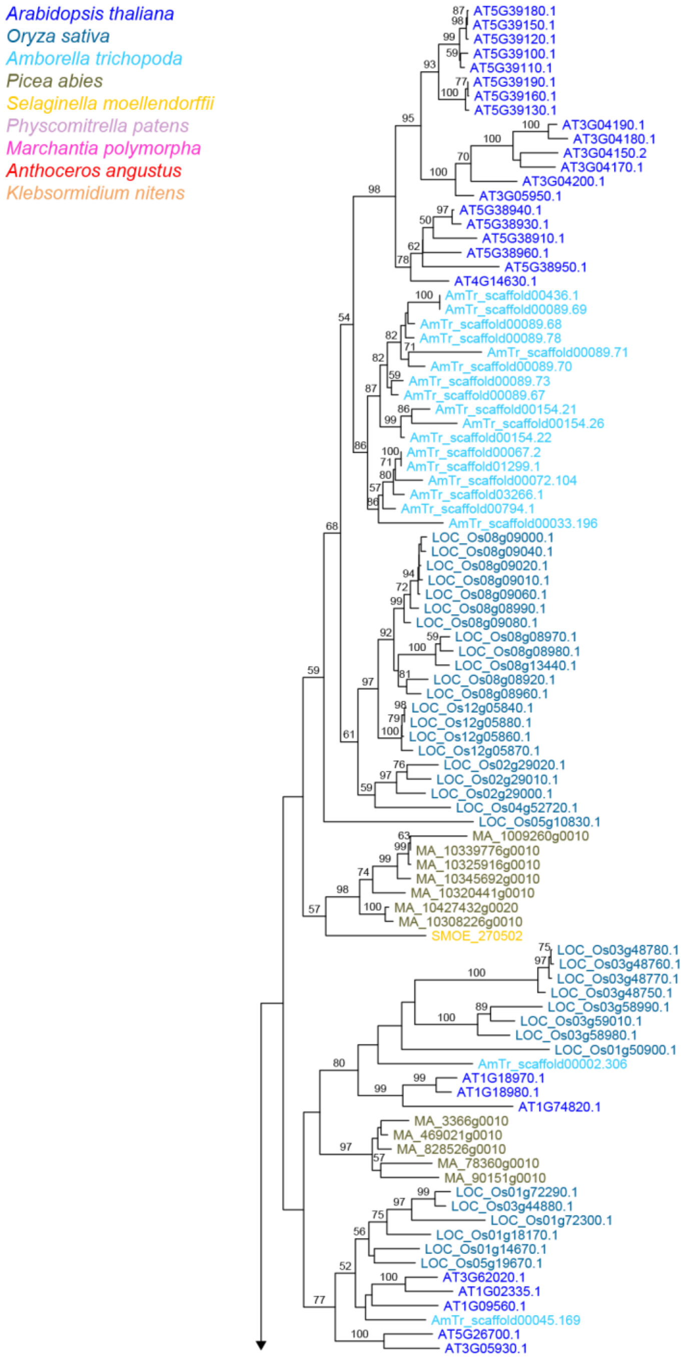
Arabidopsis thaliana
Oryza sativa
Amborella trichopoda
Picea abies
Selaginella moellendorffii
Physcomitrella patens
Marchantia polymorpha
Anthoceros angustus
Klebsormidium nitens
AT5G39180.1
AT5G39150.1
AT5G39120.1
AT5G39100.1
AT5G39110.1
AT5G39190.1
AT5G39160.1
AT5G39130.1
AT3G04190.1
AT3G04180.1
AT3G04150.2
AT3G04170.1
AT3G04200.1
AT3G05950.1
AT5G38940.1
AT5G38930.1
AT5G38910.1
AT5G38960.1
AT5G38950.1
AT4G14630.1
AmTr_scaffold00436.1
AmTr_scaffold00089.69
AmTr_scaffold00089.68
AmTr_scaffold00089.78
AmTr_scaffold00089.71
AmTr_scaffold00089.70
AmTr_scaffold00089.73
AmTr_scaffold00089.67
AmTr_scaffold00154.21
AmTr_scaffold00154.26
AmTr_scaffold00154.22
AmTr_scaffold00067.2
AmTr_scaffold01299.1
AmTr_scaffold00072.104
AmTr_scaffold03266.1
AmTr_scaffold00794.1
AmTr_scaffold00033.196
LOC_Os08g09000.1
LOC_Os08g09040.1
LOC_Os08g09020.1
LOC_Os08g09010.1
LOC_Os08g09060.1
LOC_Os08g08990.1
LOC_Os08g09080.1
LOC_Os08g08970.1
LOC_Os08g08980.1
LOC_Os08g13440.1
LOC_Os08g08920.1
LOC_Os08g08960.1
LOC_Os12g05840.1
LOC_Os12g05880.1
LOC_Os12g05860.1
LOC_Os12g05870.1
LOC_Os02g29020.1
LOC_Os02g29010.1
LOC_Os02g29000.1
LOC_Os04g52720.1
LOC_Os05g10830.1
MA_1009260g0010
MA_10339776g0010
MA_10325916g0010
MA_10345692g0010
MA_10320441g0010
MA_10427432g0020
MA_10308226g0010
SMOE_270502
LOC_Os03g48780.1
LOC_Os03g48760.1
LOC_Os03g48770.1
LOC_Os03g48750.1
LOC_Os03g58990.1
LOC_Os03g59010.1
LOC_Os03g58980.1
LOC_Os01g50900.1
AmTr_scaffold00002.306
AT1G18970.1
AT1G18980.1
AT1G74820.1
MA_3366g0010
MA_469021g0010
MA_828526g0010
MA_78360g0010
MA_90151g0010
LOC_Os01g72290.1
LOC_Os03g44880.1
LOC_Os01g72300.1
LOC_Os01g18170.1
LOC_Os01g14670.1
LOC_Os05g19670.1
AT3G62020.1
AT1G02335.1
AT1G09560.1
AmTr_scaffold00045.169
AT5G26700.1
AT3G05930.1

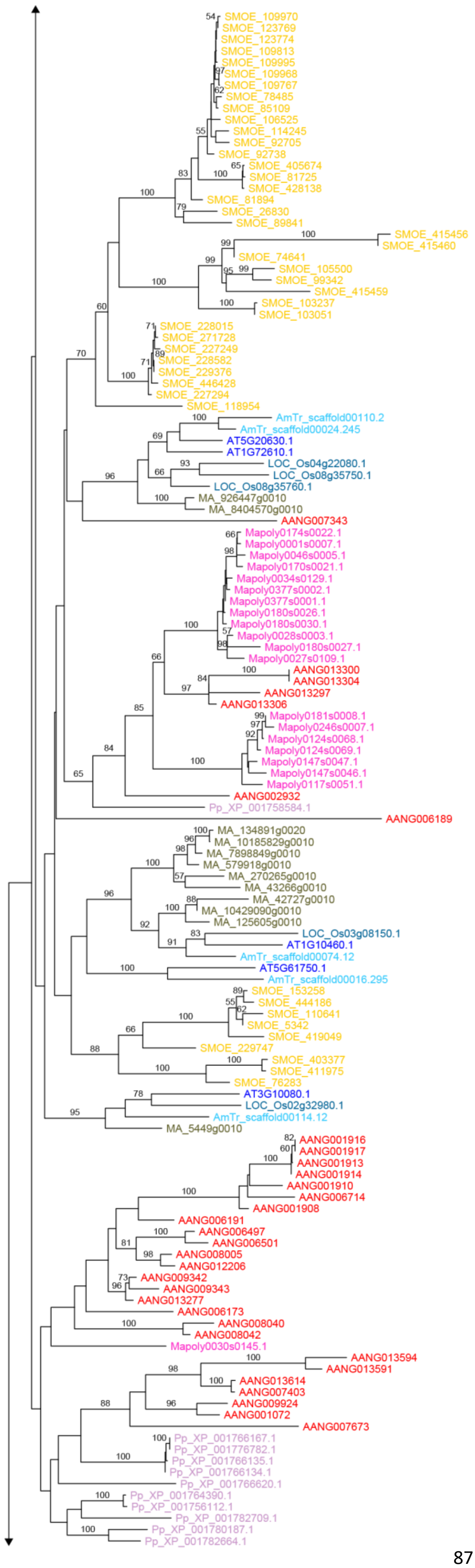
SMOE_109970
SMOE_123769
SMOE_123774
SMOE_109813
SMOE_109995
SMOE_109968
SMOE_109767
SMOE_78485
SMOE_85109
SMOE_106525
SMOE_114245
SMOE_92705
SMOE_92738
SMOE_405674
SMOE_81725
SMOE_428138
SMOE_81894
SMOE_26830
SMOE_89841
SMOE_415456
SMOE_415460
SMOE_74641
SMOE_105500
SMOE_99342
SMOE_415459
SMOE_103237
SMOE_103051
SMOE_228015
SMOE_271728
SMOE_227249
SMOE_228582
SMOE_229376
SMOE_446428
SMOE_227294
SMOE_118954
AmTr_scaffold00110.2
AmTr_scaffold00024.245
AT5G20630.1
AT1G72610.1
LOC_Os04g22080.1
LOC_Os08g35750.1
LOC_Os08g35760.1
MA_926447g0010
MA_8404570g0010
AANG007343
Mapoly0174s0022.1
Mapoly0001s0007.1
Mapoly0046s0005.1
Mapoly0170s0021.1
Mapoly0034s0129.1
Mapoly0377s0002.1
Mapoly0377s0001.1
Mapoly0180s0026.1
Mapoly0180s0030.1
Mapoly0028s0003.1
Mapoly0180s0027.1
Mapoly0027s0109.1
AANG013300
AANG013304
AANG013297
AANG013306
Mapoly0181s0008.1
Mapoly0246s0007.1
Mapoly0124s0068.1
Mapoly0124s0069.1
Mapoly0147s0047.1
Mapoly0147s0046.1
Mapoly0117s0051.1
AANG002932
Pp_XP_001758584.1
AANG006189
MA_134891g0020
MA_10185829g0010
MA_7898849g0010
MA_579918g0010
MA_270265g0010
MA_43266g0010
MA_42727g0010
MA_10429090g0010
MA_125605g0010
LOC_Os03g08150.1
AT1G10460.1
AmTr_scaffold00074.12
AT5G61750.1
AmTr_scaffold00016.295
SMOE_153258
SMOE_444186
SMOE_110641
SMOE_5342
SMOE_419049
SMOE_229747
SMOE_403377
SMOE_411975
SMOE_76283
AT3G10080.1
LOC_Os02g32980.1
AmTr_scaffold00114.12
MA_5449g0010
AANG001916
AANG001917
AANG001913
AANG001914
AANG001910
AANG006714
AANG001908
AANG006191
AANG006497
AANG006501
AANG008005
AANG012206
AANG009342
AANG009343
AANG013277
AANG006173
AANG008040
AANG008042
Mapoly0030s0145.1
AANG013594
AANG013591
AANG013614
AANG007403
AANG009924
AANG001072
AANG007673
Pp_XP_001766167.1
Pp_XP_001776782.1
Pp_XP_001766135.1
Pp_XP_001766134.1
Pp_XP_001766620.1
Pp_XP_001764390.1
Pp_XP_001756112.1
Pp_XP_001782709.1
Pp_XP_001780187.1
Pp_XP_001782664.1

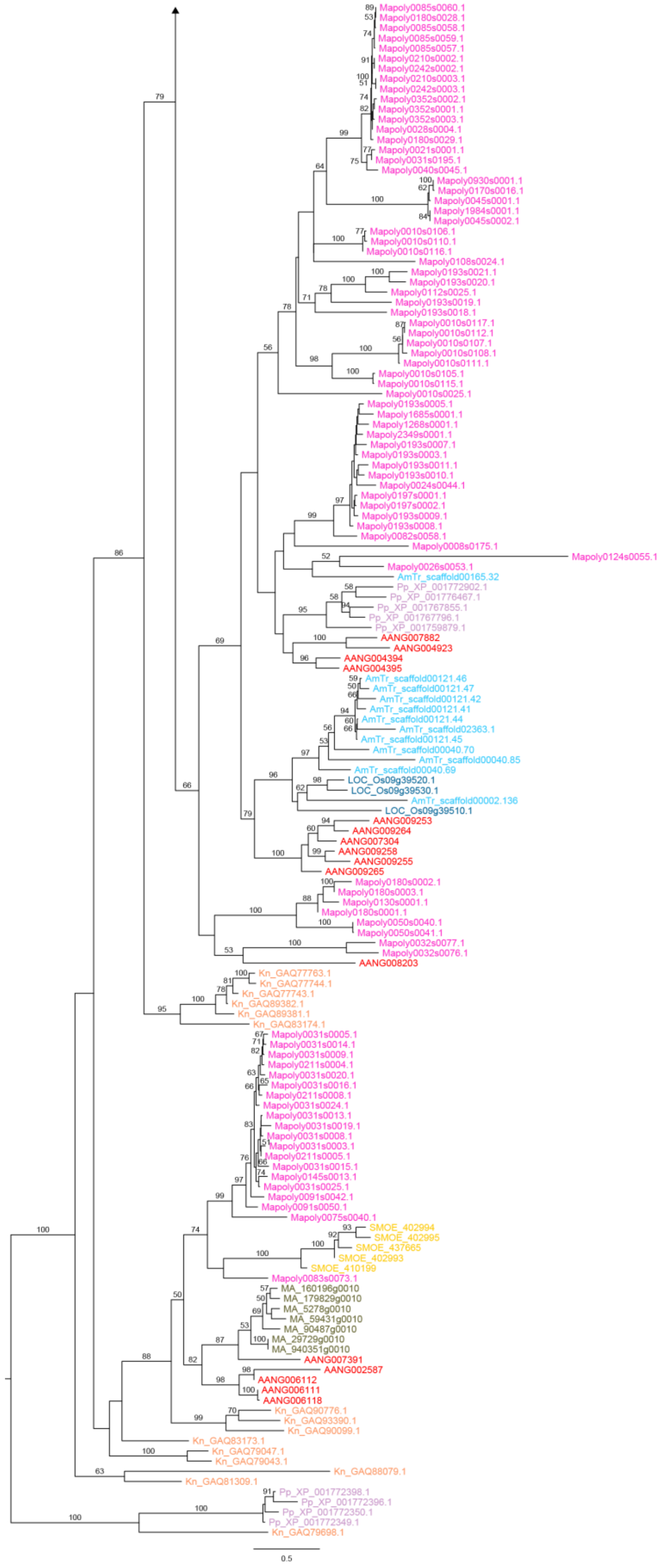

Supplementary Figure 57. Phylogeny of monocupin proteins from land plants and green algae. Bootstrap support values ≥ 50% are shown above the branches.

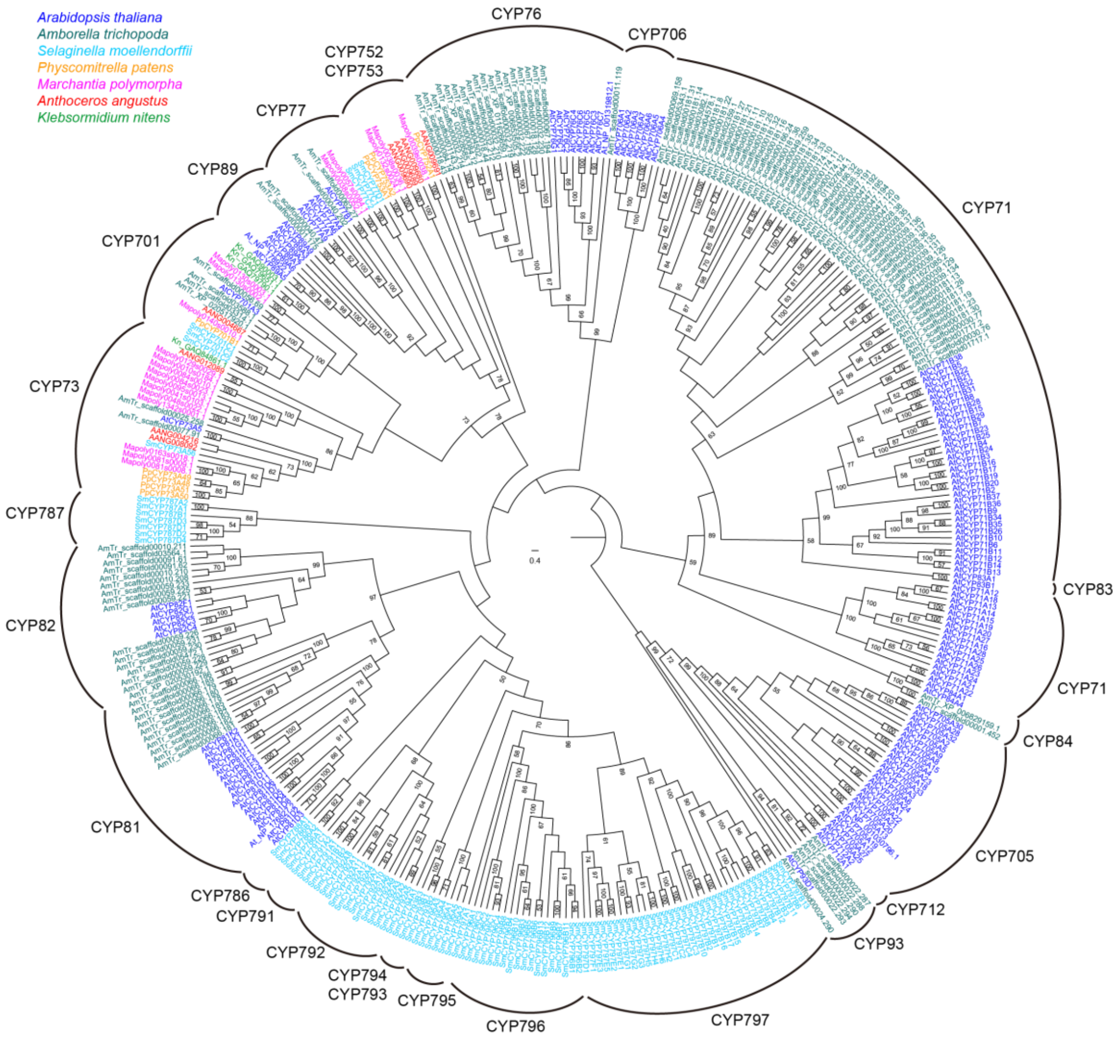

Supplementary Figure 58. Phylogeny (part I) of CYP71 clan protein sequences from land plants and green algae. Bootstrap support values ≥ 50% are shown above the branches.

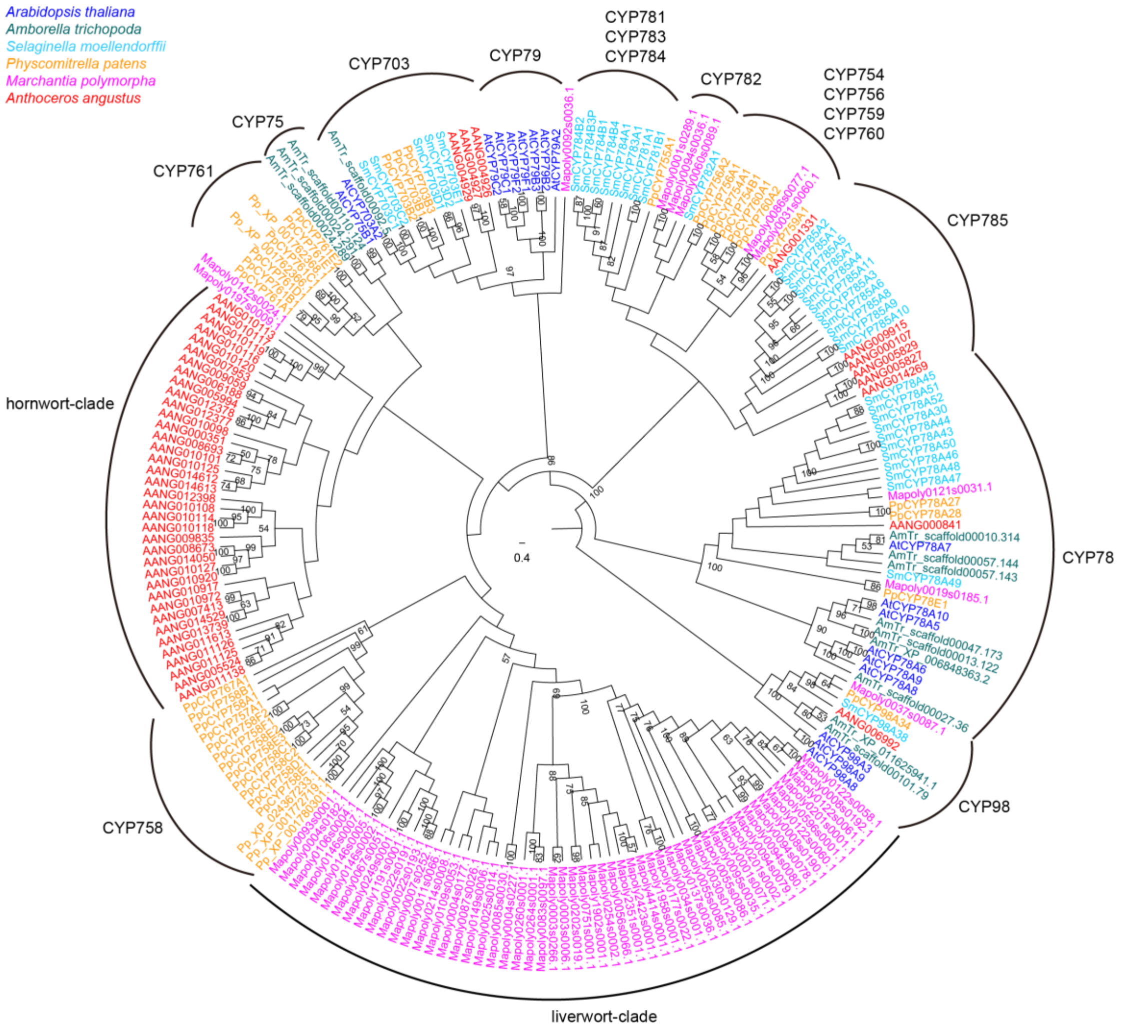

Supplementary Figure 59. Phylogeny (part II) of CYP71 clan protein sequences from land plants and green algae. Bootstrap support values ≥ 50% are shown above the branches.

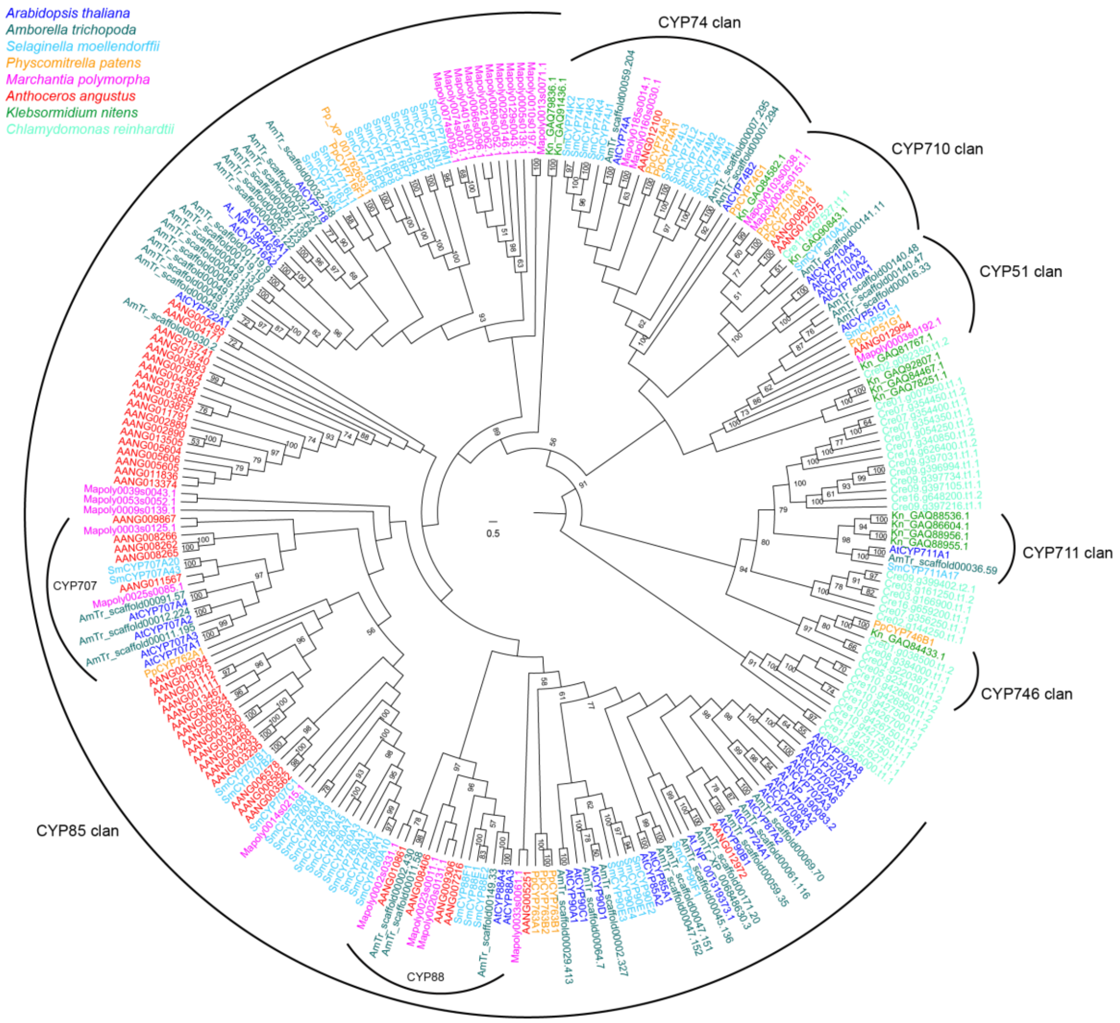

Supplementary Figure 60. Phylogeny (part I) of non-CYP71 clan protein sequences from land plants and green algae. Bootstrap support values ≥ 50% are shown above the branches.

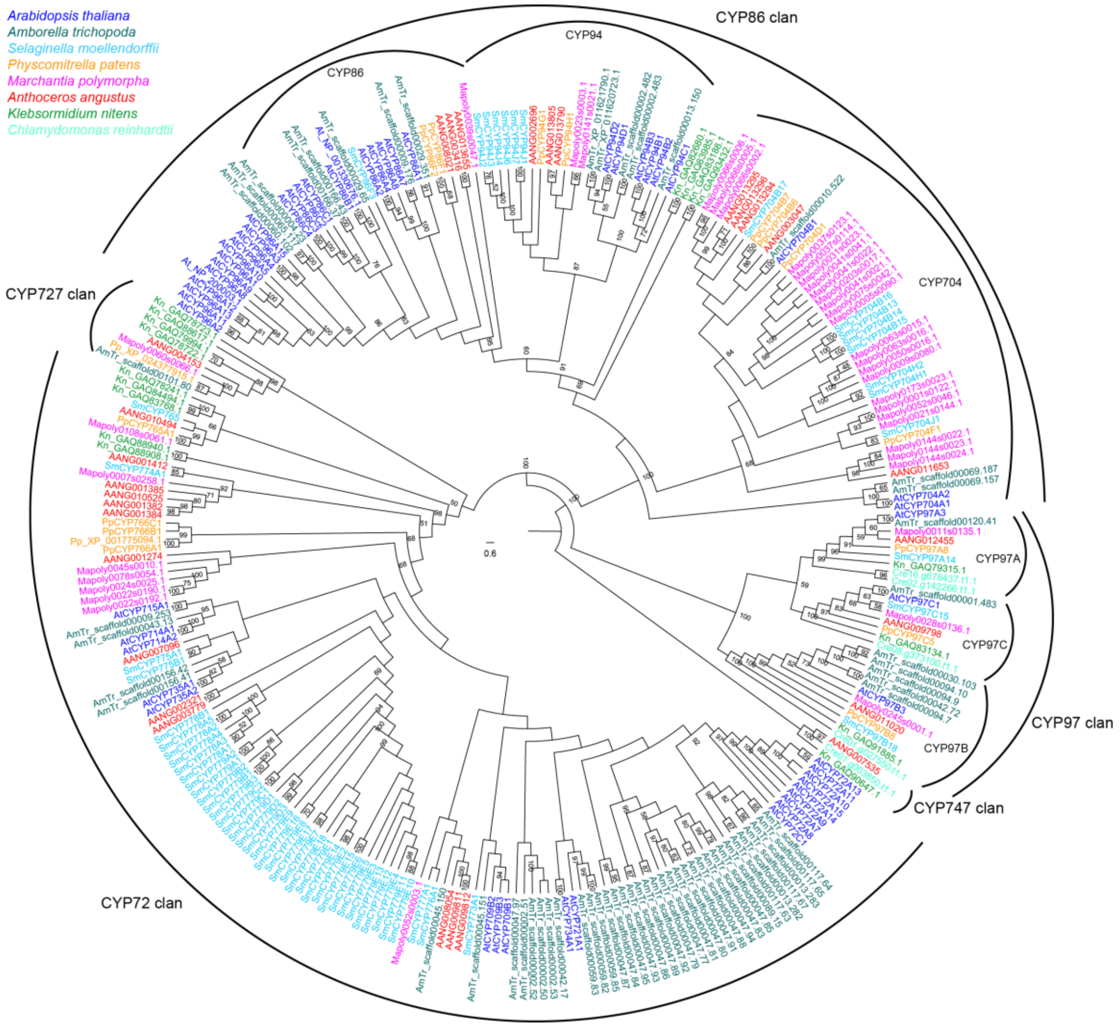

Supplementary Figure 61. Phylogeny (part II) of non-CYP71 clan protein sequences from land plants and green algae. Bootstrap support values ≥ 50% are shown above the branches.

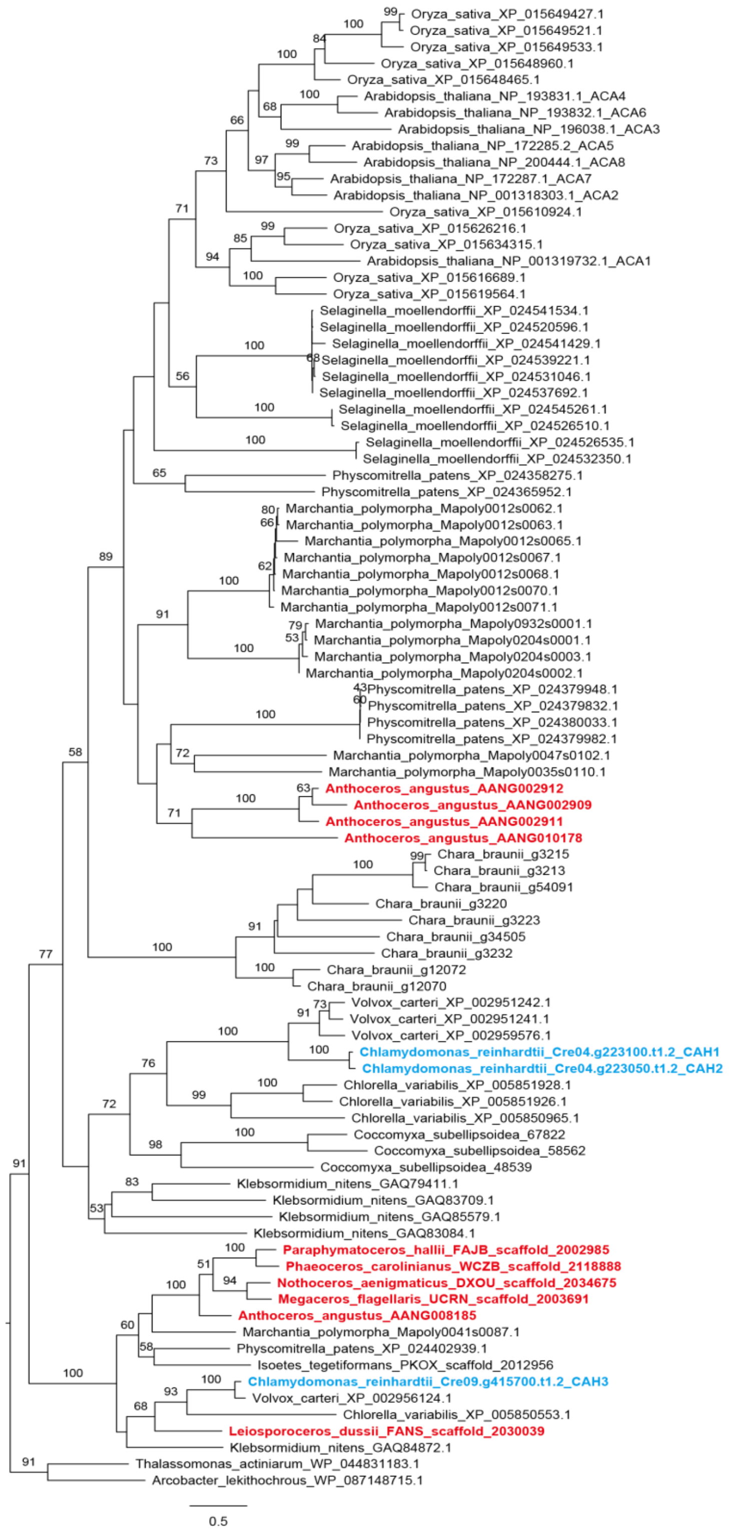

Supplementary Figure 62. Phylogeny of alpha carbonic anhydrases (α CAs) from land plants and green algae. The two homologous sequences (WP_044831183.1 and WP_087148715.1) from bacteia were used as outgroup. The ingroup sequences were retrieved via a reciprocal best BLAST hit with the *Chlamydomonas* CAH1-3 sequences as initial queries. Bootstrap support values ≥ 50% are shown above the branches. The phylogenetic tree shows that the CAH1/2 homologs formed a group including members from all investigated green plants, and the CAH3 homologs formed a distinct group only with members from non-angiosperm species.

Four CAH1/2-like sequences (AANG002912, AANG002909, AANG002911, and AANG010178) and one CAH3-like squence (AANG008185) occur in the *Anthoceros angustus* genome. The CAH1-3 sequences of *Chlamydomonas reinhardtii* are in blue and the homologs from hornworts are in red.

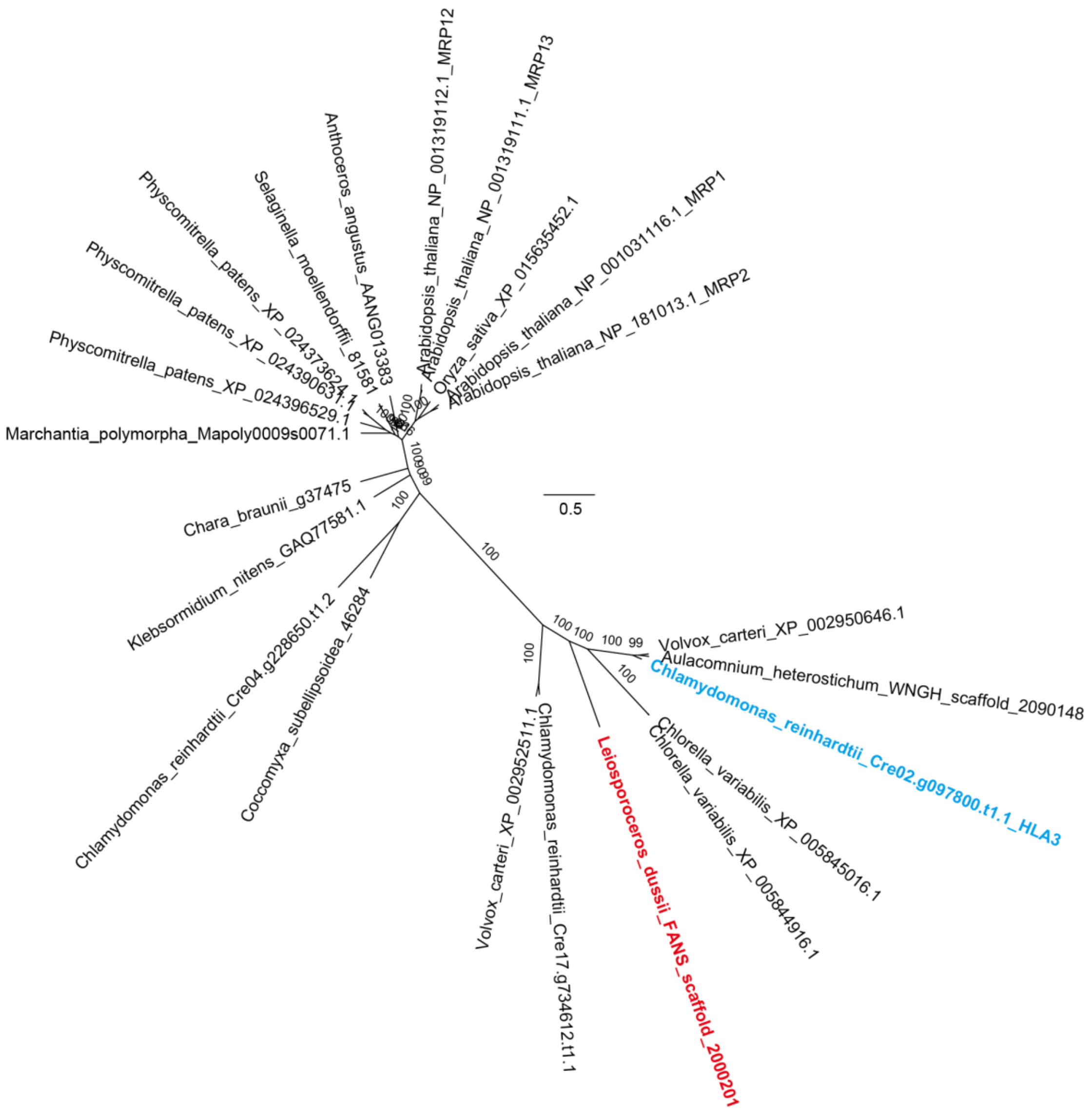

Supplementary Figure 63. Phylogeny of multidrug-resistance–related proteins (MRPs) from land plants and green algae. The *Chlamydomonas* HLA3 (high light activated gene 3) belongs to the multidrug-resistance–related protein (MRP) subfamily. This sequence is used as an initial query to retrieve the homologous sequences from green plants. Bootstrap support values ≥ 50% are shown above the branches. The phylogenetic tree shows that no MRP proteins from *Anthoceros angustus* clustered with members of the group including HLA3, whereas one MRP sequence from the hornwort *Leiosporoceros dussii* and one from the moss *Aulacomnium heterostichum* are resolved in this HLA3 group. The HLA3 sequence of *Chlamydomonas reinhardtii* is in blue and the HLA3-like sequence from the hornwort species are in red.

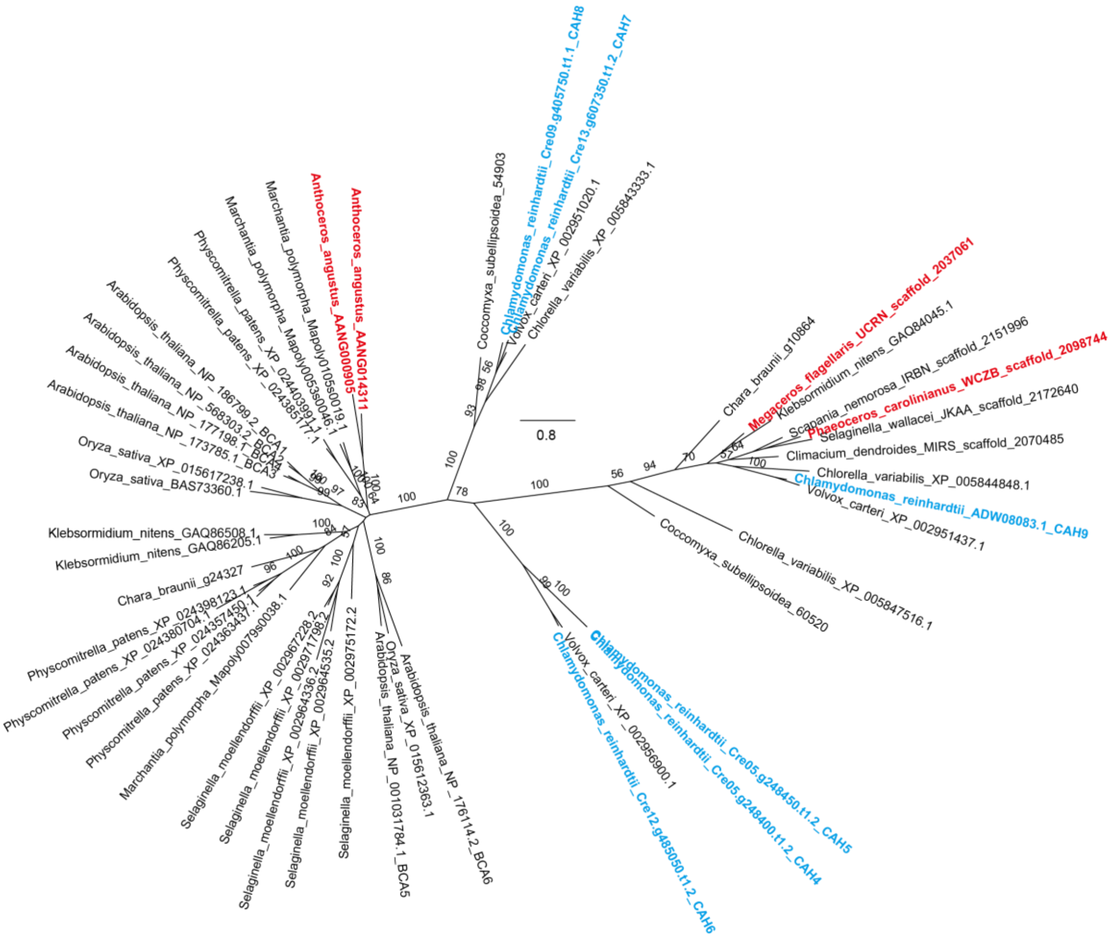

Supplementary Figure 64. Phylogeny of beta carbonic anhydrases (β-CAs) from land plants and green algae. The sequences were retrieved via a reciprocal best BLAST hit with the *Chlamydomonas* CAH4-9 sequences as initial queries. Bootstrap support values ≥ 50% are shown above the branches. The phylogenetic tree shows that most land plant β-CAs are closely related to CAH7/8, no land plant β-CAs are clustered with CHA4-6, and CAH9 homologs formed a distinct group only with members from non-angiosperm species. Two CAH7/8-like sequences (AANG000905 and AANG014311) and no CAH4-6 like or CAH9-like sequences were found in the *Anthoceros angustus* genome. The CAH4-9 sequences of *Chlamydomonas reinhardtii* are in blue and the homologs from the hornworts are in red.

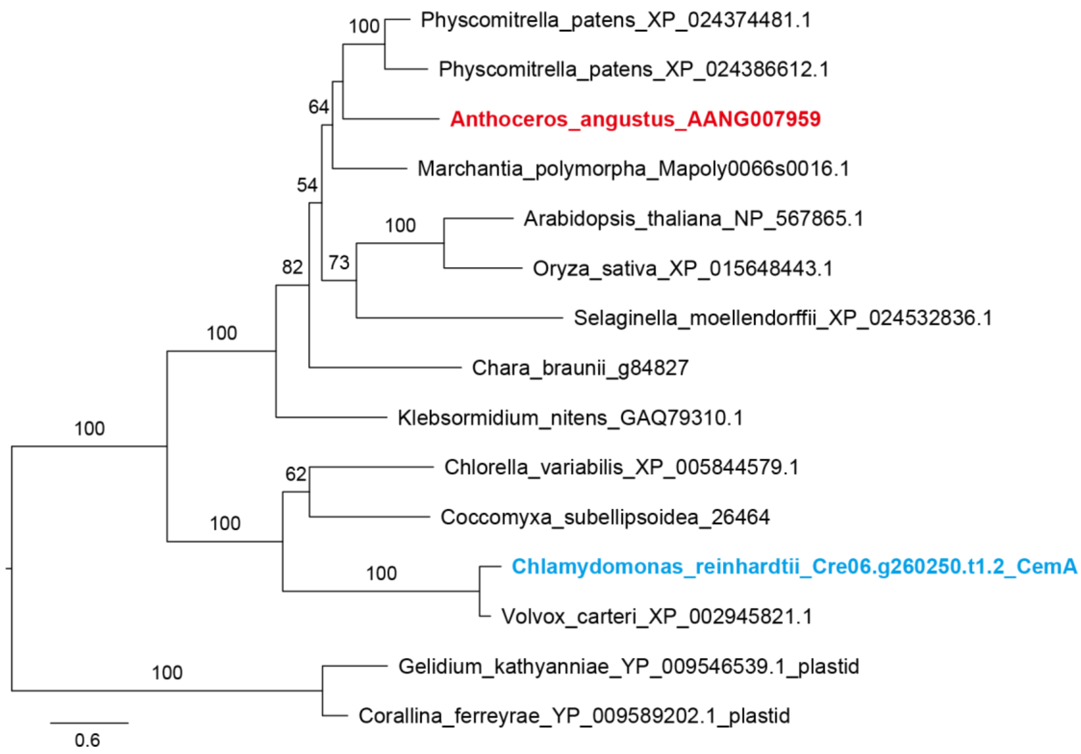

Supplementary Figure 65. Phylogeny of chloroplast envelope membrane protein-like (CemA-like) proteins from land plants and green algae. The two homologous sequences (YP_009546539.1 and YP_009589202.1) from plastid genomes were used as outgroup. The ingroup sequences were retrieved via a reciprocal best BLAST hit with the *Chlamydomonas* CemA sequence as an initial query. Bootstrap support values ≥ 50% are shown above the branches. There is one CemA-like sequence (AANG007959) in the *Anthoceros angustus* genome. The CemA sequence of *Chlamydomonas reinhardtii* is in blue and the homolog from the hornwort species is in red.

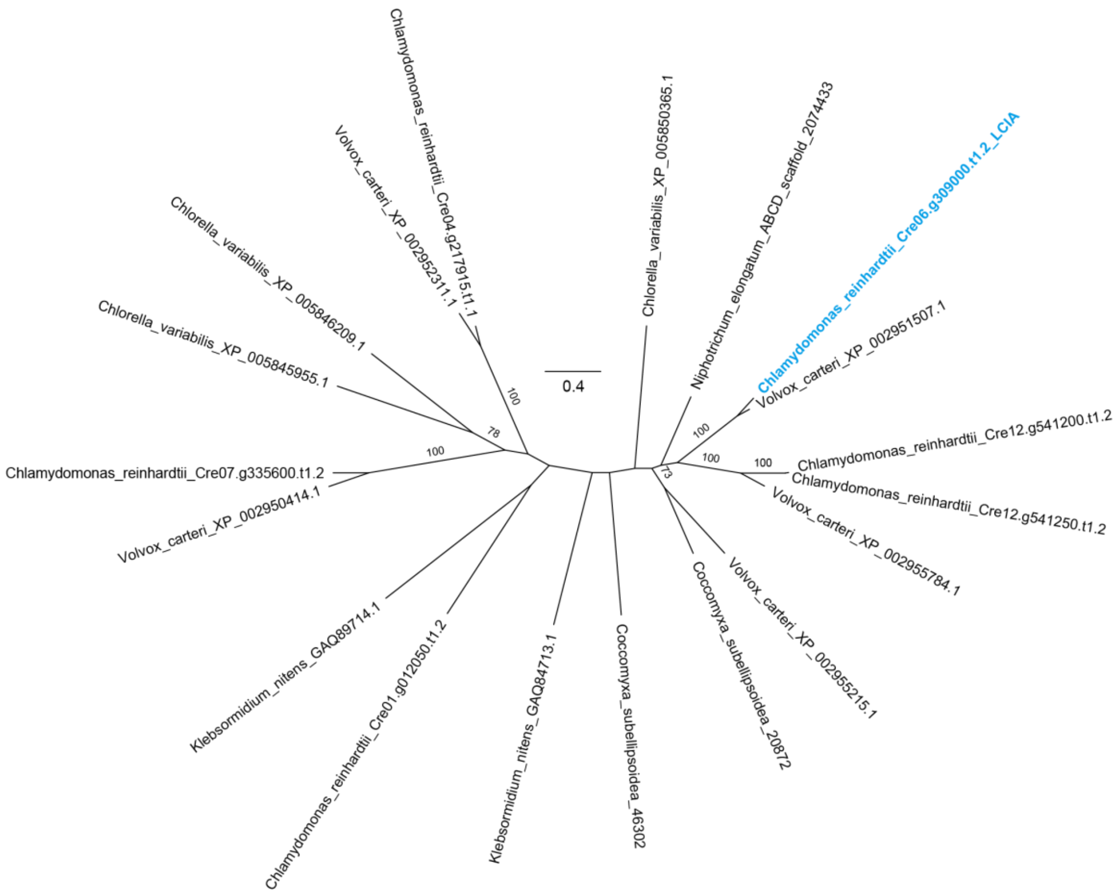

Supplementary Figure 66. Phylogeny of homologs of nitrite transporter NAR1 from land plants and green algae. The *Chlamydomonas* LCIA (low CO_2 inducible gene A) is also named as nitrate transporter NAR1, and it belongs to the formate/nitrite transporter family. This sequence is used as an initial query to retrieve the homologous sequences from green plants. Bootstrap support values ≥ 50% are shown above the branches. No LCIA-like sequences were found in the *Anthoceros angustus* genome, while one LCIA-like sequence was found in the moss *Niphotrichum elongatum*.The LCIA sequence of *Chlamydomonas reinhardtii* is in blue.

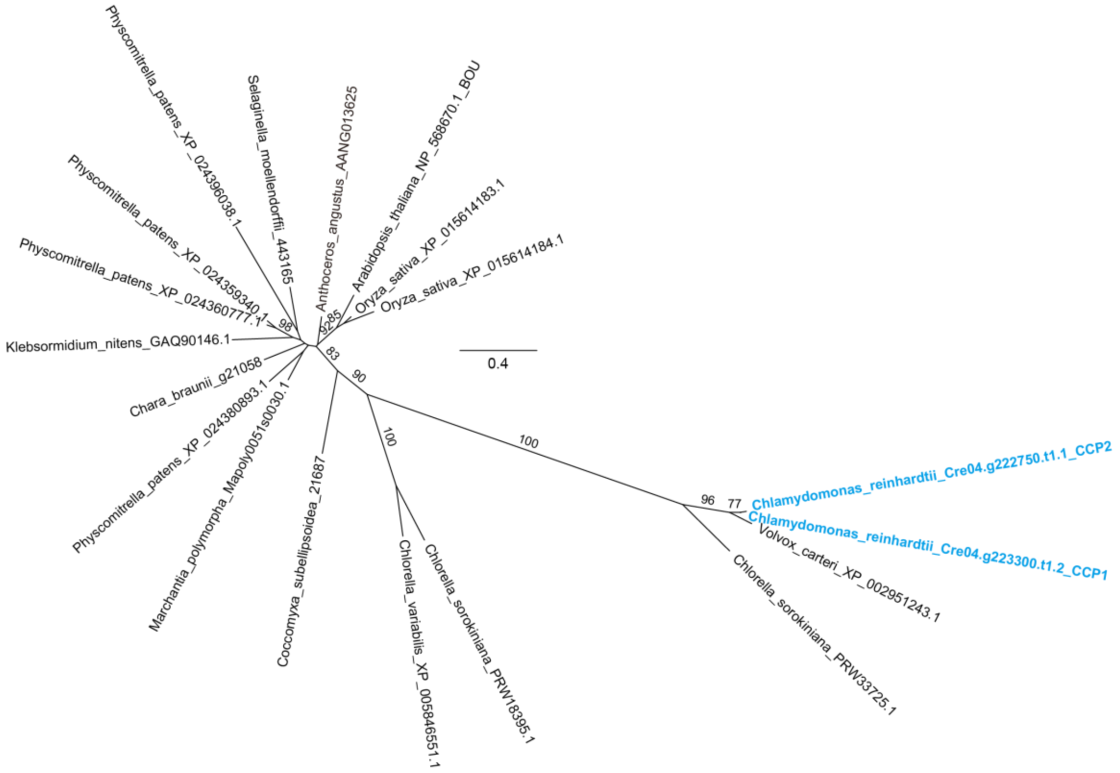

Supplementary Figure 67. Phylogeny of homologs of chloroplast carrier protein 1/2 (CCP1/2) from land plants and green algae. The *Chlamydomonas* CCP1/2 sequences are used as initial queries to retrieve the homologous sequences from green plants. Bootstrap support values ≥ 50% are shown above the branches. The chloroplast carrrier proteins of green algae are closely related to the mitochondrial carrier proteins of green plants, forming two groups. No CCP1/2-like sequences were found in the *Anthoceros angustus* genome. The CCP1/2 sequences of *Chlamydomonas reinhardtii* are in blue.

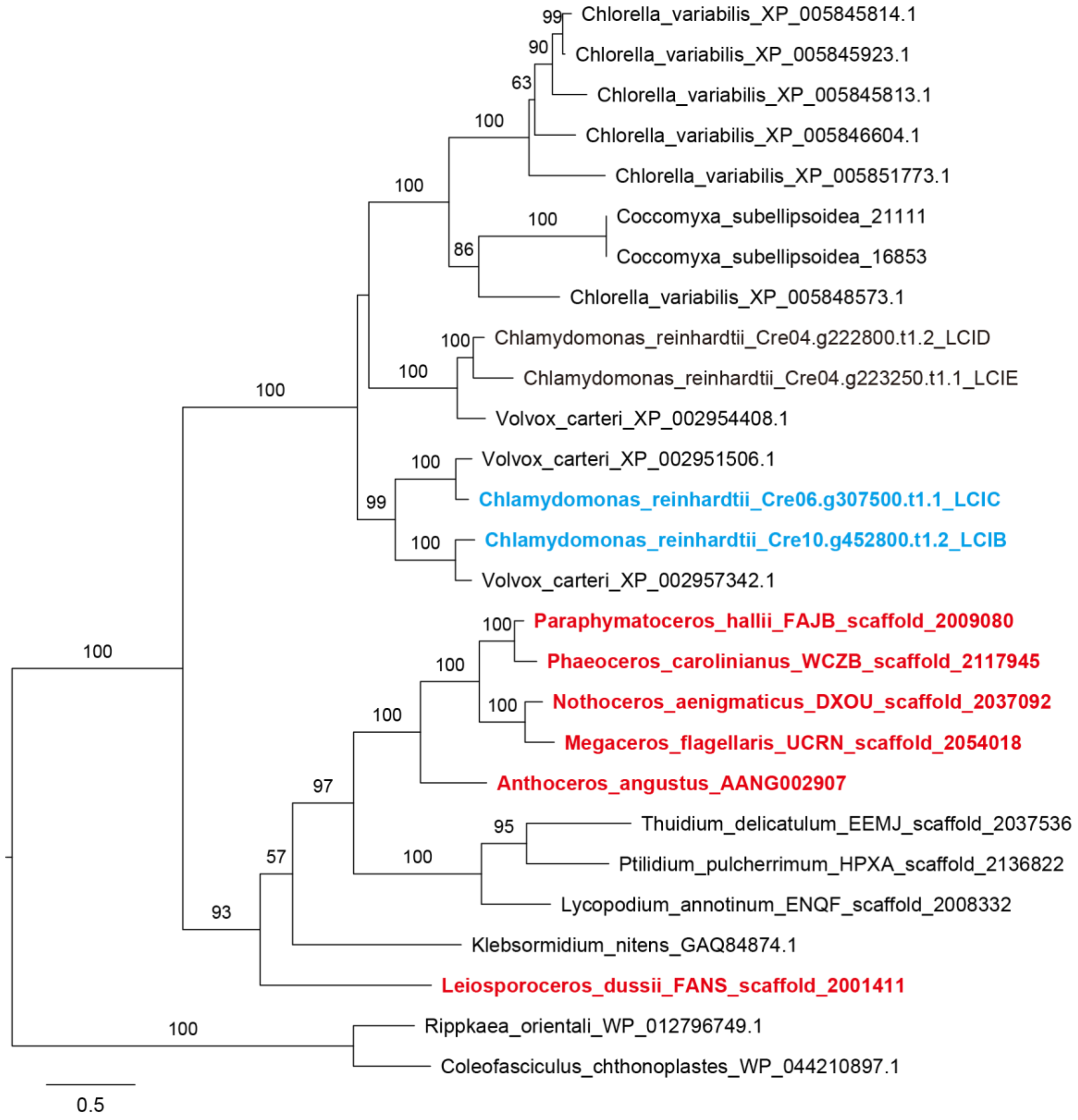

Supplementary Figure 68. Phylogeny of theta carbonic anhydrases (θ-CAs) from land plants and green algae. The two homologous sequences (WP_012796749.1 and WP_044210897.1) from bacteria were used as outgroup. The *Chlamydomonas* LCIB/C (low CO_2 inducible gene B/C) encode θ-CAs. The ingroup sequences were retrieved via a reciprocal best BLAST hit with the *Chlamydomonas* LCIB/C sequences as initial queries. Bootstrap support values ≥ 50% are shown above the branches. The LCIB/C-like sequences were only found in non-angiosperm land plants and green plants. One LCIB/C-like sequence (AANG002907) occurs in the *Anthoceros angustus* genome. The LCIB/C sequences of *Chlamydomonas reinhardtii* are in blue and the homologs from the hornwort species are in red.

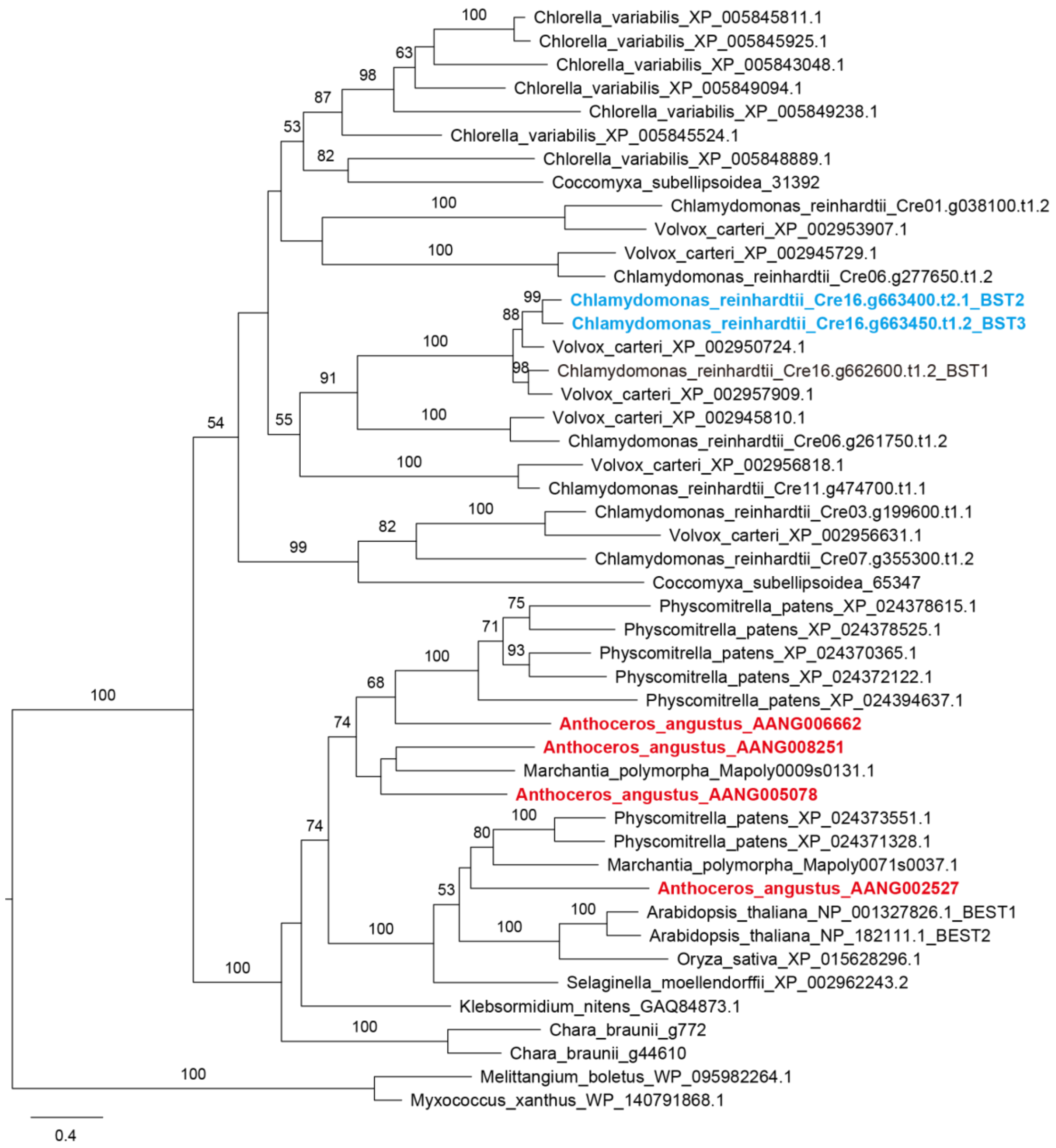

Supplementary Figure 69. Phylogeny of bestrophins (BST) from land plants and green algae. The two homologous sequences (WP_095982264.1 and WP_140791868.1) from bacteia were used as outgroup. The *Chlamydomonas* bestrophin BST2 and BST3 were previously reported as LCI11. The ingroup sequences here were retrieved via a reciprocal best BLAST hit with the *Chlamydomonas* BST2/3 sequences as initial queries. Bootstrap support values ≥ 50% are shown above the branches. There are four LCI11-like sequence (AANG006662, AANG008251, AANG005078, and AANG002527) in the *Anthoceros angustus* genome. The BST2/3 sequences of *Chlamydomonas reinhardtii* are in blue and the homologs from the hornwort species are in red.

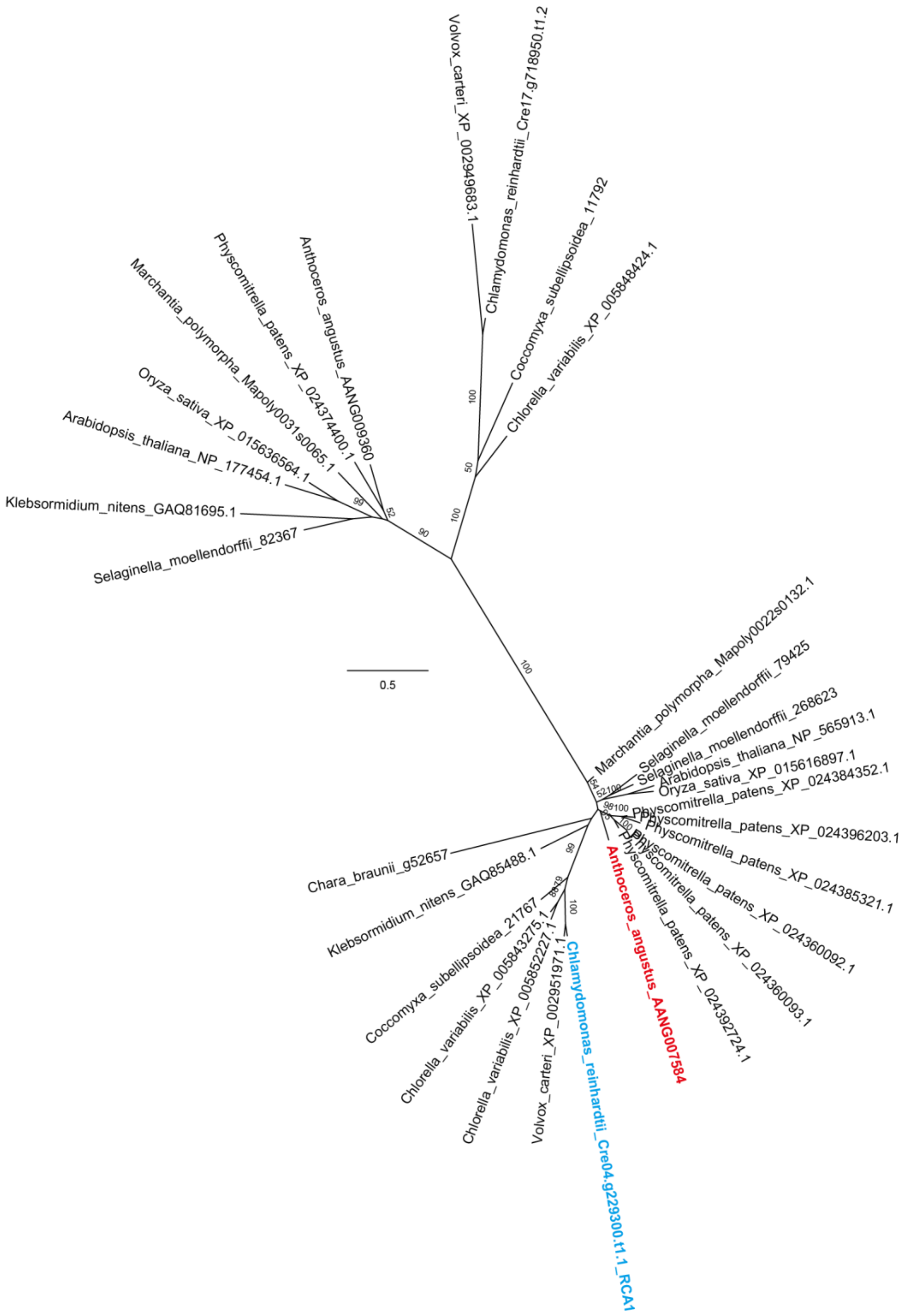

Supplementary Figure 70. Phylogeny of homologs of rubisco activase 1 (RCA1) from land plants and green algae. The *Chlamydomonas* RCA1 sequence is used as intial queries to retrieve the homologous sequences from green plants. Bootstrap support values ≥ 50% are shown above the branches. One RCA1-like sequence (AANG007584) occurs in the *Anthoceros angustus* genome. The RCA1 sequence of *Chlamydomonas reinhardtii* is in blue and the homolog from the hornwort species is in red.

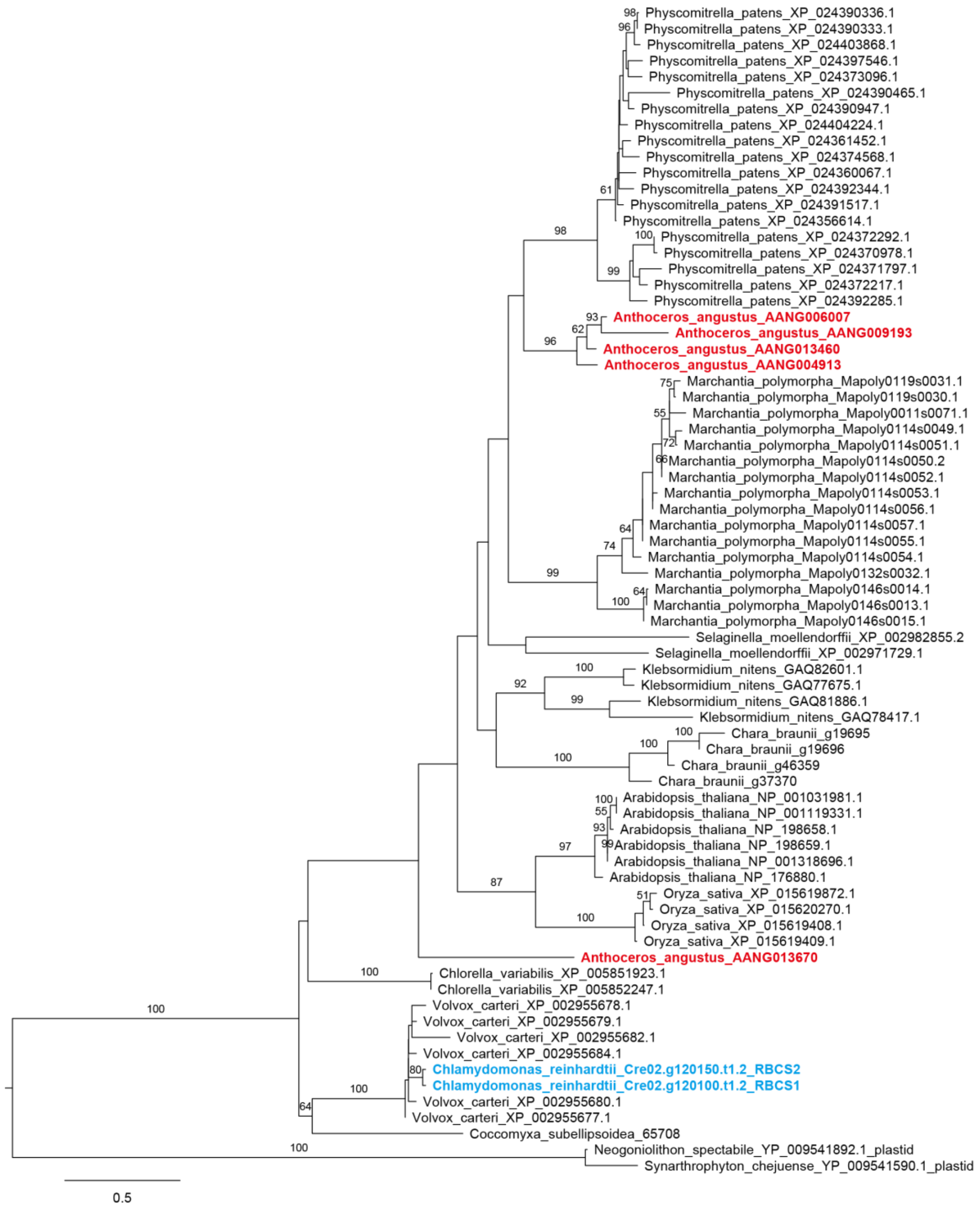

Supplementary Figure 71. Phylogeny of ribulose bisphosphate carboxylase small subunits (RBCS) from land plants and green algae. The two homologous sequences (YP_009541892.1 and YP_009541590.1) from plastid genomes were used as outgroup. The ingroup sequences were retrieved via a reciprocal best BLAST hit with the *Chlamydomonas* RBCS1/2 sequences as initial queries. Bootstrap support values ≥ 50% are shown above the branches. There are five RBCS1/2-like sequence (AANG006007, AANG009193, AANG013460, AANG004913, and AANG013670) in the *Anthoceros angustus* genome. The RBCS1/2 sequences of *Chlamydomonas reinhardtii* are in blue and the homologs from the hornwort species are in red.

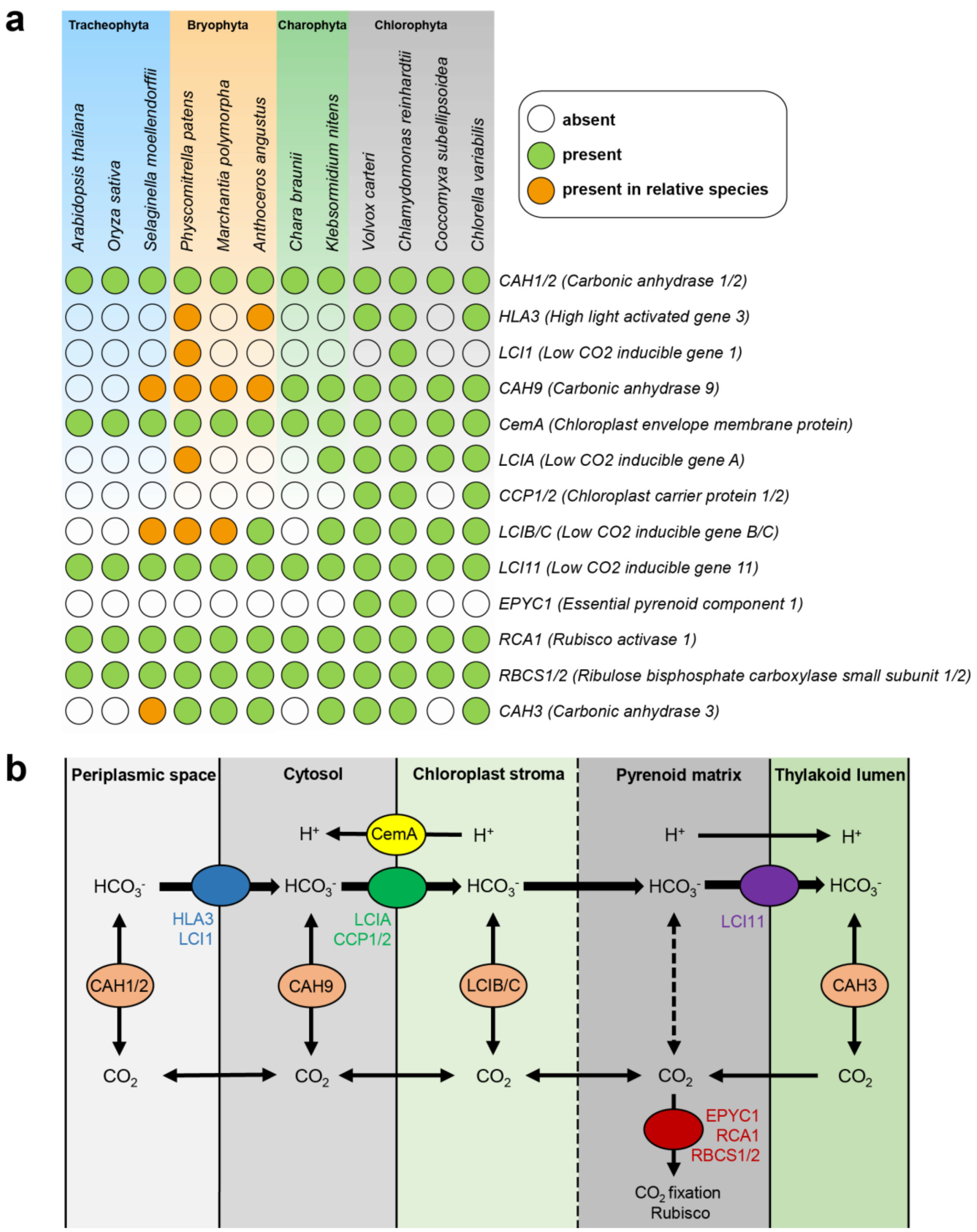

Supplementary Figure 72. Orthologs of CO_2-concentrating mechanism (CCM) genes in green plants. a, Orthologs were detected based on a BLASTP or TBLASTN search against the genome or transcriptome data of target species using *Chlamydomonas reinhardtii* sequences as queries (*E*-value < 1e-7) and further phylogenetic inferences. **b**, Simplified model of the *Chlamydomonas* CCM modified from Meyer & Griffiths (2013), Mackinder et al. (2017), and Mackinder (2018). In *C. reinharditii*, inorganic carbon is imported as HCO_3^- into the cytosol by Ci transporters HLA3 and LCI1 on the plasma membrane. HCO_3^- enters into the chloroplast stroma through the mediation by LCIA and CCP1/2 on the chloroplast envelope. CemA is a proton pump at the chloroplast envelope for maintaining the stromal pH balance, as the dehydration of bicarbonate releases hydroxyl anions. Entry of HCO_3^-into the thylakoid lumen is mediated by

LCI11 transporter on the thylakoid membrane. HCO_3^- diffuses in the pyrenoid matrix through the membrane tubules connected to the thylakoid network. In different locations, various kinds of carbonic anhydrases (orange colour) could convert HCO_3^- to CO_2, especially the luminal CAH3 and stromal LCIB-C complex that supply CO_2 available for CO_2 fixation in the pyrenoid. CO_2 is fixed by Rubisco, with the enzyme aggregated to form the pyrenoid matrix by function of EPYC1, RCA1 and RBCS1/2.

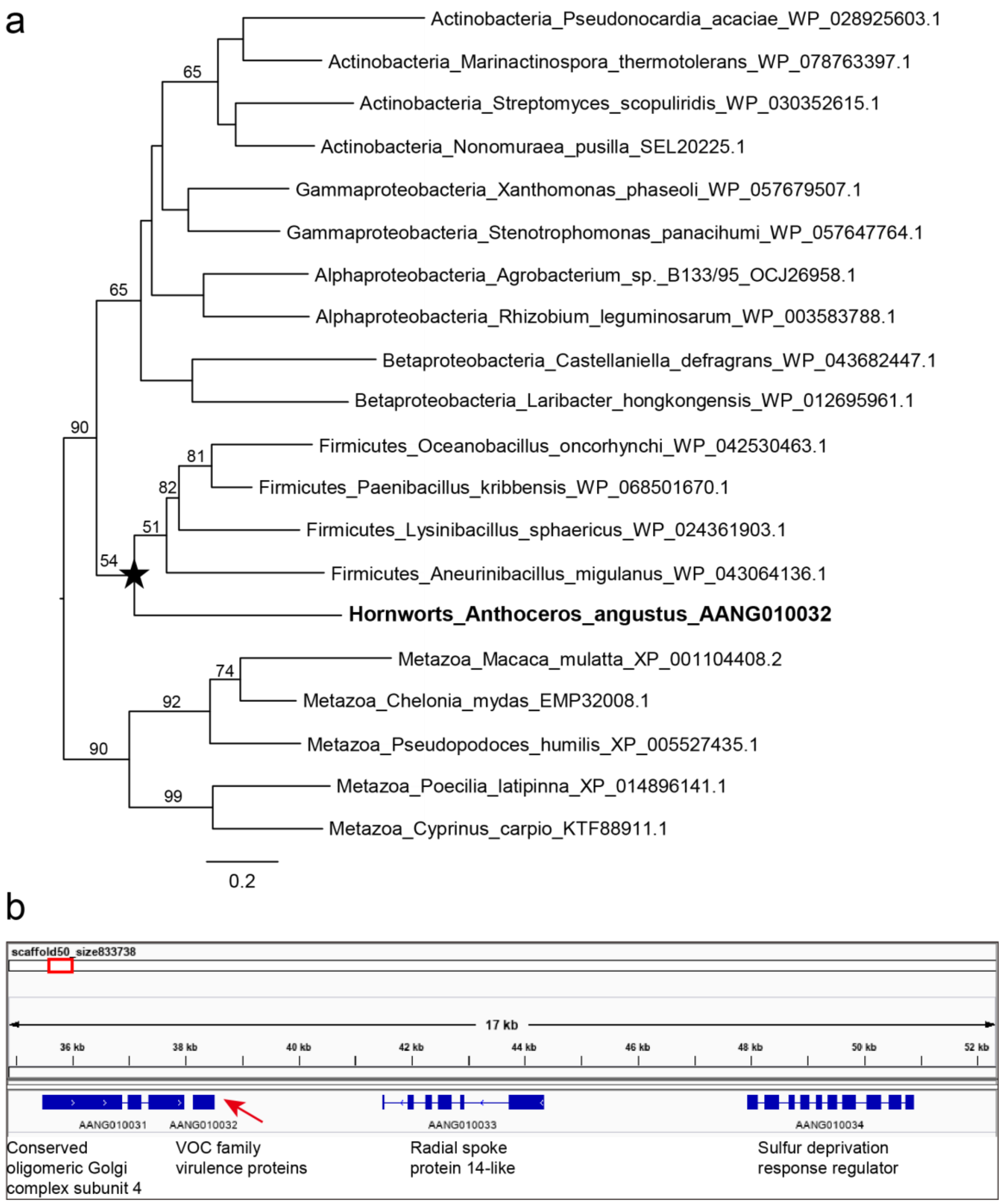

Supplementary Figure 73. Relationships of VOC family virulence proteins of *Anthoceros angustatus* and the location of their genes in the genome assembly. a, ML phylogenetic analysis of the *Anthoceros* HGT gene (AANG010032) and its homologs. Bootstrap support values ≥ 50% are shown above the branches. The metazoa homologs were used as outgroup.The asterisk indicates that the *Anthoceros* sequence (AANG010032) forms a monophyletic clade with homologs of Firmicutes, suggesting an *Anthoceros*-specific HGT event from Firmicutes. All homologous genes of the family contain the glyoxalase domain (PFAM profile PF00903). **b**, Location of the *Anthoceros* HGT gene (AANG010032) in genome assembly. The AANG010032 is intronless and located in scaffold50, with up- and down-stream genes all being annotated as plant genes.

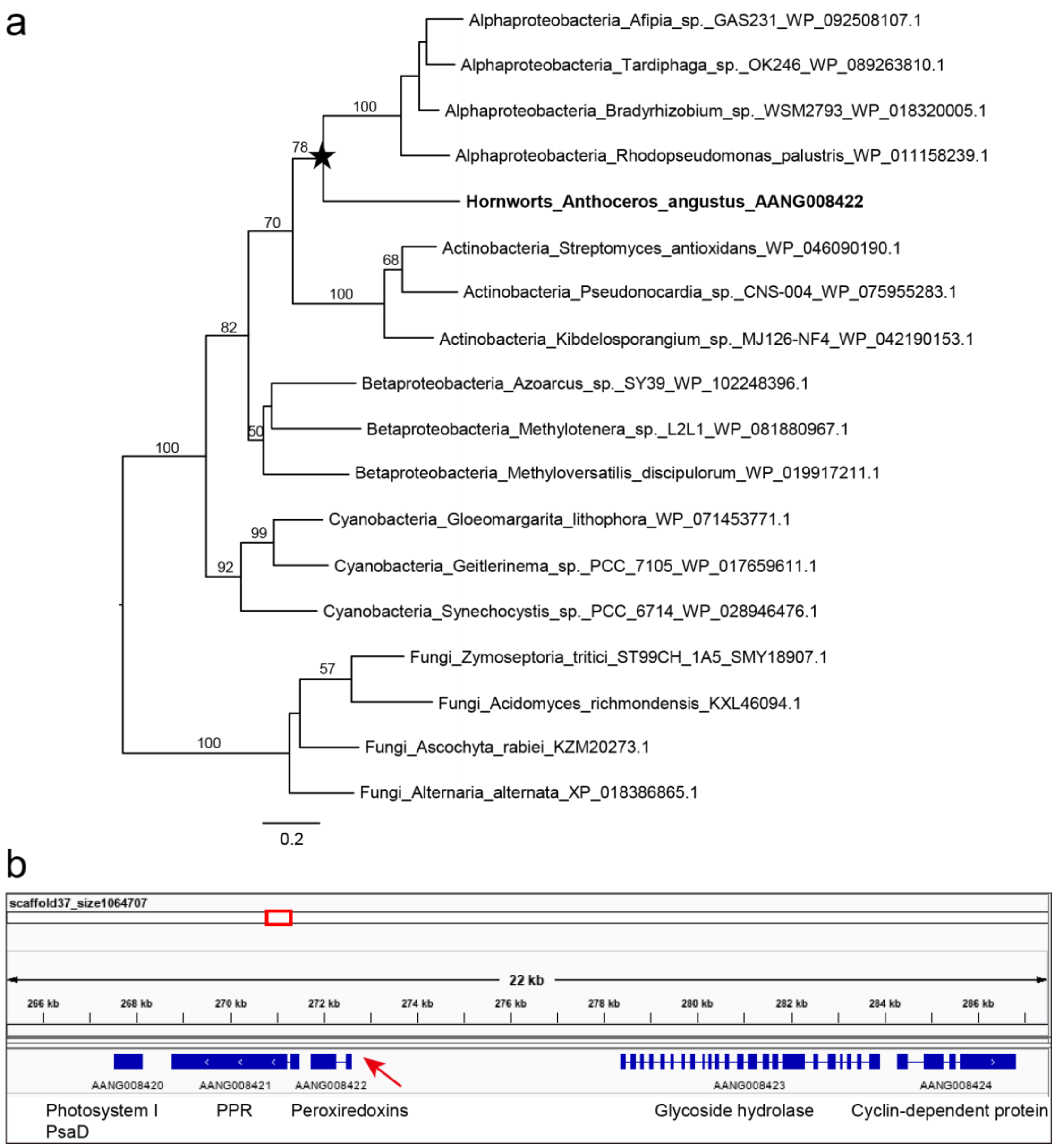

Supplementary Figure 74. Relationships of peroxiredoxins of *Anthoceros angustatus* and the location of their genes in the genome assembly. a, ML phylogenetic analysis of the *Anthoceros* HGT gene (AANG008422) and its homologs. Bootstrap support values ≥ 50% are shown above the branches. The fungal homologs were used as outgroup.The *Anthoceros* sequence (AANG008422) is sister to a highly supported clade of homologs of Alphaproteobacteria, suggesting an *Anthoceros*-specific HGT event from Alphaproteobacteria. All homologous genes of the family contain the redoxin domain (PFAM profile PF08534). **b**, Location of the *Anthoceros* HGT gene (AANG008422) in genome assembly. The AANG008422 has one intron and located in scaffold37, with up- and down-stream genes all being annotated as plant genes.

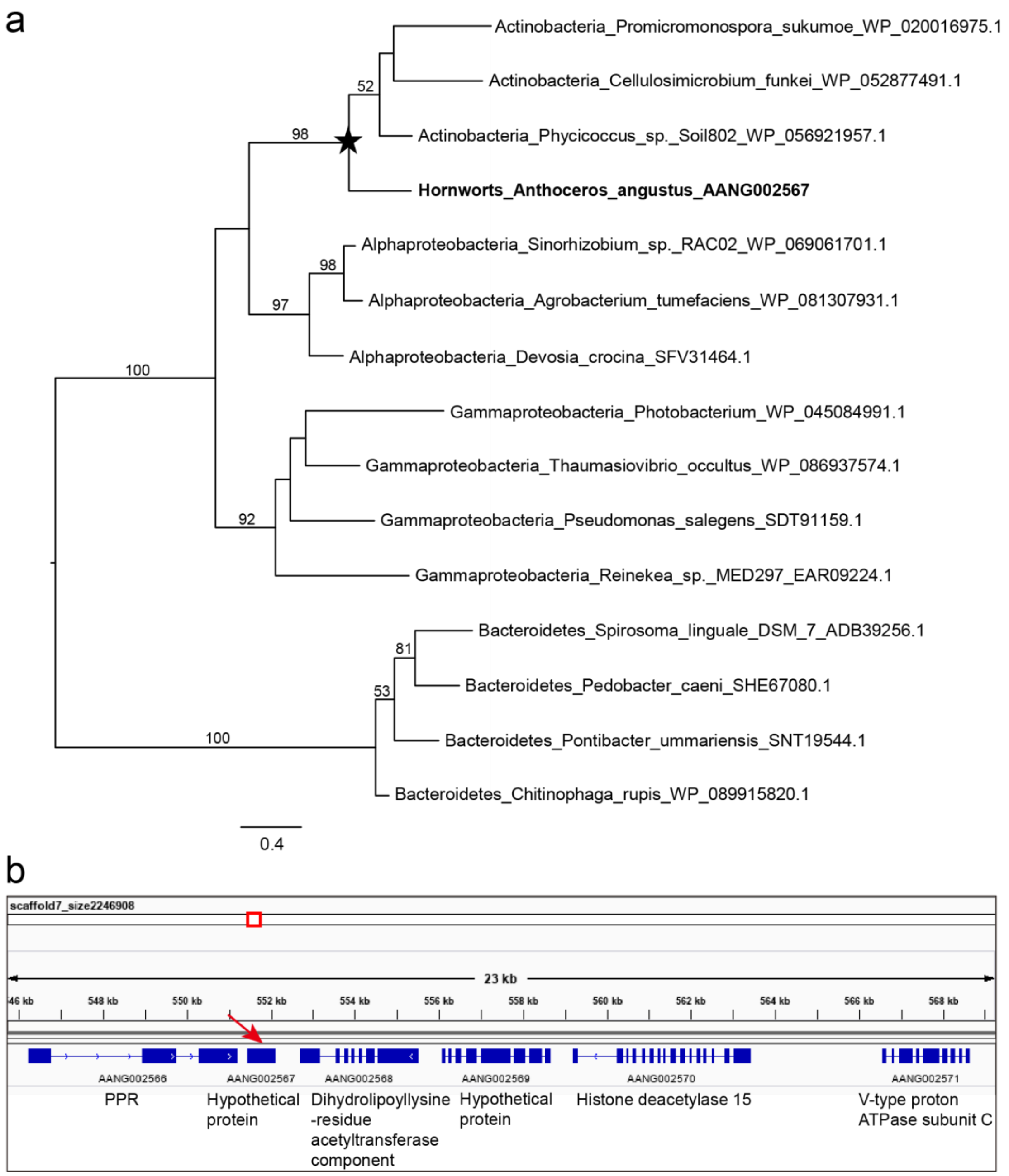

Supplementary Figure 75. Relationships of hypothetical proteins of *Anthoceros angustatus* and the location of their genes in the genome assembly. a, ML phylogenetic analysis of the *Anthoceros* HGT gene (AANG002567) and its homologs. No homologs were found in organisms other than bacteria and hornwort. Therefore, the homologous sequences from Bacteroidetes were used as outgroup. Bootstrap support values ≥ 50% are shown above the branches. The asterisk identifies the highly supported clade comprising the *Anthoceros* sequence (AANG002567) and homologs from Actinobacteria, suggesting an *Anthoceros*-specific HGT event from Actinobacteria. **b**, Location of the *Anthoceros* HGT gene (AANG002567) in genome assembly. AANG002567 is intronless and located in scaffold7, with up- and down-stream genes all being annotated as plant genes.

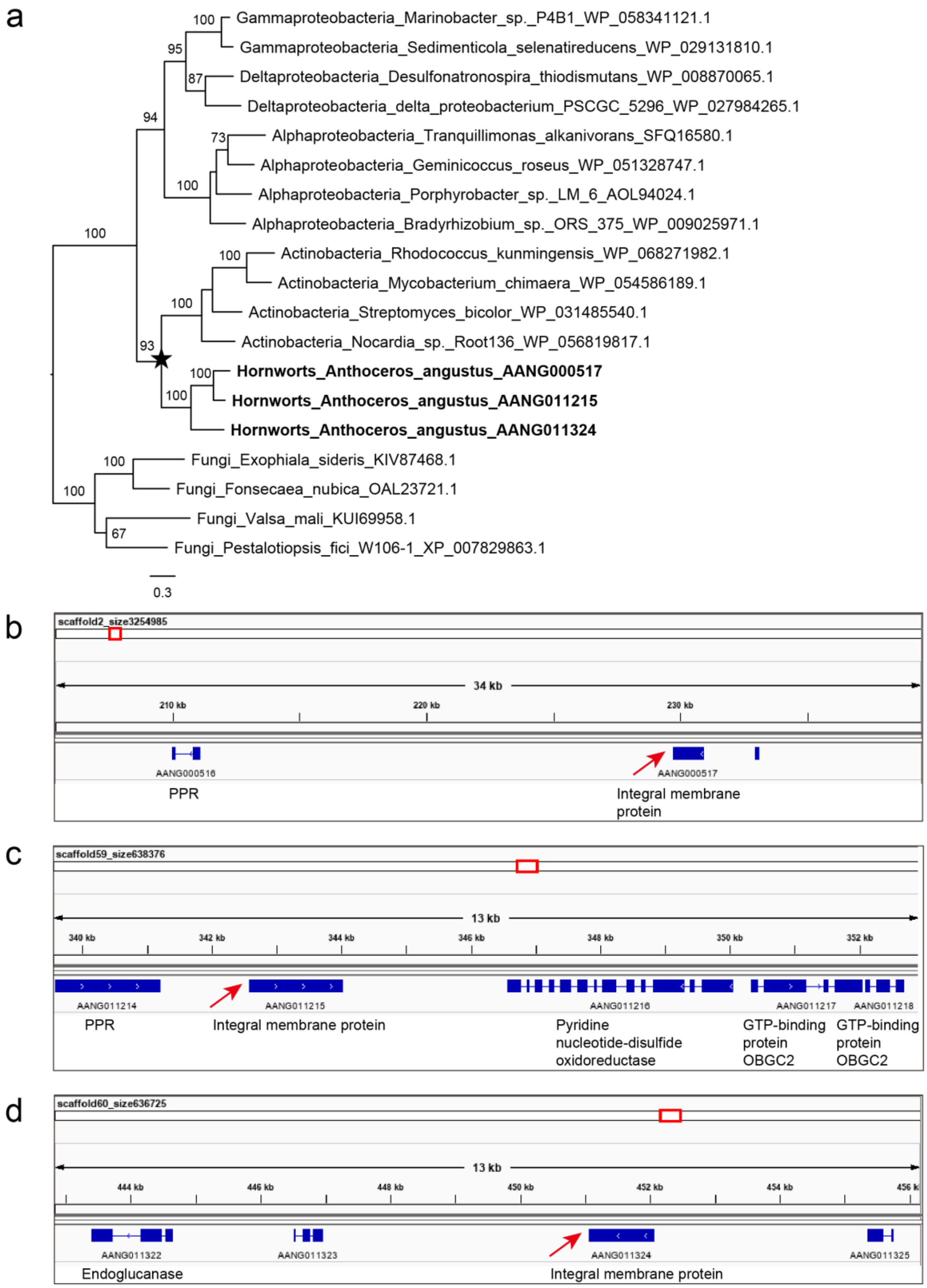

Supplementary Figure 76. Relationships of integral membrane proteins of *Anthoceros angustatus* and the location of their genes in the genome assembly. a, ML phylogenetic analysis of the *Anthoceros* HGT genes (AANG000517, AANG011215, and AANG011324) and their homologs. Bootstrap support values ≥ 50% are shown above the branches. The fungal homologs were used as outgroup. The asterisk identifies the highly supported clade comprising the three *Anthoceros* sequences and their homologs from Actinobacteria, suggesting an

Anthoceros-specific HGT event from Actinobacteria. **b-d**, Location of the *Anthoceros* HGT genes (AANG000517, AANG011215 and AANG011324) in genome assembly. AANG000517, AANG011215, and AANG011324 are all intronless, located in scaffold2 (**b**), scaffold59 (**c**), and scaffold60 (**d**), respectively, and most of their up- and down-stream genes are annotated as plant genes.

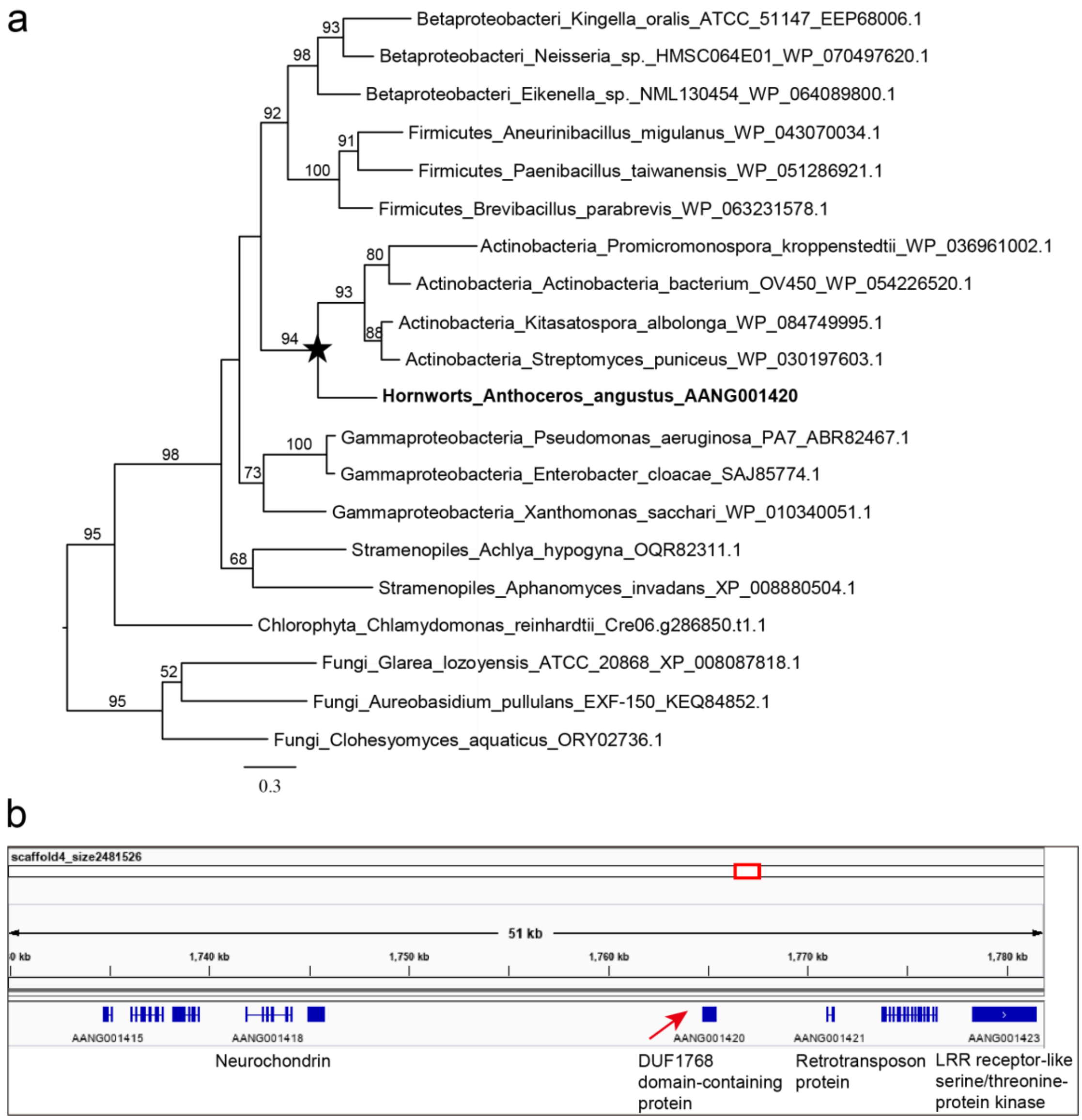

Supplementary Figure 77. Relationships of DUF1768 domain-containing proteins of *Anthoceros angustatus* and the location of their genes in the genome assembly. a, ML phylogenetic analysis of the *Anthoceros* HGT gene (AANG001420) and its homologs. Bootstrap support values ≥ 50% are shown above the branches. The fungal homologs were used as outgroup. The asterisk identifies the highly supported clade comprising the *Anthoceros* sequence (AANG001420) and the Actinobacteria homologs, suggesting an *Anthoceros*-specific HGT event from Actinobacteria. All homologous genes of the family in the phylogenetic tree contain the DUF1768 domain (PFAM profile PF08719). **b**, Location of the *Anthoceros* HGT gene (AANG001420) in the genome assembly. AANG001420 is intronless and located in scaffold4, with up- and down-stream genes all being annotated as plant genes.

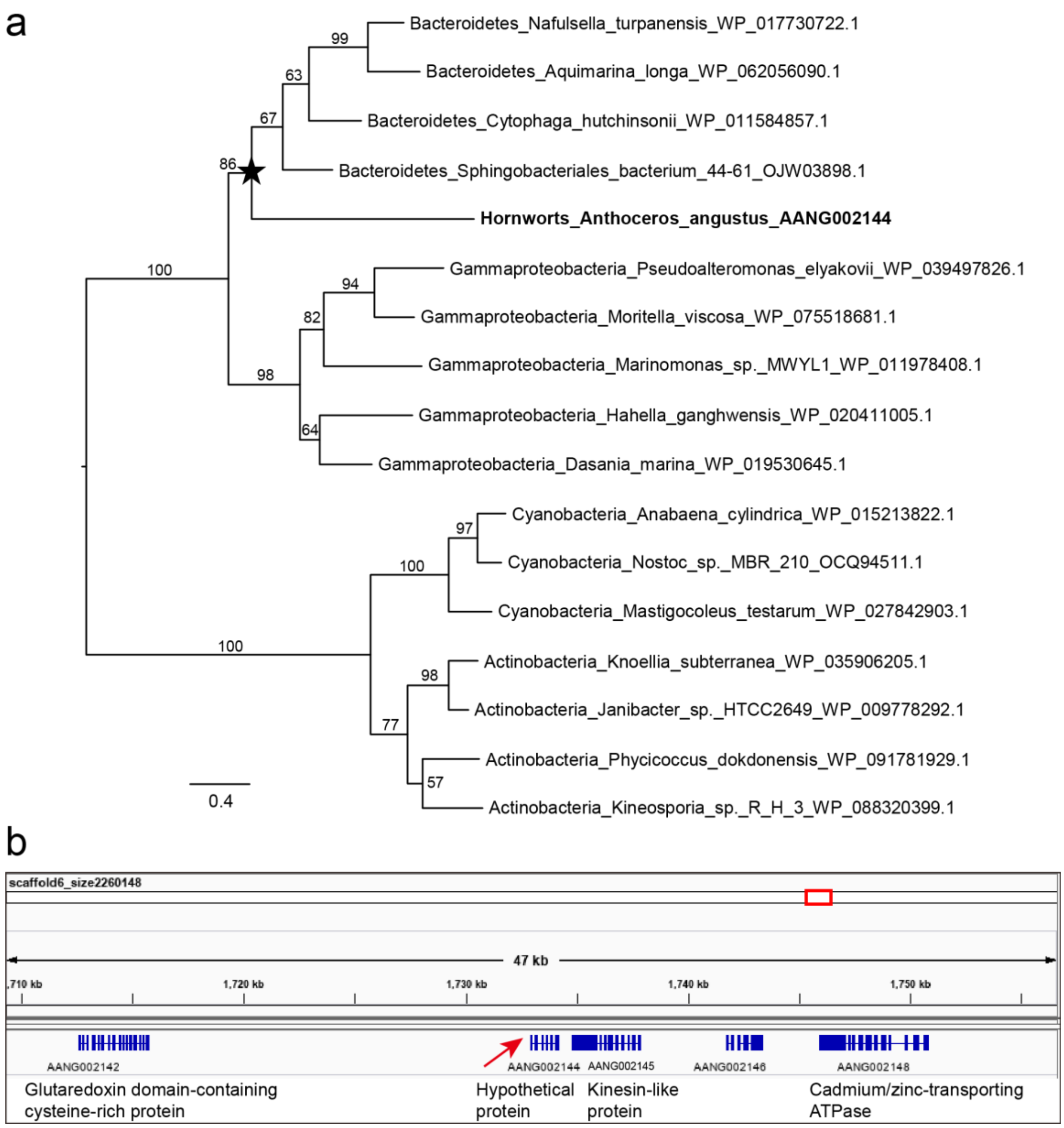

Supplementary Figure 78. Relationships of hypothetical proteins of *Anthoceros angustatus* and the location of their genes in the genome assembly. a, ML phylogenetic analysis of the *Anthoceros* HGT gene (AANG002144) and its homologs. Bootstrap support values ≥ 50% are shown above the branches. No homologs were found in organisms other than bacteria and hornwort. Therefore, the homologous sequences from Cyanobacteria and Actinobacteria were used as outgroup. The asterisk identifies the highly supported clade comprising the *Anthoceros* sequence (AANG002144) and the homologs from Bacteroidetes, suggesting an *Anthoceros*-specific HGT event from Bacteroidetes. **b**, Location of the *Anthoceros* HGT gene (AANG002144) in the genome assembly. AANG002144 has five introns and located in scaffold6, with up- and down-stream genes all being annotated as plant genes.

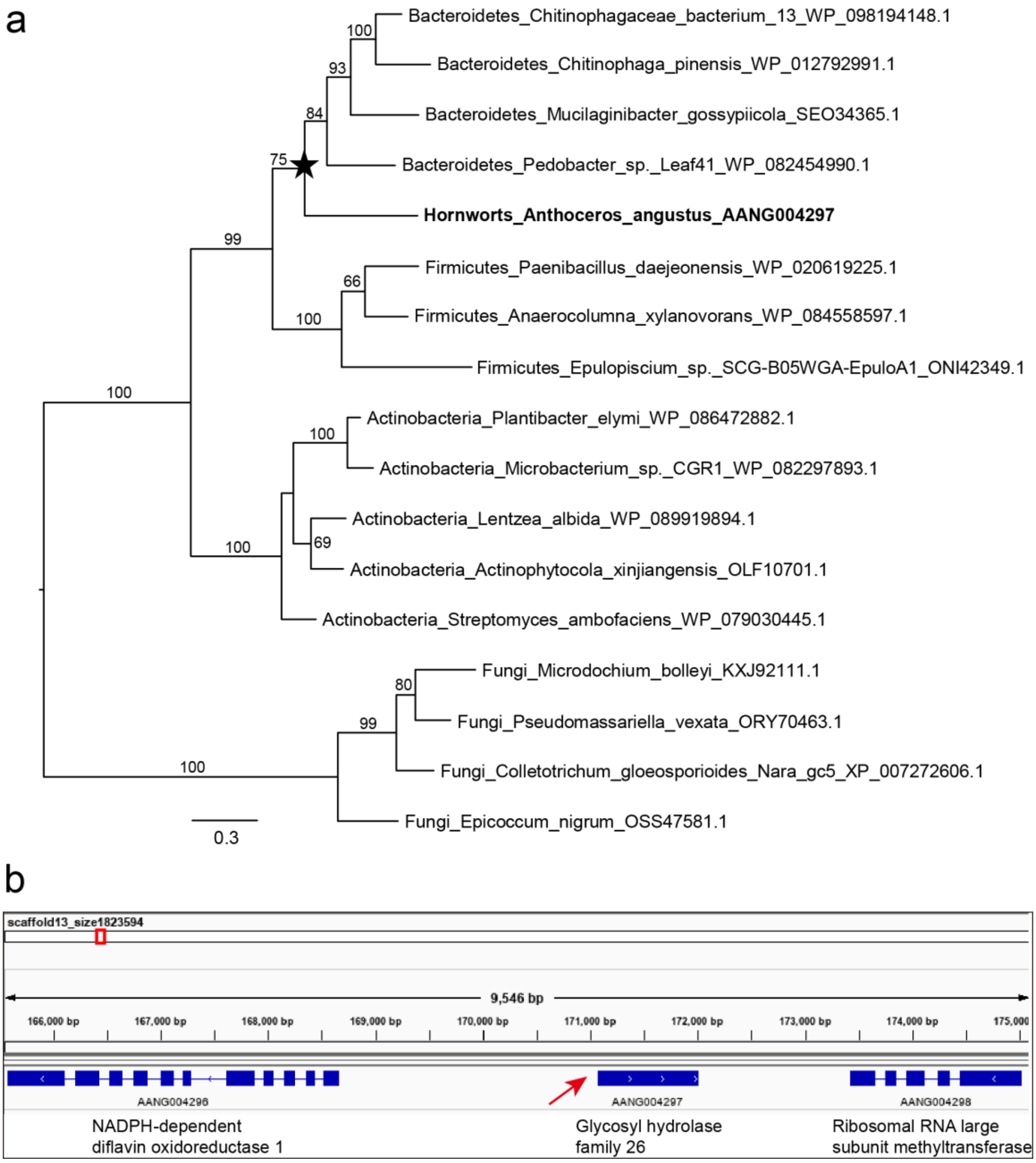

Supplementary Figure 79. Relationships of glycosyl hydrolase family 26 of *Anthoceros angustatus* and the location of their genes in the genome assembly. a, ML phylogenetic analysis of the *Anthoceros* HGT gene (AANG004297) and its homologs. Bootstrap support values ≥ 50% are shown above the branches. The fungal homologs were used as outgroup. The asterisk identifies the clade comprising the *Anthoceros* sequence (AANG004297) and the homologs from Bacteroidetes, suggesting an *Anthoceros*-specific HGT event from Bacteroidetes. All homologous genes of the family in the phylogenetic tree contain the glycosyl hydrolase family 26 domain (PFAM profile PF02156). **b**, Location of the *Anthoceros* HGT gene (AANG004297) in the genome assembly. AANG004297 is intronless and located in scaffold13, with up- and down-stream genes all being annotated as plant genes.

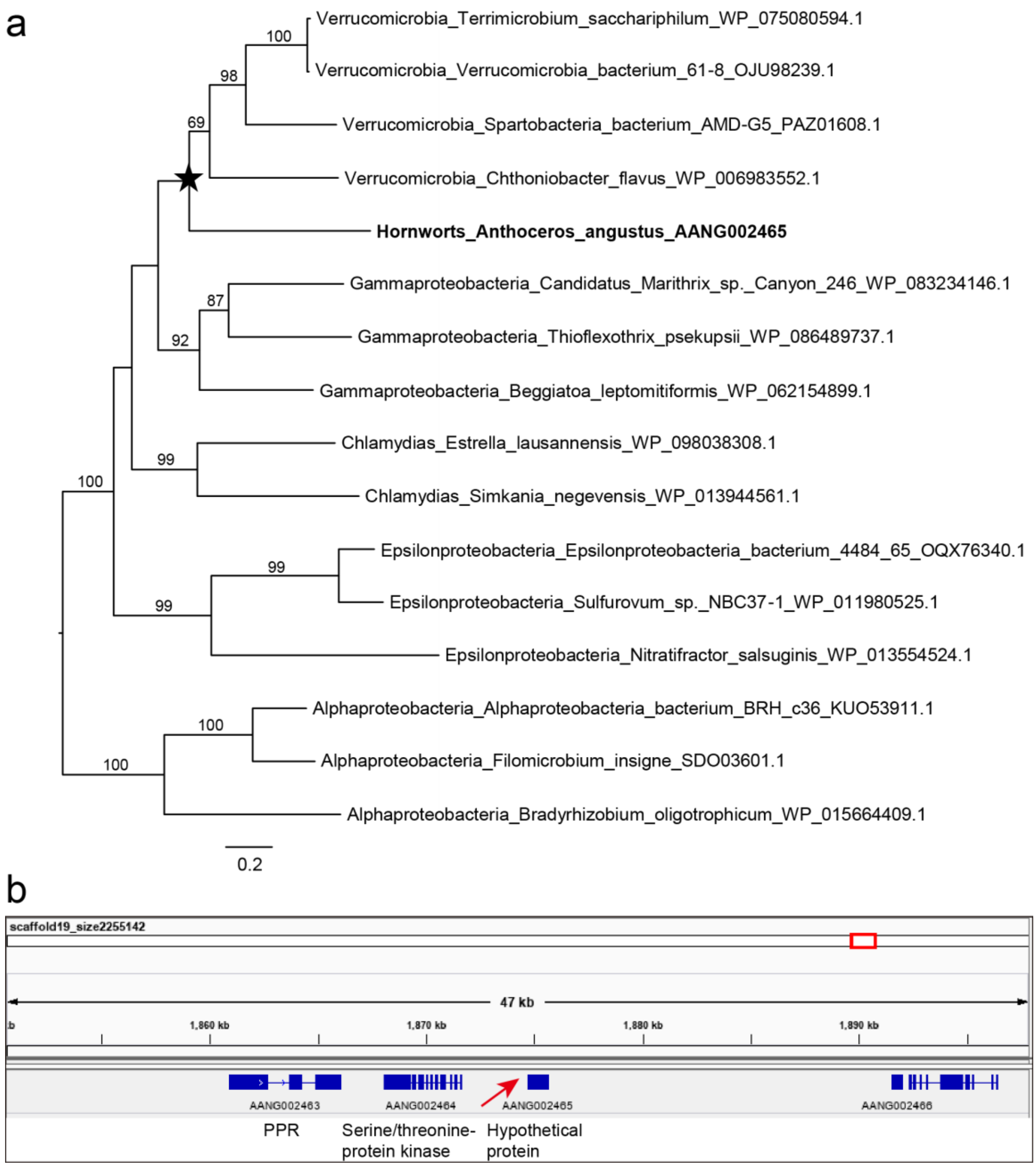

Supplementary Figure 80. Relationships of hypothetical proteins of *Anthoceros angustatus* and the location of their genes in the genome assembly. a, ML phylogenetic analysis of the *Anthoceros* HGT gene (AANG002465) and its homologs. Bootstrap support values ≥ 50% are shown above the branches. No homologs were found in organisms other than bacteria and hornwort. Therefore, the homologous sequences from Alphaproteobacteria were used as outgroup. The asterisk identifies the clade comprising the *Anthoceros* sequence (AANG002465) and its homologs in the Verrucomicrobia, suggesting an *Anthoceros*-specific HGT event from Verrucomicrobia. **b**, Location of the *Anthoceros* HGT gene (AANG002465) in the genome assembly. AANG002465 is intronless and located in scaffold19, with up- and down-stream genes all being annotated as plant genes.

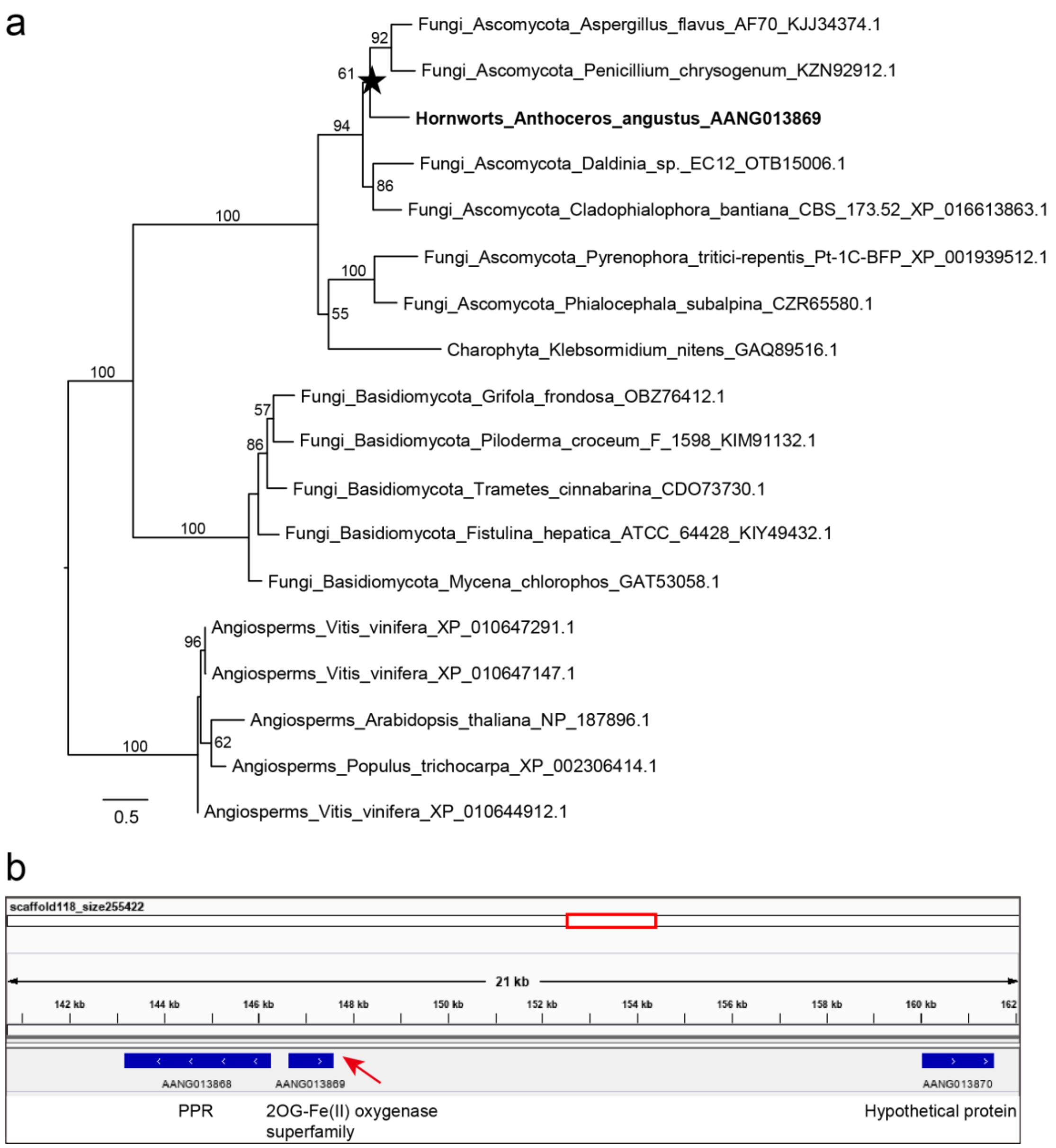

Supplementary Figure 81. Relationships of 2OG-Fe(II) oxygenase superfamily of *Anthoceros angustatus* and the location of their genes in the genome assembly. a, ML phylogenetic analysis of the *Anthoceros* HGT gene (AANG013869) and its homologs. Bootstrap support values ≥ 50% are shown above the branches. The plant homologs were used as outgroup. The asterisk identifies the clade comprising the *Anthoceros* sequence (AANG013869) and the homologs from Ascomycota, suggesting an *Anthoceros*-specific HGT event from Ascomycota. All homologous genes of the family in the phylogenetic tree contain the 2OG-Fe(II) oxygenase superfamily domain (PFAM profile PF03171). **b**, Location of the *Anthoceros* HGT gene (AANG013869) in the genome assembly. AANG013869 is intronless and located in scaffold118, with up- and down-stream genes all being annotated as plant genes.

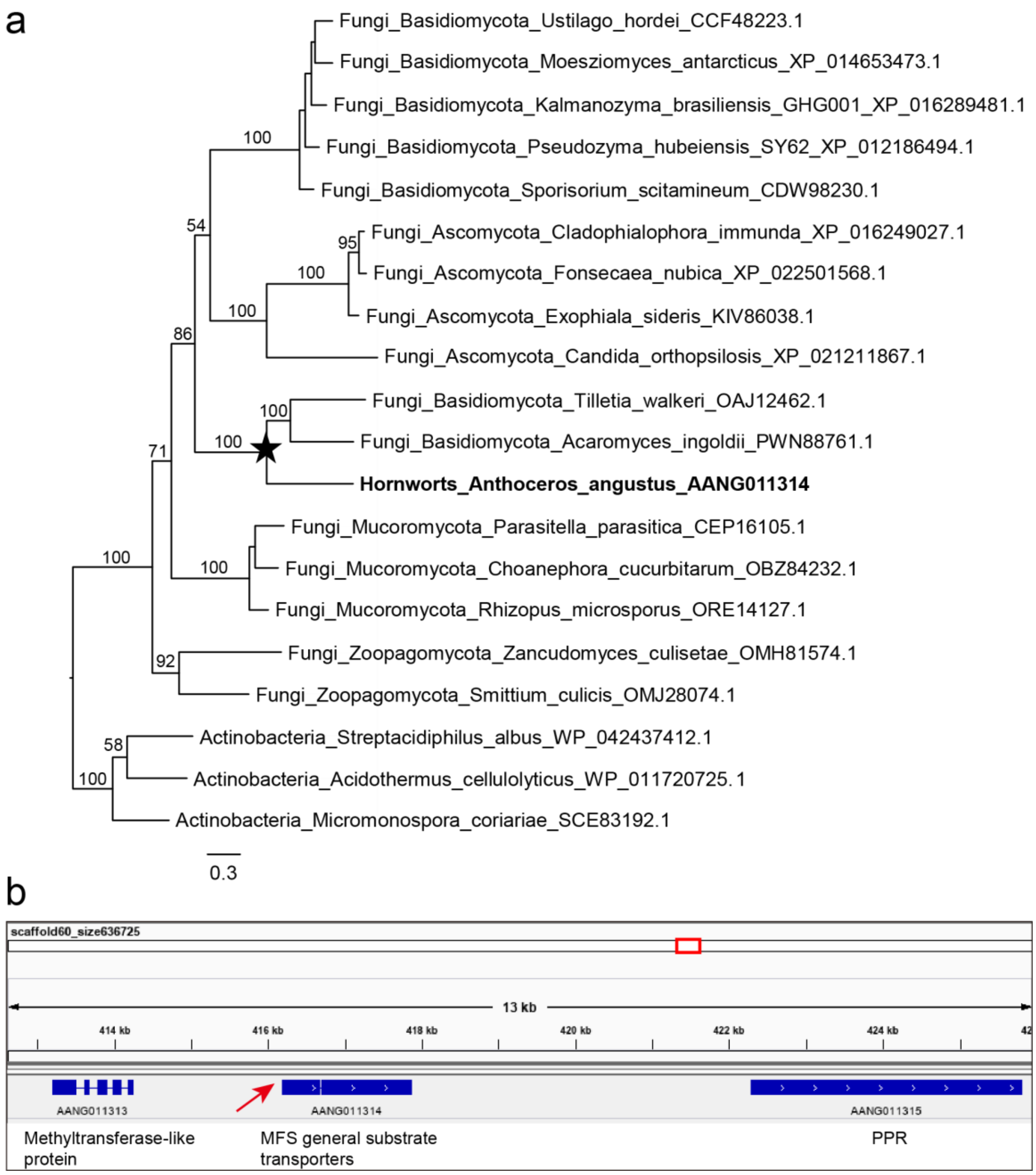

Supplementary Figure 82. Relationships of MFS general substrate transporters of *Anthoceros angustatus* and the location of their genes in the genome assembly. a, ML phylogenetic analysis of the *Anthoceros* HGT gene (AANG011314) and its homologs. Bootstrap support values ≥ 50% are shown above the branches. The bacterial homologs were used as outgroup. The asterisk identifies the highly supported clade comprising the *Anthoceros* sequence (AANG011314) and the homologs from Basidiomycota, suggesting an *Anthoceros*-specific HGT event from Basidiomycota. All homologous genes of the family in the phylogenetic tree contain the major facilitator superfamily domain (PFAM profile PF07690). **b**, Location of the *Anthoceros* HGT gene (AANG011314) in the genome assembly. AANG011314 has one intron and located in scaffold60, with up- and down-stream genes all being annotated as plant genes.

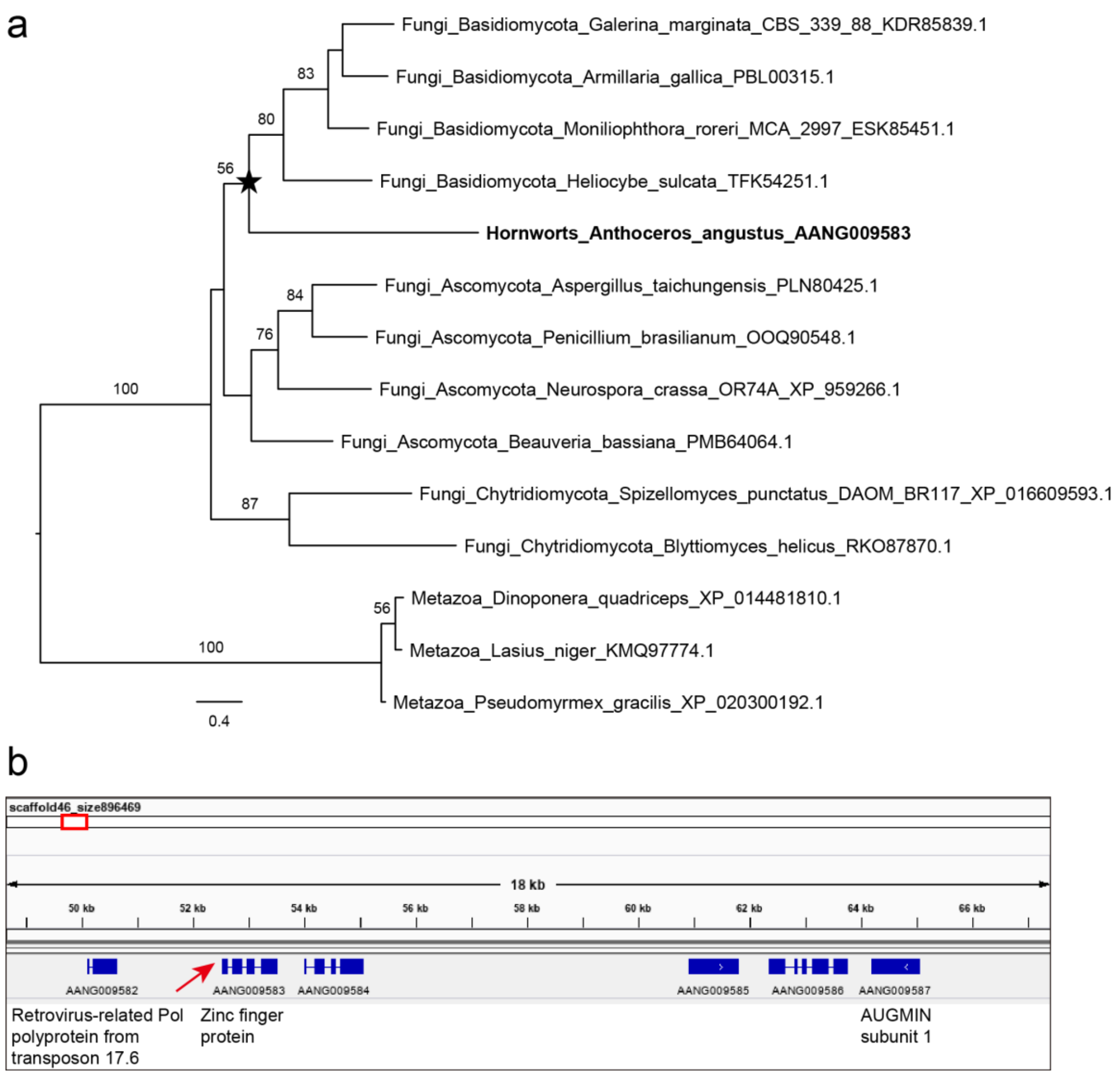

Supplementary Figure 83. Relationships of Zinc finger proteins of *Anthoceros angustatus* and the location of their genes in the genome assembly. a, ML phylogenetic analysis of the *Anthoceros* HGT gene (AANG009583) and its homologs. Bootstrap support values ≥ 50% are shown above the branches. The metazoa homologs were used as outgroup. The asterisk indicates that the *Anthoceros* sequence (AANG009583) forms a clade with the homologs from Basidiomycota, suggesting an *Anthoceros*-specific HGT event from Basidiomycota. All homologous genes of the family in the phylogenetic tree contain the Zinc finger domain (PFAM profile PF07690). **b**, Location of the *Anthoceros* HGT gene (AANG009583) in the genome assembly. AANG009583 has three introns and located in scaffold46, with up- and down-stream genes all being annotated as plant genes.

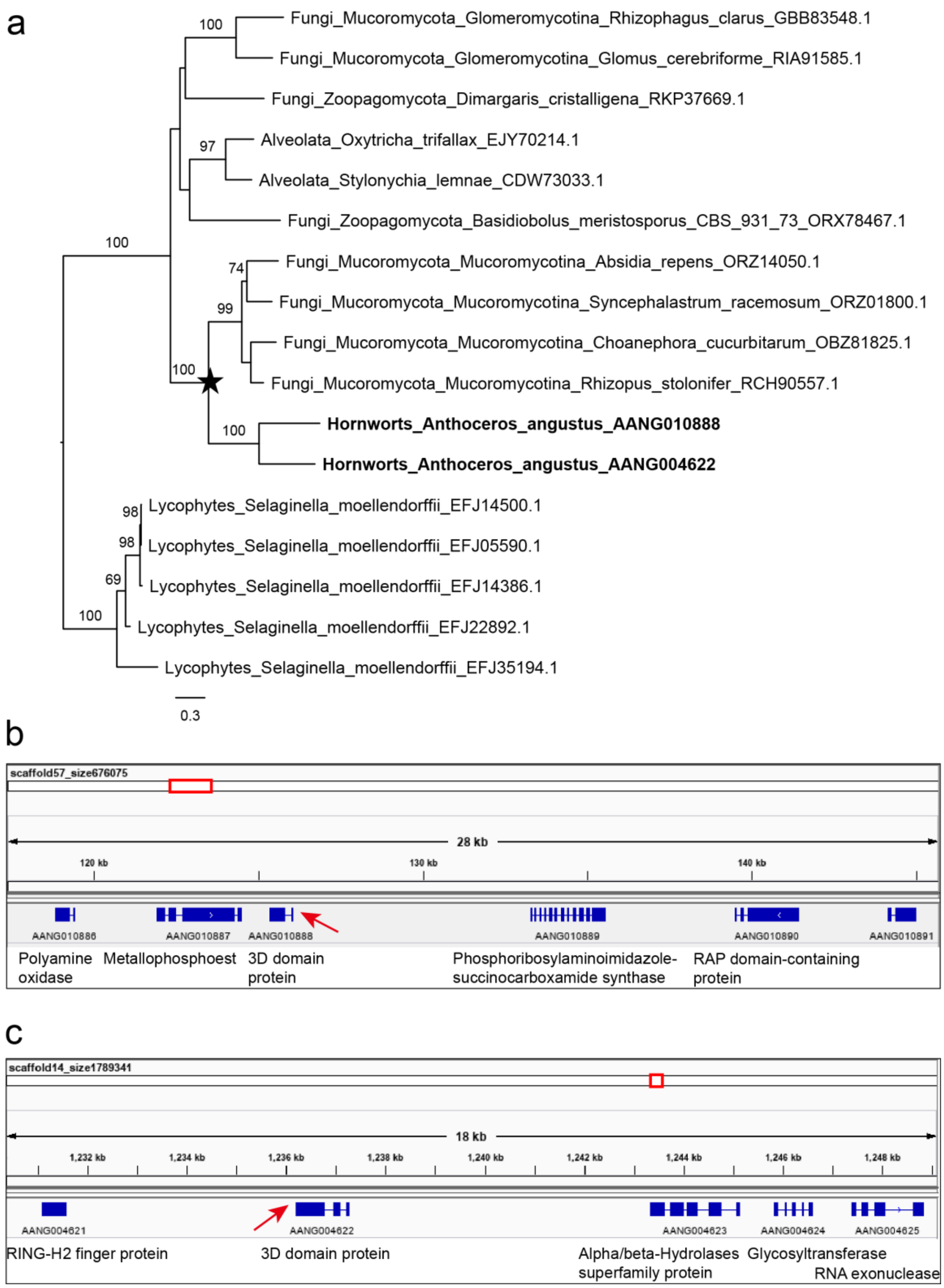

Supplementary Figure 84. Relationships of 3D domain proteins of *Anthoceros angustatus* and the location of their genes in the genome assembly. a, ML phylogenetic analysis of the *Anthoceros* HGT genes (AANG010888 and AANG004622) and their homologs. Bootstrap support values ≥ 50% are shown above the branches. The plant homologs were used as outgroup. The asterisk identifies the highly supported clade comprising the *Anthoceros* sequences (AANG010888 and AANG004622) and the homologs from Mucoromycota, suggesting an *Anthoceros*-specific HGT event from Mucoromycota. **b-c**, Location of the *Anthoceros* HGT genes

(AANG010888 and AANG004622) in the genome assembly. AANG010888 and AANG004622 have introns and located in scaffold57 (**b**) and scaffold14 (**c**), respectively, with up- and down-stream genes all being annotated as plant genes.

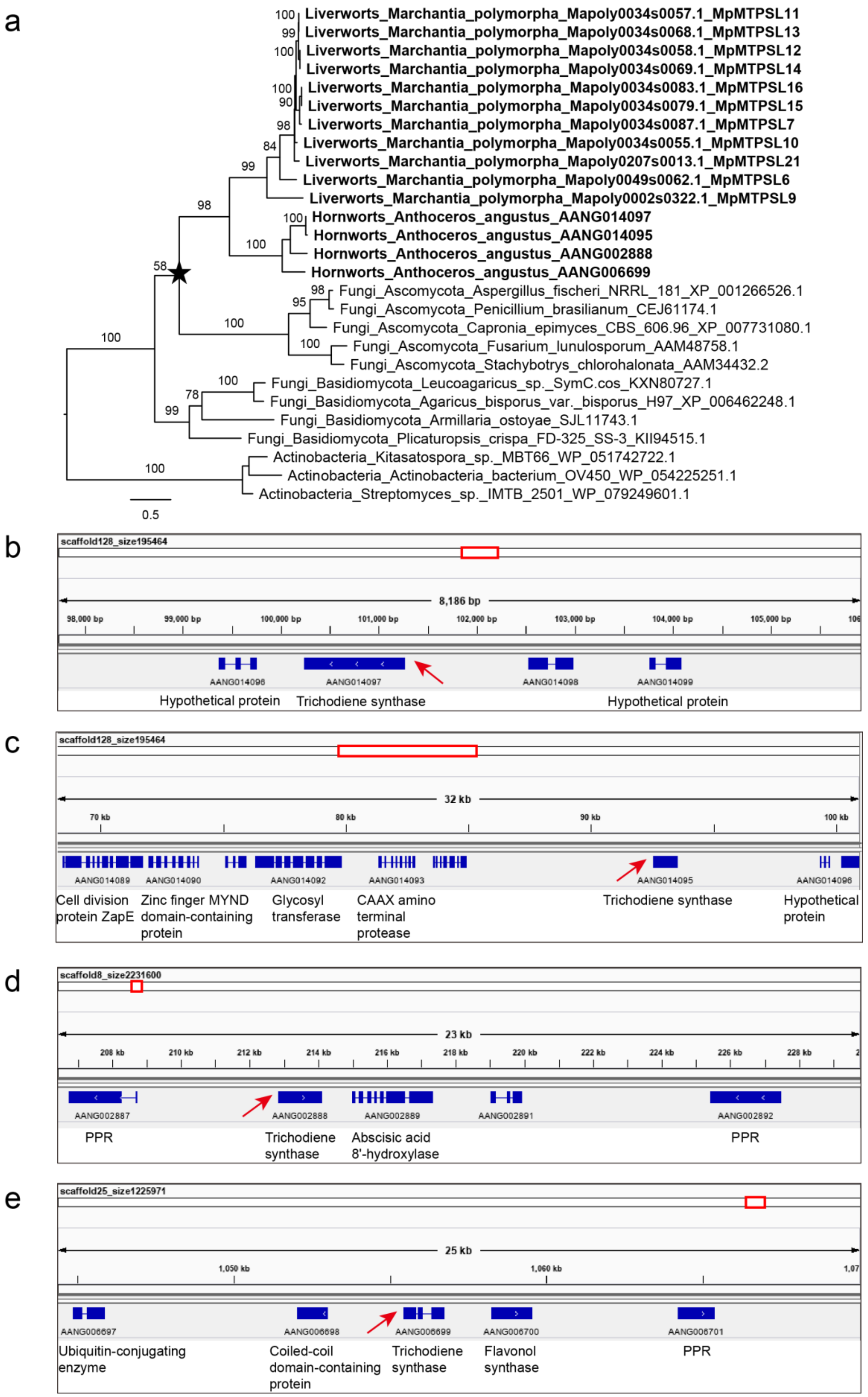
a
Liverworts_Marchantia_polymorpha_Mapoly0034s0057.1_MpMTPSL11
Liverworts_Marchantia_polymorpha_Mapoly0034s0068.1_MpMTPSL13
Liverworts_Marchantia_polymorpha_Mapoly0034s0058.1_MpMTPSL12
Liverworts_Marchantia_polymorpha_Mapoly0034s0069.1_MpMTPSL14
Liverworts_Marchantia_polymorpha_Mapoly0034s0083.1_MpMTPSL16
Liverworts_Marchantia_polymorpha_Mapoly0034s0079.1_MpMTPSL15
Liverworts_Marchantia_polymorpha_Mapoly0034s0087.1_MpMTPSL7
Liverworts_Marchantia_polymorpha_Mapoly0034s0055.1_MpMTPSL10
Liverworts_Marchantia_polymorpha_Mapoly0207s0013.1_MpMTPSL21
Liverworts_Marchantia_polymorpha_Mapoly0049s0062.1_MpMTPSL6
Liverworts_Marchantia_polymorpha_Mapoly0002s0322.1_MpMTPSL9
Hornworts_Anthoceros_angustus_AANG014097
Hornworts_Anthoceros_angustus_AANG014095
Hornworts_Anthoceros_angustus_AANG002888
Hornworts_Anthoceros_angustus_AANG006699
Fungi_Ascomycota_Aspergillus_fischeri_NRRL_181_XP_001266526.1
Fungi_Ascomycota_Penicillium_brasilianum_CEJ61174.1
Fungi_Ascomycota_Capronia_epimyces_CBS_606.96_XP_007731080.1
Fungi_Ascomycota_Fusarium_lunulosporum_AAM48758.1
Fungi_Ascomycota_Stachybotrys_chlorohalonata_AAM34432.2
Fungi_Basidiomycota_Leucoagaricus_sp._SymC.cos_KXN80727.1
Fungi_Basidiomycota_Agaricus_bisporus_var._bisporus_H97_XP_006462248.1
Fungi_Basidiomycota_Armillaria_ostoyae_SJL11743.1
Fungi_Basidiomycota_Plicaturopsis_crispa_FD-325_SS-3_KII94515.1
Actinobacteria_Kitasatospora_sp._MBT66_WP_051742722.1
Actinobacteria_Actinobacteria_bacterium_OV450_WP_054225251.1
Actinobacteria_Streptomyces_sp._IMTB_2501_WP_079249601.1
0.5
b
scaffold128_size195464
8,186 bp
AANG014096
AANG014097
AANG014098
AANG014099
Hypothetical protein
Trichodiene synthase
Hypothetical protein
c
scaffold128_size195464
32 kb
70 kb
80 kb
90 kb
100 kb
AANG014089
AANG014090
AANG014092
AANG014093
AANG014095
AANG014096
Cell division protein ZapE
Zinc finger MYND domain-containing protein
Glycosyl transferase
CAAX amino terminal protease
Trichodiene synthase
Hypothetical protein
d
scaffold8_size2231600
23 kb
208 kb
210 kb
212 kb
214 kb
216 kb
218 kb
220 kb
222 kb
224 kb
226 kb
228 kb
AANG002887
AANG002888
AANG002889
AANG002891
AANG002892
PPR
Trichodiene synthase
Abscisic acid 8'-hydroxylase
PPR
e
scaffold25_size1225971
25 kb
1,050 kb
1,060 kb
AANG006697
AANG006698
AANG006699
AANG006700
AANG006701
Ubiquitin-conjugating enzyme
Coiled-coil domain-containing protein
Trichodiene synthase
Flavonol synthase
PPR

Supplementary Figure 85. Relationships of trichodiene synthases (terpene synthase-like, MTPSL) of *Anthoceros angustatus* and the location of their genes in the genome assembly. a, ML phylogenetic analysis of the *Anthoceros* HGT genes (AANG014097, AANG014095, AANG002888, and AANG006699) and their homologs. Bootstrap support values ≥ 50% are shown above the branches. The bacterial homologs were used as outgroup. The asterisk identifies thehighly supported clade comprising the four *Anthoceros* sequences and their homologs in liverworts and ascomycetes, congruent with a bryophytes-specific HGT event from fungi. The HGT in *Marchantia* has been confirmed by previous studies (Kumar et al., 2016; Jia et al., 2016; Jia et al., 2017; Bowman et al., 2017). All homologous genes of the family in the phylogenetic tree contain the TRI5 domain (PFAM profile PF06330). **b-e**, Location of the *Anthoceros* HGT genes (AANG014097, AANG014095, AANG002888, and AANG006699) in the genome assembly. Of these four HGT genes, only AANG006699 has introns, and AANG014097, AANG014095, AANG002888, and AANG006699 located in scaffold128 (**b, c**), scaffold8 (**d**), and scaffold25 (**e**), respectively, with up- and down-stream genes all being annotated as plant genes.

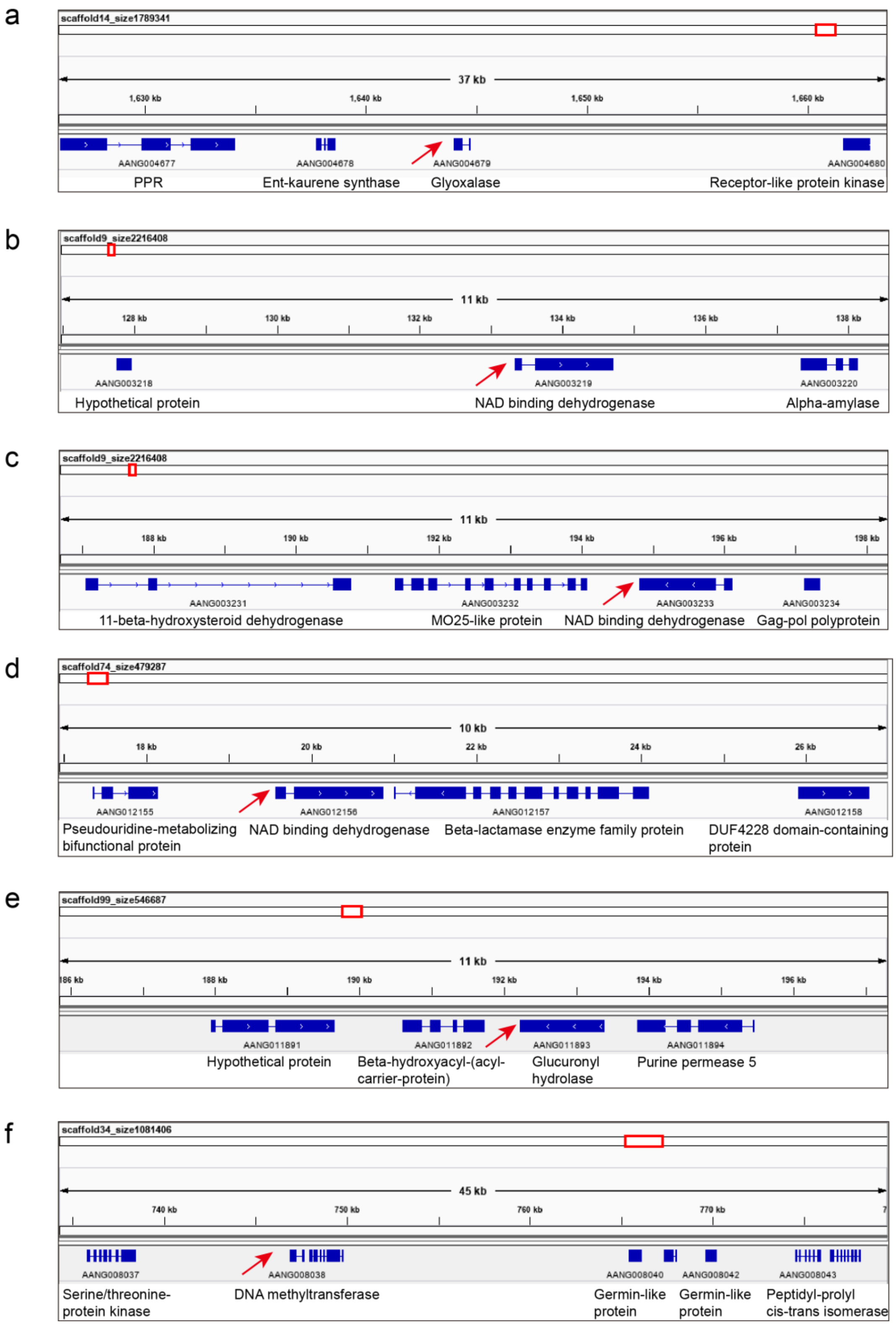

Supplementary Figure 86. Location of the *Anthoceros* HGT genes AANG004679 (a), AANG003219 (b), AANG003233 (c), AANG012156 (d), AANG011893 (e), and AANG08038 (f) in the genome assembly. Of these genes, except for AANG011893, all the other genes have introns. AANG004679, AANG003219, AANG003233, AANG012156, AANG011893 are located in scaffold14 (**a**), scaffold9 (**b, c**), scaffold74 (**d**), scaffold99 (**e**), and scaffold34 (**f**), respectively, with up- and down-stream genes all being annotated as plant genes.

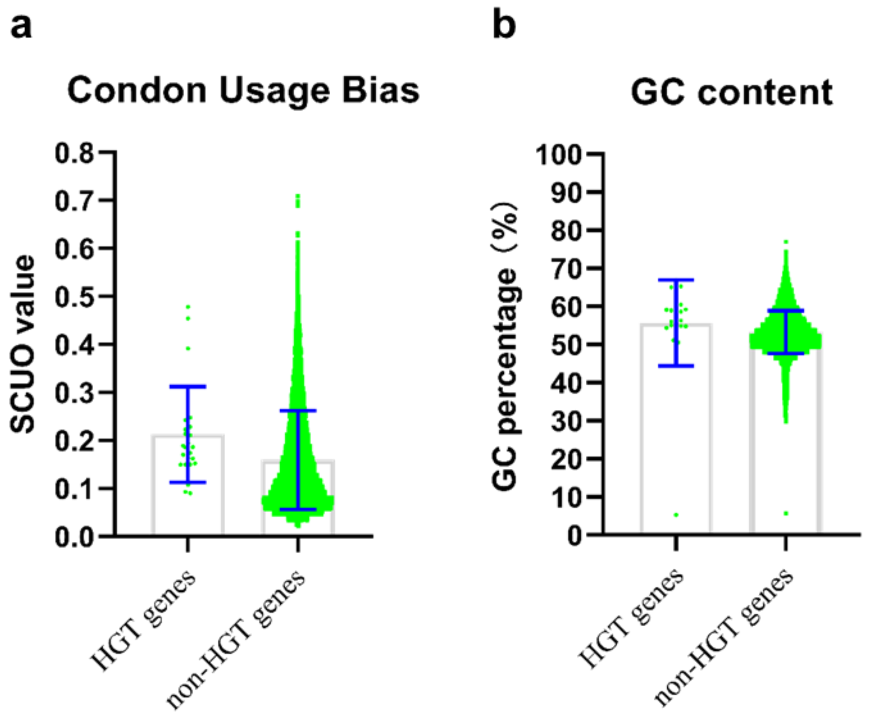

Supplementary Figure 87. The comparisons of codon usage bias and GC content of HGT genes (n=25) and non-HGT genes (n=14604). The measure of center and the error bar indicates mean and standard deviation, respectively. Significance was examined by T-test and the test was two-sided. **a**, Dot plots of codon usage bias (SCUO value) of HGT and non-HGT genes. HGT genes (0.2127) show a significantly more biased codon-usage pattern than non-HGT genes (0.1595) with P-value < 0.0100. **b**, Dot plots of GC percentages of HGT and non-HGT genes. The mean GC content of HGT genes (57.58%) is significantly higher than that of non-HGT genes (53.26%) with P-value < 0.0001.

Supplementary Tables

Supplementary Table 1. Summary of Illumina library construction information before decontamination for the *Anthoceros angustus* genome.

Pair-end libraries	Insert size	Average read length (bp)	Total data (bp)
Trimmed reads	170 bp	100	15,819,124,000
	250 bp	145/150	40,090,515,020
	350 bp	80	3,628,123,520
	500 bp	60/80/90/95/100	32,381,504,020
	800 bp	80	3,357,972,160
	2 kb	49	9,209,118,000
	5 kb	40	2,017,615,002
	10 kb	40/49	12,055,887,666
	20 kb	39/40/49	6,741,324,896
	40 kb	49	1,231,197,128
Total			126,532,381,412

Supplementary Table 2. Summary of Illumina library construction information after decontamination for the *Anthoceros angustus* genome.

Pair-end libraries	Insert size	Average read length (bp)	Total data (bp)	Sequence depth (X)*	Physical depth (X)*
Filtered reads	170bp	100	2,758,917,800	25.78	21.92
	250bp	145/150	9,351,365,110	87.40	72.97
	350bp	80	156,867,520	1.47	3.21
	500bp	60/80/90/95/100	3,383,844,710	31.62	88.04
	800bp	80	243,763,680	2.28	11.39
	2Kb	49	654,340,218	6.12	124.80
	5Kb	40	74,537,920	0.70	43.54
	10Kb	40/49	319,330,858	2.98	323.58
	20Kb	39/40/49	134,473,398	1.26	273.05
	40Kb	49	21,586,362	0.20	82.34
Total			17,099,027,576	159.8	1,044.84

*The genome size was estimated to be 107 Mb by the *k*-mer analysis.

Supplementary Table 3. Summary of Oxford Nanopore sequencing for the *Anthoceros angustus* genome.

	Raw reads (bp)	Filtered reads (bp)	Corrected reads (bp)	Decontaminated reads (bp)
Total length	63,614,292,295	36,070,452,175	9,247,957,448	3,783,984,766
Average length	2,703.88	3,247.34	40,294.36	39,476.96
N50 length	7,103	8,162	41,202	40,136

Supplementary Table 4. Summary of the final *Anthoceros angustus* genome assembly.

	Contig		Scaffold	
	**Size (bp)**	**Number**	**Size (bp)**	**Number**
**N50**	796,636	46	1,092,075	33
**N60**	546,971	64	948,865	45
**N70**	409,639	89	654,174	60
**N80**	300,535	123	458,029	82
**N90**	146,516	180	263,774	116
**Longest (bp)**	3,254,985	1	3,809,330	1
**Total size (bp)**	119,122,644	444	119,333,152	289
**Total number (>1 kp)**	119,122,644	444	119,333,152	289
**Total number (>2 kb)**	119,122,644	444	119,333,152	289
**Total number (>5 kb)**	119,122,644	444	119,333,152	289
**GC ratio (%)**	49.60		49.60	

Supplementary Table 5. Mapping summary of the transcripts on the assembled *Anthoceros angustus* genome.

Dataset	Number	Total length	Covered by assembly	with >90% sequences in one scaffold		with >50% sequences in one scaffold	
				Number	Percent	Number	Percent
>200 bp	26,805	28,098,420	97.66%	25,290	94.35%	26,118	97.44%
>500 bp	18,643	24,982,830	98.13%	17,923	96.14%	18,354	98.45%
>800 bp	12,942	21,356,694	98.39%	12,550	96.97%	12,794	98.86%

Supplementary Table 6. Prediction of the repeat sequences in the *Anthoceros angustus* genome.

Type	Repeat size (bp)	% of genome
**TRF**	8,331,234	6.98
**RepeatMasker**	4,687,639	3.93
**RepeatProteinMask**	10,020,398	8.40
De novo	63,628,660	53.32
**Total***	76,625,567	64.21

Total repeat regions were identified combining all the repeat identified. As there are some overlaps between different methods, the total region is shorter than the sum of repeats identified by all the methods.

Supplementary Table 7. Categories of transposable elements (TEs) predicted in the *Anthoceros angustus* genome.

Type	Repbase TEs		TE proteins		*De novo*		Combined TEs	
	Length (bp)	% in genome	Length (bp)	% in genome	Length (bp)	% in genome	Length (bp)	% in genome
DNA	254,445	0.21	633,086	0.53	8,964,400	7.51	9,752,109	8.17
LINE	137,194	0.11	320,550	0.27	3,401,521	2.85	3,832,690	3.21
SINE	2,083	0.00	0	0.00	641,828	0.54	643,147	0.54
LTR	4,163,476	3.49	9,079,956	7.61	44,580,906	37.36	53,812,343	45.09
Other	465	0.00	0	0.00	0	0.00	465	0.00
Unknown	4,149	0.00	0	0.00	8,683,889	7.28	8,687,957	7.28
Total	4,687,639	3.93	10,020,398	8.40	61,825,815	51.81	72,224,921	60.52

Note: Repbase TEs: the result of RepeatMasker program based on Repbase database; TE proteins: the result of RepeatProteinMask program based on known TE-related protein of Repbase; *De novo*: the result of RepeatMasker by using library predicted by *De novo* methods; Combined TEs: combine the results of Repbase TEs, TE proteins and *De novo*.

Supplementary Table 8. Gene annotation summary for the *Anthoceros angustus* genome.

Gene set		Number	Average gene length (bp)	Average CDS length (bp)	Average exon per gene	Average exon length (bp)	Average intron per gene	Average intron length (bp)
De novo	AUGUSTUS	15,662	1,855.15	1,334.19	5.02	265.62	4.03	129.50
	GlimmerHMM	23,451	1,478.75	1,004.84	2.78	361.08	1.79	265.81
Homolog	*Arabidopsis thaliana*	27,394	2,445.07	979.14	2.86	342.87	1.85	789.96
	Selaginella moellendorffii	15,607	2,338.08	815.00	3.20	254.47	2.20	691.45
	Physcomitrella patens	14,290	2,045.87	744.99	3.29	226.22	2.29	567.27
	Marchantia polymorpha	17,062	2,009.49	825.20	3.63	227.03	2.64	449.50
	Klebsormidium nitens	16,944	1,828.14	799.68	3.43	232.88	2.44	422.56
RNA-Seq		9,782	4,376.01	1,449.63	4.67	310.66	3.67	160.34
MAKER		13,134	3,071.39	1,356.08	5.75	391.83	4.76	171.80
Final set		14,629	1972.11	1313.24	4.81	272.63	3.81	172.61

Supplementary Table 9. Number of predicted protein-coding genes with homology or functional classification by each method for the *Anthoceros angustus* genome.

	Database	Number	Percent (%)
	Swissprot	5,533	37.82
	TrEMBL	6,422	43.90
**Annotated**	Pfam	10,002	68.37
	GO	6,196	42.35
	KEGG	7,850	53.66
	Total	11,468	78.39
**Unannotated**		3,161	21.61
**Total**		14,629	100.00

Supplementary Table 10. Comparison of BUSCO assessment of genome annotation among three bryophytes.

	A. angustus		*M. polymorpha*		*P. patens*	
	**Proteins**	**Percent (%)**	**Proteins**	**Percent (%)**	**Proteins**	**Percent (%)**
Complete BUSCOs	813	85.04	859	89.85	841	87.97
Complete Single-Copy BUSCOs	529	55.33	555	58.05	462	48.33
Complete Duplicated BUSCOs	284	29.71	304	31.80	379	39.64
Fragmented BUSCOs	44	4.60	22	2.30	53	5.54
Missing BUSCOs	99	10.36	75	7.85	62	6.49
Total BUSCO groups searched	956	100.00	956	100.00	956	100.00

Supplementary Table 11. Identification of non-coding RNA genes in the *Anthoceros angustus* genome.

		Copy	Average length (bp)	Total length (bp)	% of genome
miRNA	known mature	30	20.8	624	0.0005
	known precursor	56	146.46	8,202	0.0069
	novel mature	180	21.69	3,904	0.0033
	novel precursor	182	70	12,740	0.0107
tRNA		347	75.40	26,164	0.0219
rRNA	18S	25	927.92	23,198	0.0194
	28S	8	185.50	1,484	0.0012
	5.8S	18	144.94	2,609	0.0037
	5S	43	103.14	4,435	0.0018
	Total rRNA	94	337.51	31,726	0.0266
snRNA	CD-box	18	126.06	2,269	0.0019
	HACA-box	0	0	0	0
	splicing	65	152.29	9,899	0.0083
	scaRNA	0	0	0	0
	Total snRNA	83	146.60	12,168	0.0102

Supplementary Table 12. Conserved land plant miRNAs present in different land plant species.

miRNA	Hornwort	Liverworts	Moss	Lycophyte	Fern	Gymnosperm	Angiosperms		
	A. angustus	***M. polymorpha*[1]**	***P. patens***	***S. moellendorffii***	***P. minima*[2]**	***P. abies***	***A. trichopoda***	***O. sativa***	***A. thaliana***
miR156/157	+	-	+	+	+	+	+	+	+
miR159/319	+	+	+	+	+	+	+	+	+
miR160	+	+	+	+	+	+	+	+	+
miR162	-	-	-	-	+	+	-	+	+
miR164	-	-	-	-	+	+	+	+	+
miR165/166	+	+	+	+	+	+	+	+	+
miR167	-	-	-	-	+	+	+	+	+
miR168	-	-	-	-	+	+	+	+	+
miR169	-	-	-	-	+	+	+	+	+
miR170/171	+	+	+	+	+	+	+	+	+
miR172	-	-	-	-	+	-	+	+	+
miR390	-	+	+	-	+	+	+	+	+
miR395	-	-	+	-	+	+	+	+	+
miR408	+	+	+	+	+	+	-	+	+
miR477	+	-	+	-	+	-	+	-	-
miR535	+	-	+	-	+	+	+	-	-
miR536	+	+	+	+	+	+	-	-	-

P. minima* = *Pleopeltis minima*, *P. abies* = *Picea abies*, *A. trichopoda* = *Amborella trichopoda*, *O. sativa* = *Oryza sativa

[1]Tsuzuki, M. et al. Profiling and characterization of small RNAs in the liverwort, *Marchantia polymorpha*, belonging to the first diverged land plants. *Plant Cell Physiol.* **57**, 359–372 (2016).

[2]Berruezo, F. et al. Sequencing of small RNAs of the fern *Pleopeltis minima* (Polypodiaceae) offers insight into the evolution of the microrna repertoire in land plants. *PLoS One* **12**, e0177573 (2017).

The information about miRNAs of other investigated species come from miRBase (http://www.mirbase.org/).

Supplementary Table 13. Gene sets used in this study.

Species	Abbrevation	ID	Original genes	Used genes	Database	Version	Assembly
Arabidopsis thaliana	*A. thaliana*	ATHA	48,315	27,411	NCBI	TAIR10	GCF_000001735.3
Genlisea aurea	*G. aurea*	GAUR	17,685	17,685	NCBI	GenAur_1.0	GCA_000441915.1
Vitis vinifera	*V. vinifera*	VVIN	41,208	25,676	NCBI	12X	GCF_000003745.3
Oryza sativa Japonica Group	*O. sativa*	OSAT	41,070	27,912	NCBI	IRGSP-1.0	GCF_001433935.1
*Phalaenopsis equestris**	*P. equestris**	PEQU	29,431	29,431	Cai et al., 2014	-	-
Zostera marina	*Z. marina*	ZMAR	20,648	20,421	NCBI	v.2.1	GCA_001185155.1
Amborella trichopoda	*A. trichopoda*	ATRI	26,846	26,846	JGI	v1.0	-
Picea abies	*P. abies*	PABI	26,437	26,437	http://congenie.org/	Pabies1.0	-
Selaginella moellendorffii	*S. moellendorffii*	SMOE	22,273	22,273	JGI	V1.0	-
Physcomitrella patens	*P. patens*	PPAT	35,934	35,796	NCBI	V1.1	GCF_000002425.3
Marchantia polymorpha	*M. polymorpha*	MPOL	19,287	19,287	JGI	v3.1	-
Anthoceros angustus	***A. angustus***	**AANG**	**14,629**	**14,629**	**This study**	-	-
Chara braunii[#]	*C. braunii*	CBRA	35,885	22,776	https://bioinformatics.psb.ugent.be/gdb/Chara_braunii/	-	-
Klebsormidium nitens	*K. nitens*	KNIT	16,283	16,044	NCBI	ASM70883v1	GCA_000708835.1
Volvox carteri f. nagariensis	*V. carteri*	VCAR	14,436	14,434	NCBI	V1.0	GCF_000143455.1
Chlamydomonas reinhardtii	*C. reinhardtii*	CREI	17,741	17,741	JGI	V5.5	-
Ulva mutabilis	*U. mutabilis*	UMUT	12,924	12,924	https://bioinformatics.psb.ugent.be/gdb/ulva/	-	-
Coccomyxa subellipsoidea	*C. subellipsoidea*	CSUB	9,629	9,629	JGI	v2.0	-
Chlorella variabilis	*C. variabilis*	CVAR	9,780	9,780	NCBI	v1.0	GCF_000147415.1

*Cai, J., et al. The genome sequence of the orchid *Phalaenopsis equestris*. *Nat. genet*. **47**, 65–72 (2015).

[#]Remvoing the sequences with at least 50% with TE evidence accoring to the genome paper of *Chara braunii*. Nishiyama, T., et al. The *Chara* genome: secondary complexity and implications for plant terrestrialization. *Cell* **174**, 448–464 (2018).

Supplementary Table 14. Summary of clustered gene families by OrthoMCL among 19 species. The corresponding full latin name of each species is shown in Supplementary Table 13.

Species	Gene number	Unclustered genes	Genes in families	Gene family number	Unique families (genes in unique families)	Average genes per family
A. thaliana	27,411	4,206	23,205	12,301	1,078(4,764)	1.886
G. aurea	17,685	4,434	13,251	10,060	258(707)	1.317
V. vinifera	25,676	2,172	23,504	12,399	593(3,446)	1.896
O. sativa	27,912	4,647	23,265	11,950	1,300(5,565)	1.947
P. equestris	29,431	9,156	20,275	11,640	1,091(4,514)	1.742
Z. marina	20,421	3,888	16,533	10,470	581(2,507)	1.579
A. trichopoda	26,846	8,326	18,520	11,800	1,092(4,945)	1.569
P. abies	26,437	5,748	20,689	8,821	1,689(7,892)	2.345
S. moellendorffii	22,273	4,384	17,889	9,585	1,660(6,911)	1.866
P. patens	35,796	9,722	26,074	9,566	1,334(11,318)	2.726
M. polymorpha	19,287	6,292	12,995	8,944	565(2,865)	1.453
A. angustus	**14,629**	**2,997**	**11,632**	**8,141**	**497(1,845)**	**1.429**
C. braunii	22,776	5,220	17,556	6,252	618(9,370)	2.808
K. nitens	16,044	3,807	12,237	8,302	809(3,460)	1.474
V. carteri	14,434	3,978	10,456	7,934	391(1,849)	1.318
C. reinhardtii	17,741	5,991	11,750	8,669	639(2,587)	1.355
U. mutabilis	12,924	5,289	7,635	5,363	467(2,186)	1.424
C. subellipsoidea	9,629	2,614	7,015	5,850	251(877)	1.199
C. variabilis	9,780	2,629	7,151	5,857	245(973)	1.221

Supplementary Table 15. GO term enrichment of *Anthoceros angustus*-specific gene families.

GO ID	GO term	GO class	P-value	Adjusted P-value	x1	x2	n	N	GO level
GO:0045735	nutrient reservoir activity	MF	3.69E-13	5.96E-10	52	144	1845	14629	2
GO:0004097	catechol oxidase activity	MF	1.16E-09	1.88E-06	17	27	1845	14629	6
GO:0016679	oxidoreductase activity, acting on diphenols and related substances as donors	MF	9.53E-09	1.54E-05	18	33	1845	14629	4
GO:0009055	electron carrier activity	MF	3.33E-07	0.000538	70	305	1845	14629	2
GO:0016872	intramolecular lyase activity	MF	3.52E-06	0.005674	9	13	1845	14629	4

Note: N: total gene number; n: gene number in the list; x1: gene number with a GO term in the list; x2: gene number with a GO term in total. P-value was calculated via chi-square test. Fisher's exact test was used when any expected value of count was below 5, which will make the chi-square test inaccurate. The bonferroni was used for P-value adjustments.

Supplementary Table 16. The KEGG pathway enrichment of *Anthoceros angustus*-specific gene families.

Map ID	Map Title	P-value	Adjusted P-value	x	y	n	N
map00900	terpenoid backbone biosynthesis	2.00E-16	1.14E-14	41	82	1845	14629
map00908	zeatin biosynthesis	1.56E-14	8.91E-13	28	46	1845	14629
map00950	isoquinoline alkaloid biosynthesis	9.83E-08	5.61E-06	18	37	1845	14629
map00906	carotenoid biosynthesis	3.82E-07	2.18E-05	28	82	1845	14629
map00941	flavonoid biosynthesis	3.13E-06	0.000179	24	71	1845	14629
map01110	biosynthesis of secondary metabolites	6.03E-06	0.000344	148	832	1845	14629
map00350	tyrosine metabolism	1.63E-05	0.00093	21	63	1845	14629

Note: N: total gene number; n: gene number in the list; x: gene number with a KEGG term in the list; y: gene number with a KEGG term in total. P-value was calculated via chi-square test. Fisher's exact test was used when any expected value of count was below 5, which will make the chi-square test inaccurate. The bonferroni was used for P-value adjustments.

Supplementary Table 17. Single-copy gene families *vs* multiple-copy gene families in 19 plant genomes assessed by OrthoMCL. The corresponding full latin name of each species is shown in **Supplementary Table 13**.

Species	Single copy	Percent (%)	2-5 copies	Percent (%)	>5 copies	Percent (%)
A. thaliana	7,267	59.08	4,647	37.78	387	3.15
G. aurea	7,980	79.32	2,015	20.03	65	0.65
V. vinifera	8,288	66.84	3,697	29.82	414	3.34
O. sativa	7,054	59.03	4,455	37.28	441	3.69
P. equestris	7,941	68.22	3,496	30.03	203	1.74
Z. marina	7,481	71.45	2,796	26.7	193	1.84
A. trichopoda	8,966	75.98	2,580	21.86	254	2.15
P. abies	5,042	57.16	3,223	36.54	556	6.3
S. moellendorffii	6,203	64.72	3,012	31.42	370	3.86
P. patens	4,647	48.58	4,554	47.61	365	3.82
M. polymorpha	7,493	83.78	1,267	14.17	184	2.06
A. angustus	6,794	83.45	1,244	15.28	103	1.27
C. braunii	4,919	78.68	1,090	17.43	243	3.89
K. nitens	6,946	83.67	1,191	14.35	165	1.99
V. carteri	7,113	89.65	699	8.81	122	1.54
C. reinhardti	7,468	86.15	1,091	12.59	110	1.27
U. mutabilis	4,591	85.61	675	12.59	97	1.81
C. subellipsoidea	5,207	89.01	603	10.31	40	0.68
C. variabilis	5,243	89.52	564	9.63	50	0.85

Supplementary Table 18. Bryophyte genome and transcriptome data used in phylogenomic analyses.

Lineage	Class	Subclass	Family	Species	ID
Hornworts (Anthocerotophyta)	Anthocerotopsida	Anthocerotidae	Anthocerotaceae	*Anthoceros angustus**	AANG
		Dendrocerotidae	Dendrocerotaceae	*Phaeomegaceros coriaceus*	AKXB
				Megaceros flagellaris	UCRN
				Nothoceros aenigmaticus	DXOU
		Notothylidae	Notothyladaceae	*Phaeoceros carolinianus*	WCZB
				Paraphymatoceros hallii	FAJB
	Leiosporocerotopsida		Leiosporocerotaceae	*Leiosporoceros dussi*	ANON
Liverworts (Marchantiophyta)	Marchantiopsida	Marchantiidae	Marchantiaceae	*Marchantia polymorpha subsp. ruderalis**	MPOL
			Ricciaceae	*Ricciocarpos natans*	WJLO
			Lunulariaceae	*Lunularia cruciata*	TXVB
			Sphaerocarpaceae	*Sphaerocarpos texanus*	HERT
	Jungermanniopsida	Pelliidae	Pelliaceae	*Pellia neesiana*	JHFI
		Metzgeriidae	Metzgeriaceae	*Metzgeria crassipilis*	NRWZ
		Jungermanniidae	Porellaceae	*Porella pinnata*	UUHD
			Ptilidiaceae	*Ptilidium pulcherrimum*	HPXA
			Lepidoziaceae	*Bazzania trilobata*	WZYK
Mosses (Bryophyta)	Bryopsida	Funariidae	Funariaceae	*Physcomitrella patens**	PPAT
		Timmiidae	Timmiaceae	*Timmia austriaca*	ZQRI
		Bryidae	Hedwigiaceae	*Hedwigia ciliata*	YWNF
		Bryidae	Thuidiaceae	*Thuidium delicatulum*	EEMJ
		Dicranidae	Ditrichaceae	*Ceratodon purpureus*	FFPD

	Buxbaumiidae	Buxbaumiaceae	*Buxbaumia aphylla*	HRWG
Tetraphidopsida		Tetraphidaceae	*Tetraphis pellucida*	HVBQ
Polytrichopsida		Polytrichaceae	*Polytrichum commune*	SZYG
Sphagnopsida		Sphagnaceae	*Sphagnum lescurii*	GOWD

The data of the species with asterisk are genome sequences, and the others are transcriptome sequences from 1KP database (http://www.onekp.com/public_read_data.html).

Supplementary Table 19. Calibrations used in the age estimates of lineages in green plants. The nodes are labeled on the phylogenetic tree in **Supplementary Figure 10**.

Node	Clade	Lineage1	Lineage2	Min. date (Ma)	Max. date (Ma)	References
1	Viridiplantae	Chlorophyta	Streptophyta	725	1200	Becker & Marin, 2009
2	Embryophyta	Bryophytes	Tracheophyta	472	515.5	Rubinstein et al., 2010
3	Bryophyta	Sphagnopsida+ Polytrichopsida	Tetraphidopsida+Bryopsida	330.7	515.5	Morris et al., 2018
4	Marchantiophyta	Jungermanniopsida	Marchantiopsida	405	515.5	Morris et al., 2018
5	Marchantiopsida	*Marchantia*	Other Marchantiopsida species	227	515.5	Morris et al., 2018
6	Unnamed	*Anthoceros*	Other hornworts excluding *Leiosporoceros*	114.86	515.5	Villarreal & Renner, 2012
7	Tracheophyta	Lycopodiophyta	Euphyllophyta	420.7	451	Morris et al., 2018
8	Angiospermae	Amborellales	Other angiopserms	136	247.2	Magallón et al., 2013
9	Unnamed	Vitales	Rosids+Asterids	85.8	128.63	Morris et al., 2018

Becker, B. & Marin, B. Streptophyte algae and the origin of embryophytes. *Ann. Bot.* **103**, 999–1004 (2009).

Rubinstein, C. V., Gerrienne, P., de la Puente, G. S., Astini, R. A., Steemans, P. Early Middle Ordovician evidence for land plants in Argentina (eastern Gondwana). *New Phytol.* **188**, 365–369 (2010).

Morris, J. L. et al., The timescale of early land plant evolution. *Proc. Natl Acad. Sci. U S A.* **115**, E2274–E2283 (2018).

Villarreal, J. C. & Renner S. S. Hornwort pyrenoids, carbon-concentrating structures, evolved and were lost at least five times during the last 100 million years. *Proc. Natl Acad. Sci. USA* **109**, 18873–18878 (2012).

Magallón, S., Hilu, K. W., Quandt, D. Land plant evolutionary timeline: gene effects are secondary to fossil constraints in relaxed clock estimation of age and substitution rates. *Am. J. Bot.* **100**, 556–573 (2013).

Supplementary Table 20. The 95% highest posterior density (HPD) intervals of age estimates for lineages in green plants. The nodes are labeled on the dated phylogenetic tree in **Supplementary Figure 11**.

**Node**	**Clade**	**95% HPD age (Mya)**
A	Viridiplantae	670.6–1313.7
B	Streptophyta	602.9–1095.0
C	Embryophyta	478.6–516.6
D	Bryophytes	457.1–502.9
E	Bryophyta	318.4–381.3
F	Marchantiophyta	396.4–432.1
G	Anthocerotophyta	179.3–384.6
H	Tracheophyta	422.4–451.7

Supplementary Tables (excel)

Supplementary Table 21. Transcription factor numbers of 19 green plants identified by iTAK. (excel)

Supplementary Table 22. MADS-box genes uesd in this study. (excel)

Supplementary Table 23. Numbers of PPR genes and organellar RNA editing sites in various plants.

Supplementary Table 24. List of PPR proteins in *Anthoceros angustus*. (excel)

Supplementary Table 25. List of cupin proteins used in the analysis. (excel)

Supplementary Table 26. Summary of cytochrome P450 gene number in green plants. (excel)

Supplementary Table 27. List of cytochrome P450 proteins used in the analysis. (excel)

Supplementary Table 28. KEGG annotation of cytochrome P450 genes in *Anthoceros angustus*. (excel)

Supplementary Table 29. The summary of tandemly arrayed genes (TAGs) in the *Anthoceros angustus* genome. (excel)

Supplementary Table 30. Anotation of tandemly arrayed genes in the *Anthoceros angustus* genome. (excel)

Supplementary Table 31. Summary of HGT genes in *Anthoceros angustus*. (excel)

Supplementary Table 32. Fuctional annotation of the *Anthoceros angustus* protein-coding genes. (excel)
