# Supplementary material for: The hornwort genome and early land plant evolution
Source: Nat Plants. 2020 Feb 10;6(2):107–18. doi: 10.1038/s41477-019-0588-4 (PMC7027989; doi:10.1038/s41477-019-0588-4)
Supplement: Supplementary file 2 — Reporting Summary [file 41477_2019_588_MOESM2_ESM.pdf]

nature research

Corresponding author(s): Zhi-Duan Chen, Zhong-Jian Liu, Yves Van de Peer, and Shou-Zhou Zhang

Last updated by author(s): Dec 17, 2019

Reporting Summary

Nature Research wishes to improve the reproducibility of the work that we publish. This form provides structure for consistency and transparency in reporting. For further information on Nature Research policies, see Authors & Referees and the Editorial Policy Checklist.

Statistics

For all statistical analyses, confirm that the following items are present in the figure legend, table legend, main text, or Methods section.

n/a	Confirmed	
☐	☒	The exact sample size (*n*) for each experimental group/condition, given as a discrete number and unit of measurement
☐	☒	A statement on whether measurements were taken from distinct samples or whether the same sample was measured repeatedly
☐	☒	The statistical test(s) used AND whether they are one- or two-sided<br>*Only common tests should be described solely by name; describe more complex techniques in the Methods section.*
☒	☐	A description of all covariates tested
☐	☒	A description of any assumptions or corrections, such as tests of normality and adjustment for multiple comparisons
☐	☒	A full description of the statistical parameters including central tendency (e.g. means) or other basic estimates (e.g. regression coefficient) AND variation (e.g. standard deviation) or associated estimates of uncertainty (e.g. confidence intervals)
☐	☒	For null hypothesis testing, the test statistic (e.g. *F*, *t*, *r*) with confidence intervals, effect sizes, degrees of freedom and *P* value noted<br>*Give P values as exact values whenever suitable.*
☒	☐	For Bayesian analysis, information on the choice of priors and Markov chain Monte Carlo settings
☒	☐	For hierarchical and complex designs, identification of the appropriate level for tests and full reporting of outcomes
☒	☐	Estimates of effect sizes (e.g. Cohen's *d*, Pearson's *r*), indicating how they were calculated

Our web collection on statistics for biologists contains articles on many of the points above.

Software and code

Policy information about availability of computer code

Data collection	1.We constructed libraries with insert sizes from 170 bp to 40 kb for whole-genome shotgun sequencing using Illumina HiSeq 2000.<br>2. We also constructed a genomic DNA library for Oxford Nanopore sequencing.
Data analysis	Software used are listed as follows:BLASTP (ncbi-BLAST v2.2.28), BLASTN (ncbi-BLAST v2.2.28), TBLASTN (ncbi-BLAST v2.2.28), Nextdenovo (V2.0), Pilon (v1.22), SSPACE (v3.0), BUSCO (v3), Trimmomatic (v0.33), Trinity (v2.5.1), TransDecoder (v5.0.2), Tandem Repeats Finder (v4.09), RepeatMasker (v4.1.0), LTR_FINDER (v1.0.2), PILER (v1.3.4.), RepeatModeler (v1.0.3), AUGUSTUS (v2.5.5), GlimmerHMM (v3.0.1), GeneWise (v2.4.1), MAKER (v1.0), TopHat (v2.1.1), Cufflinks (v2.2.1), miREvo (v1.2), tRNAscan-SE (v1.3.1), INFERNAL (v1.1), OrthoMCL (v2.0), MAFFT (version 7), TranslatorX (v0.9), RAxML (v7.2.3), PAML (v4.7), PHYLIP (v3.695), wgd (v3.0), I-ADHoRe 3.0, iTAK (version 1.7), Mesquite (version 3.51), HMMER (v 3.1b2), CIPRES Science Gateway (V. 3.3), Genesis (v3.0), CodonO.

For manuscripts utilizing custom algorithms or software that are central to the research but not yet described in published literature, software must be made available to editors/reviewers. We strongly encourage code deposition in a community repository (e.g. GitHub). See the Nature Research guidelines for submitting code & software for further information.

Data

Policy information about availability of data

All manuscripts must include a data availability statement. This statement should provide the following information, where applicable:

- Accession codes, unique identifiers, or web links for publicly available datasets
- A list of figures that have associated raw data
- A description of any restrictions on data availability

The A. angustus genome project has been deposited at the NCBI under the BioProject number PRJNA543716. The genome sequencing data were deposited in the Sequence Read Archive (SRA) database under the accession number SRR9696346. The A. angustus transcriptome project has been deposited at the NCBI under BioProject PRJNA543724. The transcriptome sequencing data were deposited in the Sequence Read Archive (SRA) database under the accession number SRR9662965. The assembled genome sequences, gene models, miRNA data are available via DRYAD (https://doi.org/10.5061/dryad.msbcc2ftv). All data that

support the findings of this study are also available from the corresponding authors upon request.

Field-specific reporting

Please select the one below that is the best fit for your research. If you are not sure, read the appropriate sections before making your selection.

☒ Life sciences ☐ Behavioural & social sciences ☐ Ecological, evolutionary & environmental sciences

For a reference copy of the document with all sections, see nature.com/documents/nr-reporting-summary-flat.pdf

Life sciences study design

All studies must disclose on these points even when the disclosure is negative.

Sample size

We sequenced a single hornwort plant, and no statistical methods were used to predetermine sample sizes. For comparative genome analyses, the gene sequences of Anthoceros angustus and other 18 plant species were used (Supplementary Table 13), including seven angiosperms (Arabidopsis thaliana, Genlisea aurea, Vitis vinifera, Oryza sativa, Phalaenopsis equestris, Zostera marina and Amborella trichopoda), one gymnosperm (Picea abies), one lycophyte (Selaginella moellendorffii), three bryophytes (Physcomitrella patens, Marchantia polymorpha and Anthoceros angustus), two charophytes (Chara braunii and Klebsormidium nitens), five chlorophytes (Volvox carteri, Chlamydomonas reinhardtii, Ulva mutabilis, Coccomyxa subellipsoidea and Chlorella variabilis). This sampling covered all the major lineages of green plants and could present the backbone of green plant evolution.

Data exclusions

Lines 416-456, 466-472: The prokaryotic sequences and organellar sequences were removed from sequencing data and pre-assembled genome data. There are prokaryotic sequences and organellar sequences that involved in the genome sequencing data. Exclusion of the contamination from foreign DNA sequences and organellar sequences is the prerequisite for accurate genome assembly. Through choose of high-abundance k-mer reads, error-correction and MEGABLAST check, 3,78 Gb high-quality clean reads of Nanopore sequencing remained for A. angustus genome assembly.
Lines 521-522: We excluded annotations only characterized as hypothetical/predicted protein, since these proteins could not be treated as really functionally annotated ones.
Lines 542-543: During the comparative analysis, we chose the longest transcript to represent each gene and removed mitochondrial and chloroplast genes, since the used genome datasets include multiple transcripts and organellar genes that might complicate the comparative analysis.
Lines 617-618: The mean gene family size was calculated for all gene families, excluding orphans and species-specific families, since these genes are unique to individual species and do not have orthologs in other species for comparison.
Lines 624-627: During the gene family expansion identification, transposon-derived gene families were removed, since the distribution of such families is likely to be a consequence of the gene models derived from a repeat-masked genome sequence and therefore may be artefactual.
Lines 638-640: The sequences without support of transcript evidence were excluded from the HGT candidates, since these sequences might be contaminated ones but not real HGT genes.

Replication

The spore germination experiment was repeated three times independently. The DAPI staining experiment was repeated three times independently.

Randomization

We picked up spores randomly for germination experiments. We selected regions of the gametophytes randomly for DAPI staining.

Blinding

We sequenced a single hornwort plant, and no control group is referred here. Blinding is not applicable in this study.

Reporting for specific materials, systems and methods

We require information from authors about some types of materials, experimental systems and methods used in many studies. Here, indicate whether each material, system or method listed is relevant to your study. If you are not sure if a list item applies to your research, read the appropriate section before selecting a response.

Materials & experimental systems

n/a	Involved in the study	
☒	☐	Antibodies
☒	☐	Eukaryotic cell lines
☒	☐	Palaeontology
☒	☐	Animals and other organisms
☒	☐	Human research participants
☒	☐	Clinical data

Methods

n/a	Involved in the study	
☒	☐	ChIP-seq
☒	☐	Flow cytometry
☒	☐	MRI-based neuroimaging
